# The landscape of patellofemoral arthroplasty research: a bibliometric analysis

**DOI:** 10.1186/s42836-023-00215-1

**Published:** 2023-12-03

**Authors:** Yao Yang, Yuan Chen, Yingjie Wang, Junjie Wang, Baoliang Lu, Wanbo Zhu, Ning Yang, Junchen Zhu, Chen Zhu, Xianzuo Zhang

**Affiliations:** 1 Department of Orthopedics, Division of Life Sciences and Medicine, The First Affiliated Hospital of USTC, University of Science and Technology of China, Hefei, 230001 China; 2 Department of Orthopedics, The Second Affiliated Hospital of Anhui University of Chinese Medicine, Hefei, 230061 China; 3 Department of Orthopedics, Graduate School of Bengbu Medical College, Bengbu, 233030 China; 4 Department of Orthopedics, Shanghai Jiao Tong University Affiliated Sixth People’s Hospital, Shanghai Jiao Tong University, Shanghai, 200233 China

**Keywords:** Patellofemoral arthroplasty, Osteoarthritis, Knee, Cooperative networks, Bibliometric analysis

## Abstract

**Purpose:**

Patellofemoral arthroplasty (PFA) was shown to be a potentially effective surgical technique for isolated patellofemoral osteoarthritis but varying reports on PFA-related implant failure and complications have rendered the procedure controversial. This study aimed to identify impactful publications, research interests/efforts, and collaborative networks in the field of PFA research.

**Methods:**

The study used the Web of Science Core Collection (WOSCC) database, Medline, Springer, BIOSIS Citation Index, and PubMed to retrieve relevant publications on PFA research published between 1950–2022. Statistical tests in R software were used for analysis while VOSviewer, Bibliometrix, and CiteSpace were employed for data visualization.

**Results:**

Two hundred forty-one articles were analyzed with the number of published papers increasing over time. *Knee* was the most frequent journal and *Clinical Orthopaedics and Related Research* was the most cited journal. Clinical outcomes, such as prosthesis survival, revision, and complications, were researched most frequently as demonstrated by keyword analysis. The United States was the top contributor to cooperative networks, followed by the United Kingdom while Technical University Munich formed close ties among authors.

**Conclusion:**

Publications on PFA research have witnessed a notable surge. They primarily came from a limited number of centers and were characterized by low-level evidence. The majority of studies primarily focused on the clinical outcomes of PFA, while revision of PFA and patient satisfaction have emerged as new research areas.

**Supplementary Information:**

The online version contains supplementary material available at 10.1186/s42836-023-00215-1.

## Introduction

Patellofemoral osteoarthritis is a highly prevalent form of osteoarthritis, affecting approximately 25% of those aged 50 and over [1, 2]. Due to the increasing demand for a better quality of life and the growing prevalence of knee osteoarthritis among younger individuals, the incidence of patellofemoral arthroplasty (PFA) has been steadily on the rise [3–6]. PFA offers several advantages over other surgical options, such as total knee arthroplasty, since it is a less invasive and bone-conserving procedure that preserves the natural anatomy of the knee joint [7, 8], thereby minimizing complications and improving long-term outcomes. Moreover, PFA has been demonstrated to provide superior pain relief, functional improvement, and patient satisfaction in comparison to non-surgical treatments such as physical therapy and medication. Despite the success of PFA in treating patellofemoral osteoarthritis, the procedure remains somewhat controversial, with some studies reporting high rates of implant failure and complications [9–12], while others demonstrating good long-term outcomes [13, 14]. Similar to any surgical procedure, the success of PFA is contingent upon numerous factors, including patient selection, implant design, and surgical technique.

For all that mounting interest in PFA, a comprehensive analysis of current research in the field, particularly from a bibliometric perspective, is lacking. Bibliometric analysis is a quantitative method that utilizes statistical and mathematical techniques to study citation and publication patterns in a specific field [15]. Bibliometric analysis has been conducted previously in the orthopedic research field, including total hip and knee arthroplasty [16, 17], periprosthetic joint infection [18], and a variety of other orthopedic diseases [19–22]. Despite this, a lack of bibliometric analysis on PFA persists.

In this study, we aimed to identify the most impactful publications in PFA research and conduct an analysis of their characteristics. Our goal was to provide insights into the current state of the field, identify research gaps, and guide future research directions. We hope that this study will facilitate the development of evidence-based guidelines for the application of PFA in the management of patellofemoral osteoarthritis, thereby improving the quality of care for patients with these conditions.

## Materials and methods

### Data sources and search strategies

The data were obtained from the Web of Science Core Collection (WOSCC) database, Medline, Springer, BIOSIS Citation Index, and PubMed. Our search strategy consists of “Patellofemoral arthroplasty” OR “Patellofemoral replacement” OR “Patellofemoral joint arthroplasty” OR “Patellofemoral joint replacement”. The publication time of the articles was limited to the period from 1950 to 2022. To avoid bias due to frequent database updates, all our searches and export data were completed on September 19, 2023. The searched and included bibliographies are recorded in Supporting Information, as Tables S1-S6.

### Article screening

Bibliographical references from various databases were consolidated to remove duplication. Non-English papers were excluded from the analysis. Papers with unsuitable article types were further excluded by manual persusing the abstracts (including finite element analyses, mathematical analysis, etc.), remaining publications were further screened by full-text reading. Articles were screened independently by two reviewers. When there was disagreement, a third person performed the consistency assessment. Included item entries for further data collection and extraction.

### Data collection and extraction

The articles selected for this study were imported into a reference management software package for further analysis. From these articles, the following data were extracted: author information, including author names, institutions, countries, and corresponding or reprint authors; publication information, such as journal titles, publication year and impact factors; article information, including keywords, major topics, language and level of evidence and citation information, including total number of citations and cited references. The level of evidence was evaluated in accordance with the guidelines set forth by the *Journal of Bone and Joint Surgery* [23].

### Statistics analysis

The Kolmogorov-Smirnov test was used to assess the normality of the distribution for individual variables. Normally distributed data were presented as mean ± standard deviation, whereas non-normally distributed data were expressed as medians (minimum, maximum). Correlation analysis was performed using Spearman’s test and statistical significance was determined at *P* < 0.05 (two-sided). R software version 4.2.2 (https://www.r-project.org/about.html) was used for all statistical analyses. To analyze trend changes in the time series data, the Mann-Kendall test was conducted using MATLAB software (version 2021a, MathWorks Inc, Natick, MA, USA), with the trend change rate quantified by the uncertainty factor (UF). A positive UF value indicates an increasing trend, while a negative value denotes a decreasing trend.

### Data visualization

Histograms and line graphs are used to present overall publication and citation trends, with subsequent trends predicted based on these findings. The relationship between the level of evidence and citations was explored using a box plot, while clustered linkage network plots were utilized to demonstrate relationships between keywords. Line graphs were employed to display the time of occurrence and outbreak of different keywords. A world map marked with shades of color represents the volume of articles published by different countries. Furthermore, to investigate collaboration among different entities, a collaborative network coupled with full-count bibliographic analysis was performed. The data visualization was achieved by using several tools, including VOSviewer [24] version 1.6.18 (Centre for Science and Technology Research at Leiden University, Leiden, the Netherlands), R package Bibliometrix [25] version 4.0 (http://www.bibliometrix.org), CiteSpace [26] version 6.1.3 (https://citespace.podia.com/), MapChart (https://www.mapchart.net), Charticulator (https://charticulator.com), and Microsoft Excel (Microsoft Corporation, Redmond, WA, USA).

## Results

### Overview of included studies

**Fig. 1:**
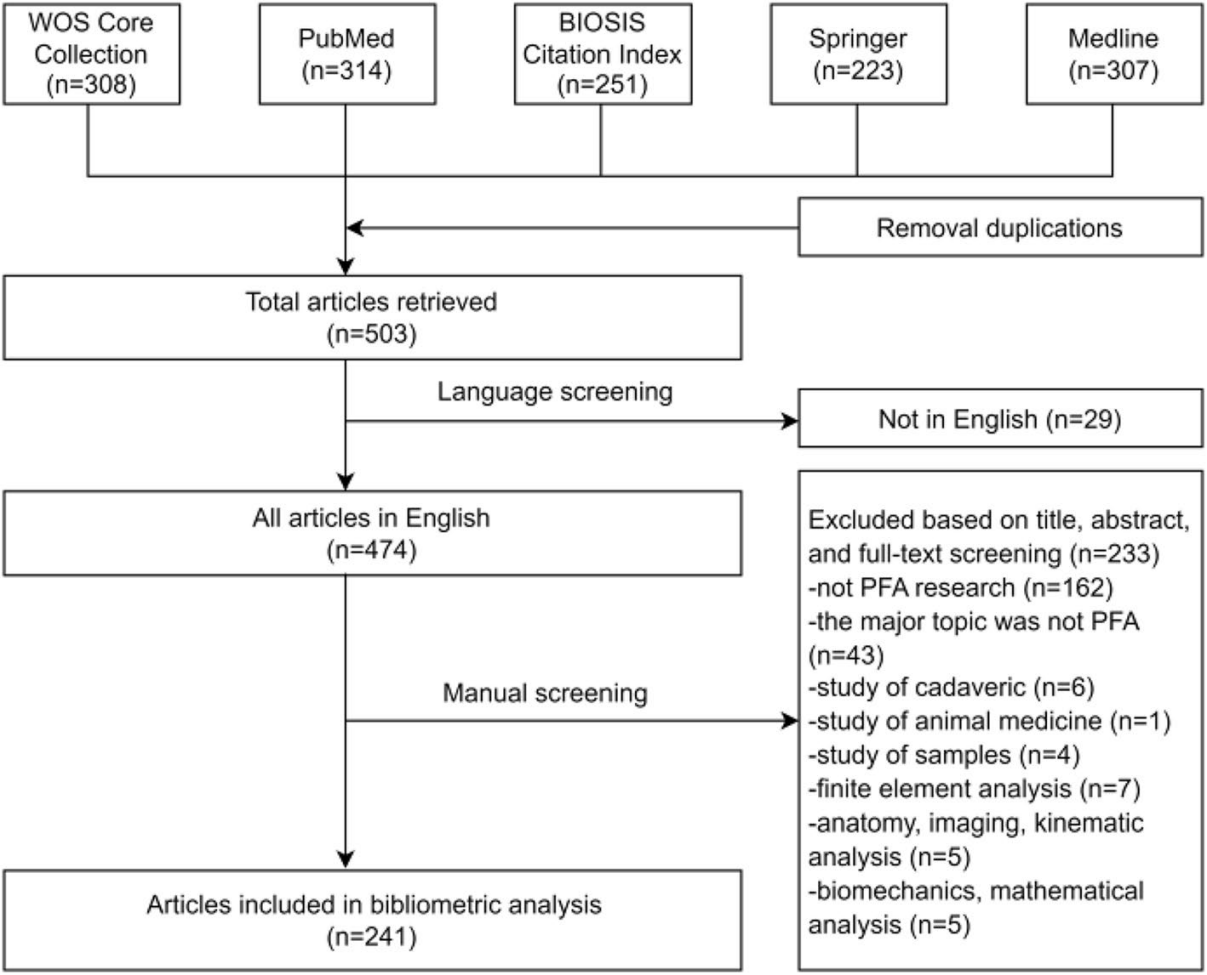
Flow chart for inclusion and exclusion of publications

After removal of duplicates, a total of 503 bibliographical references were left. Twenty-nine non-English entries were excluded. Manual screening led to the exclusion of 223 entries, 241 articles related to patellofemoral arthroplasty from the year 1979 until 2022 remained. The process for the selection and inclusion of the title catalog is illustrated in Fig. 1.

**Fig. 2:**
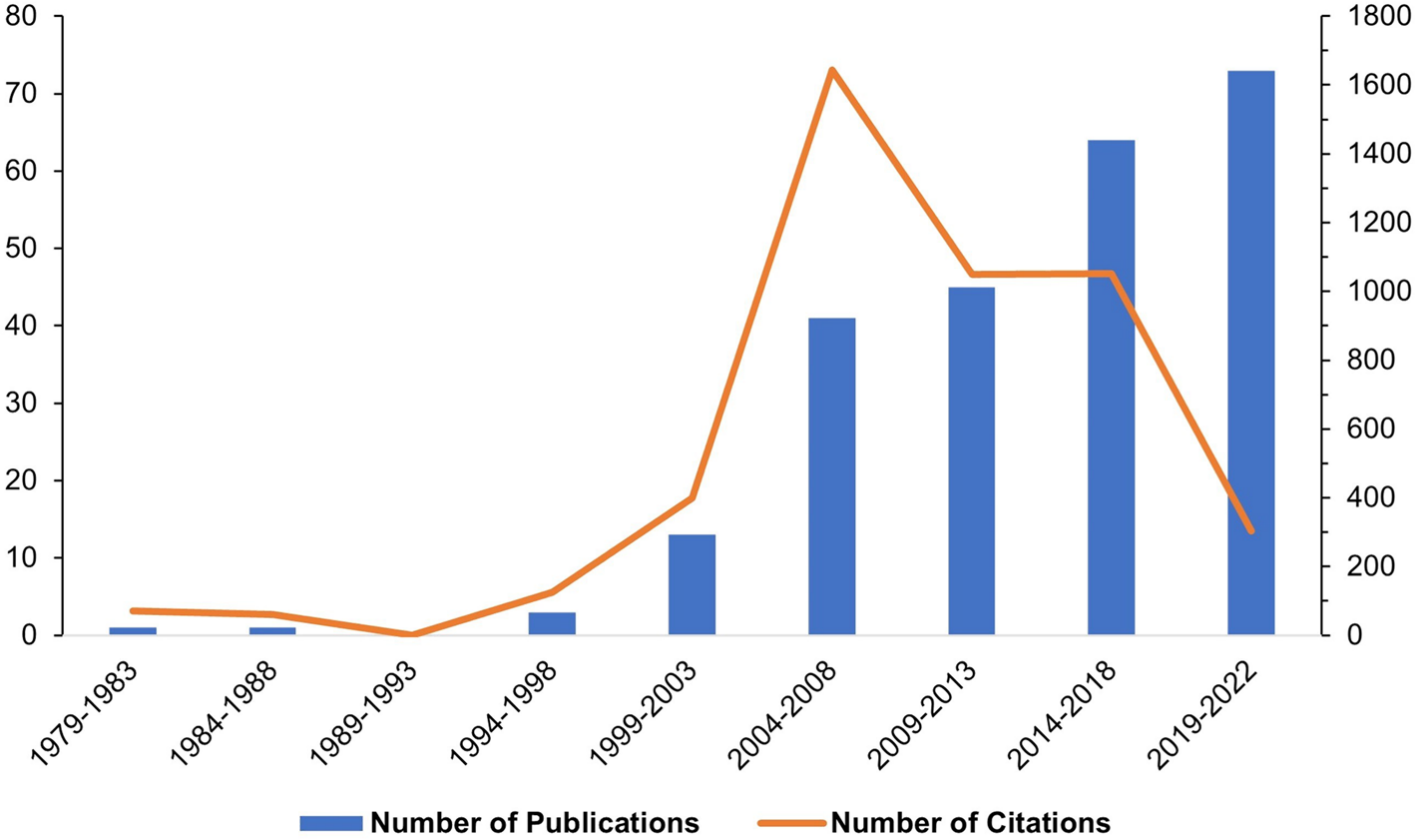
Number of publications and citations per five years

**Fig. 3:**
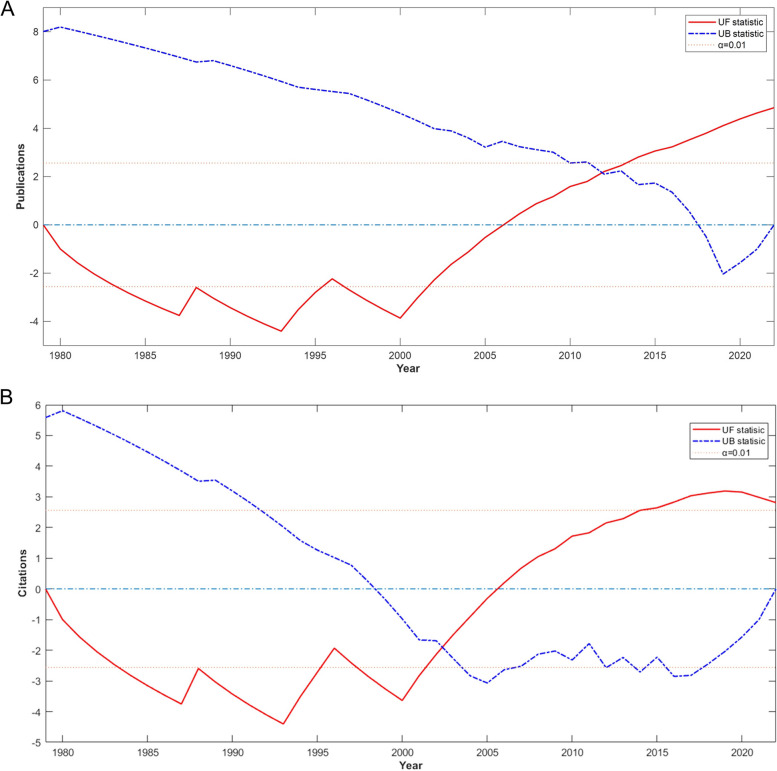
Mann-Kendall trend tests for publications **A** and citations **B** the UF curve represents the trend of change while the UB curve is its inverse series. A positive UF value indicates an increasing trend, whereas an inverse trend denotes a decreasing trend. The significance level was defined as 0.01 with a value of ±2.56. A UF value greater than 2.56 suggests a considerable upward trend, and roughly vice versa similarly. The intersection point within the confidence interval (±2.56) was taken as the mutation point

The publications and citations spanned a period of time of five years (Fig. 2). There was an overall increase in published papers during the period covered. However, although the number of citations initially rose, with a peak appearing around 2004 and 2008, which was followed by a gradual decline. Notably, the initial years of the 21st century saw a sudden surge in both publications and citations. The Mann-Kendall mutation test indicated that both the issuance and citation volumes exhibited mutational phenomena. The mutation points for the number of posts occurred around 2012, when the UF and UB curves intersected. The UF curve revealed an increasing trend in the number of posts after 2006, displaying a significant spike after 2013 (significance level above *α* = 0.01) (see Fig. 3A). Similarly, the abrupt change in citations was observed around 2002, where the UF and UB curves met. The UF curve also showed an increasing trend in citations after 2005, with a significant rise after 2015 (significance level above *α* = 0.01) (see Fig. 3B).

**Fig. 4:**
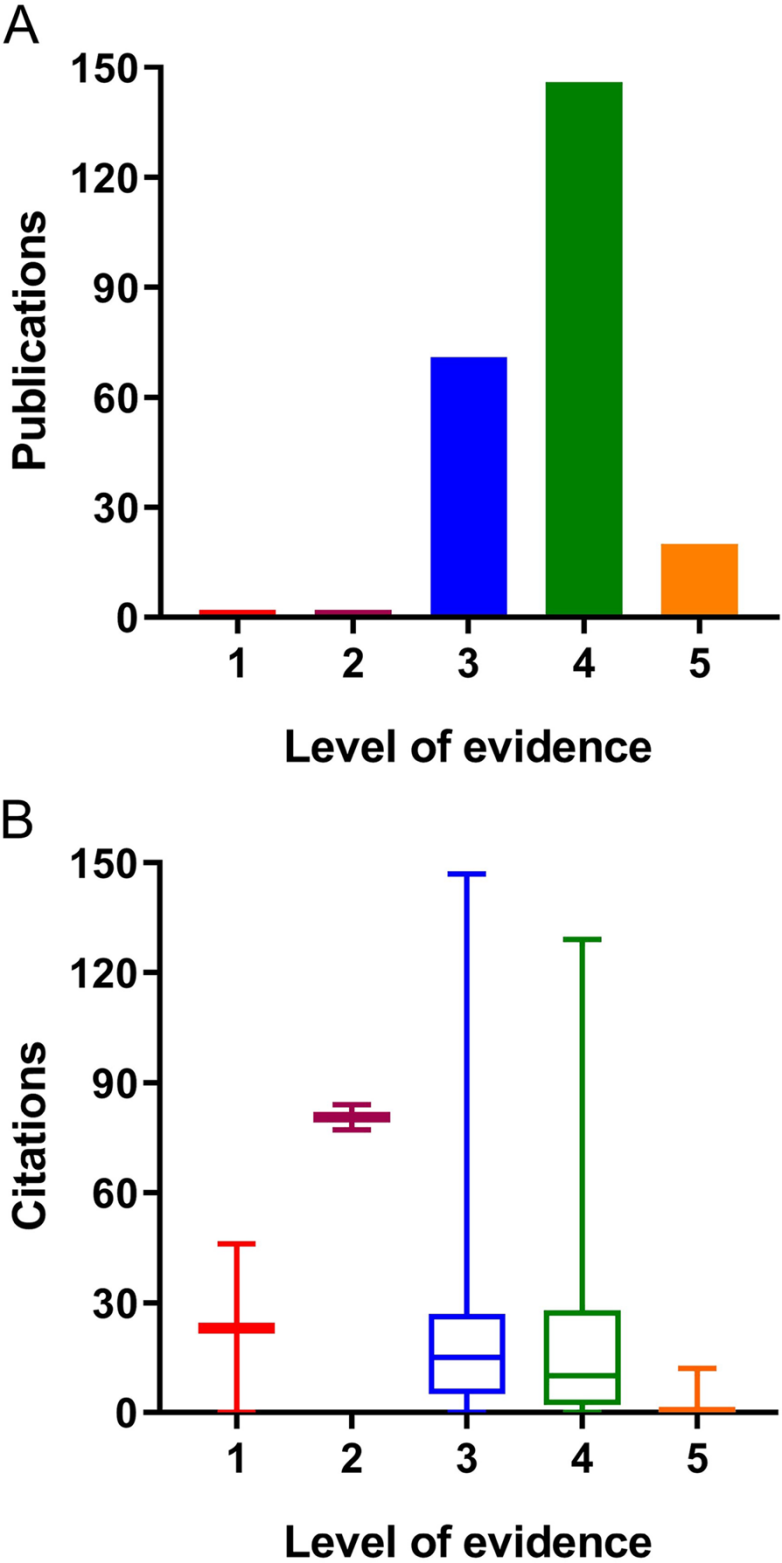
Number of publications and citations at different levels of evidence

In this study, the median citation counts of articles with level I and level II evidence were 23 (0, 46) and 80.5 (77, 84), respectively. For articles with level III, IV, and V evidence, the median citation counts were 15 (0, 147), 10 (0, 129), and 0 (0, 12), respectively (Fig. 4). By using Spearman’s test, a significant relationship was found between the number of citations and the level of evidence (*P* < 0.01). The correlation coefficient (*P* = 0.285) indicated that there was a positive and moderate association between the level of evidence and the number of citations, suggesting that higher levels of evidence are associated with a greater number of citations.

### Top publishing journals

Twelve journals were rated top 10 journals, in terms of number of articles and cumulative citations, (Tables 1 and 2). Of note, the *Knee* took the lead with the highest article count (34), which was closely followed by *Knee Surgery Sports Traumatology Arthroscopy* with 25 articles. In terms of citations, *Clinical Orthopaedics and Related Research* stood out with 951 citations, while* Knee* secured the second spot with 727 citations. Collectively, these 12 journals contributed to 67.6% of the analyzed articles, underscoring their significance in driving future research. The ten most cited articles from three journals: *Knee*, *Journal of Bone and Joint Surgery-British Volume*, and *Clinical Orthopaedics and Related Research* (Table 3). Notably, more than half of these articles were published in *Clinical Orthopaedics and Related Research*. All these articles were published between 2001 and 2007, indicating their lasting relevance.

**Table 1:** Top 10 published journals

**Journals**	**Publications**	**Citations**	**Average citations / Publications**
*Knee*	34	727	21.38
*Knee Surgery Sports Traumatology Arthroscopy*	25	449	17.96
*Clinical Orthopaedics and Related Research*	23	951	41.35
*Journal of Arthroplasty*	23	369	16.04
*Journal of Bone and Joint Surgery-British Volume*	12	537	44.75
*Bone & Joint Journal*	11	203	18.45
*Journal of Bone and Joint Surgery-American Volume*	9	235	26.11
*Journal of Knee Surgery*	7	26	3.71
*International Orthopaedics*	7	137	19.57
*Archives of Orthopaedic and Trauma Surgery*	6	102	17

**Table 2:** Top 10 journals by citations

**Journals**	**citations**	**publications**	**Average citations / publications**
*Clinical Orthopaedics and Related Research*	951	23	41.35
*Knee*	727	34	21.38
*Journal of Bone and Joint Surgery-British Volume*	537	12	44.75
*Knee Surgery Sports Traumatology Arthroscopy*	449	25	17.96
*Journal of Arthroplasty*	369	23	16.04
*Journal of Bone and Joint Surgery-American Volume*	235	9	26.11
*Bone & Joint Journal*	203	11	18.45
*Orthopedic Clinics of North America*	154	5	30.80
*International Orthopaedics*	137	7	19.57
*Scottish Medical Journal*	110	1	110.00

**Table 3:** Top 10 articles according to number of co-citations

**Title**	**Author**	**Journal**	**Year**	**Citations**
Is anterior knee pain a predisposing factor to patellofemoral osteoarthritis?	Utting, M.R.	*Knee*	2005	147
The Avon patellofemoral arthroplasty-Five-year survivorship and functional results	Ackroyd, C.E.	*Journal Of Bone and Joint Surgery-British Volume*	2007	129
Long-term results of patellofemoral arthroplasty-A report of 56 arthroplasties with 17 years of follow-up	Kooijman, H.J.	*Journal Of Bone and Joint Surgery-British Volume*	2003	128
The lubinus patellofemoral arthroplasty-A five- to ten-year prospective study	Ackroyd, C.E.	*Journal Of Bone and Joint Surgery-British Volume*	2001	126
Long-term results with the first patellotemoral prosthesis	Cartier, P.	*Clinical Orthopaedics and Related Research*	2005	96
Patellofemoral arthroplasty-Pros, cons, and design considerations	Lonner, J.H.	*Clinical Orthopaedics and Related Research*	2004	96
Patellofemoral arthroplasty-An update	Argenson, J.N.A.	*Clinical Orthopaedics and Related Research*	2005	95
Development and early results of a new patellofemoral arthroplasty	Ackroyd, C.E.	*Clinical Orthopaedics and Related Research*	2005	84
The appropriate use of patellofemoral arthroplasty-An analysis of reported indications, contraindications, and failures	Leadbetter, W.B.	*Clinical Orthopaedics and Related Research*	2005	77
Arthritis progression after patellofemoral joint replacement	Nicol, S.G.	*Knee*	2006	71

### Research interests

**Fig. 5:**
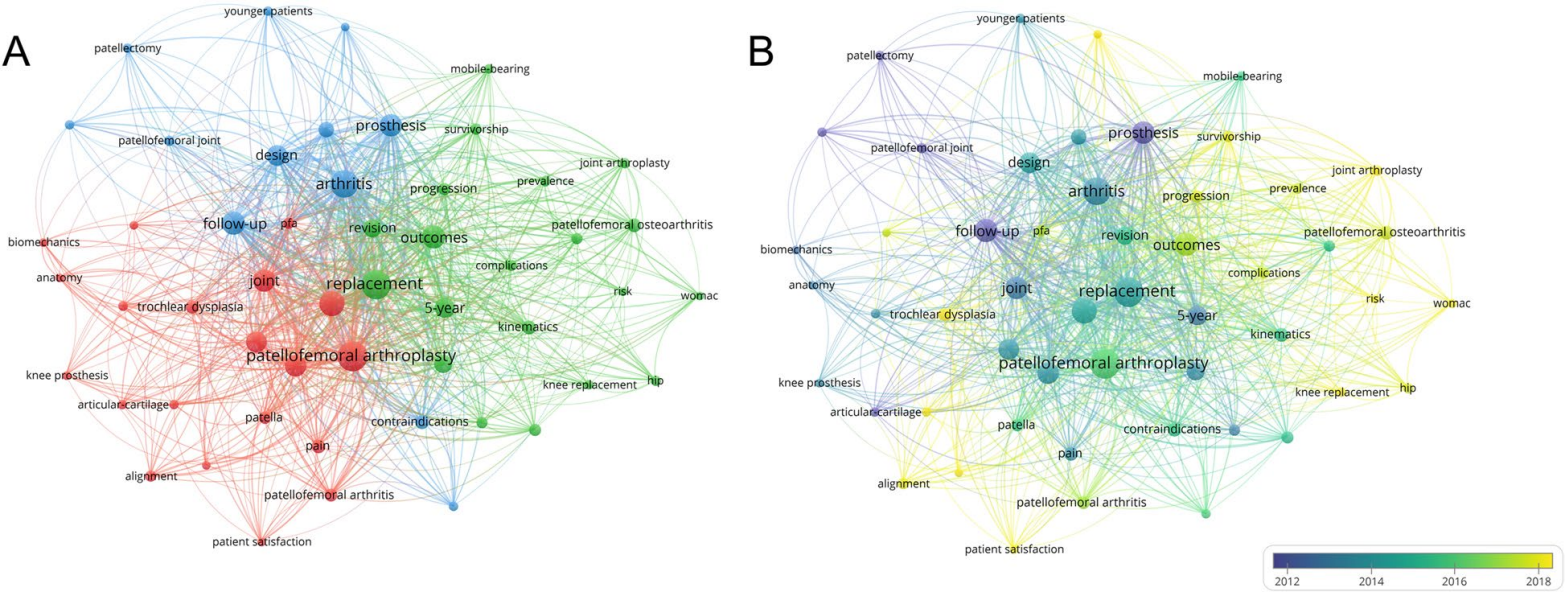
Keyword co-occurrence analysis. **A** Network co-occurrence clustering of keywords. **B** Overlay of keyword distribution by time of occurrence

**Fig. 6:**
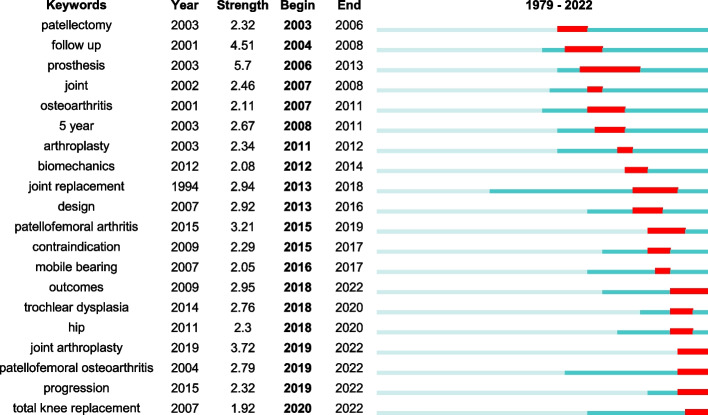
Top 20 Keywords with the Strongest Citation Bursts (The “Year” in the figure denotes the time of keyword appearance, “Begin” represents the start time of the cited outbreak of keywords, and “End” is the end time of the cited outbreak.)

By analyzing the frequency of occurrence, the study employed keyword analysis to reveal research interests and emerging trends in literature. The primary goal was to identify prevailing areas of scholarly concentration/interest in this field. Cluster analysis was utilized to group keywords that appeared five times or more (Fig. 5A). Node size in the figure indicates the frequency of occurrence, while lines represent connections between nodes, color-coded for clarity. Notably, the green cluster highlights clinical outcomes of PFA, covering prosthesis survival, revision procedures, and complications. Furthermore, the timeline graph in Fig. 5B shows the chronological distribution of the keywords. Darker shades indicate earlier appearances of these keywords over time. To look further into keyword clusters, a time-dependent evolution analysis was performed by using CiteSpace software to pinpoint the significant citation bursts during specific time spans (Fig. 6). These keywords shed light on shifts and emerging trends of PFA-related research, potentially signaling novel technological advancements and scientific breakthroughs.

### Core author groups and cooperative networks

**Fig. 7:**
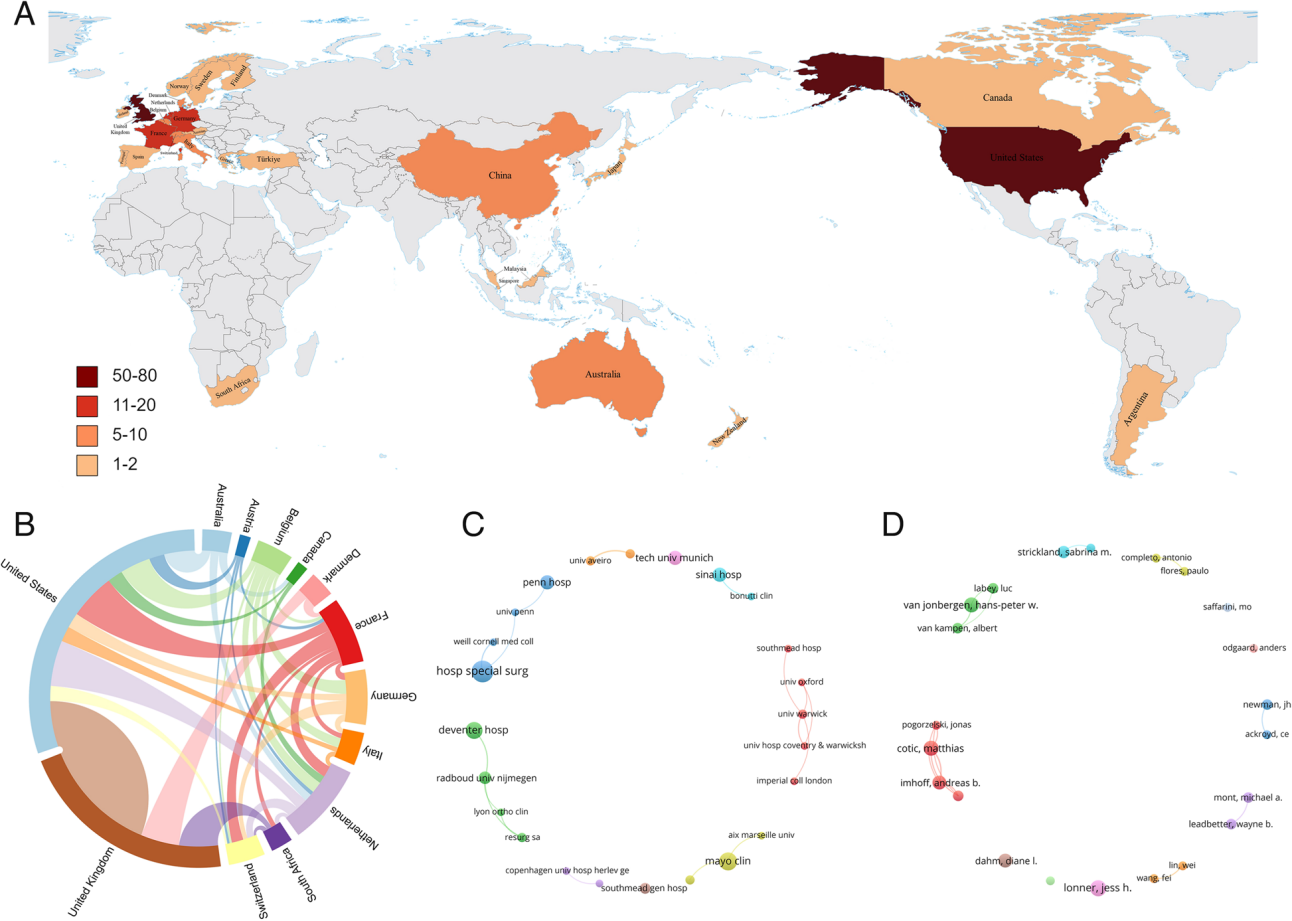
**A** World map distribution of the publishing countries. **B** Visualization of cooperative networks between countries (the colors represent different countries, the widths denote the number of publications, and the links show the international cooperation, with number of publications ≥ 3). **C** Visualization of collaborative networks between institutions (number of publications ≥ 3). **D** Collaborative network analysis among authors (number of publications ≥ 4)

The present study analyzed articles published by 27 countries or regions, as depicted in a global map (Fig. 7A). Notably, the United States emerged as the top contributor, having published 76 articles. The United Kingdom ranked second with 58 articles, followed by the Netherlands (15 articles), Germany (13 articles), and France (12 articles), among others. We investigated cooperation networks among countries having published three or more articles, and found that a total of 13 countries have established cooperation relations, as shown in Fig. 7B. The wider arcs indicate countries having published a greater number of articles and the middle line represents the extent of cooperation. Notably, the United States, the Netherlands, and France were more prominently engaged in international cooperation, mostly with other European countries, including Switzerland, Belgium, the United Kingdom, Germany, Italy, and others. Moreover, the United States and the United Kingdom exhibited frequent cooperation, whereas Denmark had comparatively minimal cooperation with other countries.

The analysis of the inter-institutional collaboration network revealed 24 institutions from 8 different countries: the United States, the United Kingdom, France, the Netherlands, Germany, Switzerland, Portugal, and Denmark (Fig. 7C). Notably, 8 institutions were from the United States and 6 from the United Kingdom, which together accounted for approximately 58.3% of the network. The Hospital for Special Surgery (13 articles) and the Mayo Clinic (10 articles) in the United States and the Deifentel Hospital (9 articles) in the Netherlands were significant contributors. These institutions tended to cooperate mainly in their own countries. In Fig. 7D, an alignment was observed among four prolific authors affiliated with the Technical University of Munich in Germany who have published a minimum of four articles. These authors are M. Cotic, M.J. Feucht, A.B. Imhoff, and J. Pogorzelski. Similarly, close collaboration was seen among individuals such as H.P.W. van Jonbergen from Deventer Hospital in the Netherlands, A. van Kampen from Nijmegen University, and L. Labey from the European Knee Research Centre in Belgium. The list of top productive authors included J.H. Lonner from Pennsylvania Hospital (9 articles), M. Cotic from the Technical University of Munich (8 articles), and H.P.W. van Jonbergen from Deventer Hospital in the Netherlands (8 articles). The most cited author was J.H. Lonner, followed by W.B. Leadbetter (Table 4). Impressively, up to 70% of authors held affiliations with institutions in the United States and the United Kingdom.

**Table 4:** Top 10 authors in terms of the number of co-citations

**Author**	**Institution**	**Country**	**Citations**
Lonner, J.H.	Pennsylvania Hospital	USA	268
Leadbetter, W.B.	Sinai Hospital	USA	182
Ackroyd, C.E.	Southmead Hospital	UK	177
Argenson, J.N.A.	Publique-Hopitaux de Marseille	France	119
Cartier, P.	Hartmann Knee Inst	France	113
Mont, M.A.	Sinai Hospital	USA	92
Tauro, B.	Southmead Hospital	UK	87
van Jonbergen, H.P.W.	Deventer Hospital	the Netherlands	85
Blazina, M.E.	Drexel University	USA	82
Davies, A.P.	Norfolk & Norwich University Hospital	UK	78

## Discussion

PFA has emerged as a promising treatment option for patients with isolated patellofemoral osteoarthritis [14, 27, 28]. However, the factors that influence the incidence of PFA in different institutions remain unclear. The higher revision rate of PFA due to progressive tibiofemoral arthritis may be a challenge. The appropriate indications and surgical techniques are crucial to the achievement of favorable outcomes in PFA surgeries [29]. In this study, we found that most of the highly-cited papers on PFA were corporately authored by a few centers. Frequently-cited papers came from research and development (R&D)-based medical institutions. Some of these were from institutions that are designers of PFA prostheses or clinical trial investigator institutions. For example, M.R. Utting, C.E. Ackroyd, and S.G. Nicol from Avon Orthopaedic Centre were the authors of highly cited articles. The Avon prosthesis is the most used PFJ prosthesis and has most research papers. Clinical outcomes reported by non-designers need to be clarified by further studies. Co-citation analysis showed that J.H. Lonner was one of the most frequently cited authors in the field.

This study revealed that there was a noticeable increase in article citations and postings around 2006. It was just about ten years after the development of the second-generation prosthesis. And good short- to middle-term follow-up results were reported. Two highly-cited articles [30, 31] about second-generation prosthesis reported on the result and served to stimulate the interest in the further study. The development of new implant designs, such as the Journey and Avon patellofemoral systems, has significantly advanced the evolution of PFA surgery [32, 33], leading to improved clinical outcomes and expanded application worldwide. Typically, the number of citations in an article was found to be related to its level of evidence. However, two Level I evidence articles were not cited as highly as the two Level II evidence articles. This discrepancy may be ascribed to the publication year of the articles, with the Level I articles published in 2018 [34] and 2022 [35], while the Level II articles appearing in 2005 [30, 36], indicating a possible bias in the results. It is crucial for researchers not to overlook shorter publications, which might not have gained enough attention due to limitations like time constraints or accessibility. We noted that frequently-cited articles tended to have greater influence in the field. These may include insightful articles published early on during the development of the research field, representative research that laid the foundation for subsequent research, or opened a new phase of development. Highly-cited articles can stimulate further research and development in the field, leading to significant advancements in the treatment of isolated patellofemoral arthritis. Our study identified the top ten most highly-cited articles published between 2001 and 2007.

The highly-cited articles mainly originated from the United States and the United Kingdom, with seven of the top ten articles also from these two countries. Our analysis with regard to countries and institutions indicated that the United States and the United Kingdom contributed to over 55% of the articles and about 58.3% of their institutions published more than three articles. This suggests that their researchers or institutions were more active in the field and are more likely to produce new results, which could impact the analysis of highly-cited papers. For instance, the first and second most highly-cited articles were authored by Utting, et al. [37] and Ackroyd, et al. [31], respectively, and both were from the United Kingdom. It is worth noting that these highly-cited articles were not the oldest ones. Therefore, our findings suggest that it is crucial to consider various factors when analyzing highly-cited papers, including publication trends, author affiliations, and article contents, to gain a comprehensive understanding of the research landscape in the field of patellofemoral arthroplasty.

Our investigation into the distribution of journal articles and the journals containing highly-cited articles revealed that these papers were typically found in well-regarded orthopedic journals such as *Knee*, *Knee Surgery Sports Traumatology Arthroscopy*, *Clinical Orthopaedics and Related Research*, and *Journal of Bone* and *Joint Surgery-British Volume*. These journals are highly popular with global researchers and have significant impact in the orthopedic community. Interestingly, we observed discrepancies between the top ten journals in the number of articles and citations. These differences may be attributed to variances in the quality of articles appearing in these journals, which may have different publication standards. For instance, some journals may use more rigorous review processes or have stricter criteria for article selection, leading to fewer but high-quality publications. Further research is warranted to identify the factors that may potentially dictate journal rankings and citation outcomes, such as editorial policies, submission trends, and emerging areas of interest in the field of orthopedics.

The visual analysis of keywords identified “follow-up”, “outcome”, and “revision” as the focal points of PFA research. Our study found that researchers often reported short- and mid-term outcomes for PFA, which were generally positive [38–40]. Upon closer examination, we discovered that studies on long-term outcomes of PFA were relatively infrequent and variable, with some studies reporting unsatisfactory results [13, 29, 41]. Notably, two studies, separately conducted by de Winter, et al. [42] and Kooijman, et al. [14], disclosed long-term follow-up results. De Winter, et al. [42] scored an 80% success rate over a mean period of 11 years, while Kooijman, et al. [14] attained an excellent success rate of 87% over a mean follow-up of 17 years. These studies focused on first-generation prostheses, thereby emphasizing the necessity for further attention to the long-term evaluation of second-generation prostheses.

Our research indicated that the second-generation patellofemoral joint arthroplasties may well be more popular with surgeons and scholars, thanks to their reported superior efficacy and durability when compared to first-generation PFA devices [27, 30, 43, 44]. To provide valuable clinical insights into the efficacy and durability of these devices, further studies are needed to examine their long-term outcomes and compare them to those of the first-generation PFA. Furthermore, research effort could be directed at the factors that influence PFA outcomes, such as prosthesis design, surgical techniques, and patient selection, to optimize treatment for patients, increase the success rate of PFA surgery, and improve overall prognosis.

For the assessment of PFA outcomes, early investigators tended to employ the Hungerford and Kenna knee scoring systems, prosthesis survival rate, as well as radiographs [14, 45–47]. However, recent research has focused more on patient-reported outcomes and satisfaction [28, 32, 44], reflecting a change in the philosophies about PFA for patellofemoral osteoarthritis in the academic community. Initially, the procedure was regarded as the ultimate joint replacement, and thus the focus was primarily on survival rates and imaging scores. In more recent years, there has been a mounting awareness of this technique as a step of a staged knee-preserving procedure prior to total knee arthroplasty (TKA), which aims at improving the subjective experience of younger patients and delaying the need for TKA. Unfortunately, revision of PFA remains relatively common and seems inevitable, despite continual improvements in surgical techniques, patient selection, and the use of better prostheses. The primary causes of revision are progressive tibiofemoral arthritis and pain [48–51] although misalignment and infection have also been reported [52, 53]. A recent systematic review of statistics showed that PFA patients receiving second-generation prostheses had 5- and 10-year survival rates of 94.28% and 88.89%, respectively. Total surgical complication rate was 14.5% over an average time of 5.5 years [27]. Most PFAs that require revision eventually convert to TKA, and the outcome after revision is similar to that of the initial TKA [54]. As such, PFA may be used as a transitional procedure for TKA in younger patients.

We acknowledge the limitations of our study. Although we searched multiple databases, some were excluded, which may have affected the inclusion of articles. Different databases utilize varying methods for calculating citations, which could have impacted the results. Additionally, recent high-impact articles may have been overlooked due to low citation rates. To address this issue, we recommend selecting comprehensive databases that encompass a wide range of articles. This will mitigate the problem. Furthermore, staying up-to-date with the latest research is essential to our investigation.

## Conclusion

In the field of PFA research, there has been an increasing trend in publications, with a majority coming from a limited number of centers and a lack of high-level evidence. Most studies have focused on clinical outcomes of PFA, while in recent years, the trend has been shifted towards refurbishment of PFA and patient satisfaction.

### Supplementary Information

**Additional file 1:** **Table S1.** Detailed bibliographies searched from WOS Core Collection in the field of patellofemoral arthroplasty. ** Table S2.** Detailed bibliographies searched from Pubmed in the field of patellofemoral arthroplasty. ** Table S3.** Detailed bibliographies searched from BIOSIS Citation Index in the field of patellofemoral arthroplasty. ** Table S4.** Detailed bibliographies searched from Springer in the field of patellofemoral arthroplasty. **Table S5.** Detailed bibliographies searched from Medline in the field of patellofemoral arthroplasty. **Table S6.** List of bibliographies that meet the criteria and are included in bibliometric analysis.

## Data Availability

Not applicable.
