# Supplementary material for: The landscape of patellofemoral arthroplasty research: a bibliometric analysis
Source: Arthroplasty. 2023 Dec 3;5:65. doi: 10.1186/s42836-023-00215-1 (PMC10693710; doi:10.1186/s42836-023-00215-1)
Supplement: Supplementary file 1 — Additional file 1: Table S1. Detailed bibliographies searched from WOS Core Collection in the field of patellofemoral arthroplasty. Table S2. Detailed bibliographies searched from Pubmed in the field of patellofemoral arthroplasty. Table S3. Detailed bibliographies searched from BIOSIS Citation Index in the field of patellofemoral arthroplasty. Table S4. Detailed bibliographies searched from Springer in the field of patellofemoral arthroplasty. Table S5. Detailed bibliographies searched from Medline in the field of patellofemoral arthroplasty. Table S6. List of bibliographies that meet the criteria and are included in bibliometric analysis. [file 42836_2023_215_MOESM1_ESM.docx]

**Supporting Information**

*Supplementary Tables*

*in*

**Assessing the Landscape of Patellofemoral Arthroplasty Research:**

**A Bibliometric Analysis**

*Yang Yao^1,2^, Chen Yuan^1,2^, Wang Yingjie^1,2^, Wang Junjie^1,2^, Lu Baoliang^1,3^, Zhu Wanbo^4^, Yang Ning^1^, Zhu Junchen^2*^, Zhu Chen^1*^ and Zhang Xianzuo^1*^*

^1^ Department of Orthopedics, The First Affiliated Hospital of USTC, Division of Life Sciences and Medicine, University of Science and Technology of China, Hefei, China

^2^ Department of Orthopedics, The Second Affiliated Hospital of Anhui University of Chinese Medicine, Hefei, Anhui Province, China

^3^ Graduate School of Bengbu Medical College

^4^ Department of Orthopedics, Shanghai Jiao Tong University Affiliated Sixth People’s Hospital, Shanghai Jiao Tong University

| **Table S1 Detailed bibliographies searched from WOS Core Collection in the field of patellofemoral arthroplasty** | | | | | | | |
| --- | --- | --- | --- | --- | --- | --- | --- |
| **WOS ID** | **Article Title** | **Authors** | **Journal** | **Publication Year** | | **DOI** | **Language** |
| WOS:000233481000011 | Patellar clunk syndrome in patellofemoral arthroplasty - a case report | Sringari, T; Maheswaran, SS | KNEE | | 2005 | 10.1016/j.knee.2004.11.008 | English |
| WOS:000433204500007 | Advances in Patellofemoral Arthroplasty | Strickland, SM; Bird, ML; Christ, AB | CURRENT REVIEWS IN MUSCULOSKELETAL MEDICINE | | 2018 | 10.1007/s12178-018-9477-0 | English |
| WOS:000346584500001 | Outcomes of Total Knee Replacement after Patellofemoral Arthroplasty | Hutt, J; Dodd, M; Bourke, H; Bell, J | JOURNAL OF KNEE SURGERY | | 2013 | 10.1055/s-0032-1329233 | English |
| WOS:000343344900010 | Patellofemoral joint replacement, an evolving concept | Borus, T; Brilhault, J; Confalonieri, N; Johnson, D; Thienpont, E | KNEE | | 2014 | 10.1016/S0968-0160(14)50010-5 | English |
| WOS:000343344900009 | Partial knee arthroplasty: patellofemoral arthroplasty and combined unicompartmental and patellofemoral arthroplasty implants - general considerations and indications, technique and clinical experience | Benazzo, F; Rossi, SMP; Ghiara, M | KNEE | | 2014 | 10.1016/S0968-0160(14)50009-9 | English |
| WOS:000300057300025 | Patellar Polyethylene Spinout After Low-contact Stress, High-congruity, Mobile-bearing Patellofemoral Arthroplasty | Amanatullah, DF; Jamali, AA | ORTHOPEDICS | | 2012 | 10.3928/01477447-20120123-27 | English |
| WOS:000728362400008 | When nothing else works: patellofemoral joint arthroplasty | Benignus, C; Meier, M; Best, R; Beckmann, J | SPORTVERLETZUNG-SPORTSCHADEN | | 2021 | 10.1055/a-1523-9937 | German |
| WOS:000256749100007 | Unique combination of patellofemoral joint arthroplasty with Osteochondral Autograft Transfer System (OATS) - A case series of six knees in five patients | Unnithan, A; Jimulia, T; Mohammed, R; Learmonth, DJA | KNEE | | 2008 | 10.1016/j.knee.2008.01.007 | English |
| WOS:000287900400014 | Medial patellofemoral ligament reconstruction for subluxating patellofemoral arthroplasty | Carmont, MR; Crane, T; Thompson, P; Spalding, T | KNEE | | 2011 | 10.1016/j.knee.2010.02.011 | English |
| WOS:000281788600006 | Patellofemoral joint replacement | Van Jonergen, HPW; Van Lingen, CP | MINERVA ORTOPEDICA E TRAUMATOLOGICA | | 2010 |  | English |
| WOS:000437071700019 | Femoral component rotation in patellofemoral joint replacement | van Jonbergen, HPW; Westerbeek, RE | KNEE | | 2018 | 10.1016/j.knee.2018.02.007 | English |
| WOS:000601532900001 | Patellofemoral inlay implants-an innovation in patellofemoral joint arthroplasty? | Degenhardt, H; Imhoff, AB; Feucht, MJ; Mhba, JP | ORTHOPADE | | 2021 | 10.1007/s00132-020-04059-4 | German |
| WOS:000241769800003 | Revision of a failed patellofemoral arthroplasty to a total knee arthroplasty | Lonner, JH; Jasko, JG; Booth, RE | JOURNAL OF BONE AND JOINT SURGERY-AMERICAN VOLUME | | 2006 | 10.2106/JBJS.F.00282 | English |
| WOS:000754051300013 | Obesity Does Not Affect Patient-Reported Outcomes following Patellofemoral Arthroplasty | Tishelman, JC; Pyne, A; Kahlenberg, CA; Gruskay, JA; Strickland, SM | JOURNAL OF KNEE SURGERY | | 2022 | 10.1055/s-0040-1713862 | English |
| WOS:000454754000038 | Patellofemoral joint replacement - Mean five year follow-up | Ajnin, S; Buchanan, D; Arbuthnot, J; Fernandes, R | KNEE | | 2018 | 10.1016/j.knee.2018.08.014 | English |
| WOS:000274927800013 | Strain shielding in distal femur after patellofemoral arthroplasty under different activity conditions | Meireles, S; Completo, A; Simoes, JA; Flores, P | JOURNAL OF BIOMECHANICS | | 2010 | 10.1016/j.jbiomech.2009.09.048 | English |
| WOS:000457234600003 | The evolution and role of patellofemoral joint arthroplasty THE ROAD LESS TRAVELLED, BUT NOT FORGOTTEN | Roussot, MA; Haddad, FS | BONE & JOINT RESEARCH | | 2018 | 10.1302/2046-3758.712.BJR-2018-0303 | English |
| WOS:000258920700007 | Medium-term results of patellofemoral joint arthroplasty | Mohammed, R; Jimulia, T; Durve, K; Bansal, M; Green, M; Learmonth, D | ACTA ORTHOPAEDICA BELGICA | | 2008 |  | English |
| WOS:000313426800024 | COMPARISON OF COMMERCIAL PATELLOFEMORAL ARTHROPLASTY SYSTEMS ON THE BASIS OF PATELLA KINEMATICS, PERI-PATELLAR SOFT TISSUE TENSION AND PROSTHESIS DESIGN | Muller, JH; Erasmus, PJ; Scheffer, C | JOURNAL OF MECHANICS IN MEDICINE AND BIOLOGY | | 2012 | 10.1142/S0219519412500868 | English |
| WOS:000447685400073 | Patellar tracking before and after a Patellofemoral joint replacement (Depuy Sigma) - a cadaveric study using computer navigation | Sussmann, P; Heinert, G; Wolfgang, K; Preiss, S | SWISS MEDICAL WEEKLY | | 2011 |  | English |
| WOS:000309878500001 | Differences in the stress distribution in the distal femur between patellofemoral joint replacement and total knee replacement: a finite element study | van Jonbergen, HPW; Innocenti, B; Gervasi, GL; Labey, L; Verdonschot, N | JOURNAL OF ORTHOPAEDIC SURGERY AND RESEARCH | | 2012 | 10.1186/1749-799X-7-28 | English |
| WOS:000529360500003 | Minimally Invasive Robotic-Assisted Patellofemoral Arthroplasty | Hassebrock, JD; Makovicka, JL; Wong, M; Patel, KA; Scott, KL; Deckey, DG; Chhabra, A | ARTHROSCOPY TECHNIQUES | | 2020 | 10.1016/j.eats.2019.11.013 | English |
| WOS:000496759400015 | The Clinical Outcome of Patellofemoral Arthroplasty vs Total Knee Arthroplasty in Patients Younger Than 55 Years | Kamikovski, I; Dobransky, J; Dervin, GE | JOURNAL OF ARTHROPLASTY | | 2019 | 10.1016/j.arth.2019.07.016 | English |
| WOS:000612962500011 | Patellofemoral Replacement With Tibial Tubercle Osteotomy | Arvesen, JE; Wyland, DJ | ARTHROSCOPY TECHNIQUES | | 2021 | 10.1016/j.eats.2020.09.009 | English |
| WOS:000441912100033 | No bias for developer publications and no difference between first-generation trochlear-resurfacing versus trochlear-cutting implants in 15,306 cases of patellofemoral joint arthroplasty | Reihs, B; Reihs, F; Labek, G; Hochegger, M; Leithner, A; Bohler, N; Sadoghi, P | KNEE SURGERY SPORTS TRAUMATOLOGY ARTHROSCOPY | | 2018 | 10.1007/s00167-017-4692-6 | English |
| WOS:000283333800010 | Long-Term Outcomes of Patellofemoral Arthroplasty | van Jonbergen, HPW; Werkman, DM; Barnaart, LF; van Kampen, A | JOURNAL OF ARTHROPLASTY | | 2010 | 10.1016/j.arth.2009.08.023 | English |
| WOS:000922293700020 | Functional Outcomes, Survival Rate, and Complications of Patellofemoral Arthroplasty: Mid-Term Results From Independent Center | Jagadeesh, N; Sales-Fernandez, R; Pammi, S; Kariya, A | CUREUS JOURNAL OF MEDICAL SCIENCE | | 2022 | 10.7759/cureus.31945 | English |
| WOS:000267608600020 | Dislocation of the mobile bearing component of a patellofemoral arthroplasty: A report of two cases | Witjes, S; Van den Broek, C; Koeter, S; Van Loon, C | ACTA ORTHOPAEDICA BELGICA | | 2009 |  | English |
| WOS:000273700400013 | Scientific evidence for the use of modern patellofemoral arthroplasty | Gupta, RR; Zywiel, MG; Leadbetter, WB; Bonutti, P; Mont, MA | EXPERT REVIEW OF MEDICAL DEVICES | | 2010 | 10.1586/ERD.09.53 | English |
| WOS:000184552100029 | Patellofemoral arthroplasty | Smith, A; Lucas, D | JOURNAL OF BONE AND JOINT SURGERY-AMERICAN VOLUME | | 2003 | 10.2106/00004623-200308000-00029 | English |
| WOS:000343136900006 | Patellofemoral arthroplasty. Indication, technique and results | Cotic, M; Imhoff, AB | ORTHOPADE | | 2014 | 10.1007/s00132-014-3006-7 | German |
| WOS:000184552100030 | Patellofemoral arthroplasty - Reply | Mont, MA; Haas, S | JOURNAL OF BONE AND JOINT SURGERY-AMERICAN VOLUME | | 2003 | 10.2106/00004623-200308000-00030 | English |
| WOS:000224736900004 | Early results with a total patellofemoral joint replacement arthroplasty prosthesis | Merchant, AC | JOURNAL OF ARTHROPLASTY | | 2004 | 10.1016/j.arth.2004.03.011 | English |
| WOS:000301208500005 | Patellofemoral Arthroplasty 7-year Mean Follow-Up | Mont, MA; Johnson, AJ; Naziri, Q; Kolisek, FR; Leadbetter, WB | JOURNAL OF ARTHROPLASTY | | 2012 | 10.1016/j.arth.2011.07.010 | English |
| WOS:000205636600009 | Ipsilateral Patellofemoral Arthroplasty and Autogenous Osteochondral Femoral Condylar Transplantation | Lonner, JH; Mehta, S; Booth, RE | JOURNAL OF ARTHROPLASTY | | 2007 | 10.1016/j.arth.2005.08.012 | English |
| WOS:000764732500001 | Patellofemoral arthroplasty: expert opinion | Hoogervorst, P; Arendt, EA | JOURNAL OF EXPERIMENTAL ORTHOPAEDICS | | 2022 | 10.1186/s40634-022-00457-z | English |
| WOS:000258226000008 | Patellofemoral arthroplasty: The impact of design on outcomes | Lonner, JH | ORTHOPEDIC CLINICS OF NORTH AMERICA | | 2008 | 10.1016/j.ocl.2008.02.002 | English |
| WOS:000501844300011 | Isolated Patellofemoral Joint Arthroplasty: Can Preoperative Bone Scans Predict Survivorship? | Baker, JF; Caborn, DN; Schlierf, TJ; Fain, TB; Smith, LS; Malkani, AL | JOURNAL OF ARTHROPLASTY | | 2020 | 10.1016/j.arth.2019.08.021 | English |
| WOS:000637664900002 | Midterm results of modern patellofemoral arthroplasty versus total knee arthroplasty for isolated patellofemoral arthritis: systematic review and meta-analysis of comparative studies | Elbardesy, H; McLeod, A; Gul, R; Harty, J | ARCHIVES OF ORTHOPAEDIC AND TRAUMA SURGERY | | 2022 | 10.1007/s00402-021-03882-4 | English |
| WOS:000230134300003 | Patellofemoral arthroplasty - The third compartment | Lotke, PA; Lonner, JH; Nelson, CL | JOURNAL OF ARTHROPLASTY | | 2005 | 10.1016/j.arth.2005.03.011 | English |
| WOS:000545922000001 | Patellofemoral joint arthroplasty What is the current situation? | Beckmann, J; Meier, M; Halder, A; Best, R; Thienpont, E; Beier, A | ARTHROSKOPIE | | 2020 | 10.1007/s00142-020-00375-1 | German |
| WOS:000853300500001 | A radiological index that influences the outcome following patellofemoral joint arthroplasty: the anterior trochlea offset ratio | Aweid, O; Ahearn, N; Metcalfe, AJ; Eldridge, J; Porteous, A; Murray, JR | KNEE SURGERY SPORTS TRAUMATOLOGY ARTHROSCOPY | | 2023 | 10.1007/s00167-022-07085-1 | English |
| WOS:000326199600010 | Clinical Results of Patellofemoral Arthroplasty | Morris, MJ; Lombardi, AV; Berend, KR; Hurst, JM; Adams, JB | JOURNAL OF ARTHROPLASTY | | 2013 | 10.1016/j.arth.2013.05.012 | English |
| WOS:000577135900001 | Patellofemoral Arthroplasty (vol 9, e15, 2019) | Odgaard | JBJS ESSENTIAL SURGICAL TECHNIQUES | | 2020 | 10.2106/JBJS.ST.ER.18.00094 | English |
| WOS:000331160300024 | Mid-term results of the FPV patellofemoral joint replacement | Al-Hadithy, N; Patel, R; Navadgi, B; Deo, S; Hollinghurst, D; Satish, V | KNEE | | 2014 | 10.1016/j.knee.2013.08.010 | English |
| WOS:000258226000009 | Patellofemoral arthroplasty with a customized trochlear prosthesis | Sisto, DJ; Sarin, VK | ORTHOPEDIC CLINICS OF NORTH AMERICA | | 2008 | 10.1016/j.ocl.2008.03.002 | English |
| WOS:000447709900196 | Early advantage of patellofemoral arthroplasty is not age dependent | Lehoczky, G; Mumme, M; Jakob, M; Pagenstert, G | SWISS MEDICAL WEEKLY | | 2017 |  | English |
| WOS:000174593400041 | The Lubinus patellofemoral arthroplasty (vol 83B, pg 696, 2001) | Tauro, B; Ackroyd, CE; Newman, JH; Shah, NA | JOURNAL OF BONE AND JOINT SURGERY-BRITISH VOLUME | | 2002 |  | English |
| WOS:000230292900015 | The appropriate use of patellofemoral arthroplasty - An analysis of reported indications, contraindications, and failures | Leadbetter, WB; Ragland, PS; Mont, MA | CLINICAL ORTHOPAEDICS AND RELATED RESEARCH | | 2005 | 10.1097/01.blo.0000172304.12533.41 | English |
| WOS:000272174300021 | Patellofemoral arthroplasty: a multi-centre study with minimum 2-year follow-up | Leadbetter, WB; Kolisek, FR; Levitt, RL; Brooker, AF; Zietz, P; Marker, DR; Bonutti, PM; Mont, MA | INTERNATIONAL ORTHOPAEDICS | | 2009 | 10.1007/s00264-008-0692-y | English |
| WOS:000314440600003 | Trochlear Inclination Angles in Normal and Dysplastic Knees | Kamath, AF; Slattery, TR; Levack, AE; Wu, CH; Kneeland, JB; Lonner, JH | JOURNAL OF ARTHROPLASTY | | 2013 | 10.1016/j.arth.2012.04.017 | English |
| WOS:000174593400038 | The Lubinus patellofemoral arthroplasty: a five- to ten-year prospective study | Roach, R | JOURNAL OF BONE AND JOINT SURGERY-BRITISH VOLUME | | 2002 | 10.1302/0301-620X.84B2.0840307 | English |
| WOS:A1988R039800008 | PATELLOFEMORAL ARTHROPLASTY A 3-YEAR TO 9-YEAR FOLLOW-UP-STUDY | ARCIERO, RA; TOOMEY, HE | CLINICAL ORTHOPAEDICS AND RELATED RESEARCH | | 1988 |  | English |
| WOS:000322847200003 | The Clinical Outcome of Patellofemoral Arthroplasty | Lonner, JH; Bloomfield, MR | ORTHOPEDIC CLINICS OF NORTH AMERICA | | 2013 | 10.1016/j.ocl.2013.03.002 | English |
| WOS:000174593400036 | The Lubinus patellofemoral arthroplasty: a five- to ten-year prospective study | Coleridge, S | JOURNAL OF BONE AND JOINT SURGERY-BRITISH VOLUME | | 2002 | 10.1302/0301-620X.84B2.0840306a | English |
| WOS:000174593400039 | The Lubinus patellofemoral arthroplasty: a five- to ten-year prospective study - Reply | Ackroyd, C; Newman, JH | JOURNAL OF BONE AND JOINT SURGERY-BRITISH VOLUME | | 2002 | 10.1302/0301-620X.84B2.0840307a | English |
| WOS:000310223100007 | Complications after patello-femoral versus total knee replacement in the treatment of isolated patello-femoral osteoarthritis. A meta-analysis | Dy, CJ; Franco, N; Ma, Y; Mazumdar, M; McCarthy, MM; Della Valle, AG | KNEE SURGERY SPORTS TRAUMATOLOGY ARTHROSCOPY | | 2012 | 10.1007/s00167-011-1677-8 | English |
| WOS:000297981000001 | The Warwick patellofemoral arthroplasty trial: a randomised clinical trial of total knee arthroplasty versus patellofemoral arthroplasty in patients with severe arthritis of the patellofemoral joint | Odumenya, M; McGuinness, K; Achten, J; Parsons, N; Spalding, T; Costa, M | BMC MUSCULOSKELETAL DISORDERS | | 2011 | 10.1186/1471-2474-12-265 | English |
| WOS:000281621800011 | Unusual mechanical complications of unicompartmental low contact stress mobile bearing patellofemoral arthroplasty: A cause for concern? | Arumilli, BRB; Ng, ABY; Ellis, DJ; Hirst, P | KNEE | | 2010 | 10.1016/j.knee.2009.10.006 | English |
| WOS:000262221600027 | Patellofemoral Arthroplasty - Results of a Nation-Wide Survey in Germany and Review of the Literature | Becher, C; Renke, A; Heyse, TJ; Schofer, M; Tibesku, CO; Fuchs-Winkelmann, S | ZEITSCHRIFT FUR ORTHOPADIE UND UNFALLCHIRURGIE | | 2008 | 10.1055/s-2008-1039000 | German |
| WOS:000259855700006 | Revision patellofemoral arthroplasty - Three- to seven-year follow-up | Hendrix, MRG; Ackroyd, CE; Lonner, JH | JOURNAL OF ARTHROPLASTY | | 2008 | 10.1016/j.arth.2007.10.019 | English |
| WOS:A1995TJ25400025 | IS THERE A PLACE FOR PATELLOFEMORAL ARTHROPLASTY | ARGENSON, JNA; GUILLAUME, JM; AUBANIAC, JM | CLINICAL ORTHOPAEDICS AND RELATED RESEARCH | | 1995 |  | English |
| WOS:000174593400037 | The Lubinus patellofemoral arthroplasty: a five- to ten-year prospective study - Reply | Ackroyd, CE; Newman, JH | JOURNAL OF BONE AND JOINT SURGERY-BRITISH VOLUME | | 2002 | 10.1302/0301-620X.84B2.0840306b | English |
| WOS:000542165100052 | Aiming for anatomical femoral axis on the coronal plane leads to good-to-excellent short-term outcomes in isolated patellofemoral arthroplasty | Vasta, S; Rosi, M; Tecame, A; Papalia, R; Adravanti, P | KNEE | | 2020 | 10.1016/j.knee.2020.02.016 | English |
| WOS:000242775500014 | Indications, contraindications and pitfalls of patellofemoral arthroplasty | Leadbetter, WB; Seyler, TM; Ragland, PS; Mont, MA | JOURNAL OF BONE AND JOINT SURGERY-AMERICAN VOLUME | | 2006 | 10.2106/JBJS.F.00856 | English |
| WOS:000251502200041 | Off-center femur-patella arthritis: Femur-patella prothesis or traditional surgery | Argenson, JN | REVUE DE CHIRURGIE ORTHOPEDIQUE ET REPARATRICE DE L APPAREIL MOTEUR | | 2007 | 10.1016/S0035-1040(07)92748-8 | French |
| WOS:000237216100017 | Computer-assisted patellofemoral arthroplasty - A mechanism for optimizing rotation | Cossey, AJ; Spriggins, AJ | JOURNAL OF ARTHROPLASTY | | 2006 | 10.1016/j.arth.2005.08.010 | English |
| WOS:000842068600096 | A prospective matched-pair analysis of two different inlay trochlear designs for patellofemoral arthroplasty | Martinho, T; Cotic, M; Pogorzelski, J; Imhoff, A | SWISS MEDICAL WEEKLY | | 2022 |  | English |
| WOS:000284387200013 | A preliminary report of patellofemoral arthroplasty in isolated patellofemoral arthritis | Gao, XA; Xu, ZJ; He, RX; Yan, SG; Wu, LD | CHINESE MEDICAL JOURNAL | | 2010 | 10.3760/cma.j.issn.0366-6999.2010.21.013 | English |
| WOS:000371106400028 | Outcome of patellofemoral arthroplasty, determinants for success | Willekens, P; Victor, J; Verbruggen, D; Vande Kerckhove, M; Van der Straeten, C | ACTA ORTHOPAEDICA BELGICA | | 2015 |  | English |
| WOS:000445304400005 | Patellofemoral arthroplasty in a young woman with anterior knee disabling pain and developmental hip dysplasia | Spina, M; Bagnis, F | MINERVA ORTOPEDICA E TRAUMATOLOGICA | | 2018 | 10.23736/S0394-3410.18.03882-1 | English |
| WOS:000654173300001 | Treatment of Unicompartmental Cartilage Defects of the Knee with Unicompartmental Knee Arthroplasty, Patellofemoral Partial Knee Arthroplasty or Focal Resurfacing | Springer, B; Boettner, F | LIFE-BASEL | | 2021 | 10.3390/life11050394 | English |
| WOS:000309193200011 | Patellofemoral Arthroplasty: The Other Unicompartmental Knee Replacement | Walker, T; Perkinson, B; Mihalko, WM | JOURNAL OF BONE AND JOINT SURGERY-AMERICAN VOLUME | | 2012 | 10.2106/JBJS.L.00539 | English |
| WOS:000282916700005 | UKA in combination with PFR at average 12-year follow-up | Heyse, TJ; Khefacha, A; Cartier, P | ARCHIVES OF ORTHOPAEDIC AND TRAUMA SURGERY | | 2010 | 10.1007/s00402-009-0997-3 | English |
| WOS:000230292900011 | Outcome instruments for pateflofemoral arthroplasty | Paxton, EW; Fithian, DC | CLINICAL ORTHOPAEDICS AND RELATED RESEARCH | | 2005 | 10.1097/01.blo.0000171544.38095.77 | English |
| WOS:000233231000011 | Patellofemoral arthroplasty - An update | Argenson, JNA; Flecher, X; Parratte, S; Aubaniac, JM | CLINICAL ORTHOPAEDICS AND RELATED RESEARCH | | 2005 | 10.1097/01.blo.0000187061.27573.70 | English |
| WOS:000264347300013 | Conversion of patellofemoral arthroplasty to total knee arthroplasty | van Jonbergen, HPW; Werkman, DM; van Kampen, A | ACTA ORTHOPAEDICA | | 2009 | 10.1080/17453670902805031 | English |
| WOS:000711677700042 | Letter to the Editor: Short-term Revision Risk of Patellofemoral Arthroplasty is High: An Analysis From Eight Large Arthroplasty Registries | van Jonbergen, HPW; Kleinlugtenbelt, YV | CLINICAL ORTHOPAEDICS AND RELATED RESEARCH | | 2021 | 10.1097/CORR.0000000000001717 | English |
| WOS:A1979JF71200017 | PATELLOFEMORAL REPLACEMENT | BLAZINA, ME; FOX, JM; DELPIZZO, W; BROUKHIM, B; IVEY, FM | CLINICAL ORTHOPAEDICS AND RELATED RESEARCH | | 1979 | 10.1097/00003086-197910000-00017 | English |
| WOS:000528331100030 | Total knee arthroplasty reduces knee extension torque in-vitro and patellofemoral arthroplasty does not | Joseph, MN; Carmont, MR; Tailor, H; Stephen, JM; Amis, AA | JOURNAL OF BIOMECHANICS | | 2020 | 10.1016/j.jbiomech.2020.109739 | English |
| WOS:000277289700001 | Distal femoral bone mineral density decreases following patellofemoral arthroplasty: 1-year follow-up study of 14 patients | van Jonbergen, HPW; Koster, K; Labey, L; Innocenti, B; van Kampen, A | BMC MUSCULOSKELETAL DISORDERS | | 2010 | 10.1186/1471-2474-11-74 | English |
| WOS:000272343600007 | The Avon patellofemoral joint replacement INDEPENDENT ASSESSMENT OF EARLY FUNCTIONAL OUTCOMES | Starks, I; Roberts, S; White, SH | JOURNAL OF BONE AND JOINT SURGERY-BRITISH VOLUME | | 2009 | 10.1302/0301-620X.91B12.23018 | English |
| WOS:000572613000001 | Pre-operative patella alta does not affect midterm clinical outcomes and survivorship of patellofemoral arthroplasty | Bernard, CD; Pareek, A; Sabbag, CM; Parkes, CW; Krych, AJ; Cummings, NM; Dahm, DL | KNEE SURGERY SPORTS TRAUMATOLOGY ARTHROSCOPY | | 2021 | 10.1007/s00167-020-06205-z | English |
| WOS:000356402000016 | Update on Patellofemoral Arthroplasty | Crowe, MM; Dahm, DL | OPERATIVE TECHNIQUES IN SPORTS MEDICINE | | 2015 | 10.1053/j.otsm.2015.05.001 | English |
| WOS:000382902100003 | Patella component loosening - A case report | Bloemheuvel, EM; van Rooij, WMJ; van den Besselaar, M | ACTA ORTHOPAEDICA BELGICA | | 2016 |  | English |
| WOS:000248642000006 | Patellofemoral arthroplasty | Lonner, JH | JOURNAL OF THE AMERICAN ACADEMY OF ORTHOPAEDIC SURGEONS | | 2007 | 10.5435/00124635-200708000-00006 | English |
| WOS:000704620900027 | Combined patellofemoral arthroplasty and medial patellofemoral ligament reconstruction for chronic patellar instability with trochlear dysplasia: a report of two cases | Yamagami, R; Inui, H; Taketomi, S; Tanaka, S | MODERN RHEUMATOLOGY CASE REPORTS | | 2020 | 10.1080/24725625.2019.1638048 | English |
| WOS:000530078000045 | The midterm results of a cohort study of patellofemoral arthroplasty from a non-designer center using an asymmetric trochlear prosthesis (vol 26, pg 1348, 2019) | Rammohan, R; Gupta, S; Lee, PYF; Chandratreya, A | KNEE | | 2020 | 10.1016/j.knee.2020.02.009 | English |
| WOS:000660880400001 | Patient-related outcomes of patellofemoral arthroplasty: experience of a single center | Abeysekera, WYM; Schenk, W | ARTHROPLASTY | | 2021 | 10.1186/s42836-021-00074-8 | English |
| WOS:000711677700043 | Reply to the Letter to the Editor: Short-term Revision Risk of Patellofemoral Arthroplasty is High: An Analysis From Eight Large Arthroplasty Registries | Lewis, PL | CLINICAL ORTHOPAEDICS AND RELATED RESEARCH | | 2021 | 10.1097/CORR.0000000000001718 | English |
| WOS:000259259400028 | Patellofemoral replacement: The third compartment | Minas, T | ORTHOPEDICS | | 2008 | 10.3928/01477447-20080901-24 | English |
| WOS:000481882700012 | Patellar tendon shortening following patellofemoral joint replacement | van Engen, LAH; Landman, EBM; Kleinlugtenbelt, YV; van Jonbergen, HPW | INTERNATIONAL ORTHOPAEDICS | | 2019 | 10.1007/s00264-018-4194-2 | English |
| WOS:000343344900002 | Biomechanics of medial unicondylar in combination with patellofemoral knee arthroplasty | Heyse, TJ; El-Zayat, BF; De Corte, R; Scheys, L; Chevalier, Y; Fuchs-Winkelmann, S; Labey, L | KNEE | | 2014 | 10.1016/S0968-0160(14)50002-6 | English |
| WOS:000259839400001 | Optimizing patellofemoral arthroplasty | Farr, J; Barrett, D | KNEE | | 2008 | 10.1016/j.knee.2008.05.008 | English |
| WOS:000304210900020 | In vivo sagittal plane kinematics of the FPV patellofemoral replacement | Monk, AP; van Duren, BH; Pandit, H; Shakespeare, D; Murray, DW; Gill, HS | KNEE SURGERY SPORTS TRAUMATOLOGY ARTHROSCOPY | | 2012 | 10.1007/s00167-011-1717-4 | English |
| WOS:000185293400026 | Patellofemoral replacement: The third compartment | Argenson, JN | ORTHOPEDICS | | 2003 |  | English |
| WOS:000244121800040 | Custom patellofemoral replacement in the presence of trochlear dysplasia | Grelsamer, RP | JOURNAL OF BONE AND JOINT SURGERY-AMERICAN VOLUME | | 2007 | 10.2106/00004623-200702000-00040 | English |
| WOS:000244121800041 | Custom patellofemoral replacement in the presence of trochlear dysplasia - Reply | Sisto, DJ; Sarin, VK | JOURNAL OF BONE AND JOINT SURGERY-AMERICAN VOLUME | | 2007 | 10.2106/00004623-200702000-00041 | English |
| WOS:000356183500012 | Four-Year Follow Up Outcome Study of Patellofemoral Arthroplasty at a Single Institution | Goh, GSH; Liow, MHL; Tay, DKJ; Lo, NN; Yeo, SJ | JOURNAL OF ARTHROPLASTY | | 2015 | 10.1016/j.arth.2015.01.020 | English |
| WOS:000445730200023 | Patellofemoral Arthroplasty | Godshaw, B; Kolodychuk, N; Williams, GK; Browning, B; Jones, D | OCHSNER JOURNAL | | 2018 | 10.31486/toj.18.0009 | English |
| WOS:000242775500022 | Patellofemoral replacement polymer stress during daily activities: A finite element study | Morra, EA; Greenwald, AS | JOURNAL OF BONE AND JOINT SURGERY-AMERICAN VOLUME | | 2006 | 10.2106/JBJS.F.00585 | English |
| WOS:000403716700006 | PATELLOFEMORAL ARTHROPLASTY CHANGES THE TROCHLEAR GROOVE ANGLE | Wiesner, FJ; Erasmus, PJ; Cho, KJ; Muller, JH | JOURNAL OF MECHANICS IN MEDICINE AND BIOLOGY | | 2017 | 10.1142/S0219519417500683 | English |
| WOS:000330720300004 | Patellofemoral arthroplasty | Lustig, S | ORTHOPAEDICS & TRAUMATOLOGY-SURGERY & RESEARCH | | 2014 | 10.1016/j.otsr.2013.06.013 | English |
| WOS:000331628900029 | Design, operative technique and ten-year results of the Hermes (TM) patellofemoral arthroplasty | Philippe, H; Caton, J | INTERNATIONAL ORTHOPAEDICS | | 2014 | 10.1007/s00264-013-2158-0 | English |
| WOS:000342468800046 | Patellofemoral arthroplasty influences tibiofemoral kinematics: the effect of patellar thickness | Vandenneucker, H; Labey, L; Victor, J; Vander Sloten, J; Desloovere, K; Bellemans, J | KNEE SURGERY SPORTS TRAUMATOLOGY ARTHROSCOPY | | 2014 | 10.1007/s00167-014-3160-9 | English |
| WOS:000258226000011 | Results of total knee replacement for isolated patellofemoral arthritis: When not to perform a patellofemoral arthroplasty | Delanois, RE; McGrath, MS; Ulrich, SD; Marker, DR; Seyler, TM; Bonutti, PM; Mont, MA | ORTHOPEDIC CLINICS OF NORTH AMERICA | | 2008 | 10.1016/j.ocl.2008.03.003 | English |
| WOS:000471034400009 | Return to the operating room after patellofemoral arthroplasty versus total knee arthroplasty for isolated patellofemoral arthritisa systematic review | Woon, CYL; Christ, AB; Goto, R; Shanaghan, K; Shubin Stein, BE; Della Valle, AG | INTERNATIONAL ORTHOPAEDICS | | 2019 | 10.1007/s00264-018-04280-z | English |
| WOS:000384867600027 | The Journey patellofemoral joint arthroplasty: A minimum 5 year follow-up study | Ahearn, N; Metcalfe, AJ; Hassaballa, MA; Porteous, AJ; Robinson, JR; Murray, JR; Newman, JH | KNEE | | 2016 | 10.1016/j.knee.2016.03.004 | English |
| WOS:000320740300011 | Early revisions of the Femoro-Patella Vialla joint replacement | Williams, DP; Pandit, HG; Athanasou, NA; Murray, DW; Gibbons, CLMH | BONE & JOINT JOURNAL | | 2013 | 10.1302/0301-620X.95B6.31355 | English |
| WOS:000339463400012 | Patellofemoral Arthroplasty in the Athlete | Farr, J; Arendt, E; Dahm, D; Daynes, J | CLINICS IN SPORTS MEDICINE | | 2014 | 10.1016/j.csm.2014.03.003 | English |
| WOS:000346066300007 | Biomechanical behaviour of cancellous bone on patellofemoral arthroplasty with Journey prosthesis: a finite element study | Castro, APG; Completo, A; Simoes, JA; Flores, P | COMPUTER METHODS IN BIOMECHANICS AND BIOMEDICAL ENGINEERING | | 2015 | 10.1080/10255842.2013.870999 | English |
| WOS:000430519800015 | Mid-term survivorship and clinical outcomes of the Avon patellofemoral joint replacement | Middleton, SWF; Toms, AD; Schranz, PJ; Mandalia, VI | KNEE | | 2018 | 10.1016/j.knee.2018.01.007 | English |
| WOS:000289311000011 | The low contact stress patellofemoral replacement HIGH EARLY FAILURE RATE | Charalambous, CP; Abiddin, Z; Mills, SP; Rogers, S; Sutton, P; Parkinson, R | JOURNAL OF BONE AND JOINT SURGERY-BRITISH VOLUME | | 2011 | 10.1302/0301-620X.93B4.25899 | English |
| WOS:000865898500001 | Robotic Assisted Patellofemoral Joint Replacement: Surgical Technique, Tips and Tricks | Selvaratnam, V; Toms, AD; Mandalia, VI | INDIAN JOURNAL OF ORTHOPAEDICS | | 2022 | 10.1007/s43465-022-00746-w | English |
| WOS:000250746300014 | Total knee arthroplasty for isolated patellofemoral arthritis in younger patients | Meding, JB; Wing, JT; Keating, EM; Ritter, MA | CLINICAL ORTHOPAEDICS AND RELATED RESEARCH | | 2007 | 10.1097/BLO.0b013e3181576069 | English |
| WOS:000245810900006 | The Avon patellofemoral arthroplasty - Five-year survivorship and functional results | Ackroyd, CE; Newman, JH; Evans, R; Eldridge, JDJ; Joslin, CC | JOURNAL OF BONE AND JOINT SURGERY-BRITISH VOLUME | | 2007 | 10.1302/0301-620X.89B3.18062 | English |
| WOS:000221692300001 | The Lubinus patellofemoral arthroplasty: a series of 17 cases | Board, TN; Mahmood, A; Ryan, WG; Banks, AJ | ARCHIVES OF ORTHOPAEDIC AND TRAUMA SURGERY | | 2004 | 10.1007/s00402-004-0645-x | English |
| WOS:000244265200010 | Outcomes of patellofemoral replacement in total knee arthroplasty using meticulous techniques | Clyburn, TA; Weitz-Marshall, A; Ambrose, CM; Ursua, V | ORTHOPEDICS | | 2007 | 10.3928/01477447-20070201-13 | English |
| WOS:000384867600026 | The effect of axial rotation of the anterior resection plane in patellofemoral arthroplasty | Cho, KJ; Erasmus, PJ; Muller, JH | KNEE | | 2016 | 10.1016/j.knee.2016.04.006 | English |
| WOS:A1994NZ41600001 | THE PATELLOFEMORAL JOINT - ITS FUNCTION AND INFLUENCE IN PATELLAR REPLACEMENT DESIGN | PAPPAS, MJ; BUECHEL, FF | JOURNAL OF ORTHOPAEDIC RHEUMATOLOGY | | 1994 |  | English |
| WOS:000343344900011 | Coronal alignment of patellofemoral arthroplasty | Thienpont, E; Lonner, JH | KNEE | | 2014 | 10.1016/S0968-0160(14)50011-7 | English |
| WOS:000505643000025 | The midterm results of a cohort study of patellofemoral arthroplasty from a non-designer centre using an asymmetric trochlear prosthesis | Rammohan, R; Gupta, S; Lee, PYF; Chandratreya, A | KNEE | | 2019 | 10.1016/j.knee.2019.10.026 | English |
| WOS:000305527400002 | Patellofemoral arthroplasty, where are we today? | Lustig, S; Magnussen, RA; Dahm, DL; Parker, D | KNEE SURGERY SPORTS TRAUMATOLOGY ARTHROSCOPY | | 2012 | 10.1007/s00167-012-1948-z | English |
| WOS:000327135200029 | Patellofemoral replacement THE THIRD COMPARTMENT | Hofmann, AA; McCandless, JB; Shaeffer, JF; Magee, TH | BONE & JOINT JOURNAL | | 2013 | 10.1302/0301-620X.95B11.32985 | English |
| WOS:000472611200029 | CORR Insights (R): What Is the Risk of Repeat Revision When Patellofemoral Replacement Is Revised to TKA? An Analysis of 482 Cases From a Large National Arthroplasty Registry | Hallstrom, BR | CLINICAL ORTHOPAEDICS AND RELATED RESEARCH | | 2019 | 10.1097/CORR.0000000000000583 | English |
| WOS:000356184300024 | Patellar Fracture Following Patellofemoral Arthroplasty | King, AH; Engasser, WM; Sousa, PL; Arendt, EA; Dahm, DL | JOURNAL OF ARTHROPLASTY | | 2015 | 10.1016/j.arth.2015.02.007 | English |
| WOS:000230292900008 | Long-term results with the first patellotemoral prosthesis | Cartier, P; Sanouiller, JL; Khefacha, A | CLINICAL ORTHOPAEDICS AND RELATED RESEARCH | | 2005 | 10.1097/01.blo.0000171918.24998.d1 | English |
| WOS:000225054300026 | Patellofemoral arthroplasty - Pros, cons, and design considerations | Lonner, JH | CLINICAL ORTHOPAEDICS AND RELATED RESEARCH | | 2004 | 10.1097/01.blo.0000148896.25708.51 | English |
| WOS:000184782000010 | Long-term results of patellofemoral arthroplasty - A report of 56 arthroplasties with 17 years of follow-up | Kooijman, HJ; Driessen, APPM; van Horn, JR | JOURNAL OF BONE AND JOINT SURGERY-BRITISH VOLUME | | 2003 | 10.1302/0301-620X.85B6.13741 | English |
| WOS:000313809600008 | Prospective clinical and radiological two-year results after patellofemoral arthroplasty using an implant with an asymmetric trochlea design | Beitzel, K; Schottle, PB; Cotic, M; Dharmesh, V; Imhoff, AB | KNEE SURGERY SPORTS TRAUMATOLOGY ARTHROSCOPY | | 2013 | 10.1007/s00167-012-2022-6 | English |
| WOS:000230292900007 | A modular prosthesis for patellofemoral arthroplasty - Design and initial results | Merchant, AC | CLINICAL ORTHOPAEDICS AND RELATED RESEARCH | | 2005 | 10.1097/01.bio.0000171917.47869.6c | English |
| WOS:000272174300022 | Midterm clinical results of the Autocentric II patellofemoral prosthesis | van Wagenberg, JMF; Speigner, B; Gosens, T; Malefijt, JD | INTERNATIONAL ORTHOPAEDICS | | 2009 | 10.1007/s00264-009-0719-z | English |
| WOS:000387286500034 | Isolated patellofemoral arthroplasty reproduces natural patellofemoral joint kinematics when the patella is resurfaced | Vandenneucker, H; Labey, L; Vander Sloten, J; Desloovere, K; Bellemans, J | KNEE SURGERY SPORTS TRAUMATOLOGY ARTHROSCOPY | | 2016 | 10.1007/s00167-014-3415-5 | English |
| WOS:000538178900002 | The Present Situation of Patellofemoral Arthroplasty in the Management of Solitary Patellofemoral Osteoarthritis | Rodriguez-Merchan, EC | ARCHIVES OF BONE AND JOINT SURGERY-ABJS | | 2020 | 10.22038/ABJS.2019.14125 | English |
| WOS:000370662700009 | Significant Functional Improvement at 2 Years After Isolated Patellofemoral Arthroplasty With an Onlay Trochlear Implant, But Low Mental Health Scores Predispose to Dissatisfaction | Kazarian, GS; Tarity, TD; Hansen, EN; Cai, J; Lonner, JH | JOURNAL OF ARTHROPLASTY | | 2016 | 10.1016/j.arth.2015.08.033 | English |
| WOS:000304231300005 | Functional relevance of patellofemoral thickness before and after unicompartmental patellofemoral replacement | Mofidi, A; Bajada, S; Holt, MD; Davies, AP | KNEE | | 2012 | 10.1016/j.knee.2011.03.002 | English |
| WOS:000310431000057 | Development of a New Femoral Component for Patellofemoral Prosthesis | Castro, A; Flores, P; Completo, A; Simoes, JA | 2012 IEEE 2ND PORTUGUESE MEETING IN BIOENGINEERING (ENBENG) | | 2012 |  | English |
| WOS:000172079900009 | The Richards type II patellofemoral arthroplasty - 26 cases followed for 1-20 years | de Winter, WEAEJM; Feith, R; van Loon, CJM | ACTA ORTHOPAEDICA SCANDINAVICA | | 2001 |  | English |
| WOS:000248916500003 | Patellofemoral arthritis and its management with isolated patellofemoral replacement: A personal experience | Newman, JH | ORTHOPEDICS | | 2007 |  | English |
| WOS:000239289000007 | Arthritis progression after patellofemoral joint replacement | Nicol, SG; Loveridge, JM; Weale, AE; Ackroyd, CE; Newman, JH | KNEE | | 2006 | 10.1016/j.knee.2006.04.005 | English |
| WOS:000244654700018 | In vivo sagittal plane kinematics of the Avon patellofemoral arthroplasty | Hollinghurst, D; Stoney, J; Ward, T; Pandit, H; Beard, D; Murray, DW | JOURNAL OF ARTHROPLASTY | | 2007 | 10.1016/j.arth.2006.02.160 | English |
| WOS:000759085000001 | Combined procedures with unicompartmental knee arthroplasty: High risk of stiffness but promising concept in selected indications | Derreveaux, V; Schmidt, A; Shatrov, J; Sappey-Marinier, E; Batailler, C; Servien, E; Lustig, S | SICOT-J | | 2022 | 10.1051/sicotj/2022002 | English |
| WOS:000707056100009 | Avon patellofemoral arthroplasty. Five year survivorship and functional results from an independent centre | Reeve, WJE; Guyver, PM; Vaughan, AJC; Farmer, K; Lee, AS | ACTA ORTHOPAEDICA BELGICA | | 2021 |  | English |
| WOS:000342468800048 | Evidence of trochlear dysplasia in patellofemoral arthroplasty designs | Saffarini, M; Ntagiopoulos, PG; Demey, G; Le Negaret, B; Dejour, DH | KNEE SURGERY SPORTS TRAUMATOLOGY ARTHROSCOPY | | 2014 | 10.1007/s00167-014-2967-8 | English |
| WOS:000700617600001 | Patient-Reported Outcomes and Risk Factors for Decreased Improvement after Patellofemoral Arthroplasty | Dai, YK; Diao, NC; Lin, W; Yang, GM; Kang, HJ; Wang, F | JOURNAL OF KNEE SURGERY | | 2021 | 10.1055/s-0041-1735159 | English |
| WOS:000848342600003 | Effect of Patellofemoral Arthroplasty on Patellar Height in Patients with Patellofemoral Osteoarthritis | Lee, H; Fletcher, C; Hartwell, M; Strickland, SM | JOURNAL OF KNEE SURGERY | | 2023 | 10.1055/s-0042-1755354 | English |
| WOS:000474380000031 | Early outcomes of an anatomic trochlear-cutting patellofemoral arthroplasty: patient selection is key | Dejour, D; Saffarini, M; Malemo, Y; Pungitore, M; Valluy, J; Nover, L; Demey, G | KNEE SURGERY SPORTS TRAUMATOLOGY ARTHROSCOPY | | 2019 | 10.1007/s00167-019-05368-8 | English |
| WOS:000406189500045 | Survivorship and functional outcomes of patellofemoral arthroplasty: a systematic review | van der List, JP; Chawla, H; Zuiderbaan, HA; Pearle, AD | KNEE SURGERY SPORTS TRAUMATOLOGY ARTHROSCOPY | | 2017 | 10.1007/s00167-015-3878-z | English |
| WOS:000342468800045 | Patellofemoral arthroplasty: outcomes and factors associated with early progression of tibiofemoral arthritis | Dahm, DL; Kalisvaart, MM; Stuart, MJ; Slettedahl, SW | KNEE SURGERY SPORTS TRAUMATOLOGY ARTHROSCOPY | | 2014 | 10.1007/s00167-014-3202-3 | English |
| WOS:000661074600067 | Onlay Patellofemoral Arthroplasty in Patients With Isolated Patellofemoral Arthritis: A Systematic Review | Villa, JC; Paoli, AR; Nelson-Williams, HW; Badr, RN; Harper, KD | JOURNAL OF ARTHROPLASTY | | 2021 | 10.1016/j.arth.2021.02.054 | English |
| WOS:000660624300001 | The short-term effectiveness and safety of second-generation patellofemoral arthroplasty and total knee arthroplasty on isolated patellofemoral osteoarthritis: a systematic review and meta-analysis | Li, CX; Li, ZZ; Shi, LJ; Gao, FQ; Sun, W | JOURNAL OF ORTHOPAEDIC SURGERY AND RESEARCH | | 2021 | 10.1186/s13018-021-02509-z | English |
| WOS:000430469400026 | Inadequacy of computed tomography for pre-operative planning of patellofemoral arthroplasty | Saffarini, M; Muller, JH; La Barbera, G; Hannink, G; Cho, KJ; Toanen, C; Dejour, D | KNEE SURGERY SPORTS TRAUMATOLOGY ARTHROSCOPY | | 2018 | 10.1007/s00167-017-4474-1 | English |
| WOS:000476523700011 | Modelling and simulation of alternative designs for the femur-implant interface of Journey patellofemoral prosthesis | Castro, APG; Completo, A; Simoes, JA; Flores, P | PROCEEDINGS OF THE INSTITUTION OF MECHANICAL ENGINEERS PART L-JOURNAL OF MATERIALS-DESIGN AND APPLICATIONS | | 2019 | 10.1177/1464420718774074 | English |
| WOS:000412610900042 | Patellofemoral arthroplasty conversion to total knee arthroplasty: Retrieval analysis and clinical correlation | Christ, AB; Baral, E; Koch, C; Stein, BES; Della Valle, AG; Strickland, SM | KNEE | | 2017 | 10.1016/j.knee.2017.06.015 | English |
| WOS:000425893000012 | Mid-Term Clinical, Functional, and Radiographic Outcomes of 105 Gender-Specific Patellofemoral Arthroplasties, With or Without the Association of Medial Unicompartmental Knee Arthroplasty | Romagnoli, S; Marullo, M | JOURNAL OF ARTHROPLASTY | | 2018 | 10.1016/j.arth.2017.10.019 | English |
| WOS:000486087800008 | Patient satisfaction reporting for patellofemoral arthroplasty is significantly lacking: a systematic review | Tishelman, JC; Kahlenberg, CA; Nwachukwu, BU; Gruskay, J; Strickland, SM | PHYSICIAN AND SPORTSMEDICINE | | 2019 | 10.1080/00913847.2019.1580913 | English |
| WOS:000561189500020 | Patellofemoral Arthroplasty Surgical Technique: Lateral or Medial Parapatellar Approach | Jeong, SH; Schneider, B; Pyne, AS; Tishelman, JC; Strickland, SM | JOURNAL OF ARTHROPLASTY | | 2020 | 10.1016/j.arth.2020.04.026 | English |
| WOS:000459152300008 | Patellofemoral arthroplasty versus total knee arthroplasty for patients with patellofemoral osteoarthritis | Clement, ND; Howard, TA; Immelman, RJ; MacDonald, D; Patton, JT; Lawson, GM; Burnett, R | BONE & JOINT JOURNAL | | 2019 | 10.1302/0301-620X.101B1.BJJ-2018-0654.R2 | English |
| WOS:000400679700018 | Modular bicompartmental knee arthroplasty : Indications, technique, prosthetic design, and results | Kanna, R | ACTA ORTHOPAEDICA BELGICA | | 2017 |  | English |
| WOS:000221020600006 | Trochlear resurfacing for extensor mechanism instability following patellectomy | Ackroyd, CE; Smith, EJ; Newman, JH | KNEE | | 2004 | 10.1016/S0968-0160(03)00079-6 | English |
| WOS:000416662100007 | Correction of Patellofemoral Malalignment With Patellofemoral Arthroplasty | Valoroso, M; Saffarini, M; La Barbera, G; Toanen, C; Hannink, G; Nover, L; Dejour, DH | JOURNAL OF ARTHROPLASTY | | 2017 | 10.1016/j.arth.2017.06.048 | English |
| WOS:000876838900001 | Patellofemoral arthroplasty in combination with high tibial osteotomy can achieve good outcome for patients with medial-patellofemoral osteoarthritis | Peng, YG; Lin, W; Zhang, YF; Wang, F | FRONTIERS IN SURGERY | | 2022 | 10.3389/fsurg.2022.999208 | English |
| WOS:000373418800029 | Obesity and the absence of trochlear dysplasia increase the risk of revision in patellofemoral arthroplasty | Liow, MHL; Goh, GSH; Tay, DKJ; Chia, SL; Lo, NN; Yeo, SJ | KNEE | | 2016 | 10.1016/j.knee.2015.05.009 | English |
| WOS:000564264400002 | Patellofemoral Arthroplasty Improves Patellofemoral Alignment in Patients with Patellofemoral Osteoarthritis with Trochlear Dysplasia | Yang, GM; Wang, J; Dai, YK; Lin, W; Niu, JH; Wang, F | JOURNAL OF KNEE SURGERY | | 2022 | 10.1055/s-0040-1715099 | English |
| WOS:000401125600018 | Outcomes of Patellofemoral Arthroplasty Based on Radiographic Severity | deDeugd, CM; Pareek, A; Krych, AJ; Cummings, NM; Dahm, DL | JOURNAL OF ARTHROPLASTY | | 2017 | 10.1016/j.arth.2016.11.006 | English |
| WOS:000238701800007 | Custom patellofemoral arthroplasty of the knee | Sisto, DJ; Sarin, VK | JOURNAL OF BONE AND JOINT SURGERY-AMERICAN VOLUME | | 2006 | 10.2106/JBJS.E.00382 | English |
| WOS:000470052400027 | Hermes patellofemoral arthroplasty: Annual revision rate and clinical results after two to 20 years of follow-up | Bohu, Y; Klouche, S; Sezer, HB; Gerometta, A; Lefevre, N; Herman, S | KNEE | | 2019 | 10.1016/j.knee.2019.01.014 | English |
| WOS:000408407200019 | A matched-pair comparison of inlay and onlay trochlear designs for patellofemoral arthroplasty: no differences in clinical outcome but less progression of osteoarthritis with inlay designs | Feucht, MJ; Cotic, M; Beitzel, K; Baldini, JF; Meidinger, G; Schottle, PB; Imhoff, AB | KNEE SURGERY SPORTS TRAUMATOLOGY ARTHROSCOPY | | 2017 | 10.1007/s00167-015-3733-2 | English |
| WOS:000392622100002 | Midterm Outcome of Avon Patellofemoral Arthroplasty for Posttraumatic Unicompartmental Osteoarthritis | Konan, S; Haddad, FS | JOURNAL OF ARTHROPLASTY | | 2016 | 10.1016/j.arth.2016.06.005 | English |
| WOS:000232178200007 | Is anterior knee pain a predisposing factor to patellofemoral osteoarthritis? | Utting, MR; Davies, G; Newman, JH | KNEE | | 2005 | 10.1016/j.knee.2004.12.006 | English |
| WOS:A1996VG55100017 | Patellofemoral arthroplasty - A 2- to 18-year followup study | KrajcaRadcliffe, JB; Coker, TP | CLINICAL ORTHOPAEDICS AND RELATED RESEARCH | | 1996 | 10.1097/00003086-199609000-00017 | English |
| WOS:000363600800003 | Long-term results of compartmental arthroplasties of the knee LONG TERM RESULTS OF PARTIAL KNEE ARTHROPLASTY | Parratte, S; Ollivier, M; Lunebourg, A; Abdel, MP; Argenson, JN | BONE & JOINT JOURNAL | | 2015 | 10.1302/0301-620X.97B10.36426 | English |
| WOS:000352740400034 | TROCHLEAR GROOVE ALIGNMENT MEASUREMENT METHOD FOR SURGICAL APPLICATIONS | Cho, KJ; Muller, JH; Erasmus, PJ | JOURNAL OF MECHANICS IN MEDICINE AND BIOLOGY | | 2015 | 10.1142/S0219519415400333 | English |
| WOS:000273877900004 | Return to work following knee arthroplasty | Foote, JAJ; Smith, HK; Jonas, SC; Greenwood, R; Weale, AE | KNEE | | 2010 | 10.1016/j.knee.2009.06.001 | English |
| WOS:000294176400011 | Medium term results of Avon patellofemoral joint replacement | Sarda, PK; Shetty, A; Maheswaran, SS | INDIAN JOURNAL OF ORTHOPAEDICS | | 2011 | 10.4103/0019-5413.83761 | English |
| WOS:000258226000010 | Patellofemoral arthroplasty in the treatment of patellofemoral arthritis: Rationale and outcomes in younger patients | Leadbetter, WB | ORTHOPEDIC CLINICS OF NORTH AMERICA | | 2008 | 10.1016/j.ocl.2008.04.001 | English |
| WOS:000564916000011 | Patellofemoral Arthroplasty: Short-Term Complications and Risk Factors | Rezzadeh, K; Behery, OA; Kester, BS; Dogra, T; Vigdorchik, J; Schwarzkopf, R | JOURNAL OF KNEE SURGERY | | 2020 | 10.1055/s-0039-1688960 | English |
| WOS:000704442700036 | Editorial Commentary: Is the Pendulum Swinging Away From Patellar Fixation in Medial Patellofemoral Ligament Reconstruction? | Sherman, SL; Curtis, DM | ARTHROSCOPY-THE JOURNAL OF ARTHROSCOPIC AND RELATED SURGERY | | 2021 | 10.1016/j.arthro.2021.06.001 | English |
| WOS:000258964700017 | Surgery for osteoarthritis of the knee | Richmond, JC | RHEUMATIC DISEASE CLINICS OF NORTH AMERICA | | 2008 | 10.1016/j.rdc.2008.05.010 | English |
| WOS:000398873800003 | Annual revision rates of partial versus total knee arthroplasty: A comparative meta-analysis | Chawla, H; van der List, JP; Christ, AB; Sobrero, MR; Zuiderbaan, HA; Pearle, AD | KNEE | | 2017 | 10.1016/j.knee.2016.11.006 | English |
| WOS:000230292900003 | Development and early results of a new patellofemoral arthroplasty | Ackroyd, CE | CLINICAL ORTHOPAEDICS AND RELATED RESEARCH | | 2005 | 10.1097/01.blo.0000171914.94503.d1 | English |
| WOS:000262339100014 | Surgery for Osteoarthritis of the Knee | Richmond, JC | MEDICAL CLINICS OF NORTH AMERICA | | 2009 | 10.1016/j.mcna.2008.09.012 | English |
| WOS:A1996UJ01700008 | Unicompartmental knee arthroplasty with patelloplasty | Antoniou, J; Hadjipavlou, A; Enker, P; Antoniou, A | INTERNATIONAL ORTHOPAEDICS | | 1996 | 10.1007/s002640050038 | English |
| WOS:000927937200001 | A Newly Identified Complication of Patellofemoral Arthroplasty: Case Report and Literature Review | Solarino, G; Maccagnano, G; Vicenti, G; Buono, C; Simone, F; Ottaviani, G; Zavattini, G; Zaccari, D; Carrozzo, M; Spinarelli, A; Bizzoca, D; Moretti, B | GERIATRIC ORTHOPAEDIC SURGERY & REHABILITATION | | 2022 | 10.1177/21514593221138662 | English |
| WOS:000473954400001 | Failure modes of patellofemoral arthroplasty-registries vs. clinical studies: a systematic review | Bendixen, NB; Eskelund, PW; Odgaard, A | ACTA ORTHOPAEDICA | | 2019 | 10.1080/17453674.2019.1634865 | English |
| WOS:000273805000009 | The Avon patellofemoral joint replacement FIVE-YEAR RESULTS FROM AN INDEPENDENT CENTRE | Odumenya, M; Costa, ML; Parsons, N; Achten, J; Dhillon, M; Krikler, SJ | JOURNAL OF BONE AND JOINT SURGERY-BRITISH VOLUME | | 2010 | 10.1302/0301-620X.92B1.23135 | English |
| WOS:000630971000001 | Joint Awareness after Patellofemoral Arthroplasty Evaluated with the Forgotten Joint Score: A Comparison Study | Lin, W; Dai, YK; Dong, CL; Piao, K; Hao, K; Wang, F | ORTHOPAEDIC SURGERY | | 2021 | 10.1111/os.12921 | English |
| WOS:000497799000001 | Smoking, unemployment, female sex, obesity, and medication use yield worse outcomes in patellofemoral arthroplasty | Desai, VS; Pareek, A; DeDeugd, CM; Sabbag, OD; Krych, AJ; Cummings, NM; Dahm, DL | KNEE SURGERY SPORTS TRAUMATOLOGY ARTHROSCOPY | | 2020 | 10.1007/s00167-019-05704-y | English |
| WOS:000774604300001 | Patellofemoral arthroplasty: obesity linked to high risk of revision and progression of medial tibiofemoral osteoarthritis | Marullo, M; Bargagliotti, M; Vigano, M; Lacagnina, C; Romagnoli, S | KNEE SURGERY SPORTS TRAUMATOLOGY ARTHROSCOPY | | 2022 | 10.1007/s00167-022-06947-y | English |
| WOS:000584483900002 | Robotic-Assisted Patellofemoral Replacement-Correlation of Preoperative Planning with Intraoperative Implant Position and Early Clinical Experience: A Minimum 2-Year Follow-up | Selvaratnam, V; Cattell, A; Eyres, KS; Toms, AD; Phillips, JRP; Mandalia, VI | JOURNAL OF KNEE SURGERY | | 2022 | 10.1055/s-0040-1716848 | English |
| WOS:000477976500007 | Classification of combined partial knee arthroplasty | Garner, A; van Arkel, RJ; Cobb, J | BONE & JOINT JOURNAL | | 2019 | 10.1302/0301-620X.101B8.BJJ-2019-0125.R1 | English |
| WOS:000234624700012 | MBARS: mini bone-attached robotic system for joint arthroplasty | Wolf, A; Jaramaz, B; Lisien, B; DiGioia, AM | INTERNATIONAL JOURNAL OF MEDICAL ROBOTICS AND COMPUTER ASSISTED SURGERY | | 2005 | 10.1581/mrcas.2005.010210 | English |
| WOS:000532080900001 | Patellofemoral arthritis Plea for a customized therapy | Keshmiri, A; Imhoff, AB; Dirisamer, F | ARTHROSKOPIE | | 2020 | 10.1007/s00142-020-00379-x | German |
| WOS:000641481700002 | Patellofemoral arthroplasty versus total knee arthroplasty for isolated patellofemoral osteoarthritis: a systematic review and meta-analysis | Peng, GR; Liu, M; Guan, ZH; Hou, YF; Liu, Q; Sun, XB; Zhu, XY; Feng, WJ; Zeng, JC; Zhong, ZR; Zeng, YR | JOURNAL OF ORTHOPAEDIC SURGERY AND RESEARCH | | 2021 | 10.1186/s13018-021-02414-5 | English |
| WOS:000474380000024 | Similar postoperative patient-reported outcome in both second generation patellofemoral arthroplasty and total knee arthroplasty for treatment of isolated patellofemoral osteoarthritis: a systematic review | Bunyoz, KI; Lustig, S; Troelsen, A | KNEE SURGERY SPORTS TRAUMATOLOGY ARTHROSCOPY | | 2019 | 10.1007/s00167-018-5151-8 | English |
| WOS:000646477200001 | Patient-specific instrumentation and partial replacement of the knee | Benignus, C; Meier, MK; Hirschmann, MT; Tibesku, CO; Beckmann, J | ARTHROSKOPIE | | 2021 | 10.1007/s00142-021-00463-w | German |
| WOS:000772267600001 | Rotational alignment of the femoral trochlea in Asians: Implication on implant choice and position for managing isolated patellofemoral osteoarthritis | Li, MKL; Wan, SYC; Lo, KCH; Hung, YW; Fan, JCH | JOURNAL OF ORTHOPAEDICS TRAUMA AND REHABILITATION | | 2022 | 10.1177/22104917221085718 | English |
| WOS:000735445800017 | Arthroscopic Debridement, Facetectomy, and Synovectomy for Isolated Patellofemoral Osteoarthritis | Zhao, JZ | ARTHROSCOPY TECHNIQUES | | 2021 | 10.1016/j.eats.2021.08.021 | English |
| WOS:000209167700006 | The Basic Science of the Patella: Structure, Composition, and Function | Fox, AJS; Wanivenhaus, F; Rodeo, SA | JOURNAL OF KNEE SURGERY | | 2012 | 10.1055/s-0032-1313741 | English |
| WOS:000307670000063 | MRI after patellofemoral replacement: The preserved compartments | Heyse, TJ; Figiel, J; Hahnlein, U; Timmesfeld, N; Lakemeier, S; Schofer, MD; Fuchs-Winkelmann, S; Efe, T | EUROPEAN JOURNAL OF RADIOLOGY | | 2012 | 10.1016/j.ejrad.2011.06.012 | English |
| WOS:000780879800001 | Patellar button compatibility in the conversion of Patellofemoral Arthroplasty to a Total Knee Arthroplasty: A review of the contemporary literature | McDonald, LK; Kurmis, AP | JOURNAL OF ORTHOPAEDIC SURGERY | | 2022 | 10.1177/10225536221084147 | English |
| WOS:000395359800002 | Why do patellofemoral arthroplasties fail today? A systematic review | van der List, JP; Chawla, H; Villa, JC; Pearle, AD | KNEE | | 2017 | 10.1016/j.knee.2015.11.002 | English |
| WOS:000307748300009 | Complications in Patellofemoral Surgery | Tompkins, M; Arendt, EA | SPORTS MEDICINE AND ARTHROSCOPY REVIEW | | 2012 | 10.1097/JSA.0b013e31825c74cf | English |
| WOS:000322847200004 | Bicompartmental Knee Arthroplasty: The Clinical Outcomes | Tria, AJ | ORTHOPEDIC CLINICS OF NORTH AMERICA | | 2013 | 10.1016/j.ocl.2013.03.003 | English |
| WOS:000886540900012 | Knee arthroplasty utilization trends from 2010 to 2019 | Fuller, SI; Cohen, JS; Malyavko, A; Agarwal, AR; Stake, S; Golladay, GJ; Thakkar, SC | KNEE | | 2022 | 10.1016/j.knee.2022.09.006 | English |
| WOS:000360436200029 | A 21 % conversion rate to total knee arthroplasty of a first-generation patellofemoral prosthesis at a mean follow-up of 9.7 years | Hoogervorst, P; de Jong, RJ; Hannink, G; van Kampen, A | INTERNATIONAL ORTHOPAEDICS | | 2015 | 10.1007/s00264-015-2941-1 | English |
| WOS:000258025300006 | Pre-operative mental wellbeing and the outcome of knee replacement | Walton, MJ; Newman, JH | KNEE | | 2008 | 10.1016/j.knee.2008.03.001 | English |
| WOS:000311619700012 | The correct rotation of the femoral component in patellofemoral replacement A LABORATORY ASSESSMENT OF A SURGICAL TECHNIQUE | Clark, DA; Upadhyay, N; Gillespie, G; Wakeley, C; Eldridge, JD | JOURNAL OF BONE AND JOINT SURGERY-BRITISH VOLUME | | 2012 | 10.1302/0301-620X.94B12.29506 | English |
| WOS:000329086500015 | Outcomes and Complications of Unicondylar Arthroplasty | Riff, AJ; Sah, AP; Della Valle, CJ | CLINICS IN SPORTS MEDICINE | | 2014 | 10.1016/j.csm.2013.06.005 | English |
| WOS:000489692100016 | The Combination of Inlay Patellofemoral Arthroplasty and Medial Unicompartmental Knee Arthroplasty Versus Total Knee Arthroplasty for Mediopatellofemoral Osteoarthritis: A Comparison of Mid-Term Outcomes | Uluyardimci, E; Isik, C; Tahta, M; Emre, F; Cepni, S; Oltulu, I | JOURNAL OF ARTHROPLASTY | | 2019 | 10.1016/j.arth.2019.06.043 | English |
| WOS:000256769200004 | In vitro simulation and quantification of wear within the patellofemoral joint replacement | Ellison, P; Barton, DC; Esler, C; Shaw, DL; Stone, MH; Fisher, J | JOURNAL OF BIOMECHANICS | | 2008 | 10.1016/j.jbiomech.2008.02.029 | English |
| WOS:000646364700008 | Mid- to long-term follow-up of combined small implants A THIRD-GENERATION PATELLOFEMORAL ARTHROPLASTY ASSOCIATED WITH A MEDIAL OR LATERAL UNICOMPARTMENTAL KNEE ARTHROPLASTY | Rossi, SMP; Perticarini, L; Clocchiatti, S; Ghiara, M; Benazzo, F | BONE & JOINT JOURNAL | | 2021 | 10.1302/0301-620X.103B5.BJJ-2020-0720.R3 | English |
| WOS:000457113200018 | Surgical technique in patellofemoral arthroplasty | Remy, F | ORTHOPAEDICS & TRAUMATOLOGY-SURGERY & RESEARCH | | 2019 | 10.1016/j.otsr.2018.05.020 | English |
| WOS:000518776800007 | The PAT randomized clinical trial TOTAL KNEE ARTHROPLASTY VERSUS PATELLOFEMORAL ARTHROPLASTY IN PATIENTS WITH SEVERE ARTHRITIS OF THE PATELLOFEMORAL JOINT | Joseph, MN; Achten, J; Parsons, NR; Costa, ML | BONE & JOINT JOURNAL | | 2020 | 10.1302/0301-620X.102B3.BJJ-2019-0723.R1 | English |
| WOS:000349783700005 | Arthroplasty - current strategies for the management of knee osteoarthritis | Ahmad, SS; Gantenbein, B; Evangelopoulos, DS; Schwienbacher, S; Schar, MO; Kohlhof, H; Kohl, S | SWISS MEDICAL WEEKLY | | 2015 | 10.4414/smw.2015.14096 | English |
| WOS:000244445100182 | MBARS: Mini bone attached robotic system for joint arthroplasty | Wolf, A; Jaramaz, B | 2006 1ST IEEE RAS-EMBS INTERNATIONAL CONFERENCE ON BIOMEDICAL ROBOTICS AND BIOMECHATRONICS, VOLS 1-3 | | 2006 |  | English |
| WOS:000458361500018 | Treatment with custom partial condyle prosthesis of a comminuted femoral condyle fracture in a dog: a case report | Altunatmaz, K; Yalin, EE; Gunay, BI; Mckinstry, DJS; Sevim, ZT | TURKISH JOURNAL OF VETERINARY & ANIMAL SCIENCES | | 2019 | 10.3906/vet-1807-115 | English |
| WOS:000585893300003 | Preoperative patellofemoral anatomy affects failure rate after isolated patellofemoral inlay arthroplasty | Feucht, MJ; Lutz, PM; Ketzer, C; Rupp, MC; Cotic, M; Imhoff, AB; Pogorzelski, J | ARCHIVES OF ORTHOPAEDIC AND TRAUMA SURGERY | | 2020 | 10.1007/s00402-020-03651-9 | English |
| WOS:001043681700001 | Conversion Total Knee Arthroplasty | Ramamurti, P; Fassihi, SC; Stake, S; Stadecker, M; Whiting, Z; Thakkar, SC | JBJS REVIEWS | | 2021 | 10.2106/JBJS.RVW.20.00198 | English |
| WOS:000230292900013 | Alternatives to patellofemoral arthroplasty | Fulkerson, JP | CLINICAL ORTHOPAEDICS AND RELATED RESEARCH | | 2005 | 10.1097/01.bio.0000172305.20156.ba | English |
| WOS:000233788700006 | Anterior knee pain: Diagnosis and treatment | Post, WR | JOURNAL OF THE AMERICAN ACADEMY OF ORTHOPAEDIC SURGEONS | | 2005 | 10.5435/00124635-200512000-00006 | English |
| WOS:000230292900002 | Patellofemoral replacement (reprinted from Clin. Orthop vol. 144, pg 98-102, 1979) | Blazina, ME; Fox, JM; Del Pizzo, W; Broukhim, B; Ivey, FM | CLINICAL ORTHOPAEDICS AND RELATED RESEARCH | | 2005 | 10.1097/01.blo.0000172301.66790.62 | English |
| WOS:000523258700009 | Patellofemoral arthroplasty is cheaper and more effective in the short term than total knee arthroplasty for isolated patellofemoral osteoarthritis: cost-effectiveness analysis based on a randomized trial | Fredborg, C; Odgaard, A; Sorensen, J | BONE & JOINT JOURNAL | | 2020 | 10.1302/0301-620X.102B4.BJJ-2018-1580.R3 | English |
| WOS:000711694000010 | CONVERSION TOTAL KNEE ARTHROPLASTY | Ramamurti, P; Fassihi, SC; Stake, S; Stadecker, M; Whiting, Z; Thakkar, SC | JBJS REVIEWS | | 2021 | 10.2106/JBJS.RVW.20.00198 | English |
| WOS:000382573400002 | Knee osteoarthritis: a review of management options | Hussain, SM; Neilly, DW; Baliga, S; Patil, S; Meek, RMD | SCOTTISH MEDICAL JOURNAL | | 2016 | 10.1177/0036933015619588 | English |
| WOS:000459149700007 | The Avon patellofemoral joint arthroplasty TWO- TO 18-YEAR RESULTS OF A LARGE SINGLE-CENTRE COHORT | Metcalfe, AJ; Ahearn, N; Hassaballa, MA; Parsons, N; Ackroyd, CE; Murray, JR; Robinson, JR; Eldridge, JD; Porteous, AJ | BONE & JOINT JOURNAL | | 2018 | 10.1302/0301-620X.100B9.BJJ-2018-0174.R1 | English |
| WOS:000267063700014 | HyBAR: hybrid bone-attached robot for joint arthroplasty | Song, S; Mor, A; Jaramaz, B | INTERNATIONAL JOURNAL OF MEDICAL ROBOTICS AND COMPUTER ASSISTED SURGERY | | 2009 | 10.1002/rcs.254 | English |
| WOS:000172796500009 | Operative management of patellofemoral pain with degenerative arthrosis | Herrenbruck, TM; Mullen, DJ; Parker, RD | SPORTS MEDICINE AND ARTHROSCOPY REVIEW | | 2001 | 10.1097/00132585-200110000-00009 | English |
| WOS:000480692200004 | PATELLOFEMORAL ARTHROPLASTY | Odgaard, A; Eldridge, J; Madsen, F | JBJS ESSENTIAL SURGICAL TECHNIQUES | | 2019 | 10.2106/JBJS.ST.18.00094 | English |
| WOS:000710597500001 | Medial bicompartmental arthroplasty patients display more normal gait and improved satisfaction, compared to matched total knee arthroplasty patients | Garner, AJ; Dandridge, OW; van Arkel, RJ; Cobb, JP | KNEE SURGERY SPORTS TRAUMATOLOGY ARTHROSCOPY | | 2021 | 10.1007/s00167-021-06773-8 | English |
| WOS:000452898500014 | Modeling and simulation of revolute clearance joint with friction using the NURBS-based isogeometric analysis | Pi, T; Zhang, YQ | NONLINEAR DYNAMICS | | 2019 | 10.1007/s11071-018-4559-5 | English |
| WOS:000170051900015 | The lubinus patellofemoral arthroplasty - A five- to ten-year prospective study | Tauro, B; Ackroyd, CE; Newman, JH; Shah, NA | JOURNAL OF BONE AND JOINT SURGERY-BRITISH VOLUME | | 2001 | 10.1302/0301-620X.83B5.11577 | English |
| WOS:000720249900001 | Insufficient evidence to confirm benefits of custom partial knee arthroplasty: a systematic review | Demey, G; Muller, JH; Liebensteiner, M; Pilot, P; Nover, L; Kort, N | KNEE SURGERY SPORTS TRAUMATOLOGY ARTHROSCOPY | | 2022 | 10.1007/s00167-021-06766-7 | English |
| WOS:000589425600024 | No Major Functional Benefit After Bicompartmental Knee Arthroplasty Compared to Total Knee Arthroplasty at 5-Year Follow-Up | Schrednitzki, D; Beier, A; Marx, A; Halder, AM | JOURNAL OF ARTHROPLASTY | | 2020 | 10.1016/j.arth.2020.07.003 | English |
| WOS:000329318600010 | Revision cartilage cell transplantation for failed autologous chondrocyte transplantation in chronic osteochondral defects of the knee | Vijayan, S; Bentley, G; Rahman, J; Briggs, TWR; Skinner, JA; Carrington, RWJ | BONE & JOINT JOURNAL | | 2014 | 10.1302/0301-620X.96B1.31979 | English |
| WOS:000337692500019 | Arthroplasty of the Femoropatellar Joint - What Data are Available? | Fink, B; Schwenninger, C | ZEITSCHRIFT FUR ORTHOPADIE UND UNFALLCHIRURGIE | | 2014 | 10.1055/s-0033-1360353 | German |
| WOS:000468459400019 | A high level of satisfaction after bicompartmental individualized knee arthroplasty with patient-specific implants and instruments | Ogura, T; Le, K; Merkely, G; Bryant, T; Minas, T | KNEE SURGERY SPORTS TRAUMATOLOGY ARTHROSCOPY | | 2019 | 10.1007/s00167-018-5155-4 | English |
| WOS:000311329300016 | Aseptic loosening of the patellar component at the cement-implant interface | Rath, NK; Dudhniwala, AG; White, SP; Forster, MC | KNEE | | 2012 | 10.1016/j.knee.2011.08.006 | English |
| WOS:000437254300012 | Patient-reported outcome measures (PROMs) in patients undergoing patellofemoral arthroplasty and total knee replacement: A comparative study | Perrone, FL; Baron, S; Suero, EM; Lausmann, C; Kendof, D; Zahar, A; Gehrke, T; Citak, M | TECHNOLOGY AND HEALTH CARE | | 2018 | 10.3233/THC-181185 | English |
| WOS:000174223600007 | Treatment of patello-femoral arthritis using the Lubinus patello-femoral arthroplasty: A retrospective review | Smith, AM; Peckett, WRC; Butler-Manuel, PA; Venu, KM; d'Arcy, JC | KNEE | | 2002 | 10.1016/S0968-0160(01)00127-2 | English |
| WOS:000540814700014 | Short-term Revision Risk of Patellofemoral Arthroplasty Is High: An Analysis from Eight Large Arthroplasty Registries | Lewis, PL; Tudor, F; Lorimer, M; McKie, J; Bohm, E; Robertsson, O; Makela, KT; Haapakoski, J; Furnes, O; Bartz-Johannessen, C; Nelissen, RGHH; Van Steenbergen, LN; Fithian, DC; Prentice, HA | CLINICAL ORTHOPAEDICS AND RELATED RESEARCH | | 2020 | 10.1097/CORR.0000000000001268 | English |
| WOS:000416144000026 | High mid-term revision rate after treatment of large, full-thickness cartilage lesions and OA in the patellofemoral joint using a large inlay resurfacing prosthesis: HemiCAP-WaveA (R) | Laursen, JO | KNEE SURGERY SPORTS TRAUMATOLOGY ARTHROSCOPY | | 2017 | 10.1007/s00167-016-4352-2 | English |
| WOS:000274767200011 | Survival of Bicompartmental Knee Arthroplasty at 5 to 23 Years | Parratte, S; Pauly, V; Aubaniac, JM; Argenson, JNA | CLINICAL ORTHOPAEDICS AND RELATED RESEARCH | | 2010 | 10.1007/s11999-009-1018-0 | English |
| WOS:000083198200011 | Total knee replacement for patients with patellofemoral arthritis | Laskin, RS; van Steijn, M | CLINICAL ORTHOPAEDICS AND RELATED RESEARCH | | 1999 |  | English |
| WOS:000705474000003 | Unicompartmental vs. segmental bicompartmental vs. total knee replacement: comparison of clinical outcomes | Al-Dadah, O; Hawes, G; Chapman-Sheath, PJ; Tice, JW; Barrett, DS | KNEE SURGERY & RELATED RESEARCH | | 2020 | 10.1186/s43019-020-00065-0 | English |
| WOS:000305852300035 | Patellofemoral osteoarthritis treated by partial lateral facetectomy: Results at long-term follow up | Wetzels, T; Bellemans, J | KNEE | | 2012 | 10.1016/j.knee.2011.04.005 | English |
| WOS:000376415700017 | Patella tracking and patella contact pressure in modular patellofemoral arthroplasty: a biomechanical in vitro analysis | Calliess, T; Ettinger, M; Schado, S; Becher, C; Hurschler, C; Ostermeier, S | ARCHIVES OF ORTHOPAEDIC AND TRAUMA SURGERY | | 2016 | 10.1007/s00402-016-2451-7 | English |
| WOS:000209171200017 | Mobile-bearing, congruent patellofemoral prosthesis: short-term results | Yadav, B; Shaw, D; Radcliffe, G; Dachepalli, S; Kluge, W | JOURNAL OF ORTHOPAEDIC SURGERY | | 2012 | 10.1177/230949901202000317 | English |
| WOS:000332829900002 | Knee osteoarthritis and role for surgical intervention: lessons learned from randomized clinical trials and population-based cohorts | Buchbinder, R; Richards, B; Harris, I | CURRENT OPINION IN RHEUMATOLOGY | | 2014 | 10.1097/BOR.0000000000000022 | English |
| WOS:A1992JT97500004 | MORPHOLOGICAL AND NUMERICAL INVESTIGATIONS INTO THE STRESS COMPATIBILITY OF PATELLAR IMPLANTS | LENGSFELD, M; WEISS, H; KIENAPFEL, H | BIOMEDIZINISCHE TECHNIK | | 1992 | 10.1515/bmte.1992.37.10.222 | German |
| WOS:000276361700008 | Isolated patellofemoral osteoarthritis | van Jonbergen, HPW; Poolman, RW; van Kampen, A | ACTA ORTHOPAEDICA | | 2010 | 10.3109/17453671003628756 | English |
| WOS:000447411100021 | Simulation of planar mechanisms with revolute clearance joints using the multipatch based isogeometric analysis | Pi, T; Zhang, YQ | COMPUTER METHODS IN APPLIED MECHANICS AND ENGINEERING | | 2019 | 10.1016/j.cma.2018.08.039 | English |
| WOS:000266568500006 | Surgical options for patients with osteoarthritis of the knee | Lutzner, J; Kasten, P; Gunther, KP; Kirschner, S | NATURE REVIEWS RHEUMATOLOGY | | 2009 | 10.1038/nrrheum.2009.88 | English |
| WOS:000357785300001 | Patellectomy for osteoarthritis: a new tension preserving surgical technique to reconstruct the extensor mechanism with retrospective review of long-term follow-up | Asopa, V; Willis-Owen, C; Keene, G | JOURNAL OF ORTHOPAEDIC SURGERY AND RESEARCH | | 2015 | 10.1186/s13018-015-0237-1 | English |
| WOS:000487280200008 | Solutions for frequent complications of partial knee arthroplasty | Mathis, DT; Hirschmann, MT | ARTHROSKOPIE | | 2019 | 10.1007/s00142-019-00300-1 | German |
| WOS:000335619500032 | Association and impact of patellofemoral dysplasia on patellofemoral arthropathy and arthroplasty | Mofidi, A; Veravalli, K; Jinnah, RH; Poehling, GG | KNEE | | 2014 | 10.1016/j.knee.2013.09.009 | English |
| WOS:000394464100005 | Patellofemoral arthroplasty | Cotic, M; Forkel, P; Imhoff, AB | OPERATIVE ORTHOPADIE UND TRAUMATOLOGIE | | 2017 | 10.1007/s00064-016-0477-1 | German |
| WOS:000505643000027 | Early post-operative opioid consumption: A comparison between medial unicompartmental, patellofemoral, and total knee arthroplasty | Melnic, CM; Kazarian, ER; Dwyer, MK; Domingo-Johnson, EL; Freiberg, AA; Bedair, HS | KNEE | | 2019 | 10.1016./j.knee.2019.06.016 | English |
| WOS:000408554000039 | Pin Site Complications Associated With Computer-Assisted Navigation in Hip and Knee Arthroplasty | Kamara, E; Berliner, ZP; Hepinstall, MS; Cooper, HJ | JOURNAL OF ARTHROPLASTY | | 2017 | 10.1016/j.arth.2017.03.073 | English |
| WOS:000353829500006 | Prospective evaluation of anatomic patellofemoral inlay resurfacing: clinical, radiographic, and sports-related results after 24 months | Imhoff, AB; Feucht, MJ; Meidinger, G; Schottle, P; Cotic, M | KNEE SURGERY SPORTS TRAUMATOLOGY ARTHROSCOPY | | 2015 | 10.1007/s00167-013-2786-3 | English |
| WOS:000619609500024 | Use of Natural Language Processing Algorithms to Identify Common Data Elements in Operative Notes for Knee Arthroplasty | Sagheb, E; Ramazanian, T; Tafti, AP; Fu, SY; Kremers, WK; Berry, DJ; Lewallen, DG; Sohn, S; Kremers, HM | JOURNAL OF ARTHROPLASTY | | 2021 | 10.1016/j.arth.2020.09.029 | English |
| WOS:000377428600009 | Short-term outcomes of robotically assisted patello-femoral arthroplasty | Turktas, U; Piskin, A; Poehling, GG | INTERNATIONAL ORTHOPAEDICS | | 2016 | 10.1007/s00264-015-2786-7 | English |
| WOS:000518203000001 | Practical guidelines for the treatment of patellar fractures in adults | Sylvain, S; Alexandre, B; Jules, C; Francois, C; Olivier, B; Eric, T | SWISS MEDICAL WEEKLY | | 2020 | 10.4414/smw.2020.20165 | English |
| WOS:000672063500001 | Does bicompartmental knee arthroplasty hold an advantage over total knee arthroplasty? Systematic review and meta-analysis | Elbardesy, H; Awad, AK; McLeod, A; Farahat, ST; Sayed, SZE; Guerin, S; Harty, J | SICOT-J | | 2021 | 10.1051/sicotj/2021036 | English |
| WOS:000600605600026 | Does resurfacing of asymptomatic full-thickness localized articular defects of the trochlea influence the outcome following unicompartmental knee arthroplasty of the medial compartment?: A retrospective cohort study with minimum seven-year follow-up | van Buul, GM; Headon, R; O'Toole, G; Neligan, M; O'Donnell, T | KNEE | | 2020 | 10.1016/j.knee.2020.07.088 | English |
| WOS:000593357400001 | Medical Interventions for Patellofemoral Pain and Patellofemoral Osteoarthritis: A Systematic Review | Macri, EM; Hart, HF; Thwaites, D; Barton, CJ; Crossley, KM; Bierma-Zeinstra, SMA; van Middelkoop, M | JOURNAL OF CLINICAL MEDICINE | | 2020 | 10.3390/jcm9113397 | English |
| WOS:000474380000026 | High patient satisfaction with significant improvement in knee function and pain relief after mid-term follow-up in patients with isolated patellofemoral inlay arthroplasty | Imhoff, AB; Feucht, MJ; Bartsch, E; Cotic, M; Pogorzelski, J | KNEE SURGERY SPORTS TRAUMATOLOGY ARTHROSCOPY | | 2019 | 10.1007/s00167-018-5173-2 | English |
| WOS:000564203800002 | Reliable improvements in participation in low-impact sports following implantation of a patellofemoral inlay arthroplasty at mid-term follow-up | Pogorzelski, J; Rupp, MC; Ketzer, C; Cotic, M; Lutz, P; Beeck, S; Imhoff, AB; Feucht, MJ | KNEE SURGERY SPORTS TRAUMATOLOGY ARTHROSCOPY | | 2021 | 10.1007/s00167-020-06245-5 | English |
| WOS:000446084200024 | Estimating an Individual's Probability of Revision Surgery After Knee Replacement: A Comparison of Modeling Approaches Using a National Data Set | Aram, P; Trela-Larsen, L; Sayers, A; Hills, AF; Blom, AW; McCloskey, EV; Kadirkamanathan, V; Wilkinson, JM | AMERICAN JOURNAL OF EPIDEMIOLOGY | | 2018 | 10.1093/aje/kwy121 | English |
| WOS:000339723100008 | In vivo kinematics of a robot-assisted uni- and multi-compartmental knee arthroplasty | Watanabe, T; Abbasi, AZ; Conditt, MA; Christopher, J; Kreuzer, S; Otto, JK; Banks, SA | JOURNAL OF ORTHOPAEDIC SCIENCE | | 2014 | 10.1007/s00776-014-0578-3 | English |
| WOS:000457503000002 | The clinical outcome of the different HemiCAP and UniCAP knee implants: A systematic and comprehensive review | Malahias, MA; Chytas, D; Thorey, F | ORTHOPEDIC REVIEWS | | 2018 | 10.4081/or.2018.7531 | English |
| WOS:000505643000003 | Replacement for patellofemoral arthritis | Johnson, DS; Turner, PG | KNEE | | 2019 | 10.1016/j.knee.2019.10.016 | English |
| WOS:000244491200008 | Functional outcomes after different types of knee arthroplasty: Kneeling ability versus descending stairs | Hassaballa, MA; Porteous, AJ; Learmonth, ID | MEDICAL SCIENCE MONITOR | | 2007 |  | English |
| WOS:000320660500011 | Results of a French multicentre retrospective experience with four hundred and eighteen failed unicondylar knee arthroplasties | Saragaglia, D; Bonnin, M; Dejour, D; Deschamps, G; Chol, C; Chabert, B; Refaie, R | INTERNATIONAL ORTHOPAEDICS | | 2013 | 10.1007/s00264-013-1915-4 | English |
| WOS:000496697200012 | The patient results and satisfaction of knee arthroplasty in a validated grading system | Oosthuizen, CR; Van Der Straeten, C; Maposa, I; Snyckers, CH; Vermaak, DP; Magobotha, S | INTERNATIONAL ORTHOPAEDICS | | 2019 | 10.1007/s00264-019-04412-z | English |
| WOS:000404659400004 | The influence of malalignment and ageing following sterilisation by gamma irradiation in an inert atmosphere on the wear of ultra-high-molecular-weight polyethylene in patellofemoral replacements | Maiti, R; Cowie, RM; Fisher, J; Jennings, LM | PROCEEDINGS OF THE INSTITUTION OF MECHANICAL ENGINEERS PART H-JOURNAL OF ENGINEERING IN MEDICINE | | 2017 | 10.1177/0954411917696112 | English |
| WOS:000512494900001 | Prospective Outcomes of Cryopreserved Osteochondral Allograft for Patellofemoral Cartilage Defects at Minimum 2-Year Follow-up | Melugin, HP; Ridley, TJ; Bernard, CD; Wischmeier, D; Farr, J; Stuart, MJ; Macalena, JA; Krych, AJ | CARTILAGE | | 2021 | 10.1177/1947603520903420 | English |
| WOS:000371658300013 | Osteochondral Allograft Transplantation of the Femoral Trochlea | Cameron, JI; Pulido, PA; McCauley, JC; Bugbee, WD | AMERICAN JOURNAL OF SPORTS MEDICINE | | 2016 | 10.1177/0363546515620193 | English |
| WOS:000505034800015 | Mid-term survivorship and patient-reported outcomes of robotic-arm assisted partial knee arthroplasty A SINGLE-SURGEON STUDY OF 1,018 KNEES | Burger, JA; Kleeblad, LJ; Laas, N; Pearle, AD | BONE & JOINT JOURNAL | | 2020 | 10.1302/0301-620X.102B1.BJJ-2019-0510.R1 | English |
| WOS:000418970700030 | Patellofemoral arthritis treated with resurfacing implant: Clinical outcome and complications at a minimum two-year follow-up | Zicaro, JP; Yacuzzi, C; Bonorino, JA; Carbo, L; Costa-Paz, M | KNEE | | 2017 | 10.1016/j.knee.2017.09.003 | English |
| WOS:000408732200007 | Cost effectiveness of patellofemoral versus total knee arthroplasty in younger patients | Chawla, H; Nwachukwu, BU; Van der List, JP; Eggman, AA; Pearle, AD; Ghomrawi, HM | BONE & JOINT JOURNAL | | 2017 | 10.1302/0301-620X.99B8.BJJ-2016-1032.R1 | English |
| WOS:000673173100002 | Contemporary knee arthroplasty: one fits all or time for diversity? | Beckmann, J; Meier, MK; Benignus, C; Hecker, A; Thienpont, E | ARCHIVES OF ORTHOPAEDIC AND TRAUMA SURGERY | | 2021 | 10.1007/s00402-021-04042-4 | English |
| WOS:000683527500004 | Augmented reality in robotic assisted orthopaedic surgery: A pilot study | Iqbal, H; Tatti, F; Baena, FRY | JOURNAL OF BIOMEDICAL INFORMATICS | | 2021 | 10.1016/j.jbi.2021.103841 | English |
| WOS:000653083500008 | Recurrent patellar dislocations with patellar cartilage defects: A pain in the knee? | Dalal, S; Setia, P; Debnath, A; Guro, R; Kotwal, R; Chandratreya, A | KNEE | | 2021 | 10.1016/j.knee.2021.01.019 | English |
| WOS:000071533400004 | Patellofemoral arthroplasty (PFA) for patellofemoral arthritis: a retrospective study of 50 implants | Mertl, P; Van, FT; Bonhomme, P; Vives, P | REVUE DE CHIRURGIE ORTHOPEDIQUE ET REPARATRICE DE L APPAREIL MOTEUR | | 1997 |  | French |
| WOS:000323155300020 | Advanced Patellofemoral Cartilage Lesions in Patients Younger Than 50 Years of Age: Is There an Ideal Operative Option? | Noyes, FR; Barber-Westin, SD | ARTHROSCOPY-THE JOURNAL OF ARTHROSCOPIC AND RELATED SURGERY | | 2013 | 10.1016/j.arthro.2013.03.077 | English |
| WOS:000928090000001 | Short term clinical outcomes of a Prochondrix (R) thin laser-etched osteochondral allograft for the treatment of articular cartilage defects in the knee | Mehta, VM; Mehta, S; Santoro, S; Shriver, R; Mandala, C; Weess, C | JOURNAL OF ORTHOPAEDIC SURGERY | | 2022 | 10.1177/10225536221141781 | English |
| WOS:000431409000019 | The Mark Coventry Award: Patellofemoral Arthroplasty Results in Better Range of Movement and Early Patient-reported Outcomes Than TKA | Odgaard, A; Madsen, F; Kristensen, PW; Kappel, A; Fabrin, J | CLINICAL ORTHOPAEDICS AND RELATED RESEARCH | | 2018 | 10.1007/s11999.0000000000000017 | English |
| WOS:000265366600010 | Manipulation under anaesthesia for stiffness following knee arthroplasty | Mohammed, R; Syed, S; Ahmed, N | ANNALS OF THE ROYAL COLLEGE OF SURGEONS OF ENGLAND | | 2009 | 10.1308/003588409X359321 | English |
| WOS:000578467800001 | Comparing Return to Sports After Patellofemoral and Knee Arthroplasty in an Age- and Sex-Matched Cohort | Schneider, BL; Ling, DI; Kleebad, LJ; Strickland, S; Pearle, A | ORTHOPAEDIC JOURNAL OF SPORTS MEDICINE | | 2020 | 10.1177/2325967120957425 | English |
| WOS:000809615400009 | Staged BiCompartmental Knee Arthroplasty has Greater Functional Improvement, but Equivalent Midterm Survivorship, as Revision TKA for Progressive Osteoarthritis After Partial Knee Arthroplasty | Haffar, A; Krueger, CA; Marullo, M; Banerjee, S; Dobelle, E; Argenson, JN; Sprenzel, JF; Berger, RA; Romagnoli, S; Lonner, JH | JOURNAL OF ARTHROPLASTY | | 2022 | 10.1016/j.arth.2022.02.084 | English |
| WOS:000539904900001 | Clinical outcome of bi-unicompartmental knee arthroplasty for both medial and lateral femorotibial arthritis: a systematic review-is there proof of concept? | Wada, K; Price, A; Gromov, K; Lustig, S; Troelsen, A | ARCHIVES OF ORTHOPAEDIC AND TRAUMA SURGERY | | 2020 | 10.1007/s00402-020-03492-6 | English |
| WOS:000387286500017 | Navigated small implants in knee reconstruction | Confalonieri, N; Biazzo, A; Cerveri, P; Pullen, C; Manzotti, A | KNEE SURGERY SPORTS TRAUMATOLOGY ARTHROSCOPY | | 2016 | 10.1007/s00167-016-4324-6 | English |
| WOS:000636162400001 | The lack of retropatellar resurfacing at index surgery is significantly associated with failure in patients following patellofemoral inlay arthroplasty: a multi-center study of more than 260 patients | Imhoff, AB; Bartsch, E; Becher, C; Behrens, P; Bode, G; Cotic, M; Diermeier, T; Falk, H; Feucht, MJ; Haupt, U; Hinterwimmer, S; Holz, J; Hutter, R; Kaiser, R; Knoblauch, T; Nebelung, W; Niemeyer, P; O'Donnel, T; Pagenstert, G; Patzer, T; Rose, T; Rupp, MC; Tischer, T; Venjakob, AJ; Vogt, S; Pogorzelski, J | KNEE SURGERY SPORTS TRAUMATOLOGY ARTHROSCOPY | | 2022 | 10.1007/s00167-021-06544-5 | English |
| WOS:000561872100012 | Patterns of cartilage loss and anterior cruciate ligament status in end-stage osteoarthritis of the knee ASSESSING SUITABILITY FOR PARTIAL KNEE ARTHROPLASTY | Scott, CEH; Holland, G; Krahelski, O; Murray, IR; Keating, JF; Keenan, OJF | BONE & JOINT JOURNAL | | 2020 | 10.1302/0301-620X.102B6.BJJ-2019-1434.R1 | English |
| WOS:000428796600031 | A stand-alone lateral condyle-elevating trochlear osteotomy leads to high residual instability but no excessive increase in patellofemoral osteoarthritis at 12-year follow-up | Tigchelaar, S; van Sambeeck, J; Koeter, S; van Kampen, A | KNEE SURGERY SPORTS TRAUMATOLOGY ARTHROSCOPY | | 2018 | 10.1007/s00167-017-4602-y | English |
| WOS:000460315200039 | Derotational osteotomy at the distal femur is effective to treat patients with patellar instability | Imhoff, FB; Cotic, M; Liska, F; Dyrna, FGE; Beitzel, K; Imhoff, AB; Herbst, E | KNEE SURGERY SPORTS TRAUMATOLOGY ARTHROSCOPY | | 2019 | 10.1007/s00167-018-5212-z | English |
| WOS:000927923100001 | Effectiveness of an Accelerated Rehabilitation Protocol After Tibial Tubercle Osteotomy | Morgan, C; Bell, RM; Burland, JP; Kriscenski, D; Ilinski, A; Cote, MP; Edgar, CM | ORTHOPAEDIC JOURNAL OF SPORTS MEDICINE | | 2022 | 10.1177/23259671221133105 | English |
| WOS:000361242000006 | Is knee function better with contemporary modular bicompartmental arthroplasty compared to total knee arthroplasty? Short-term outcomes of a prospective matched study including 68 cases | Parratte, S; Ollivier, M; Opsomer, G; Lunebourg, A; Argenson, JN; Thienpont, E | ORTHOPAEDICS & TRAUMATOLOGY-SURGERY & RESEARCH | | 2015 | 10.1016/j.otsr.2015.03.019 | English |
| WOS:000333686100024 | Autologous Chondrocyte Implantation and Anteromedialization for Isolated Patellar Articular Cartilage Lesions 5- to 11-Year Follow-up | Gillogly, SD; Arnold, RM | AMERICAN JOURNAL OF SPORTS MEDICINE | | 2014 | 10.1177/0363546513519077 | English |
| WOS:000597215000001 | Has the modern design of Attune total knee replacement improved outcome in patients with isolated patellofemoral arthritis? | Ashraf, Y; Iqbal, HJ; Senevirathana, S; Ashraf, T | JOURNAL OF ORTHOPAEDIC SURGERY | | 2020 | 10.1177/2309499020975553 | English |
| WOS:000341973100016 | Densitometric kneecap changes after unilateral knee arthroplasty | Barchetti, F; Stagnitti, A; Al Ansari, N; De Marco, V; Montechiarello, S; Forte, V; Noce, V; Glorioso, M; Sorrentino, V; Barchetti, G; Pasqualitto, E; Marini, A; Marini, M | EUROPEAN REVIEW FOR MEDICAL AND PHARMACOLOGICAL SCIENCES | | 2014 |  | English |
| WOS:000841548200016 | Patellofemoral Arthroplasty Results in Better Time-weighted Patient-reported Outcomes After 6 Years than TKA: A Randomized Controlled Trial | Odgaard, A; Kappel, A; Madsen, F; Kristensen, PW; Stephensen, S; Attarzadeh, AP | CLINICAL ORTHOPAEDICS AND RELATED RESEARCH | | 2022 | 10.1097/CORR.0000000000002178 | English |
| WOS:000422744800003 | Does a simple syringe applicator enhance bone cement set up time in knee arthroplasty? | Sodhi, N; Dalton, SE; Khlopas, A; Sultan, AA; Curtis, GL; Harb, MA; Naziri, Q; Newman, JM; Barrington, JW; Mont, MA | ANNALS OF TRANSLATIONAL MEDICINE | | 2017 | 10.21037/atm.2017.11.17 | English |
| WOS:000346902900042 | Are Revisions of Patellofemoral Arthroplasties More Like Primary or Revision TKAs | Parratte, S; Lunebourg, A; Ollivier, M; Abdel, MP; Argenson, JN | CLINICAL ORTHOPAEDICS AND RELATED RESEARCH | | 2015 | 10.1007/s11999-014-3756-x | English |
| WOS:000509666800023 | The Dubousset Functional Test is a Novel Assessment of Physical Function and Balance | Diebo, BG; Challier, V; Shah, NV; Kim, D; Murray, DP; Kelly, JJ; Lafage, R; Paulino, CB; Passias, PG; Schwab, FJ; Lafage, V | CLINICAL ORTHOPAEDICS AND RELATED RESEARCH | | 2019 | 10.1097/CORR.0000000000000820 | English |
| WOS:000472611200028 | What Is the Risk of Repeat Revision When Patellofemoral Replacement Is Revised to TKA? An Analysis of 482 Cases From a Large National Arthroplasty Registry | Lewis, PL; Graves, SE; Cuthbert, A; Parker, D; Myers, P | CLINICAL ORTHOPAEDICS AND RELATED RESEARCH | | 2019 | 10.1097/CORR.0000000000000541 | English |
| WOS:000870427400003 | COMBINED MPFL RECONSTRUCTION WITH TIBIAL TUBERCLE OSTEOTOMY AND REPAIR OF PATELLAR CARTILAGE DEFECT WITH PARTICULATED JUVENILE ARTICULAR CARTILAGE | Dennis, ER; Marmor, WA; Stein, BES | JBJS ESSENTIAL SURGICAL TECHNIQUES | | 2022 | 10.2106/JBJS.ST.21.00013 | English |

| **Table S2 Detailed bibliographies searched from Pubmed in the field of patellofemoral arthroplasty** | | | | | | |
| --- | --- | --- | --- | --- | --- | --- |
| **PMID** | **Article Title** | **Authors** | **Journal** | **Publication Year** | **DOI** | **Language** |
| 27330013 | Knee osteoarthritis: a review of management options | Hussain SM, Neilly DW, Baliga S, Patil S, Meek R. | Scott Med J | 2016 | 10.1177/0036933015619588 | English |
| 22928430 | The basic science of the patella: structure, composition, and function | Fox AJ, Wanivenhaus F, Rodeo SA. | J Knee Surg | 2012 | 10.1055/s-0032-1313741 | English |
| 31579533 | Patellofemoral Arthroplasty | Odgaard A, Eldridge J, Madsen F. | JBJS Essent Surg Tech | 2019 | 10.2106/JBJS.ST.18.00094 | English |
| 24412044 | Patellofemoral arthroplasty | Lustig S. | Orthop Traumatol Surg Res | 2014 | 10.1016/j.otsr.2013.06.013 | English |
| 25150332 | Isolated patellofemoral arthroplasty | Oni JK, Hochfelder J, Dayan A. | Bull Hosp Jt Dis (2013) | 2014 |  | English |
| 30275797 | Patellofemoral Arthroplasty | Godshaw B, Kolodychuk N, Williams GK Jr, Browning B, Jones D. | Ochsner J | 2018 | 10.31486/toj.18.0009 | English |
| 17664369 | Patellofemoral arthroplasty | Lonner JH. | J Am Acad Orthop Surg | 2007 | 10.5435/00124635-200708000-00006 | English |
| 20415369 | Patellofemoral arthroplasty | Lonner JH. | Instr Course Lect | 2010 |  | English |
| 20839682 | Patellofemoral arthroplasty | Lonner JH. | Orthopedics | 2010 | 10.3928/01477447-20100722-39 | English |
| 29628679 | Patellofemoral arthroplasty: Current concepts | Cuthbert R, Tibrewal S, Tibrewal SB. | J Clin Orthop Trauma | 2018 | 10.1016/j.jcot.2017.11.006 | English |
| 29721690 | Advances in Patellofemoral Arthroplasty | Strickland SM, Bird ML, Christ AB. | Curr Rev Musculoskelet Med | 2018 | 10.1007/s12178-018-9477-0 | English |
| 33214935 | Erratum: Patellofemoral Arthroplasty |  | JBJS Essent Surg Tech | 2020 | 10.2106/JBJS.ST.ER.18.00094 | English |
| 35244809 | Patellofemoral arthroplasty: expert opinion | Hoogervorst P, Arendt EA. | J Exp Orthop | 2022 | 10.1186/s40634-022-00457-z | English |
| 28138717 | [Patellofemoral arthroplasty] | Cotic M, Forkel P, Imhoff AB. | Oper Orthop Traumatol | 2017 | 10.1007/s00064-016-0477-1 | German |
| 31699493 | Replacement for patellofemoral arthritis | Johnson DS, Turner PG. | Knee | 2019 | 10.1016/j.knee.2019.10.016 | English |
| 18722775 | Optimizing patellofemoral arthroplasty | Farr J 2nd, Barrett D. | Knee | 2008 | 10.1016/j.knee.2008.05.008 | English |
| 25382369 | Patellofemoral joint replacement, an evolving concept | Borus T, Brilhault J, Confalonieri N, Johnson D, Thienpont E. | Knee | 2014 | 10.1016/S0968-0160(14)50010-5 | English |
| 535258 | Patellofemoral replacement | Blazina ME, Fox JM, Del Pizzo W, Broukhim B, Ivey FM. | Clin Orthop Relat Res | 1979 |  | English |
| 30635231 | Surgical technique in patellofemoral arthroplasty | Remy F. | Orthop Traumatol Surg Res | 2019 | 10.1016/j.otsr.2018.05.020 | English |
| 33355685 | [Patellofemoral inlay implants-an innovation in patellofemoral joint arthroplasty?] | Degenhardt H, Imhoff AB, Feucht MJ, Pogorzelski J. | Orthopade | 2021 | 10.1007/s00132-020-04059-4 | German |
| 20175647 | Isolated patellofemoral osteoarthritis | van Jonbergen HP, Poolman RW, van Kampen A. | Acta Orthop | 2010 | 10.3109/17453671003628756 | English |
| 30193976 | Patellofemoral joint replacement - Mean five year follow-up | Ajnin S, Buchanan D, Arbuthnot J, Fernandes R. | Knee | 2018 | 10.1016/j.knee.2018.08.014 | English |
| 22878660 | Complications in patellofemoral surgery | Tompkins M, Arendt EA. | Sports Med Arthrosc Rev | 2012 | 10.1097/JSA.0b013e31825c74cf | English |
| 27891054 | Patellofemoral Joint Arthroplasty: Our Experience in Isolated Patellofemoral and Bicompartmental Arthritic Knees | Sabatini L, Schirò M, Atzori F, Ferrero G, Massè A. | Clin Med Insights Arthritis Musculoskelet Disord | 2016 | 10.4137/CMAMD.S40498 | English |
| 33749150 | Joint Awareness after Patellofemoral Arthroplasty Evaluated with the Forgotten Joint Score: A Comparison Study | Lin W, Dai Y, Dong C, Piao K, Hao K, Wang F. | Orthop Surg | 2021 | 10.1111/os.12921 | English |
| 24993415 | Patellofemoral arthroplasty in the athlete | Farr J, Arendt E, Dahm D, Daynes J. | Clin Sports Med | 2014 | 10.1016/j.csm.2014.03.003 | English |
| 36507211 | Robotic Assisted Patellofemoral Joint Replacement: Surgical Technique, Tips and Tricks | Selvaratnam V, Toms AD, Mandalia VI. | Indian J Orthop | 2022 | 10.1007/s43465-022-00746-w | English |
| 29551276 | Femoral component rotation in patellofemoral joint replacement | van Jonbergen HW, Westerbeek RE. | Knee | 2018 | 10.1016/j.knee.2018.02.007 | English |
| 32766389 | The Present Situation of Patellofemoral Arthroplasty in the Management of Solitary Patellofemoral Osteoarthritis | Rodriguez-Merchan EC. | Arch Bone Jt Surg | 2020 | 10.22038/ABJS.2019.14125 | English |
| 31495529 | Isolated Patellofemoral Joint Arthroplasty: Can Preoperative Bone Scans Predict Survivorship? | Baker JF, Caborn DN, Schlierf TJ, Fain TB, Smith LS, Malkani AL. | J Arthroplasty | 2020 | 10.1016/j.arth.2019.08.021 | English |
| 30315337 | Patellar tendon shortening following patellofemoral joint replacement | van Engen LAH, Landman EBM, Kleinlugtenbelt YV, van Jonbergen HW. | Int Orthop | 2019 | 10.1007/s00264-018-4194-2 | English |
| 36574834 | Midterm outcomes of patellofemoral arthroplasty | Martínez-Sañudo B, Fornell S, Vallejo M, Domecq G. | Rev Esp Cir Ortop Traumatol | 2023 | 10.1016/j.recot.2022.12.016 | English |
| 33438917 | Cartilage Disease of the Patellofemoral Joint: Realignment, Restoration, Replacement | Otlans P, Lattermann C, Sherman SL, Gomoll AH, Lonner JH, Freedman KB. | Instr Course Lect | 2021 |  | English |
| 33825970 | Midterm results of modern patellofemoral arthroplasty versus total knee arthroplasty for isolated patellofemoral arthritis: systematic review and meta-analysis of comparative studies | Elbardesy H, McLeod A, Gul R, Harty J. | Arch Orthop Trauma Surg | 2022 | 10.1007/s00402-021-03882-4 | English |
| 33532210 | Patellofemoral Replacement With Tibial Tubercle Osteotomy | Arvesen JE, Wyland DJ. | Arthrosc Tech | 2021 | 10.1016/j.eats.2020.09.009 | English |
| 15995424 | Alternatives to patellofemoral arthroplasty | Fulkerson JP. | Clin Orthop Relat Res | 2005 | 10.1097/01.blo.0000172305.20156.ba | English |
| 22407183 | Patellofemoral arthroplasty, where are we today? | Lustig S, Magnussen RA, Dahm DL, Parker D. | Knee Surg Sports Traumatol Arthrosc | 2012 | 10.1007/s00167-012-1948-z | English |
| 36519003 | A Newly Identified Complication of Patellofemoral Arthroplasty: Case Report and Literature Review | Solarino G, Maccagnano G, Vicenti G, Buono C, Simone F, Ottaviani G, Zavattini G, Zaccari D, Carrozzo M, Spinarelli A, Bizzoca D, Moretti B. | Geriatr Orthop Surg Rehabil | 2022 | 10.1177/21514593221138662 | English |
| 25382368 | Partial knee arthroplasty: patellofemoral arthroplasty and combined unicompartmental and patellofemoral arthroplasty implants--general considerations and indications, technique and clinical experience | Benazzo F, Rossi SM, Ghiara M. | Knee | 2014 | 10.1016/S0968-0160(14)50009-9 | English |
| 32368460 | Minimally Invasive Robotic-Assisted Patellofemoral Arthroplasty | Hassebrock JD, Makovicka JL, Wong M, Patel KA, Scott KL, Deckey DG, Chhabra A. | Arthrosc Tech | 2020 | 10.1016/j.eats.2019.11.013 | English |
| 22992881 | Patellofemoral arthroplasty: the other unicompartmental knee replacement | Walker T, Perkinson B, Mihalko WM. | J Bone Joint Surg Am | 2012 | 10.2106/JBJS.L.00539 | English |
| 25381467 | Isolated patellofemoral arthroplasty reproduces natural patellofemoral joint kinematics when the patella is resurfaced | Vandenneucker H, Labey L, Vander Sloten J, Desloovere K, Bellemans J. | Knee Surg Sports Traumatol Arthrosc | 2016 | 10.1007/s00167-014-3415-5 | English |
| 28735802 | Correction of Patellofemoral Malalignment With Patellofemoral Arthroplasty | Valoroso M, Saffarini M, La Barbera G, Toanen C, Hannink G, Nover L, Dejour DH. | J Arthroplasty | 2017 | 10.1016/j.arth.2017.06.048 | English |
| 32444231 | Patellofemoral Arthroplasty Surgical Technique: Lateral or Medial Parapatellar Approach | Jeong SH, Schneider B, Pyne AS, Tishelman JC, Strickland SM. | J Arthroplasty | 2020 | 10.1016/j.arth.2020.04.026 | English |
| 25382370 | Coronal alignment of patellofemoral arthroplasty | Thienpont E, Lonner JH. | Knee | 2014 | 10.1016/S0968-0160(14)50011-7 | English |
| 15483797 | Early results with a total patellofemoral joint replacement arthroplasty prosthesis | Merchant AC. | J Arthroplasty | 2004 | 10.1016/j.arth.2004.03.011 | English |
| 23203512 | Outcomes of total knee replacement after patellofemoral arthroplasty | Hutt J, Dodd M, Bourke H, Bell J. | J Knee Surg | 2013 | 10.1055/s-0032-1329233 | English |
| 29270562 | Patellofemoral Arthroplasty: Current Concepts and Review of the Literature | Pisanu G, Rosso F, Bertolo C, Dettoni F, Blonna D, Bonasia DE, Rossi R. | Joints | 2017 | 10.1055/s-0037-1606618 | English |
| 32869235 | Patellofemoral Arthroplasty Improves Patellofemoral Alignment in Patients with Patellofemoral Osteoarthritis with Trochlear Dysplasia | Yang G, Wang J, Dai Y, Lin W, Niu J, Wang F. | J Knee Surg | 2022 | 10.1055/s-0040-1715099 | English |
| 15995413 | Patellofemoral replacement. 1979 | Blazina ME, Fox JM, Del Pizzo W, Broukhim B, Ivey FM. | Clin Orthop Relat Res | 2005 |  | English |
| 23827831 | The clinical outcome of patellofemoral arthroplasty | Lonner JH, Bloomfield MR. | Orthop Clin North Am | 2013 | 10.1016/j.ocl.2013.03.002 | English |
| 27131405 | The Journey patellofemoral joint arthroplasty: A minimum 5year follow-up study | Ahearn N, Metcalfe AJ, Hassaballa MA, Porteous AJ, Robinson JR, Murray JR, Newman JH. | Knee | 2016 | 10.1016/j.knee.2016.03.004 | English |
| 18602565 | Patellofemoral arthroplasty in the treatment of patellofemoral arthritis: rationale and outcomes in younger patients | Leadbetter WB. | Orthop Clin North Am | 2008 | 10.1016/j.ocl.2008.04.001 | English |
| 28594500 | Patellofemoral Arthroplasty: An Evolving Science | Lonner JH. | Instr Course Lect | 2017 |  | English |
| 34883522 | [When nothing else works: patellofemoral joint arthroplasty] | Benignus C, Meier M, Best R, Beckmann J. | Sportverletz Sportschaden | 2021 | 10.1055/a-1523-9937 | German |
| 26543657 | Patellofemoral Joint Replacement and Nickel Allergy: An Unusual Presentation | Syed F, Jenner E, Faisal M. | Case Rep Orthop | 2015 | 10.1155/2015/635082 | English |
| 36049772 | Effect of Patellofemoral Arthroplasty on Patellar Height in Patients with Patellofemoral Osteoarthritis | Lee H, Fletcher C, Hartwell M, Strickland SM. | J Knee Surg | 2023 | 10.1055/s-0042-1755354 | English |
| 26590562 | Survivorship and functional outcomes of patellofemoral arthroplasty: a systematic review | van der List JP, Chawla H, Zuiderbaan HA, Pearle AD. | Knee Surg Sports Traumatol Arthrosc | 2017 | 10.1007/s00167-015-3878-z | English |
| 18602563 | Patellofemoral arthroplasty: the impact of design on outcomes | Lonner JH. | Orthop Clin North Am | 2008 | 10.1016/j.ocl.2008.02.002 | English |
| 18602566 | Results of total knee replacement for isolated patellofemoral arthritis: when not to perform a patellofemoral arthroplasty | Delanois RE, McGrath MS, Ulrich SD, Marker DR, Seyler TM, Bonutti PM, Mont MA. | Orthop Clin North Am | 2008 | 10.1016/j.ocl.2008.03.003 | English |
| 24417902 | Association and impact of patellofemoral dysplasia on patellofemoral arthropathy and arthroplasty | Mofidi A, Veravalli K, Jinnah RH, Poehling GG. | Knee | 2014 | 10.1016/j.knee.2013.09.009 | English |
| 35282735 | Patellar button compatibility in the conversion of Patellofemoral Arthroplasty to a Total Knee Arthroplasty: A review of the contemporary literature | McDonald LK, Kurmis AP. | J Orthop Surg (Hong Kong) | 2022 | 10.1177/10225536221084147 | English |
| 28793978 | Patellofemoral arthroplasty conversion to total knee arthroplasty: Retrieval analysis and clinical correlation | Christ AB, Baral E, Koch C, Shubin Stein BE, Gonzalez Della Valle A, Strickland SM. | Knee | 2017 | 10.1016/j.knee.2017.06.015 | English |
| 25861405 | Femoral component rotation in patellofemoral joint replacement: a study protocol for a prospective observational study | Westerbeek RE, Derks RP, Zonneveld BJ, van Jonbergen HP. | Open Orthop J | 2015 | 10.2174/1874325001509010045 | English |
| 28861196 | Patellofemoral Joint Arthroplasty: Early Results and Functional Outcome of the Zimmer Gender Solutions Patello-Femoral Joint System | Osarumwense D, Syed F, Nzeako O, Akilapa S, Zubair O, Waite J. | Clin Orthop Surg | 2017 | 10.4055/cios.2017.9.3.295 | English |
| 24579096 | Mid-term results of the FPV patellofemoral joint replacement | Al-Hadithy N, Patel R, Navadgi B, Deo S, Hollinghurst D, Satish V. | Knee | 2014 | 10.1016/j.knee.2013.08.010 | English |
| 32115918 | [Patellofemoral arthroplasty for the treatment of patellofemoral arthritis] | Gao HL, Xiao LB, Zhang Q, Zhang JC, He Y, Zhai WT, Shi Q. | Zhongguo Gu Shang | 2020 | 10.3969/j.issn.1003-0034.2020.01.003 | Chinese |
| 30103596 | [Progress on treatment for patellofemoral arthritis] | Zhang ZX, Xu F, Yang XL. | Zhongguo Gu Shang | 2018 | 10.3969/j.issn.1003-0034.2018.07.019 | Chinese |
| 33125548 | Preoperative patellofemoral anatomy affects failure rate after isolated patellofemoral inlay arthroplasty | Feucht MJ, Lutz PM, Ketzer C, Rupp MC, Cotic M, Imhoff AB, Pogorzelski J. | Arch Orthop Trauma Surg | 2020 | 10.1007/s00402-020-03651-9 | English |
| 21162949 | A preliminary report of patellofemoral arthroplasty in isolated patellofemoral arthritis | Gao X, Xu ZJ, He RX, Yan SG, Wu LD. | Chin Med J (Engl) | 2010 |  | English |
| 33858458 | Patellofemoral arthroplasty versus total knee arthroplasty for isolated patellofemoral osteoarthritis: a systematic review and meta-analysis | Peng G, Liu M, Guan Z, Hou Y, Liu Q, Sun X, Zhu X, Feng W, Zeng J, Zhong Z, Zeng Y. | J Orthop Surg Res | 2021 | 10.1186/s13018-021-02414-5 | English |
| 21886926 | Medium term results of Avon patellofemoral joint replacement | Sarda PK, Shetty A, Maheswaran SS. | Indian J Orthop | 2011 | 10.4103/0019-5413.83761 | English |
| 18602564 | Patellofemoral arthroplasty with a customized trochlear prosthesis | Sisto DJ, Sarin VK. | Orthop Clin North Am | 2008 | 10.1016/j.ocl.2008.03.002 | English |
| 25382365 | Biomechanics of medial unicondylar in combination with patellofemoral knee arthroplasty | Heyse TJ, El-Zayat BF, De Corte R, Scheys L, Chevalier Y, Fuchs-Winkelmann S, Labey L. | Knee | 2014 | 10.1016/S0968-0160(14)50002-6 | English |
| 19491912 | Surgical options for patients with osteoarthritis of the knee | Lützner J, Kasten P, Günther KP, Kirschner S. | Nat Rev Rheumatol | 2009 | 10.1038/nrrheum.2009.88 | English |
| 25154928 | [Patellofemoral arthroplasty: indication, technique and results] | Cotic M, Imhoff AB. | Orthopade | 2014 | 10.1007/s00132-014-3006-7 | German |
| 30601045 | Patellofemoral arthroplasty versus total knee arthroplasty for patients with patellofemoral osteoarthritis: equal function and satisfaction but higher revision rate for partial arthroplasty at a minimum eight years’ follow-up | Clement ND, Howard TA, Immelman RJ, MacDonald D, Patton JT, Lawson GM, Burnett R. | Bone Joint J | 2019 | 10.1302/0301-620X.101B1.BJJ-2018-0654.R2 | English |
| 31362558 | Classification of combined partial knee arthroplasty | Garner A, van Arkel RJ, Cobb J. | Bone Joint J | 2019 | 10.1302/0301-620X.101B8.BJJ-2019-0125.R1 | English |
| 26955615 | Are "Patellofemoral Symptoms" Truly Related to the Patellofemoral Joint? | Yassa R, Khalfaoui MY, Davies AP. | Knee Surg Relat Res | 2016 | 10.5792/ksrr.2016.28.1.68 | English |
| 23395041 | Patellofemoral arthroplasty: the other unicompartmental knee replacement | Walker T, Perkinson B, Mihalko WM. | Instr Course Lect | 2013 |  | English |
| 27825938 | Why do patellofemoral arthroplasties fail today? A systematic review | van der List JP, Chawla H, Villa JC, Pearle AD. | Knee | 2017 | 10.1016/j.knee.2015.11.002 | English |
| 23102421 | Blood loss and transfusion rates following patellofemoral arthroplasty | Courtney J, Liebelt D, Nett MP, Cushner FD. | Orthop Clin North Am | 2012 | 10.1016/j.ocl.2012.07.007 | English |
| 23790605 | Clinical results of patellofemoral arthroplasty | Morris MJ, Lombardi AV Jr, Berend KR, Hurst JM, Adams JB. | J Arthroplasty | 2013 | 10.1016/j.arth.2013.05.012 | English |
| 33925287 | Treatment of Unicompartmental Cartilage Defects of the Knee with Unicompartmental Knee Arthroplasty, Patellofemoral Partial Knee Arthroplasty or Focal Resurfacing | Springer B, Boettner F. | Life (Basel) | 2021 | 10.3390/life11050394 | English |
| 19340381 | Modular bicompartmental knee arthroplasty with robotic arm assistance | Lonner JH. | Am J Orthop (Belle Mead NJ) | 2009 |  | English |
| 32114806 | The PAT randomized clinical trial | Joseph MN, Achten J, Parsons NR, Costa ML; PAT Trial Collaborators. | Bone Joint J | 2020 | 10.1302/0301-620X.102B3.BJJ-2019-0723.R1 | English |
| 15995422 | Outcome instruments for patellofemoral arthroplasty | Paxton EW, Fithian DC. | Clin Orthop Relat Res | 2005 | 10.1097/01.blo.0000171544.38095.77 | English |
| 24482626 | Surgical treatment of isolated patellofemoral osteoarthritis | Rodriguez-Merchan EC. | HSS J | 2014 | 10.1007/s11420-013-9375-3 | English |
| 15995426 | The appropriate use of patellofemoral arthroplasty: an analysis of reported indications, contraindications, and failures | Leadbetter WB, Ragland PS, Mont MA. | Clin Orthop Relat Res | 2005 |  | English |
| 33438913 | Partial Knee Arthroplasty: The State of the Art | Fuller BC, Lonner JH, Berend KR, Berger RA, Gerlinger TL. | Instr Course Lect | 2021 |  | English |
| 15534537 | Patellofemoral arthroplasty: pros, cons, and design considerations | Lonner JH. | Clin Orthop Relat Res | 2004 |  | English |
| 33795175 | Onlay Patellofemoral Arthroplasty in Patients With Isolated Patellofemoral Arthritis: A Systematic Review | Villa JC, Paoli AR, Nelson-Williams HW, Badr RN, Harper KD. | J Arthroplasty | 2021 | 10.1016/j.arth.2021.02.054 | English |
| 27979409 | Outcomes of Patellofemoral Arthroplasty Based on Radiographic Severity | deDeugd CM, Pareek A, Krych AJ, Cummings NM, Dahm DL. | J Arthroplasty | 2017 | 10.1016/j.arth.2016.11.006 | English |
| 24187369 | Patellofemoral replacement: the third compartment | Hofmann AA, McCandless JB, Shaeffer JF, Magee TH. | Bone Joint J | 2013 | 10.1302/0301-620X.95B11.32985 | English |
| 31500912 | The Clinical Outcome of Patellofemoral Arthroplasty vs Total Knee Arthroplasty in Patients Younger Than 55 Years | Kamikovski I, Dobransky J, Dervin GF. | J Arthroplasty | 2019 | 10.1016/j.arth.2019.07.016 | English |
| 29475782 | Mid-term survivorship and clinical outcomes of the Avon patellofemoral joint replacement | Middleton SWF, Toms AD, Schranz PJ, Mandalia VI. | Knee | 2018 | 10.1016/j.knee.2018.01.007 | English |
| 19223947 | Patellofemoral resurfacing arthroplasty: literature review and description of a novel technique | Cannon A, Stolley M, Wolf B, Amendola A. | Iowa Orthop J | 2008 |  | English |
| 20056375 | Long-term outcomes of patellofemoral arthroplasty | van Jonbergen HP, Werkman DM, Barnaart LF, van Kampen A. | J Arthroplasty | 2010 | 10.1016/j.arth.2009.08.023 | English |
| 26217462 | The Avon Patellofemoral Joint Replacement: Mid-Term Prospective Results from an Independent Centre | Akhbari P, Malak T, Dawson-Bowling S, East D, Miles K, Butler-Manuel PA. | Clin Orthop Surg | 2015 | 10.4055/cios.2015.7.2.171 | English |
| 19085728 | [Patellofemoral arthroplasty--results of a nation-wide survey in Germany and review of the literature] | Becher C, Renke A, Heyse TJ, Schofer M, Tibesku CO, Fuchs-Winkelmann S. | Z Orthop Unfall | 2008 | 10.1055/s-2008-1039000 | German |
| 29129614 | Mid-Term Clinical, Functional, and Radiographic Outcomes of 105 Gender-Specific Patellofemoral Arthroplasties, With or Without the Association of Medial Unicompartmental Knee Arthroplasty | Romagnoli S, Marullo M. | J Arthroplasty | 2018 | 10.1016/j.arth.2017.10.019 | English |
| 17079388 | Revision of a failed patellofemoral arthroplasty to a total knee arthroplasty | Lonner JH, Jasko JG, Booth RE Jr. | J Bone Joint Surg Am | 2006 | 10.2106/JBJS.F.00282 | English |
| 18811029 | Medium-term results of patellofemoral joint arthroplasty | Mohammed R, Jimulia T, Durve K, Bansal M, Green M, Learmonth D. | Acta Orthop Belg | 2008 |  | English |
| 22111771 | The Warwick patellofemoral arthroplasty trial: a randomised clinical trial of total knee arthroplasty versus patellofemoral arthroplasty in patients with severe arthritis of the patellofemoral joint | Odumenya M, McGuinness K, Achten J, Parsons N, Spalding T, Costa M. | BMC Musculoskelet Disord | 2011 | 10.1186/1471-2474-12-265 | English |
| 21489801 | Functional relevance of patellofemoral thickness before and after unicompartmental patellofemoral replacement | Mofidi A, Bajada S, Holt MD, Davies AP. | Knee | 2012 | 10.1016/j.knee.2011.03.002 | English |
| 26908975 | Patellofemoral arthroplasty in a bilateral Syme's amputee | Kanna R, Barrett DS. | J Clin Orthop Trauma | 2016 | 10.1016/j.jcot.2015.09.004 | English |
| 16239783 | Patellofemoral arthroplasty: an update | Argenson JN, Flecher X, Parratte S, Aubaniac JM. | Clin Orthop Relat Res | 2005 | 10.1097/01.blo.0000187061.27573.70 | English |
| 22704638 | Differences in the stress distribution in the distal femur between patellofemoral joint replacement and total knee replacement: a finite element study | van Jonbergen HP, Innocenti B, Gervasi GL, Labey L, Verdonschot N. | J Orthop Surg Res | 2012 | 10.1186/1749-799X-7-28 | English |
| 35236486 | Patient-related outcomes of patellofemoral arthroplasty: experience of a single center | Abeysekera WYM, Schenk W. | Arthroplasty | 2021 | 10.1186/s42836-021-00074-8 | English |
| 30662710 | The evolution and role of patellofemoral joint arthroplasty: The road less travelled, but not forgotten | Roussot MA, Haddad FS. | Bone Joint Res | 2019 | 10.1302/2046-3758.712.BJR-2018-0303 | English |
| 21964234 | Patellofemoral arthroplasty: 7-year mean follow-up | Mont MA, Johnson AJ, Naziri Q, Kolisek FR, Leadbetter WB. | J Arthroplasty | 2012 | 10.1016/j.arth.2011.07.010 | English |
| 32659820 | Obesity Does Not Affect Patient-Reported Outcomes following Patellofemoral Arthroplasty | Tishelman JC, Pyne A, Kahlenberg CA, Gruskay JA, Strickland SM. | J Knee Surg | 2022 | 10.1055/s-0040-1713862 | English |
| 30617612 | Return to the operating room after patellofemoral arthroplasty versus total knee arthroplasty for isolated patellofemoral arthritis-a systematic review | Woon CYL, Christ AB, Goto R, Shanaghan K, Shubin Stein BE, Gonzalez Della Valle A. | Int Orthop | 2019 | 10.1007/s00264-018-04280-z | English |
| 27916580 | Annual revision rates of partial versus total knee arthroplasty: A comparative meta-analysis | Chawla H, van der List JP, Christ AB, Sobrero MR, Zuiderbaan HA, Pearle AD. | Knee | 2017 | 10.1016/j.knee.2016.11.006 | English |
| 36317169 | Patellofemoral arthroplasty in combination with high tibial osteotomy can achieve good outcome for patients with medial-patellofemoral osteoarthritis | Peng Y, Lin W, Zhang Y, Wang F. | Front Surg | 2022 | 10.3389/fsurg.2022.999208 | English |
| 25737384 | Patellar Fracture Following Patellofemoral Arthroplasty | King AH, Engasser WM, Sousa PL, Arendt EA, Dahm DL. | J Arthroplasty | 2015 | 10.1016/j.arth.2015.02.007 | English |
| 21290009 | Patellofemoral arthroplasty versus total knee arthroplasty in patients with isolated patellofemoral osteoarthritis | Dahm DL, Al-Rayashi W, Dajani K, Shah JP, Levy BA, Stuart MJ. | Am J Orthop (Belle Mead NJ) | 2010 |  | English |
| 32178848 | Total knee arthroplasty reduces knee extension torque in-vitro and patellofemoral arthroplasty does not | Joseph MN, Carmont MR, Tailor H, Stephen JM, Amis AA. | J Biomech | 2020 | 10.1016/j.jbiomech.2020.109739 | English |
| 19234887 | Conversion of patellofemoral arthroplasty to total knee arthroplasty: A matched case-control study of 13 patients | van Jonbergen HP, Werkman DM, van Kampen A. | Acta Orthop | 2009 | 10.1080/17453670902805031 | English |
| 16769215 | Arthritis progression after patellofemoral joint replacement | Nicol SG, Loveridge JM, Weale AE, Ackroyd CE, Newman JH. | Knee | 2006 | 10.1016/j.knee.2006.04.005 | English |
| 22927894 | Patellofemoral arthroplasty: a systematic review of the literature | Tarassoli P, Punwar S, Khan W, Johnstone D. | Open Orthop J | 2012 | 10.2174/1874325001206010340 | English |
| 32228074 | Patellofemoral arthroplasty is cheaper and more effective in the short term than total knee arthroplasty for isolated patellofemoral osteoarthritis: cost-effectiveness analysis based on a randomized trial | Fredborg C, Odgaard A, Sørensen J. | Bone Joint J | 2020 | 10.1302/0301-620X.102B4.BJJ-2018-1580.R3 | English |
| 30168762 | The Avon patellofemoral joint arthroplasty: two- to 18-year results of a large single-centre cohort | Metcalfe AJ, Ahearn N, Hassaballa MA, Parsons N, Ackroyd CE, Murray JR, Robinson JR, Eldridge JD, Porteous AJ. | Bone Joint J | 2018 | 10.1302/0301-620X.100B9.BJJ-2018-0174.R1 | English |
| 34078392 | The short-term effectiveness and safety of second-generation patellofemoral arthroplasty and total knee arthroplasty on isolated patellofemoral osteoarthritis: a systematic review and meta-analysis | Li C, Li Z, Shi L, Gao F, Sun W. | J Orthop Surg Res | 2021 | 10.1186/s13018-021-02509-z | English |
| 28211301 | Early results of patellofemoral inlay resurfacing arthroplasty using the HemiCap Wave prosthesis | Patel A, Haider Z, Anand A, Spicer D. | J Orthop Surg (Hong Kong) | 2017 | 10.1177/2309499017692705 | English |
| 26790802 | Outcome of patellofemoral arthroplasty, determinants for success | Willekens P, Victor J, Verbruggen D, Vande Kerckhove M, Van Der Straeten C. | Acta Orthop Belg | 2015 |  | English |
| 21987361 | Complications after patello-femoral versus total knee replacement in the treatment of isolated patello-femoral osteoarthritis. A meta-analysis | Dy CJ, Franco N, Ma Y, Mazumdar M, McCarthy MM, Gonzalez Della Valle A. | Knee Surg Sports Traumatol Arthrosc | 2012 | 10.1007/s00167-011-1677-8 | English |
| 36582575 | Functional Outcomes, Survival Rate, and Complications of Patellofemoral Arthroplasty: Mid-Term Results From Independent Center | Jagadeesh N, Sales-Fernández R, Pammi S, Kariya A. | Cureus | 2022 | 10.7759/cureus.31945 | English |
| 28768779 | Cost effectiveness of patellofemoral versus total knee arthroplasty in younger patients | Chawla H, Nwachukwu BU, van der List JP, Eggman AA, Pearle AD, Ghomrawi HM. | Bone Joint J | 2017 | 10.1302/0301-620X.99B8.BJJ-2016-1032.R1 | English |
| 18399598 | Controversies and techniques in the surgical management of patellofemoral arthritis | Mihalko WM, Boachie-Adjei Y, Spang JT, Fulkerson JP, Arendt EA, Saleh KJ. | Instr Course Lect | 2008 |  | English |
| 34602157 | Editorial Commentary: Is the Pendulum "Swinging" Away From Patellar Fixation in Medial Patellofemoral Ligament Reconstruction? | Sherman SL, Curtis DM. | Arthroscopy | 2021 | 10.1016/j.arthro.2021.06.001 | English |
| 20044679 | The Avon patellofemoral joint replacement: Five-year results from an independent centre | Odumenya M, Costa ML, Parsons N, Achten J, Dhillon M, Krikler SJ. | J Bone Joint Surg Br | 2010 | 10.1302/0301-620X.92B1.23135 | English |
| 15991118 | Patellofemoral arthroplasty: the third compartment | Lotke PA, Lonner JH, Nelson CL. | J Arthroplasty | 2005 | 10.1016/j.arth.2005.03.011 | English |
| 30264243 | Similar postoperative patient-reported outcome in both second generation patellofemoral arthroplasty and total knee arthroplasty for treatment of isolated patellofemoral osteoarthritis: a systematic review | Bunyoz KI, Lustig S, Troelsen A. | Knee Surg Sports Traumatol Arthrosc | 2019 | 10.1007/s00167-018-5151-8 | English |
| 34513103 | Total Hip Arthroplasty Concomitant with Patellofemoral Arthroplasty and Medial Patellofemoral Ligament Reconstruction for a Patient with Patellar Dislocation Combined with Hip Dysplasia: A Case Report of a Successful Outcome at 5-Year Follow-Up | Iseki T, Iseki T, Okahisa S, Yoshiya S, Fukunishi S, Tachibana T. | Case Rep Orthop | 2021 | 10.1155/2021/9970975 | English |
| 26430081 | Long-term results of compartmental arthroplasties of the knee: Long term results of partial knee arthroplasty | Parratte S, Ollivier M, Lunebourg A, Abdel MP, Argenson JN. | Bone Joint J | 2015 | 10.1302/0301-620X.97B10.36426 | English |
| 20021240 | Scientific evidence for the use of modern patellofemoral arthroplasty | Gupta RR, Zywiel MG, Leadbetter WB, Bonutti P, Mont MA. | Expert Rev Med Devices | 2010 | 10.1586/erd.09.53 | English |
| 9586725 | Patellofemoral arthrosis: the treatment options | Oberlander MA, Baker CL, Morgan BE. | Am J Orthop (Belle Mead NJ) | 1998 |  | English |
| 16330515 | Anterior knee pain: diagnosis and treatment | Post WR. | J Am Acad Orthop Surg | 2005 | 10.5435/00124635-200512000-00006 | English |
| 34792611 | Insufficient evidence to confirm benefits of custom partial knee arthroplasty: a systematic review | Demey G, Müller JH, Liebensteiner M, Pilot P, Nover L; European Knee Associates (EKA); Kort N. | Knee Surg Sports Traumatol Arthrosc | 2022 | 10.1007/s00167-021-06766-7 | English |
| 17768216 | Custom patellofemoral arthroplasty of the knee. Surgical technique | Sisto DJ, Sarin VK. | J Bone Joint Surg Am | 2007 | 10.2106/JBJS.G.00186 | English |
| 16818972 | Custom patellofemoral arthroplasty of the knee | Sisto DJ, Sarin VK. | J Bone Joint Surg Am | 2006 | 10.2106/JBJS.E.00382 | English |
| 36215923 | Knee arthroplasty utilization trends from 2010 to 2019 | Fuller SI, Cohen JS, Malyavko A, Agarwal AR, Stake S, Golladay GJ, Thakkar SC. | Knee | 2022 | 10.1016/j.knee.2022.09.006 | English |
| 17824336 | Patellofemoral arthritis and its management with isolated patellofemoral replacement: a personal experience | Newman JH. | Orthopedics | 2007 |  | English |
| 19949120 | The Avon patellofemoral joint replacement: independent assessment of early functional outcomes | Starks I, Roberts S, White SH. | J Bone Joint Surg Br | 2009 | 10.1302/0301-620X.91B12.23018 | English |
| 25023661 | Patellofemoral arthroplasty influences tibiofemoral kinematics: the effect of patellar thickness | Vandenneucker H, Labey L, Victor J, Vander Sloten J, Desloovere K, Bellemans J. | Knee Surg Sports Traumatol Arthrosc | 2014 | 10.1007/s00167-014-3160-9 | English |
| 19057900 | Patellofemoral arthroplasty: a multi-centre study with minimum 2-year follow-up | Leadbetter WB, Kolisek FR, Levitt RL, Brooker AF, Zietz P, Marker DR, Bonutti PM, Mont MA. | Int Orthop | 2009 | 10.1007/s00264-008-0692-y | English |
| 2229117 | The patellofemoral joint after total knee arthroplasty without patellar resurfacing | Picetti GD 3rd, McGann WA, Welch RB. | J Bone Joint Surg Am | 1990 |  | English |
| 29654550 | Bicompartmental (uni plus patellofemoral) versus total knee arthroplasty: a match-paired study | Biazzo A, Silvestrini F, Manzotti A, Confalonieri N. | Musculoskelet Surg | 2019 | 10.1007/s12306-018-0540-1 | English |
| 36098748 | A radiological index that influences the outcome following patellofemoral joint arthroplasty: the anterior trochlea offset ratio | Aweid O, Ahearn N, Metcalfe AJ, Eldridge J, Porteous A, Murray JR; Bristol Knee Group. | Knee Surg Sports Traumatol Arthrosc | 2023 | 10.1007/s00167-022-07085-1 | English |
| 31136442 | What Is the Risk of Repeat Revision When Patellofemoral Replacement Is Revised to TKA? An Analysis of 482 Cases From a Large National Arthroplasty Registry | Lewis PL, Graves SE, Cuthbert A, Parker D, Myers P. | Clin Orthop Relat Res | 2019 | 10.1097/CORR.0000000000000541 | English |
| 31121631 | Patellofemoral Arthroplasty: Short-Term Complications and Risk Factors | Rezzadeh K, Behery OA, Kester BS, Dogra T, Vigdorchik J, Schwarzkopf R. | J Knee Surg | 2020 | 10.1055/s-0039-1688960 | English |
| 17142442 | Indications, contraindications, and pitfalls of patellofemoral arthroplasty | Leadbetter WB, Seyler TM, Ragland PS, Mont MA. | J Bone Joint Surg Am | 2006 | 10.2106/JBJS.F.00856 | English |
| 25079134 | Patellofemoral arthroplasty: outcomes and factors associated with early progression of tibiofemoral arthritis | Dahm DL, Kalisvaart MM, Stuart MJ, Slettedahl SW. | Knee Surg Sports Traumatol Arthrosc | 2014 | 10.1007/s00167-014-3202-3 | English |
| 29309448 | Return to Activities After Patellofemoral Arthroplasty | Shubin Stein BE, Brady JM, Grawe B, Tuakli-Wosornu Y, Nguyen JT, Wolfe E, Voigt M, Mahony G, Strickland S. | Am J Orthop (Belle Mead NJ) | 2017 |  | English |
| 26151039 | Bi-unicompartmental and combined uni plus patellofemoral replacement: indications and surgical technique | Romagnoli S, Marullo M, Massaro M, Rustemi E, D'Amario F, Corbella M. | Joints | 2015 | 10.11138/jts/2015.3.1.042 | English |
| 17323632 | Outcomes of patellofemoral replacement in total knee arthroplasty using meticulous techniques | Clyburn TA, Weitz-Marshall A, Ambrose CM, Ursua V. | Orthopedics | 2007 | 10.3928/01477447-20070201-13 | English |
| 35348817 | Patellofemoral arthroplasty: obesity linked to high risk of revision and progression of medial tibiofemoral osteoarthritis | Marullo M, Bargagliotti M, Vigano' M, Lacagnina C, Romagnoli S. | Knee Surg Sports Traumatol Arthrosc | 2022 | 10.1007/s00167-022-06947-y | English |
| 24426856 | Computer-assisted navigation in patellofemoral arthroplasty: a new technique to improve rotational position of the trochlea | Hernigou P, Flouzat-Lachaniette CH, Delblond W, Duffiet P, Julian D. | HSS J | 2013 | 10.1007/s11420-013-9328-x | English |
| 21464487 | The low contact stress patellofemoral replacement: high early failure rate | Charalambous CP, Abiddin Z, Mills SP, Rogers S, Sutton P, Parkinson R. | J Bone Joint Surg Br | 2011 | 10.1302/0301-620X.93B4.25899 | English |
| 18814615 | Patellofemoral replacement: the third compartment | Minas T. | Orthopedics | 2008 | 10.3928/01477447-20080901-24 | English |
| 32348089 | Short-term Revision Risk of Patellofemoral Arthroplasty Is High: An Analysis from Eight Large Arthroplasty Registries | Lewis PL, Tudor F, Lorimer M, McKie J, Bohm E, Robertsson O, Makela KT, Haapakoski J, Furnes O, Bartz-Johannessen C, Nelissen RGHH, Van Steenbergen LN, Fithian DC, Prentice HA. | Clin Orthop Relat Res | 2020 | 10.1097/CORR.0000000000001268 | English |
| 19301809 | Patellofemoral arthroplasty with a custom-fit femoral prosthesis | Butler JE, Shannon R. | Orthopedics | 2009 |  | English |
| 20619661 | Medial patellofemoral ligament reconstruction for subluxating patellofemoral arthroplasty | Carmont MR, Crane T, Thompson P, Spalding T. | Knee | 2011 | 10.1016/j.knee.2010.02.011 | English |
| 27522543 | Midterm Outcome of Avon Patellofemoral Arthroplasty for Posttraumatic Unicompartmental Osteoarthritis | Konan S, Haddad FS. | J Arthroplasty | 2016 | 10.1016/j.arth.2016.06.005 | English |
| 32970202 | Pre-operative patella alta does not affect midterm clinical outcomes and survivorship of patellofemoral arthroplasty | Bernard CD, Pareek A, Sabbag CM, Parkes CW, Krych AJ, Cummings NM, Dahm DL. | Knee Surg Sports Traumatol Arthrosc | 2021 | 10.1007/s00167-020-06205-z | English |
| 31136443 | CORR Insights®: What Is the Risk of Repeat Revision When Patellofemoral Replacement Is Revised to TKA? An Analysis of 482 Cases From a Large National Arthroplasty Registry | Hallstrom BR. | Clin Orthop Relat Res | 2019 | 10.1097/CORR.0000000000000583 | English |
| 33835097 | Reply to the Letter to the Editor: Short-term Revision Risk of Patellofemoral Arthroplasty is High: An Analysis From Eight Large Arthroplasty Registries | Lewis PL. | Clin Orthop Relat Res | 2021 | 10.1097/CORR.0000000000001718 | English |
| 23711753 | Advanced patellofemoral cartilage lesions in patients younger than 50 years of age: is there an ideal operative option? | Noyes FR, Barber-Westin SD. | Arthroscopy | 2013 | 10.1016/j.arthro.2013.03.077 | English |
| 24190590 | Design, operative technique and ten-year results of the Hermes™ patellofemoral arthroplasty | Hernigou P, Caton J. | Int Orthop | 2014 | 10.1007/s00264-013-2158-0 | English |
| 29614710 | Patient-reported outcome measures (PROMs) in patients undergoing patellofemoral arthroplasty and total knee replacement: A comparative study | Perrone FL, Baron S, Suero EM, Lausmann C, Kendoff D, Zahar A, Gehrke T, Citak M. | Technol Health Care | 2018 | 10.3233/THC-181185 | English |
| 26984650 | Patella component loosening--A case report | Bloemheuvel EM, van Rooij WM, van den Besselaar M. | Acta Orthop Belg | 2016 |  | English |
| 12925646 | Patellofemoral arthroplasty | Smith A, Lucas D. | J Bone Joint Surg Am | 2003 | 10.2106/00004623-200308000-00029 | English |
| 8926124 | Unicompartmental knee arthroplasty with patelloplasty | Antoniou J, Hadjipavlou A, Enker P, Antoniou A. | Int Orthop | 1996 | 10.1007/s002640050038 | English |
| 35004156 | Arthroscopic Debridement, Facetectomy, and Synovectomy for Isolated Patellofemoral Osteoarthritis | Zhao J. | Arthrosc Tech | 2021 | 10.1016/j.eats.2021.08.021 | English |
| 19632120 | Return to work following knee arthroplasty | Foote JA, Smith HK, Jonas SC, Greenwood R, Weale AE. | Knee | 2010 | 10.1016/j.knee.2009.06.001 | English |
| 17975374 | Total knee arthroplasty for isolated patellofemoral arthritis in younger patients | Meding JB, Wing JT, Keating EM, Ritter MA. | Clin Orthop Relat Res | 2007 | 10.1097/BLO.0b013e3181576069 | English |
| 36580050 | Reduced Early Revision Surgery and Medical Complications in Computer-assisted Knee Arthroplasty Compared With Non-computer-assisted Arthroplasty | Malyavko A, Cohen JS, Fuller SI, Agarwal AR, Golladay GJ, Thakkar SC. | J Am Acad Orthop Surg | 2023 | 10.5435/JAAOS-D-22-00596 | English |
| 12905797 | [Patellofemoral replacement for severe patellofemoral osteoarthritis: a 2-10 years follow-up study] | Zhang J, Ye QB, Qiu GX, Wang YP. | Zhongguo Yi Xue Ke Xue Yuan Xue Bao | 2002 |  | Chinese |
| 33835108 | Letter to the Editor: Short-term Revision Risk of Patellofemoral Arthroplasty is High: An Analysis From Eight Large Arthroplasty Registries | van Jonbergen HW, Kleinlugtenbelt YV. | Clin Orthop Relat Res | 2021 | 10.1097/CORR.0000000000001717 | English |
| 18078881 | Ipsilateral patellofemoral arthroplasty and autogenous osteochondral femoral condylar transplantation | Lonner JH, Mehta S, Booth RE Jr. | J Arthroplasty | 2007 | 10.1016/j.arth.2005.08.012 | English |
| 33088840 | Comparing Return to Sports After Patellofemoral and Knee Arthroplasty in an Age- and Sex-Matched Cohort | Schneider BL, Ling DI, Kleebad LJ, Strickland S, Pearle A. | Orthop J Sports Med | 2020 | 10.1177/2325967120957425 | English |
| 23255644 | Mobile-bearing, congruent patellofemoral prosthesis: short-term results | Yadav B, Shaw D, Radcliffe G, Dachepalli S, Kluge W. | J Orthop Surg (Hong Kong) | 2012 | 10.1177/230949901202000317 | English |
| 31320188 | The Combination of Inlay Patellofemoral Arthroplasty and Medial Unicompartmental Knee Arthroplasty Versus Total Knee Arthroplasty for Mediopatellofemoral Osteoarthritis: A Comparison of Mid-Term Outcomes | Uluyardimci E, Isik C, Tahta M, Emre F, Cepni S, Oltulu I. | J Arthroplasty | 2019 | 10.1016/j.arth.2019.06.043 | English |
| 24980643 | Are revisions of patellofemoral arthroplasties more like primary or revision TKAs | Parratte S, Lunebourg A, Ollivier M, Abdel MP, Argenson JN. | Clin Orthop Relat Res | 2015 | 10.1007/s11999-014-3756-x | English |
| 23723274 | Early revisions of the Femoro-Patella Vialla joint replacement | Williams DP, Pandit HG, Athanasou NA, Murray DW, Gibbons CL. | Bone Joint J | 2013 | 10.1302/0301-620X.95B6.31355 | English |
| 31888356 | Mid-term survivorship and patient-reported outcomes of robotic-arm assisted partial knee arthroplasty | Burger JA, Kleeblad LJ, Laas N, Pearle AD. | Bone Joint J | 2020 | 10.1302/0301-620X.102B1.BJJ-2019-0510.R1 | English |
| 25660614 | Four-Year Follow Up Outcome Study of Patellofemoral Arthroplasty at a Single Institution | Goh GS, Liow MH, Tay DK, Lo NN, Yeo SJ. | J Arthroplasty | 2015 | 10.1016/j.arth.2015.01.020 | English |
| 30797677 | Hermes patellofemoral arthroplasty: Annual revision rate and clinical results after two to 20 years of follow-up | Bohu Y, Klouche S, Sezer HB, Gerometta A, Lefevre N, Herman S. | Knee | 2019 | 10.1016/j.knee.2019.01.014 | English |
| 24426854 | MRI after patellofemoral replacement: the component-bone interface and rotational alignment | Heyse TJ, Figiel J, Hähnlein U, Timmesfeld N, Schofer MD, Fuchs-Winkelmann S, Efe T. | HSS J | 2013 | 10.1007/s11420-013-9336-x | English |
| 10546602 | Total knee replacement for patients with patellofemoral arthritis | Laskin RS, van Steijn M. | Clin Orthop Relat Res | 1999 |  | English |
| 28840268 | No bias for developer publications and no difference between first-generation trochlear-resurfacing versus trochlear-cutting implants in 15,306 cases of patellofemoral joint arthroplasty | Reihs B, Reihs F, Labek G, Hochegger M, Leithner A, Böhler N, Sadoghi P. | Knee Surg Sports Traumatol Arthrosc | 2018 | 10.1007/s00167-017-4692-6 | English |
| 22009560 | In vivo sagittal plane kinematics of the FPV patellofemoral replacement | Monk AP, van Duren BH, Pandit H, Shakespeare D, Murray DW, Gill HS. | Knee Surg Sports Traumatol Arthrosc | 2012 | 10.1007/s00167-011-1717-4 | English |
| 30721343 | Early outcomes of an anatomic trochlear-cutting patellofemoral arthroplasty: patient selection is key | Dejour D, Saffarini M, Malemo Y, Pungitore M, Valluy J, Nover L, Demey G. | Knee Surg Sports Traumatol Arthrosc | 2019 | 10.1007/s00167-019-05368-8 | English |
| 33114034 | Medical Interventions for Patellofemoral Pain and Patellofemoral Osteoarthritis: A Systematic Review | Macri EM, Hart HF, Thwaites D, Barton CJ, Crossley KM, Bierma-Zeinstra SMA, van Middelkoop M. | J Clin Med | 2020 | 10.3390/jcm9113397 | English |
| 15995418 | A modular prosthesis for patellofemoral arthroplasty: design and initial results | Merchant AC. | Clin Orthop Relat Res | 2005 | 10.1097/01.blo.0000171917.47869.6c | English |
| 15995414 | Development and early results of a new patellofemoral arthroplasty | Ackroyd CE, Chir B. | Clin Orthop Relat Res | 2005 | 10.1097/01.blo.0000171914.94503.d1 | English |
| 33571948 | Recurrent patellar dislocations with patellar cartilage defects: A pain in the knee? | Dalal S, Setia P, Debnath A, Guro R, Kotwal R, Chandratreya A. | Knee | 2021 | 10.1016/j.knee.2021.01.019 | English |
| 20004900 | Strain shielding in distal femur after patellofemoral arthroplasty under different activity conditions | Meireles S, Completo A, António Simões J, Flores P. | J Biomech | 2010 | 10.1016/j.jbiomech.2009.09.048 | English |
| 32246606 | [Functional results in isolated patellofemoral arthrosis treated by arthroscopy plus Fulkerson osteotomy vs patellofemoral arthroplasty] | Martínez-Sánchez JA, Blanco-Bucio P, Valencia-Martínez G. | Acta Ortop Mex | 2019 |  | Spanish |
| 17892539 | Effects of laterally wedged insoles on symptoms and disease progression in medial knee osteoarthritis: a protocol for a randomised, double-blind, placebo controlled trial | Kim BennellKelly-Ann BowlesCraig PayneFlavia CicuttiniRichard OsborneAnthony HarrisRana Hinman | BMC Musculoskeletal Disorders | 2007 | 10.1186/1471-2474-8-96 | English |
| 11728076 | The Richards type II patellofemoral arthroplasty: 26 cases followed for 1-20 years | de Winter WE, Feith R, van Loon CJ. | Acta Orthop Scand | 2001 | 10.1080/000164701753532826 | English |
| 17197318 | In vivo sagittal plane kinematics of the Avon patellofemoral arthroplasty | Hollinghurst D, Stoney J, Ward T, Pandit H, Beard D, Murray DW. | J Arthroplasty | 2007 | 10.1016/j.arth.2006.02.160 | English |
| 30732508 | Patient satisfaction reporting for patellofemoral arthroplasty is significantly lacking: a systematic review | Tishelman JC, Kahlenberg CA, Nwachukwu BU, Gruskay J, Strickland SM. | Phys Sportsmed | 2019 | 10.1080/00913847.2019.1580913 | English |
| 22704031 | Trochlear inclination angles in normal and dysplastic knees | Kamath AF, Slattery TR, Levack AE, Wu CH, Kneeland JB, Lonner JH. | J Arthroplasty | 2013 | 10.1016/j.arth.2012.04.017 | English |
| 33934658 | Mid- to long-term follow-up of combined small implants | Rossi SMP, Perticarini L, Clocchiatti S, Ghiara M, Benazzo F. | Bone Joint J | 2021 | 10.1302/0301-620X.103B5.BJJ-2020-0720.R3 | English |
| 33937460 | Tibial Osteolysis After Long-Term Isolated Polyethylene Patellar Resurfacing | Apple AE, Montgomery CO, Mears SC. | Arthroplast Today | 2021 | 10.1016/j.artd.2021.03.012 | English |
| 33086962 | Combined patellofemoral arthroplasty and medial patellofemoral ligament reconstruction for chronic patellar instability with trochlear dysplasia: a report of two cases | Yamagami R, Inui H, Taketomi S, Tanaka S. | Mod Rheumatol Case Rep | 2020 | 10.1080/24725625.2019.1638048 | English |
| 32867858 | Unicompartmental vs. segmental bicompartmental vs. total knee replacement: comparison of clinical outcomes | Al-Dadah O, Hawes G, Chapman-Sheath PJ, Tice JW, Barrett DS. | Knee Surg Relat Res | 2020 | 10.1186/s43019-020-00065-0 | English |
| 28271370 | Inadequacy of computed tomography for pre-operative planning of patellofemoral arthroplasty | Saffarini M, Müller JH, La Barbera G, Hannink G, Cho KJ, Toanen C, Dejour D. | Knee Surg Sports Traumatol Arthrosc | 2018 | 10.1007/s00167-017-4474-1 | English |
| 7497663 | Is there a place for patellofemoral arthroplasty? | Argenson JN, Guillaume JM, Aubaniac JM. | Clin Orthop Relat Res | 1995 |  | English |
| 20406477 | Distal femoral bone mineral density decreases following patellofemoral arthroplasty: 1-year follow-up study of 14 patients | van Jonbergen HP, Koster K, Labey L, Innocenti B, van Kampen A. | BMC Musculoskelet Disord | 2010 | 10.1186/1471-2474-11-74 | English |
| 31754729 | Smoking, unemployment, female sex, obesity, and medication use yield worse outcomes in patellofemoral arthroplasty | Desai VS, Pareek A, DeDeugd CM, Sabbag OD, Krych AJ, Cummings NM, Dahm DL. | Knee Surg Sports Traumatol Arthrosc | 2020 | 10.1007/s00167-019-05704-y | English |
| 34689224 | Medial bicompartmental arthroplasty patients display more normal gait and improved satisfaction, compared to matched total knee arthroplasty patients | Garner AJ, Dandridge OW, van Arkel RJ, Cobb JP. | Knee Surg Sports Traumatol Arthrosc | 2023 | 10.1007/s00167-021-06773-8 | English |
| 17356140 | The Avon patellofemoral arthroplasty: five-year survivorship and functional results | Ackroyd CE, Newman JH, Evans R, Eldridge JD, Joslin CC. | J Bone Joint Surg Br | 2007 | 10.1302/0301-620X.89B3.18062 | English |
| 35191830 | Combined procedures with unicompartmental knee arthroplasty: High risk of stiffness but promising concept in selected indications | Derreveaux V, Schmidt A, Shatrov J, Sappey-Marinier E, Batailler C, Servien E, Lustig S. | SICOT J | 2022 | 10.1051/sicotj/2022002 | English |
| 31515593 | The patient results and satisfaction of knee arthroplasty in a validated grading system | Oosthuizen CR, Van Der Straeten C, Maposa I, Snyckers CH, Vermaak DP, Magobotha S. | Int Orthop | 2019 | 10.1007/s00264-019-04412-z | English |
| 29021089 | Patellofemoral arthritis treated with resurfacing implant: Clinical outcome and complications at a minimum two-year follow-up | Zicaro JP, Yacuzzi C, Astoul Bonorino J, Carbo L, Costa-Paz M. | Knee | 2017 | 10.1016/j.knee.2017.09.003 | English |
| 19224212 | Midterm clinical results of the Autocentric II patellofemoral prosthesis | van Wagenberg JM, Speigner B, Gosens T, de Waal Malefijt J. | Int Orthop | 2009 | 10.1007/s00264-009-0719-z | English |
| 27193203 | The effect of axial rotation of the anterior resection plane in patellofemoral arthroplasty | Cho KJ, Erasmus PJ, Müller JH. | Knee | 2016 | 10.1016/j.knee.2016.04.006 | English |
| 16146626 | Is anterior knee pain a predisposing factor to patellofemoral osteoarthritis? | Utting MR, Davies G, Newman JH. | Knee | 2005 | 10.1016/j.knee.2004.12.006 | English |
| 23594895 | [Dislocation of the polyethylene in a patello-femoral replacement with a movable patella] | Sangüesa Nebot MJ, Cabanes Soriano F, Fernández Gabarda R, Darder Prats A. | Rev Esp Cir Ortop Traumatol | 2012 | 10.1016/j.recot.2012.05.008 | Spanish |
| 26094823 | Obesity and the absence of trochlear dysplasia increase the risk of revision in patellofemoral arthroplasty | Liow MH, Goh GS, Tay DK, Chia SL, Lo NN, Yeo SJ. | Knee | 2016 | 10.1016/j.knee.2015.05.009 | English |
| 19347881 | HyBAR: hybrid bone-attached robot for joint arthroplasty | Song S, Mor A, Jaramaz B. | Int J Med Robot | 2009 | 10.1002/rcs.254 | English |
| 32739080 | No Major Functional Benefit After Bicompartmental Knee Arthroplasty Compared to Total Knee Arthroplasty at 5-Year Follow-Up | Schrednitzki D, Beier A, Marx A, Halder AM. | J Arthroplasty | 2020 | 10.1016/j.arth.2020.07.003 | English |
| 24696005 | Evidence of trochlear dysplasia in patellofemoral arthroplasty designs | Saffarini M, Ntagiopoulos PG, Demey G, Le Negaret B, Dejour DH. | Knee Surg Sports Traumatol Arthrosc | 2014 | 10.1007/s00167-014-2967-8 | English |
| 36516356 | Expression of Concern: Patellofemoral Arthroplasty Results in Better Time-weighted Patient-reported Outcomes After 6 Years Than TKA: A Randomized Controlled Trial |  | Clin Orthop Relat Res | 2023 | 10.1097/CORR.0000000000002523 | English |
| 31791722 | The midterm results of a cohort study of patellofemoral arthroplasty from a non-designer centre using an asymmetric trochlear prosthesis | Rammohan R, Gupta S, Lee PYF, Chandratreya A. | Knee | 2019 | 10.1016/j.knee.2019.10.026 | English |
| 31259645 | Failure modes of patellofemoral arthroplasty-registries vs. clinical studies: a systematic review | Bendixen NB, Eskelund PW, Odgaard A. | Acta Orthop | 2019 | 10.1080/17453674.2019.1634865 | English |
| 15024580 | The Lubinus patellofemoral arthroplasty: a series of 17 cases | Board TN, Mahmood A, Ryan WG, Banks AJ. | Arch Orthop Trauma Surg | 2004 | 10.1007/s00402-004-0645-x | English |
| 28522245 | Pin Site Complications Associated With Computer-Assisted Navigation in Hip and Knee Arthroplasty | Kamara E, Berliner ZP, Hepinstall MS, Cooper HJ. | J Arthroplasty | 2017 | 10.1016/j.arth.2017.03.073 | English |
| 30291399 | A high level of satisfaction after bicompartmental individualized knee arthroplasty with patient-specific implants and instruments | Ogura T, Le K, Merkely G, Bryant T, Minas T. | Knee Surg Sports Traumatol Arthrosc | 2019 | 10.1007/s00167-018-5155-4 | English |
| 34583395 | Patient-Reported Outcomes and Risk Factors for Decreased Improvement after Patellofemoral Arthroplasty | Dai Y, Diao N, Lin W, Yang G, Kang H, Wang F. | J Knee Surg | 2023 | 10.1055/s-0041-1735159 | English |
| 22547249 | Prospective clinical and radiological two-year results after patellofemoral arthroplasty using an implant with an asymmetric trochlea design | Beitzel K, Schöttle PB, Cotic M, Dharmesh V, Imhoff AB. | Knee Surg Sports Traumatol Arthrosc | 2013 | 10.1007/s00167-012-2022-6 | English |
| 34241595 | Does bicompartmental knee arthroplasty hold an advantage over total knee arthroplasty? Systematic review and meta-analysis | Elbardesy H, Awad AK, McLeod A, Farahat ST, Sayed SZE, Guerin S, Harty J. | SICOT J | 2021 | 10.1051/sicotj/2021036 | English |
| 19888591 | UKA in combination with PFR at average 12-year follow-up | Heyse TJ, Khefacha A, Cartier P. | Arch Orthop Trauma Surg | 2010 | 10.1007/s00402-009-0997-3 | English |
| 27714438 | High mid-term revision rate after treatment of large, full-thickness cartilage lesions and OA in the patellofemoral joint using a large inlay resurfacing prosthesis: HemiCAP-Wave® | Laursen JO. | Knee Surg Sports Traumatol Arthrosc | 2017 | 10.1007/s00167-016-4352-2 | English |
| 18407278 | In vitro simulation and quantification of wear within the patellofemoral joint replacement | Ellison P, Barton DC, Esler C, Shaw DL, Stone MH, Fisher J. | J Biomech | 2008 | 10.1016/j.jbiomech.2008.02.029 | English |
| 12931801 | Long-term results of patellofemoral arthroplasty. A report of 56 arthroplasties with 17 years of follow-up | Kooijman HJ, Driessen AP, van Horn JR. | J Bone Joint Surg Br | 2003 |  | English |
| 17272470 | Custom patellofemoral replacement in the presence of trochlear dysplasia | Grelsamer RP. | J Bone Joint Surg Am | 2007 | 10.2106/00004623-200702000-00040 | English |
| 34146717 | Augmented reality in robotic assisted orthopaedic surgery: A pilot study | Iqbal H, Tatti F, Rodriguez Y Baena F. | J Biomed Inform | 2021 | 10.1016/j.jbi.2021.103841 | English |
| 27622149 | Femoro Patella Vialla patellofemoral arthroplasty: An independent assessment of outcomes at minimum 2-year follow-up | Halai M, Ker A, Anthony I, Holt G, Jones B, Blyth M. | World J Orthop | 2016 | 10.5312/wjo.v7.i8.487 | English |
| 32475247 | Patterns of cartilage loss and anterior cruciate ligament status in end-stage osteoarthritis of the knee | Scott CEH, Holland G, Krahelski O, Murray IR, Keating JF, Keenan OJF. | Bone Joint J | 2020 | 10.1302/0301-620X.102B6.BJJ-2019-1434.R1 | English |
| 8804284 | Patellofemoral arthroplasty. A 2- to 18-year followup study | Krajca-Radcliffe JB, Coker TP. | Clin Orthop Relat Res | 1996 |  | English |
| 18979927 | Patient-based outcomes in patellofemoral arthroplasty | Utukuri MM, Khanduja V, Somayaji HS, Dowd GS. | J Knee Surg | 2008 | 10.1055/s-0030-1247830 | English |
| 16627153 | Computer-assisted patellofemoral arthroplasty: a mechanism for optimizing rotation | Cossey AJ, Spriggins AJ. | J Arthroplasty | 2006 | 10.1016/j.arth.2005.08.010 | English |
| 32192817 | Aiming for anatomical femoral axis on the coronal plane leads to good-to-excellent short-term outcomes in isolated patellofemoral arthroplasty | Vasta S, Rosi M, Tecame A, Papalia R, Adravanti P. | Knee | 2020 | 10.1016/j.knee.2020.02.016 | English |
| 33272080 | Has the modern design of Attune total knee replacement improved outcome in patients with isolated patellofemoral arthritis? | Ashraf Y, Iqbal HJ, Senevirathana S, Ashraf T. | J Orthop Surg (Hong Kong) | 2020 | 10.1177/2309499020975553 | English |
| 18534464 | Revision patellofemoral arthroplasty: three- to seven-year follow-up | Hendrix MR, Ackroyd CE, Lonner JH. | J Arthroplasty | 2008 | 10.1016/j.arth.2007.10.019 | English |
| 21703794 | MRI after patellofemoral replacement: the preserved compartments | Heyse TJ, Figiel J, Hähnlein U, Timmesfeld N, Lakemeier S, Schofer MD, Fuchs-Winkelmann S, Efe T. | Eur J Radiol | 2012 | 10.1016/j.ejrad.2011.06.012 | English |
| 21596570 | Patellofemoral osteoarthritis treated by partial lateral facetectomy: results at long-term follow up | Wetzels T, Bellemans J. | Knee | 2012 | 10.1016/j.knee.2011.04.005 | English |
| 26231153 | A matched-pair comparison of inlay and onlay trochlear designs for patellofemoral arthroplasty: no differences in clinical outcome but less progression of osteoarthritis with inlay designs | Feucht MJ, Cotic M, Beitzel K, Baldini JF, Meidinger G, Schöttle PB, Imhoff AB. | Knee Surg Sports Traumatol Arthrosc | 2017 | 10.1007/s00167-015-3733-2 | English |
| 36479459 | Effectiveness of an Accelerated Rehabilitation Protocol After Tibial Tubercle Osteotomy | Morgan C, Bell RM, Burland JP, Kriscenski D, Ilinski A, Cote MP, Edgar CM. | Orthop J Sports Med | 2022 | 10.1177/23259671221133105 | English |
| 32044787 | Femoropatellar Osteoarthritis and Trochlear Femoral Bone Defect due to Giant Cell Tumor of the Knee: A Selected Patellofemoral Joint Arthroplasty and Reconstructive Technique: A Case Report | Conti A, Boffano M, Pellegrino P, Ratto N, Sabatini L, Piana R. | JBJS Case Connect | 2020 | 10.2106/JBJS.CC.19.00378 | English |
| 17518383 | MBARS: mini bone-attached robotic system for joint arthroplasty | Wolf A, Jaramaz B, Lisien B, DiGioia AM. | Int J Med Robot | 2005 | 10.1002/rcs.20 | English |
| 3180587 | Patellofemoral arthroplasty. A three- to nine-year follow-up study | Arciero RA, Toomey HE. | Clin Orthop Relat Res | 1988 |  | English |
| 17261986 | Functional outcomes after different types of knee arthroplasty: kneeling ability versus descending stairs | Hassaballa MA, Porteous AJ, Learmonth ID. | Med Sci Monit | 2007 |  | English |
| 23188904 | The correct rotation of the femoral component in patellofemoral replacement: a laboratory assessment of a surgical technique | Clark DA, Upadhyay N, Gillespie G, Wakeley C, Eldridge JD. | J Bone Joint Surg Br | 2012 | 10.1302/0301-620X.94B12.29506 | English |
| 33811265 | The lack of retropatellar resurfacing at index surgery is significantly associated with failure in patients following patellofemoral inlay arthroplasty: a multi-center study of more than 260 patients | Imhoff AB, Bartsch E, Becher C, Behrens P, Bode G, Cotic M, Diermeier T, Falk H, Feucht MJ, Haupt U, Hinterwimmer S, Holz J, Hutter R, Kaiser R, Knoblauch T, Nebelung W, Niemeyer P, O'Donnel T, Pagenstert G, Patzer T, Rose T, Rupp MC, Tischer T, Venjakob AJ, Vogt S, Pogorzelski J. | Knee Surg Sports Traumatol Arthrosc | 2022 | 10.1007/s00167-021-06544-5 | English |
| 29992071 | The Benefits of an In-Office Arthroscopy in the Diagnosis of Unresolved Knee Pain | Chapman GL, Amin NH. | Case Rep Orthop | 2018 | 10.1155/2018/6125676 | English |
| 26483259 | Significant Functional Improvement at 2 Years After Isolated Patellofemoral Arthroplasty With an Onlay Trochlear Implant, But Low Mental Health Scores Predispose to Dissatisfaction | Kazarian GS, Tarity TD, Hansen EN, Cai J, Lonner JH. | J Arthroplasty | 2016 | 10.1016/j.arth.2015.08.033 | English |
| 17142450 | Patellofemoral replacement polymer stress during daily activities: a finite element study | Morra EA, Greenwald AS. | J Bone Joint Surg Am | 2006 | 10.2106/JBJS.F.00585 | English |
| 31427243 | Early post-operative opioid consumption: A comparison between medial unicompartmental, patellofemoral, and total knee arthroplasty | Melnic CM, Kazarian ER, Dwyer MK, Domingo-Johnson EL, Freiberg AA, Bedair HS. | Knee | 2019 | 10.1016/j.knee.2019.06.016 | English |
| 15995419 | Long-term results with the first patellofemoral prosthesis | Cartier P, Sanouiller JL, Khefacha A. | Clin Orthop Relat Res | 2005 | 10.1097/01.blo.0000171918.24998.d1 | English |
| 32529388 | Clinical outcome of bi-unicompartmental knee arthroplasty for both medial and lateral femorotibial arthritis: a systematic review-is there proof of concept? | Wada K, Price A, Gromov K, Lustig S, Troelsen A. | Arch Orthop Trauma Surg | 2020 | 10.1007/s00402-020-03492-6 | English |
| 28661229 | The influence of malalignment and ageing following sterilisation by gamma irradiation in an inert atmosphere on the wear of ultra-high-molecular-weight polyethylene in patellofemoral replacements | Maiti R, Cowie RM, Fisher J, Jennings LM. | Proc Inst Mech Eng H | 2017 | 10.1177/0954411917696112 | English |
| 30276437 | High patient satisfaction with significant improvement in knee function and pain relief after mid-term follow-up in patients with isolated patellofemoral inlay arthroplasty | Imhoff AB, Feucht MJ, Bartsch E, Cotic M, Pogorzelski J. | Knee Surg Sports Traumatol Arthrosc | 2019 | 10.1007/s00167-018-5173-2 | English |
| 11922379 | The Lubinus patellofemoral arthroplasty: a five- to ten-year prospective study | Coleridge S. | J Bone Joint Surg Br | 2002 |  | English |
| 24395311 | Revision cartilage cell transplantation for failed autologous chondrocyte transplantation in chronic osteochondral defects of the knee | Vijayan S, Bentley G, Rahman J, Briggs TW, Skinner JA, Carrington RW. | Bone Joint J | 2014 | 10.1302/0301-620X.96B1.31979 | English |
| 22310419 | Patellar polyethylene spinout after low-contact stress, high-congruity, mobile-bearing patellofemoral arthroplasty | Amanatullah DF, Jamali AA. | Orthopedics | 2012 | 10.3928/01477447-20120123-27 | English |
| 32037873 | Prospective Outcomes of Cryopreserved Osteochondral Allograft for Patellofemoral Cartilage Defects at Minimum 2-Year Follow-up | Melugin HP, Ridley TJ, Bernard CD, Wischmeier D, Farr J, Stuart MJ, Macalena JA, Krych AJ. | Cartilage | 2021 | 10.1177/1947603520903420 | English |
| 28713612 | In vivo magnetic resonance imaging morphometry of the patella bone in South Indian population | Muhamed R, Saralaya VV, Murlimanju BV, Chettiar GK. | Anat Cell Biol | 2017 | 10.5115/acb.2017.50.2.99 | English |
| 32845359 | Reliable improvements in participation in low-impact sports following implantation of a patellofemoral inlay arthroplasty at mid-term follow-up | Pogorzelski J, Rupp MC, Ketzer C, Cotic M, Lutz P, Beeck S, Imhoff AB, Feucht MJ. | Knee Surg Sports Traumatol Arthrosc | 2021 | 10.1007/s00167-020-06245-5 | English |
| 20062723 | Patellofemoral arthroplasty for symptomatic nonunion after trochlear osteotomy for patellar instability: a case report | van Jonbergen HP, van Egmond K. | Cases J | 2009 | 10.1186/1757-1626-2-9086 | English |
| 18417346 | Pre-operative mental wellbeing and the outcome of knee replacement | Walton MJ, Newman JH. | Knee | 2008 | 10.1016/j.knee.2008.03.001 | English |
| 33126284 | Robotic-Assisted Patellofemoral Replacement-Correlation of Preoperative Planning with Intraoperative Implant Position and Early Clinical Experience: A Minimum 2-Year Follow-up | Selvaratnam V, Cattell A, Eyres KS, Toms AD, Phillips JRP, Mandalia VI. | J Knee Surg | 2022 | 10.1055/s-0040-1716848 | English |
| 30315327 | Derotational osteotomy at the distal femur is effective to treat patients with patellar instability | Imhoff FB, Cotic M, Liska F, Dyrna FGE, Beitzel K, Imhoff AB, Herbst E. | Knee Surg Sports Traumatol Arthrosc | 2019 | 10.1007/s00167-018-5212-z | English |
| 27052369 | Patella tracking and patella contact pressure in modular patellofemoral arthroplasty: a biomechanical in vitro analysis | Calliess T, Ettinger M, Schado S, Becher C, Hurschler C, Ostermeier S. | Arch Orthop Trauma Surg | 2016 | 10.1007/s00402-016-2451-7 | English |
| 11830378 | Treatment of patello-femoral arthritis using the Lubinus patello-femoral arthroplasty: a retrospective review | Smith AM, Peckett WR, Butler-Manuel PA, Venu KM, d'Arcy JC. | Knee | 2002 | 10.1016/s0968-0160(01)00127-2 | English |
| 19681331 | Dislocation of the mobile bearing component of a patellofemoral arthroplasty: a report of two cases | Witjes S, Van den Broek C, Koëter S, Van Loon C. | Acta Orthop Belg | 2009 |  | English |
| 19102828 | Manipulation under anaesthesia for stiffness following knee arthroplasty | Mohammed R, Syed S, Ahmed N. | Ann R Coll Surg Engl | 2009 | 10.1308/003588409X359321 | English |
| 26156155 | Patellectomy for osteoarthritis: a new tension preserving surgical technique to reconstruct the extensor mechanism with retrospective review of long-term follow-up | Asopa V, Willis-Owen C, Keene G. | J Orthop Surg Res | 2015 | 10.1186/s13018-015-0237-1 | English |
| 33051119 | Use of Natural Language Processing Algorithms to Identify Common Data Elements in Operative Notes for Knee Arthroplasty | Sagheb E, Ramazanian T, Tafti AP, Fu S, Kremers WK, Berry DJ, Lewallen DG, Sohn S, Maradit Kremers H. | J Arthroplasty | 2021 | 10.1016/j.arth.2020.09.029 | English |
| 23715952 | Results of a French multicentre retrospective experience with four hundred and eighteen failed unicondylar knee arthroplasties | Saragaglia D, Bonnin M, Dejour D, Deschamps G, Chol C, Chabert B, Refaie R; French Society of Hip and Knee. | Int Orthop | 2013 | 10.1007/s00264-013-1915-4 | English |
| 18372179 | Unique combination of patellofemoral joint arthroplasty with Osteochondral Autograft Transfer System (OATS) - a case series of six knees in five patients | Unnithan A, Jimulia T, Mohammed R, Learmonth DJ. | Knee | 2008 | 10.1016/j.knee.2008.01.007 | English |
| 24460149 | Biomechanical behaviour of cancellous bone on patellofemoral arthroplasty with Journey prosthesis: a finite element study | Castro AP, Completo A, Simões JA, Flores P. | Comput Methods Biomech Biomed Engin | 2015 | 10.1080/10255842.2013.870999 | English |
| 11476308 | The Lubinus patellofemoral arthroplasty. A five- to ten-year prospective study | Tauro B, Ackroyd CE, Newman JH, Shah NA. | J Bone Joint Surg Br | 2001 | 10.1302/0301-620x.83b5.11577 | English |
| 23376989 | Incidence of symptomatic thromboembolic disease after patellofemoral arthroplasty | Levack A, Kamath AF, Lonner JH. | Am J Orthop (Belle Mead NJ) | 2012 |  | English |
| 3409568 | Blood loss with total knee arthroplasty | Berman AT, Geissele AE, Bosacco SJ. | Clin Orthop Relat Res | 1988 | 10.1097/00003086-198809000-00024 | English |
| 26231494 | A 21% conversion rate to total knee arthroplasty of a first-generation patellofemoral prosthesis at a mean follow-up of 9.7 years | Hoogervorst P, de Jong RJ, Hannink G, van Kampen A. | Int Orthop | 2015 | 10.1007/s00264-015-2941-1 | English |
| 19897372 | Unusual mechanical complications of unicompartmental low contact stress mobile bearing patellofemoral arthroplasty: a cause for concern? | Arumilli BR, Ng AB, Ellis DJ, Hirst P. | Knee | 2010 | 10.1016/j.knee.2009.10.006 | English |
| 14503763 | Patellofemoral replacement: the third compartment | Argenson JN. | Orthopedics | 2003 | 10.3928/0147-7447-20030901-32 | English |
| 29893799 | Estimating an Individual's Probability of Revision Surgery After Knee Replacement: A Comparison of Modeling Approaches Using a National Data Set | Aram P, Trela-Larsen L, Sayers A, Hills AF, Blom AW, McCloskey EV, Kadirkamanathan V, Wilkinson JM. | Am J Epidemiol | 2018 | 10.1093/aje/kwy121 | English |
| 16006129 | Patellar clunk syndrome in patellofemoral arthroplasty--a case report | Sringari T, Maheswaran SS. | Knee | 2005 | 10.1016/j.knee.2004.11.008 | English |
| 7867096 | [Long-term follow-up of patients with patella resurfacing] | Shen J, Ye Q, Li S. | Zhongguo Yi Xue Ke Xue Yuan Xue Bao | 1994 |  | Chinese |
| 19829980 | Dissociation of mobile-bearing patellar component in low contact stress patellofemoral arthroplasty, its mechanism and management: two case reports | van Jonbergen HP, Werkman DM, Barnaart AF. | Cases J | 2009 | 10.1186/1757-1626-2-7502 | English |
| 32147094 | Erratum to "The midterm results of a cohort study of patellofemoral arthroplasty from a non-designer center using an asymmetric trochlear prosthesis" [Knee 26 (2019) 1348-1353] | Rammohan R, Gupta S, Lee PYF, Chandratreya A. | Knee | 2020 | 10.1016/j.knee.2020.02.009 | English |
| 24519181 | Autologous chondrocyte implantation and anteromedialization for isolated patellar articular cartilage lesions: 5- to 11-year follow-up | Gillogly SD, Arnold RM. | Am J Sports Med | 2014 | 10.1177/0363546513519077 | English |
| 29299472 | Does a simple syringe applicator enhance bone cement set up time in knee arthroplasty? | Sodhi N, Dalton SE, Khlopas A, Sultan AA, Curtis GL, Harb MA, Naziri Q, Newman JM, Barrington JW, Mont MA. | Ann Transl Med | 2017 | 10.21037/atm.2017.11.17 | English |
| 36527357 | Short term clinical outcomes of a Prochondrix® thin laser-etched osteochondral allograft for the treatment of articular cartilage defects in the knee | Mehta VM, Mehta S, Santoro S, Shriver R, Mandala C, Weess C. | J Orthop Surg (Hong Kong) | 2022 | 10.1177/10225536221141781 | English |
| 35315804 | Patellofemoral Arthroplasty Results in Better Time-weighted Patient-reported Outcomes After 6 Years than TKA: A Randomized Controlled Trial | Odgaard A, Kappel A, Madsen F, Kristensen PW, Stephensen S, Attarzadeh AP. | Clin Orthop Relat Res | 2022 | 10.1097/CORR.0000000000002178 | English |
| 31135543 | The Dubousset Functional Test is a Novel Assessment of Physical Function and Balance | Diebo BG, Challier V, Shah NV, Kim D, Murray DP, Kelly JJ, Lafage R, Paulino CB, Passias PG, Schwab FJ, Lafage V. | Clin Orthop Relat Res | 2019 | 10.1097/CORR.0000000000000820 | English |
| 26047754 | Is knee function better with contemporary modular bicompartmental arthroplasty compared to total knee arthroplasty? Short-term outcomes of a prospective matched study including 68 cases | Parratte S, Ollivier M, Opsomer G, Lunebourg A, Argenson JN, Thienpont E. | Orthop Traumatol Surg Res | 2015 | 10.1016/j.otsr.2015.03.019 | English |
| 12905899 | [Preliminary investigation on the pathogeny, diagnosis and treatment of chondromalacia patella] | Ye QB, Wu ZH, Wang YP, Lin J, Qiu GX. | Zhongguo Yi Xue Ke Xue Yuan Xue Bao | 2001 |  | Chinese |
| 36743281 | Combined MPFL Reconstruction with Tibial Tubercle Osteotomy and Repair of Patellar Cartilage Defect with Particulated Juvenile Articular Cartilage | Dennis ER, Marmor WA, Shubin Stein BE. | JBJS Essent Surg Tech | 2022 | 10.2106/JBJS.ST.21.00013 | English |
| 29529622 | The Mark Coventry Award: Patellofemoral Arthroplasty Results in Better Range of Movement and Early Patient-reported Outcomes Than TKA | Odgaard A, Madsen F, Kristensen PW, Kappel A, Fabrin J. | Clin Orthop Relat Res | 2018 | 10.1007/s11999.0000000000000017 | English |
| 3665248 | Pathomechanics of the femoropatellar joint following total knee arthroplasty | Hofmann GO, Hagena FW. | Clin Orthop Relat Res | 1987 |  | English |
| 11922380 | The Lubinus patellofemoral arthroplasty: a five- to ten-year prospective study | Roach R. | J Bone Joint Surg Br | 2002 |  | English |
| 28623415 | A stand-alone lateral condyle-elevating trochlear osteotomy leads to high residual instability but no excessive increase in patellofemoral osteoarthritis at 12-year follow-up | Tigchelaar S, van Sambeeck J, Koeter S, van Kampen A. | Knee Surg Sports Traumatol Arthrosc | 2018 | 10.1007/s00167-017-4602-y | English |
| 17592686 | [Treatment of patellofemoral arthritis with patello-femoral arthroplasties] | Jørgensen PS, Konradsen LA, Mati WB, Tørholm C. | Ugeskr Laeger | 2007 |  | Danish |
| 10427798 | [Femoro-patellar prosthesis. A retrospective study of 45 consecutive cases with a follow-up of 3-12 years] | De Cloedt P, Legaye J, Lokietek W. | Acta Orthop Belg | 1999 |  | French |
| 3967411 | Prospective analysis of Sheehan total knee arthroplasty | Callihan SM, Halley DK. | Clin Orthop Relat Res | 1985 |  | English |
| 1420832 | [Morphological and numerical studies of the stress compatibility of patella implants] | Lengsfeld M, Weiss H, Kienapfel H. | Biomed Tech (Berl) | 1992 | 10.1515/bmte.1992.37.10.222 | German |
| 19384616 | Tissue sparing surgery in knee reconstruction: unicompartmental (UKA), patellofemoral (PFA), UKA + PFA, bi-unicompartmental (Bi-UKA) arthroplasties | N Confalonieri, A Manzotti, F Montironi, C Pullen | J Orthop Traumatol | 2008 | 10.1007/s10195-008-0015-5 | English |

| **Table S3 Detailed bibliographies searched from BIOSIS Citation Index in the field of patellofemoral arthroplasty** | | | | | |
| --- | --- | --- | --- | --- | --- |
| **Article Title** | **Authors** | **Journal** | **Publication Year** | **DOI** | **Language** |
| The mark coventry award: Patellofemoral arthroplasty results in better range of movement and early patient-reported outcomes than TKA | Odgaard A.; Madsen F.; Kristensen P.W.; Kappel A.; Fabrin J. | Clinical Orthopaedics and Related Research | 2018 | 10.1007/s11999.0000000000000017 | English |
| Onlay Patellofemoral Arthroplasty in Patients With Isolated Patellofemoral Arthritis: A Systematic Review | Villa J.C.; Paoli A.R.; Nelson-Williams H.W.; Badr R.N.; Harper K.D. | Journal of Arthroplasty | 2021 | 10.1016/j.arth.2021.02.054 | English |
| Correction of Patellofemoral Malalignment With Patellofemoral Arthroplasty | Valoroso M.; Saffarini M.; La Barbera G.; Toanen C.; Hannink G.; Nover L.; Dejour D.H. | Journal of Arthroplasty | 2017 | 10.1016/j.arth.2017.06.048 | English |
| Short term clinical outcomes of a Prochondrix® thin laser-etched osteochondral allograft for the treatment of articular cartilage defects in the knee | Mehta V.M.; Mehta S.; Santoro S.; Shriver R.; Mandala C.; Weess C. | Journal of Orthopaedic Surgery | 2022 | 10.1177/10225536221141781 | English |
| Treatment of unicompartmental cartilage defects of the knee with unicompartmental knee arthroplasty, patellofemoral partial knee arthroplasty or focal resurfacing | Springer B.; Boettner F. | Life | 2021 | 10.3390/life11050394 | English |
| Failure modes of patellofemoral arthroplasty—registries vs. clinical studies: a systematic review | Bendixen N.B.; Eskelund P.W.; Odgaard A. | Acta Orthopaedica | 2019 | 10.1080/17453674.2019.1634865 | English |
| Combined patellofemoral arthroplasty and medial patellofemoral ligament reconstruction for chronic patellar instability with trochlear dysplasia: a report of two cases | Yamagami R.; Inui H.; Taketomi S.; Tanaka S. | Modern Rheumatology Case Reports | 2020 | 10.1080/24725625.2019.1638048 | English |
| Classification of combined partial knee arthroplasty | Garner A.; Van Arkel R.J.; Cobb J. | Bone and Joint Journal | 2019 | 10.1302/0301-620X.101B8.BJJ-2019-0125.R1 | English |
| No bias for developer publications and no difference between first-generation trochlear-resurfacing versus trochlear-cutting implants in 15,306 cases of patellofemoral joint arthroplasty | Reihs B.; Reihs F.; Labek G.; Hochegger M.; Leithner A.; Böhler N.; Sadoghi P. | Knee Surgery, Sports Traumatology, Arthroscopy | 2018 | 10.1007/s00167-017-4692-6 | English |
| Early post-operative opioid consumption: A comparison between medial unicompartmental, patellofemoral, and total knee arthroplasty | Melnic C.M.; Kazarian E.R.; Dwyer M.K.; Domingo-Johnson E.L.; Freiberg A.A.; Bedair H.S. | Knee | 2019 | 10.1016/j.knee.2019.06.016 | English |
| Patellofemoral arthroplasty versus total knee arthroplasty for isolated patellofemoral osteoarthritis: a systematic review and meta-analysis | Peng G.; Liu M.; Guan Z.; Hou Y.; Liu Q.; Sun X.; Zhu X.; Feng W.; Zeng J.; Zhong Z.; Zeng Y. | Journal of Orthopaedic Surgery and Research | 2021 | 10.1186/s13018-021-02414-5 | English |
| Patellar tendon shortening following patellofemoral joint replacement | van Engen L.A.H.; Landman E.B.M.; Kleinlugtenbelt Y.V.; van Jonbergen H.-P.W. | International Orthopaedics | 2019 | 10.1007/s00264-018-4194-2 | English |
| Rotational alignment of the femoral trochlea in Asians: Implication on implant choice and position for managing isolated patellofemoral osteoarthritis | Li M.K.L.; Wan S.Y.-C.; Lo K.C.H.; Hung Y.W.; Fan J.C.H. | Journal of Orthopaedics, Trauma and Rehabilitation | 2022 | 10.1177/22104917221085718 | English |
| Use of Natural Language Processing Algorithms to Identify Common Data Elements in Operative Notes for Knee Arthroplasty | Sagheb E.; Ramazanian T.; Tafti A.P.; Fu S.; Kremers W.K.; Berry D.J.; Lewallen D.G.; Sohn S.; Maradit Kremers H. | Journal of Arthroplasty | 2021 | 10.1016/j.arth.2020.09.029 | English |
| The midterm results of a cohort study of patellofemoral arthroplasty from a non-designer centre using an asymmetric trochlear prosthesis | R. R.; Gupta S.; Lee P.Y.F.; Chandratreya A. | Knee | 2019 | 10.1016/j.knee.2019.10.026 | English |
| Survivorship and functional outcomes of patellofemoral arthroplasty: a systematic review | van der List J.P.; Chawla H.; Zuiderbaan H.A.; Pearle A.D. | Knee Surgery, Sports Traumatology, Arthroscopy | 2017 | 10.1007/s00167-015-3878-z | English |
| Patellofemoral arthroplasty: obesity linked to high risk of revision and progression of medial tibiofemoral osteoarthritis | Marullo M.; Bargagliotti M.; Vigano’ M.; Lacagnina C.; Romagnoli S. | Knee Surgery, Sports Traumatology, Arthroscopy | 2022 | 10.1007/s00167-022-06947-y | English |
| Patient-related outcomes of patellofemoral arthroplasty: experience of a single center | Abeysekera W.Y.M.; Schenk W. | Arthroplasty | 2021 | 10.1186/s42836-021-00074-8 | English |
| Patellofemoral arthroplasty conversion to total knee arthroplasty: Retrieval analysis and clinical correlation | Christ A.B.; Baral E.; Koch C.; Shubin Stein B.E.; Gonzalez Della Valle A.; Strickland S.M. | Knee | 2017 | 10.1016/j.knee.2017.06.015 | English |
| In vivo magnetic resonance imaging morphometry of the patella bone in South Indian population | Muhamed R.; Saralaya V.V.; Murlimanju B.V.; Chettiar G.K. | Anatomy and Cell Biology | 2017 | 10.5115/acb.2017.50.2.99 | English |
| Joint Awareness after Patellofemoral Arthroplasty Evaluated with the Forgotten Joint Score: A Comparison Study | Lin W.; Dai Y.; Dong C.; Piao K.; Hao K.; Wang F. | Orthopaedic Surgery | 2021 | 10.1111/os.12921 | English |
| Patellofemoral Arthroplasty: Short-Term Complications and Risk Factors | Rezzadeh K.; Behery O.A.; Kester B.S.; Dogra T.; Vigdorchik J.; Schwarzkopf R. | Journal of Knee Surgery | 2020 | 10.1055/s-0039-1688960 | English |
| A stand-alone lateral condyle-elevating trochlear osteotomy leads to high residual instability but no excessive increase in patellofemoral osteoarthritis at 12-year follow-up | Tigchelaar S.; van Sambeeck J.; Koeter S.; van Kampen A. | Knee Surgery, Sports Traumatology, Arthroscopy | 2018 | 10.1007/s00167-017-4602-y | English |
| Medical interventions for patellofemoral pain and patellofemoral osteoarthritis: A systematic review | Macri E.M.; Hart H.F.; Thwaites D.; Barton C.J.; Crossley K.M.; Bierma-Zeinstra S.M.A.; van Middelkoop M. | Journal of Clinical Medicine | 2020 | 10.3390/jcm9113397 | English |
| Patient satisfaction reporting for patellofemoral arthroplasty is significantly lacking: a systematic review | Tishelman J.C.; Kahlenberg C.A.; Nwachukwu B.U.; Gruskay J.; Strickland S.M. | Physician and Sportsmedicine | 2019 | 10.1080/00913847.2019.1580913 | English |
| Patellofemoral Arthroplasty Results in Better Time-weighted Patient-reported Outcomes after 6 Years than TKA: A Randomized Controlled Trial | Odgaard A.; Kappel A.; Madsen F.; Kristensen P.W.; Stephensen S.; Attarzadeh A.P. | Clinical Orthopaedics and Related Research | 2022 | 10.1097/CORR.0000000000002178 | English |
| Smoking, unemployment, female sex, obesity, and medication use yield worse outcomes in patellofemoral arthroplasty | Desai V.S.; Pareek A.; DeDeugd C.M.; Sabbag O.D.; Krych A.J.; Cummings N.M.; Dahm D.L. | Knee Surgery, Sports Traumatology, Arthroscopy | 2020 | 10.1007/s00167-019-05704-y | English |
| Clinical outcome of bi-unicompartmental knee arthroplasty for both medial and lateral femorotibial arthritis: a systematic review—is there proof of concept? | Wada K.; Price A.; Gromov K.; Lustig S.; Troelsen A. | Archives of Orthopaedic and Trauma Surgery | 2020 | 10.1007/s00402-020-03492-6 | English |
| Femoropatellar osteoarthritis and trochlear femoral bone defect due to giant cell tumor of the knee: A selected patellofemoral joint arthroplasty and reconstructive technique a case report | Conti A.; Boffano M.; Pellegrino P.; Ratto N.; Sabatini L.; Piana R. | JBJS Case Connector | 2020 | 10.2106/JBJS.CC.19.00378 | English |
| The Dubousset Functional Test is a Novel Assessment of Physical Function and Balance | Diebo B.G.; Challier V.; Shah N.V.; Kim D.; Murray D.P.; Kelly J.J.; Lafage R.; Paulino C.B.; Passias P.G.; Schwab F.J.; Lafage V. | Clinical Orthopaedics and Related Research | 2019 | 10.1097/CORR.0000000000000820 | English |
| The influence of malalignment and ageing following sterilisation by gamma irradiation in an inert atmosphere on the wear of ultra-high-molecular-weight polyethylene in patellofemoral replacements | Maiti R.; Cowie R.M.; Fisher J.; Jennings L.M. | Proceedings of the Institution of Mechanical Engineers, Part H: Journal of Engineering in Medicine | 2017 | 10.1177/0954411917696112 | English |
| Effectiveness of an Accelerated Rehabilitation Protocol After Tibial Tubercle Osteotomy | Morgan C.; Bell R.M.; Burland J.P.; Kriscenski D.; Ilinski A.; Cote M.P.; Edgar C.M. | Orthopaedic Journal of Sports Medicine | 2022 | 10.1177/23259671221133105 | English |
| Patellofemoral Replacement With Tibial Tubercle Osteotomy | Arvesen J.E.; Wyland D.J. | Arthroscopy Techniques | 2021 | 10.1016/j.eats.2020.09.009 | English |
| Tibial Osteolysis After Long-Term Isolated Polyethylene Patellar Resurfacing | Apple A.E.; Montgomery C.O.; Mears S.C. | Arthroplasty Today | 2021 | 10.1016/j.artd.2021.03.012 | English |
| No Major Functional Benefit After Bicompartmental Knee Arthroplasty Compared to Total Knee Arthroplasty at 5-Year Follow-Up | Schrednitzki D.; Beier A.; Marx A.; Halder A.M. | Journal of Arthroplasty | 2020 | 10.1016/j.arth.2020.07.003 | English |
| Obesity Does Not Affect Patient-Reported Outcomes following Patellofemoral Arthroplasty | Tishelman J.C.; Pyne A.; Kahlenberg C.A.; Gruskay J.A.; Strickland S.M. | Journal of Knee Surgery | 2022 | 10.1055/s-0040-1713862 | English |
| Recurrent patellar dislocations with patellar cartilage defects: A pain in the knee? | Dalal S.; Setia P.; Debnath A.; Guro R.; Kotwal R.; Chandratreya A. | Knee | 2021 | 10.1016/j.knee.2021.01.019 | English |
| Total knee arthroplasty reduces knee extension torque in-vitro and patellofemoral arthroplasty does not | Joseph M.N.; Carmont M.R.; Tailor H.; Stephen J.M.; Amis A.A. | Journal of Biomechanics | 2020 | 10.1016/j.jbiomech.2020.109739 | English |
| Patterns of cartilage loss and anterior cruciate ligament status in end-stage osteoarthritis of the knee: Assessing suitability for partial knee arthroplasty | Scott C.E.H.; Holland G.; Krahelski O.; Murray I.R.; Keating J.F.; Keenan O.J.F. | Bone and Joint Journal | 2020 | 10.1302/0301-620X.102B6.BJJ-2019-1434.R1 | English |
| Mid-term survivorship and patient-reported outcomes of robotic-arm assisted partial knee arthroplasty: A SINGLE-SURGEON STUDY of 1,018 KNEES | Burger J.A.; Kleeblad L.J.; Laas N.; Pearle A.D. | Bone and Joint Journal | 2020 | 10.1302/0301-620X.102B1.BJJ-2019-0510.R1 | English |
| Early outcomes of an anatomic trochlear-cutting patellofemoral arthroplasty: patient selection is key | Dejour D.; Saffarini M.; Malemo Y.; Pungitore M.; Valluy J.; Nover L.; Demey G. | Knee Surgery, Sports Traumatology, Arthroscopy | 2019 | 10.1007/s00167-019-05368-8 | English |
| The present situation of patellofemoral arthroplasty in the management of solitary patellofemoral osteoarthritis | Rodriguez-Merchan E.C. | Archives of Bone and Joint Surgery | 2020 | 10.22038/ABJS.2019.14125 | English |
| Midterm results of modern patellofemoral arthroplasty versus total knee arthroplasty for isolated patellofemoral arthritis: systematic review and meta-analysis of comparative studies | Elbardesy H.; McLeod A.; Gul R.; Harty J. | Archives of Orthopaedic and Trauma Surgery | 2022 | 10.1007/s00402-021-03882-4 | English |
| Cost effectiveness of patellofemoral versus total knee arthroplasty in younger patients | Chawla H.; Nwachukwu B.U.; Van Der List J.P.; Eggman A.A.; Pearle A.D.; Ghomrawi H.M. | Bone and Joint Journal | 2017 | 10.1302/0301-620X.99B8.BJJ-2016-1032.R1 | English |
| Prospective Outcomes of Cryopreserved Osteochondral Allograft for Patellofemoral Cartilage Defects at Minimum 2-Year Follow-up | Melugin H.P.; Ridley T.J.; Bernard C.D.; Wischmeier D.; Farr J.; Stuart M.J.; Macalena J.A.; Krych A.J. | Cartilage | 2021 | 10.1177/1947603520903420 | English |
| Knee arthroplasty utilization trends from 2010 to 2019 | Fuller S.I.; Cohen J.S.; Malyavko A.; Agarwal A.R.; Stake S.; Golladay G.J.; Thakkar S.C. | Knee | 2022 | 10.1016/j.knee.2022.09.006 | English |
| Surgical technique in patellofemoral arthroplasty | Remy F. | Orthopaedics and Traumatology: Surgery and Research | 2019 | 10.1016/j.otsr.2018.05.020 | English |
| Mid-Term Clinical, Functional, and Radiographic Outcomes of 105 Gender-Specific Patellofemoral Arthroplasties, With or Without the Association of Medial Unicompartmental Knee Arthroplasty | Romagnoli S.; Marullo M. | Journal of Arthroplasty | 2018 | 10.1016/j.arth.2017.10.019 | English |
| Combined MPFL Reconstruction with Tibial Tubercle Osteotomy and Repair of Patellar Cartilage Defect with Particulated Juvenile Articular Cartilage | Dennis E.R.; Marmor W.A.; Shubin Stein B.E. | JBJS Essential Surgical Techniques | 2022 | 10.2106/JBJS.ST.21.00013 | English |
| [Morphological and numerical studies of the stress compatibility of patella implants] | Lengsfeld M, Weiss H, Kienapfel H. | Biomed Tech (Berl) | 1992 | 10.1515/bmte.1992.37.10.222 | German |
| A Newly Identified Complication of Patellofemoral Arthroplasty: Case Report and Literature Review | Solarino G.; Maccagnano G.; Vicenti G.; Buono C.; Simone F.; Ottaviani G.; Zavattini G.; Zaccari D.; Carrozzo M.; Spinarelli A.; Bizzoca D.; Moretti B. | Geriatric Orthopaedic Surgery and Rehabilitation | 2022 | 10.1177/21514593221138662 | English |
| Unicompartmental vs. segmental bicompartmental vs. total knee replacement: comparison of clinical outcomes | Al-Dadah O.; Hawes G.; Chapman-Sheath P.J.; Tice J.W.; Barrett D.S. | Knee Surgery and Related Research | 2020 | 10.1186/s43019-020-00065-0 | English |
| Isolated Patellofemoral Joint Arthroplasty: Can Preoperative Bone Scans Predict Survivorship? | Baker J.F.; Caborn D.N.; Schlierf T.J.; Fain T.B.; Smith L.S.; Malkani A.L. | Journal of Arthroplasty | 2020 | 10.1016/j.arth.2019.08.021 | English |
| Augmented reality in robotic assisted orthopaedic surgery: A pilot study | Iqbal H.; Tatti F.; Rodriguez y Baena F. | Journal of Biomedical Informatics | 2021 | 10.1016/j.jbi.2021.103841 | English |
| Patellar button compatibility in the conversion of Patellofemoral Arthroplasty to a Total Knee Arthroplasty: A review of the contemporary literature | McDonald L.K.; Kurmis A.P. | Journal of Orthopaedic Surgery | 2022 | 10.1177/10225536221084147 | English |
| Patellofemoral arthroplasty versus total knee arthroplasty for patients with patellofemoral osteoarthritis EQUAL FUNCTION AND SATISFACTION BUT HIGHER REVISION RATE FOR PARTIAL ARTHROPLASTY AT A MINIMUM EIGHT YEARS’ FOLLOW-UP | Clement N.D.; Howard T.A.; Immelman R.J.; MacDonald D.; Patton J.T.; Lawson G.M.; Burnett R. | Bone and Joint Journal | 2019 | 10.1302/0301-620X.101B1.BJJ-2018-0654.R2 | English |
| Replacement for patellofemoral arthritis | Johnson D.S.; Turner P.G. | Knee | 2019 | 10.1016/j.knee.2019.10.016 | English |
| The patient results and satisfaction of knee arthroplasty in a validated grading system | Oosthuizen C.R.; Van Der Straeten C.; Maposa I.; Snyckers C.H.; Vermaak D.P.; Magobotha S. | International Orthopaedics | 2019 | 10.1007/s00264-019-04412-z | English |
| [Patellofemoral arthroplasty: indication, technique and results] | Cotic M, Imhoff AB. | Orthopade | 2014 | 10.1007/s00132-014-3006-7 | German |
| Patellofemoral arthroplasty in combination with high tibial osteotomy can achieve good outcome for patients with medial-patellofemoral osteoarthritis | Peng Y.; Lin W.; Zhang Y.; Wang F. | Frontiers in Surgery | 2022 | 10.3389/fsurg.2022.999208 | English |
| Mid-to long-term follow-up of combined small implants: A third-generation patellofemoral arthroplasty associated with a medial or lateral unicompartmental knee arthroplasty | Rossi S.M.P.; Perticarini L.; Clocchiatti S.; Ghiara M.; Benazzo F. | Bone and Joint Journal | 2021 | 10.1302/0301-620X.103B5.BJJ-2020-0720.R3 | English |
| Does a simple syringe applicator enhance bone cement set up time in knee arthroplasty? | Sodhi N.; Dalton S.E.; Khlopas A.; Sultan A.A.; Curtis G.L.; Harb M.A.; Naziri Q.; Newman J.M.; Barrington J.W.; Mont M.A. | Annals of Translational Medicine | 2017 | 10.21037/atm.2017.11.17 | English |
| Similar postoperative patient-reported outcome in both second generation patellofemoral arthroplasty and total knee arthroplasty for treatment of isolated patellofemoral osteoarthritis: a systematic review | Bunyoz K.I.; Lustig S.; Troelsen A. | Knee Surgery, Sports Traumatology, Arthroscopy | 2019 | 10.1007/s00167-018-5151-8 | English |
| The Clinical Outcome of Patellofemoral Arthroplasty vs Total Knee Arthroplasty in Patients Younger Than 55 Years | Kamikovski I.; Dobransky J.; Dervin G.F. | Journal of Arthroplasty | 2019 | 10.1016/j.arth.2019.07.016 | English |
| Preoperative patellofemoral anatomy affects failure rate after isolated patellofemoral inlay arthroplasty | Feucht M.J.; Lutz P.M.; Ketzer C.; Rupp M.C.; Cotic M.; Imhoff A.B.; Pogorzelski J. | Archives of Orthopaedic and Trauma Surgery | 2020 | 10.1007/s00402-020-03651-9 | English |
| Inadequacy of computed tomography for pre-operative planning of patellofemoral arthroplasty | Saffarini M.; Müller J.H.; La Barbera G.; Hannink G.; Cho K.J.; Toanen C.; Dejour D. | Knee Surgery, Sports Traumatology, Arthroscopy | 2018 | 10.1007/s00167-017-4474-1 | English |
| Modelling and simulation of alternative designs for the femur–implant interface of Journey patellofemoral prosthesis | Castro A.P.G.; Completo A.; Simões J.A.; Flores P. | Proceedings of the Institution of Mechanical Engineers, Part L: Journal of Materials: Design and Applications | 2019 | 10.1177/1464420718774074 | English |
| Estimating an Individual's Probability of Revision Surgery after Knee Replacement: A Comparison of Modeling Approaches Using a National Data Set | Aram P.; Trela-Larsen L.; Sayers A.; Hills A.F.; Blom A.W.; McCloskey E.V.; Kadirkamanathan V.; Wilkinson J.M. | American Journal of Epidemiology | 2018 | 10.1093/aje/kwy121 | English |
| Has the modern design of Attune total knee replacement improved outcome in patients with isolated patellofemoral arthritis? | Ashraf Y.; Iqbal H.J.; Senevirathana S.; Ashraf T. | Journal of Orthopaedic Surgery | 2020 | 10.1177/2309499020975553 | English |
| Advances in Patellofemoral Arthroplasty | Strickland S.M.; Bird M.L.; Christ A.B. | Current Reviews in Musculoskeletal Medicine | 2018 | 10.1007/s12178-018-9477-0 | English |
| Minimally Invasive Robotic-Assisted Patellofemoral Arthroplasty | Hassebrock J.D.; Makovicka J.L.; Wong M.; Patel K.A.; Scott K.L.; Deckey D.G.; Chhabra A. | Arthroscopy Techniques | 2020 | 10.1016/j.eats.2019.11.013 | English |
| Pre-operative patella alta does not affect midterm clinical outcomes and survivorship of patellofemoral arthroplasty | Bernard C.D.; Pareek A.; Sabbag C.M.; Parkes C.W.; Krych A.J.; Cummings N.M.; Dahm D.L. | Knee Surgery, Sports Traumatology, Arthroscopy | 2021 | 10.1007/s00167-020-06205-z | English |
| Arthroscopic Debridement, Facetectomy, and Synovectomy for Isolated Patellofemoral Osteoarthritis | Zhao J. | Arthroscopy Techniques | 2021 | 10.1016/j.eats.2021.08.021 | English |
| Comparing Return to Sports After Patellofemoral and Knee Arthroplasty in an Age- and Sex-Matched Cohort | Schneider B.L.; Ling D.I.; Kleebad L.J.; Strickland S.; Pearle A. | Orthopaedic Journal of Sports Medicine | 2020 | 10.1177/2325967120957425 | English |
| Derotational osteotomy at the distal femur is effective to treat patients with patellar instability | Imhoff F.B.; Cotic M.; Liska F.; Dyrna F.G.E.; Beitzel K.; Imhoff A.B.; Herbst E. | Knee Surgery, Sports Traumatology, Arthroscopy | 2019 | 10.1007/s00167-018-5212-z | English |
| Patellofemoral Arthroplasty Improves Patellofemoral Alignment in Patients with Patellofemoral Osteoarthritis with Trochlear Dysplasia | Yang G.; Wang J.; Dai Y.; Lin W.; Niu J.; Wang F. | Journal of Knee Surgery | 2022 | 10.1055/s-0040-1715099 | English |
| Patellofemoral joint replacement — Mean five year follow-up | Ajnin S.; Buchanan D.; Arbuthnot J.; Fernandes R. | Knee | 2018 | 10.1016/j.knee.2018.08.014 | English |
| Return to the operating room after patellofemoral arthroplasty versus total knee arthroplasty for isolated patellofemoral arthritis—a systematic review | Woon C.Y.L.; Christ A.B.; Goto R.; Shanaghan K.; Shubin Stein B.E.; Gonzalez Della Valle A. | International Orthopaedics | 2019 | 10.1007/s00264-018-04280-z | English |
| Pin Site Complications Associated With Computer-Assisted Navigation in Hip and Knee Arthroplasty | Kamara E.; Berliner Z.P.; Hepinstall M.S.; Cooper H.J. | Journal of Arthroplasty | 2017 | 10.1016/j.arth.2017.03.073 | English |
| Aiming for anatomical femoral axis on the coronal plane leads to good-to-excellent short-term outcomes in isolated patellofemoral arthroplasty | Vasta S.; Rosi M.; Tecame A.; Papalia R.; Adravanti P. | Knee | 2020 | 10.1016/j.knee.2020.02.016 | English |
| Mid-term survivorship and clinical outcomes of the Avon patellofemoral joint replacement | Middleton S.W.F.; Toms A.D.; Schranz P.J.; Mandalia V.I. | Knee | 2018 | 10.1016/j.knee.2018.01.007 | English |
| Femoral component rotation in patellofemoral joint replacement | van Jonbergen H.-P.W.; Westerbeek R.E. | Knee | 2018 | 10.1016/j.knee.2018.02.007 | English |
| Patellofemoral arthroplasty is cheaper and more effective in the short term than total knee arthroplasty for isolated patellofemoral osteoarthritis: Cost-effectiveness analysis based on a randomized trial | Fredborg C.; Odgaard A.; Sørensen J. | Bone and Joint Journal | 2020 | 10.1302/0301-620X.102B4.BJJ-2018-1580.R3 | English |
| Patellofemoral arthroplasty | Godshaw B.; Kolodychuk N.; Williams G.K., Jr.; Browning B.; Jones D. | Ochsner Journal | 2018 | 10.31486/toj.18.0009 | English |
| The short-term effectiveness and safety of second-generation patellofemoral arthroplasty and total knee arthroplasty on isolated patellofemoral osteoarthritis: a systematic review and meta-analysis | Li C.; Li Z.; Shi L.; Gao F.; Sun W. | Journal of Orthopaedic Surgery and Research | 2021 | 10.1186/s13018-021-02509-z | English |
| Patellofemoral arthroplasty: Current concepts | Cuthbert R.; Tibrewal S.; Tibrewal S.B. | Journal of Clinical Orthopaedics and Trauma | 2018 | 10.1016/j.jcot.2017.11.006 | English |
| Effect of Patellofemoral Arthroplasty on Patellar Height in Patients with Patellofemoral Osteoarthritis | Lee H.; Fletcher C.; Hartwell M.; Strickland S.M. | Journal of Knee Surgery | 2021 | 10.1055/s-0042-1755354 | English |
| The Combination of Inlay Patellofemoral Arthroplasty and Medial Unicompartmental Knee Arthroplasty Versus Total Knee Arthroplasty for Mediopatellofemoral Osteoarthritis: A Comparison of Mid-Term Outcomes | Uluyardimci E.; Isik C.; Tahta M.; Emre F.; Cepni S.; Oltulu I. | Journal of Arthroplasty | 2019 | 10.1016/j.arth.2019.06.043 | English |
| Patellofemoral Arthroplasty Surgical Technique: Lateral or Medial Parapatellar Approach | Jeong S.H.; Schneider B.; Pyne A.S.; Tishelman J.C.; Strickland S.M. | Journal of Arthroplasty | 2020 | 10.1016/j.arth.2020.04.026 | English |
| [Patellofemoral arthroplasty] | Cotic M, Forkel P, Imhoff AB. | Oper Orthop Traumatol | 2017 | 10.1007/s00064-016-0477-1 | German |
| Insufficient evidence to confirm benefits of custom partial knee arthroplasty: a systematic review | Demey G.; Müller J.H.; Liebensteiner M.; Pilot P.; Nover L.; Saffarini M.; Arnold M.P.; Beckmann J.; Kort N. | Knee Surgery, Sports Traumatology, Arthroscopy | 2022 | 10.1007/s00167-021-06766-7 | English |
| A matched-pair comparison of inlay and onlay trochlear designs for patellofemoral arthroplasty: no differences in clinical outcome but less progression of osteoarthritis with inlay designs | Feucht M.J.; Cotic M.; Beitzel K.; Baldini J.F.; Meidinger G.; Schöttle P.B.; Imhoff A.B. | Knee Surgery, Sports Traumatology, Arthroscopy | 2017 | 10.1007/s00167-015-3733-2 | English |
| The PAT randomized clinical trial | Joseph M.N.; Achten J.; Parsons N.R.; Costa M.L. | Bone and Joint Journal | 2020 | 10.1302/0301-620X.102B3.BJJ-2019-0723.R1 | English |
| Hermes patellofemoral arthroplasty: Annual revision rate and clinical results after two to 20 years of follow-up | Bohu Y.; Klouche S.; Sezer H.B.; Gerometta A.; Lefevre N.; Herman S. | Knee | 2019 | 10.1016/j.knee.2019.01.014 | English |
| Patellofemoral arthroplasty: expert opinion | Hoogervorst P.; Arendt E.A. | Journal of Experimental Orthopaedics | 2022 | 10.1186/s40634-022-00457-z | English |
| A high level of satisfaction after bicompartmental individualized knee arthroplasty with patient-specific implants and instruments | Ogura T.; Le K.; Merkely G.; Bryant T.; Minas T. | Knee Surgery, Sports Traumatology, Arthroscopy | 2019 | 10.1007/s00167-018-5155-4 | English |
| Robotic Assisted Patellofemoral Joint Replacement: Surgical Technique, Tips and Tricks | Selvaratnam V.; Toms A.D.; Mandalia V.I. | Indian Journal of Orthopaedics | 2022 | 10.1007/s43465-022-00746-w | English |
| Return to Activities After Patellofemoral Arthroplasty | Shubin Stein B.E.; Brady J.M.; Grawe B.; Tuakli-Wosornu Y.; Nguyen J.T.; Wolfe E.; Voigt M.; Mahony G.; Strickland S. | American journal of orthopedics (Belle Mead, N.J.) | 2017 |  | English |
| Patellofemoral Arthroplasty | Odgaard A.; Eldridge J.; Madsen F. | JBJS Essential Surgical Techniques | 2019 | 10.2106/JBJS.ST.18.00094 | English |
| Patellofemoral arthroplasty in a bilateral Syme's amputee | Kanna R.; Barrett D.S. | Journal of Clinical Orthopaedics and Trauma | 2016 | 10.1016/j.jcot.2015.09.004 | English |
| When nothing else works: patellofemoral joint arthroplasty | Benignus, C; Meier, M; Best, R; Beckmann, J | SPORTVERLETZUNG-SPORTSCHADEN | 2021 | 10.1055/a-1523-9937 | German |
| Are Revisions of Patellofemoral Arthroplasties More Like Primary or Revision TKAs | Parratte S.; Lunebourg A.; Ollivier M.; Abdel M.P.; Argenson J.-N.A. | Clinical Orthopaedics and Related Research | 2015 | 10.1007/s11999-014-3756-x | English |
| Patellofemoral arthroplasty. | Lonner J.H. | Orthopedics | 2010 |  | English |
| Medium term results of Avon patellofemoral joint replacement | Sarda P.K.; Shetty A.; Maheswaran S.S. | Indian Journal of Orthopaedics | 2011 | 10.4103/0019-5413.83761 | English |
| Evidence of trochlear dysplasia in patellofemoral arthroplasty designs | Saffarini M.; Ntagiopoulos P.G.; Demey G.; Le Negaret B.; Dejour D.H. | Knee Surgery, Sports Traumatology, Arthroscopy | 2014 | 10.1007/s00167-014-2967-8 | English |
| Complications in patellofemoral surgery | Tompkins M.; Arendt E.A. | Sports Medicine and Arthroscopy Review | 2012 | 10.1097/JSA.0b013e31825c74cf | English |
| Prospective clinical and radiological two-year results after patellofemoral arthroplasty using an implant with an asymmetric trochlea design | Beitzel K.; Schöttle P.B.; Cotic M.; Dharmesh V.; Imhoff A.B. | Knee Surgery, Sports Traumatology, Arthroscopy | 2013 | 10.1007/s00167-012-2022-6 | English |
| A 21 % conversion rate to total knee arthroplasty of a first-generation patellofemoral prosthesis at a mean follow-up of 9.7 years | Hoogervorst P.; de Jong R.J.; Hannink G.; van Kampen A. | International Orthopaedics | 2015 | 10.1007/s00264-015-2941-1 | English |
| Patellofemoral arthroplasty. | Lonner J.H. | Instructional course lectures | 2010 |  | English |
| Bi-unicompartmental and combined uni plus patellofemoral replacement: Indications and surgical technique | Romagnoli S.; Marullo M.; Massaro M.; Rustemi E.; D'Amario F.; Corbella M. | Joints | 2015 | 10.11138/jts/2015.3.1.042 | English |
| Patellofemoral joint replacement, an evolving concept | Borus T.; Brilhault J.; Confalonieri N.; Johnson D.; Thienpont E. | Knee | 2014 | 10.1016/S0968-0160(14)50010-5 | English |
| The effect of axial rotation of the anterior resection plane in patellofemoral arthroplasty | Cho K.J.; Erasmus P.J.; Müller J.H. | Knee | 2016 | 10.1016/j.knee.2016.04.006 | English |
| Mobile-bearing, congruent patellofemoral prosthesis: short-term results | Yadav B.; Shaw D.; Radcliffe G.; Dachepalli S.; Kluge W. | Journal of orthopaedic surgery (Hong Kong) | 2012 | 10.1177/230949901202000317 | English |
| Patellofemoral arthroplasty influences tibiofemoral kinematics: the effect of patellar thickness | Vandenneucker H.; Labey L.; Victor J.; Vander Sloten J.; Desloovere K.; Bellemans J. | Knee Surgery, Sports Traumatology, Arthroscopy | 2014 | 10.1007/s00167-014-3160-9 | English |
| Early results of patellofemoral inlay resurfacing arthroplasty using the HemiCap wave prosthesis | Patel A.; Haider Z.; Anand A.; Spicer D. | Journal of Orthopaedic Surgery | 2017 | 10.1177/2309499017692705 | English |
| Scientific evidence for the use of modern patellofemoral arthroplasty | Gupta R.R.; Zywiel M.G.; Leadbetter W.B.; Bonutti P.; Mont M.A. | Expert Review of Medical Devices | 2010 | 10.1586/erd.09.53 | English |
| Distal femoral bone mineral density decreases following patellofemoral arthroplasty: 1-year follow-up study of 14 patients | Van Jonbergen H.-P.W.; Koster K.; Labey L.; Innocenti B.; Van Kampen A. | BMC Musculoskeletal Disorders | 2010 | 10.1186/1471-2474-11-74 | English |
| Computer-Assisted Navigation in Patellofemoral Arthroplasty: A New Technique to Improve Rotational Position of the Trochlea | Hernigou P.; Flouzat-Lachaniette C.H.; Delblond W.; Duffiet P.; Julian D. | HSS Journal | 2013 | 10.1007/s11420-013-9328-x | English |
| Blood Loss and Transfusion Rates Following Patellofemoral Arthroplasty | Courtney J.; Liebelt D.; Nett M.P.; Cushner F.D. | Orthopedic Clinics of North America | 2012 | 10.1016/j.ocl.2012.07.007 | English |
| Patellofemoral arthroplasty: the other unicompartmental knee replacement. | Walker T.; Perkinson B.; Mihalko W.M. | Instructional course lectures | 2013 |  | English |
| Why do patellofemoral arthroplasties fail today? A systematic review | van der List J.P.; Chawla H.; Villa J.C.; Pearle A.D. | Knee | 2017 | 10.1016/j.knee.2015.11.002 | English |
| Biomechanical behaviour of cancellous bone on patellofemoral arthroplasty with Journey prosthesis: a finite element study | Castro A.P.G.; Completo A.; Simões J.A.; Flores P. | Computer Methods in Biomechanics and Biomedical Engineering | 2015 | 10.1080/10255842.2013.870999 | English |
| Patellofemoral arthroplasty: A multi-centre study with minimum 2-year follow-up | Leadbetter W.B.; Kolisek F.R.; Levitt R.L.; Brooker A.F.; Zietz P.; Marker D.R.; Bonutti P.M.; Mont M.A. | International Orthopaedics | 2009 | 10.1007/s00264-008-0692-y | English |
| Patellofemoral arthroplasty | Lustig S. | Orthopaedics and Traumatology: Surgery and Research | 2014 | 10.1016/j.otsr.2013.06.013 | English |
| Autologous chondrocyte implantation and anteromedialization for isolated patellar articular cartilage lesions: 5- to 11-year follow-up | Gillogly S.D.; Arnold R.M. | American Journal of Sports Medicine | 2014 | 10.1177/0363546513519077 | English |
| Annual revision rates of partial versus total knee arthroplasty: A comparative meta-analysis | Chawla H.; van der List J.P.; Christ A.B.; Sobrero M.R.; Zuiderbaan H.A.; Pearle A.D. | Knee | 2017 | 10.1016/j.knee.2016.11.006 | English |
| Comparison of commercial patellofemoral arthroplasty systems on the basis of patella kinematics, peri-patellar soft tissue tension and prosthesis design | Müller J.H.; Erasmus P.J.; Scheffer C. | Journal of Mechanics in Medicine and Biology | 2012 | 10.1142/S0219519412500868 | English |
| The Avon patellofemoral joint replacement: Five-year results from an independent centre | Odumenya M.; Costa M.L.; Parsons N.; Achten J.; Dhillon M.; Krikler S.J. | Journal of Bone and Joint Surgery - Series B | 2010 | 10.1302/0301-620X.92B1.23135 | English |
| Functional relevance of patellofemoral thickness before and after unicompartmental patellofemoral replacement | Mofidi A.; Bajada S.; Holt M.D.; Davies A.P. | Knee | 2012 | 10.1016/j.knee.2011.03.002 | English |
| Outcomes of Total Knee Replacement after Patellofemoral Arthroplasty | Hutt J.; Dodd M.; Bourke H.; Bell J. | Journal of Knee Surgery | 2012 | 10.1055/s-0032-1329233 | English |
| Patellofemoral arthroplasty changes the trochlear groove angle | Wiesner F.J.; Erasmus P.J.; Cho K.J.; Müller J.H. | Journal of Mechanics in Medicine and Biology | 2017 | 10.1142/S0219519417500683 | English |
| UKA in combination with PFR at average 12-year follow-up | Heyse T.J.; Khefacha A.; Cartier P. | Archives of Orthopaedic and Trauma Surgery | 2010 | 10.1007/s00402-009-0997-3 | English |
| Patellofemoral joint arthroplasty: Early results and functional outcome of the Zimmer gender solutions patello-femoral joint system | Osarumwense D.; Syed F.; Nzeako O.; Akilapa S.; Zubair O.; Waite J. | CiOS Clinics in Orthopedic Surgery | 2017 | 10.4055/cios.2017.9.3.295 | English |
| The clinical outcome of patellofemoral arthroplasty | Lonner J.H.; Bloomfield M.R. | Orthopedic Clinics of North America | 2013 | 10.1016/j.ocl.2013.03.002 | English |
| Patellofemoral osteoarthritis treated by partial lateral facetectomy: Results at long-term follow up | Wetzels T.; Bellemans J. | Knee | 2012 | 10.1016/j.knee.2011.04.005 | English |
| A preliminary report of patellofemoral arthroplasty in isolated patellofemoral arthritis | Gao X.; Xu Z.-J.; He R.-X.; Yan S.-G.; Wu L.-D. | Chinese Medical Journal | 2010 | 10.3760/cma.j.issn.0366-6999.2010.21.013 | English |
| Patellofemoral arthritis Plea for a customized therapy | Keshmiri, A; Imhoff, AB; Dirisamer, F | ARTHROSKOPIE | 2020 | 10.1007/s00142-020-00379-x | German |
| Strain shielding in distal femur after patellofemoral arthroplasty under different activity conditions | Meireles S.; Completo A.; António Simões J.; Flores P. | Journal of Biomechanics | 2010 | 10.1016/j.jbiomech.2009.09.048 | English |
| Obesity and the absence of trochlear dysplasia increase the risk of revision in patellofemoral arthroplasty | Liow M.H.L.; Goh G.S.H.; Tay D.K.J.; Chia S.-L.; Lo N.-N.; Yeo S.-J. | Knee | 2016 | 10.1016/j.knee.2015.05.009 | English |
| The basic science of the patella: structure, composition, and function. | Fox A.J.; Wanivenhaus F.; Rodeo S.A. | The journal of knee surgery | 2012 | 10.1055/s-0032-1313741 | English |
| Knee osteoarthritis: A review of management options | Hussain S.M.; Neilly D.W.; Baliga S.; Patil S.; Meek R.M.D. | Scottish Medical Journal | 2016 | 10.1177/0036933015619588 | English |
| Are “patellofemoral symptoms” truly related to the patellofemoral joint? | Yassa R.; Khalfaoui M.Y.; Davies A.P. | Knee Surgery and Related Research | 2016 | 10.5792/ksrr.2016.28.1.68 | English |
| Biomechanics of medial unicondylar in combination with patellofemoral knee arthroplasty | Heyse T.J.; El-Zayat B.F.; De Corte R.; Scheys L.; Chevalier Y.; Fuchs-Winkelmann S.; Labey L. | Knee | 2014 | 10.1016/S0968-0160(14)50002-6 | English |
| MRI after patellofemoral replacement: The preserved compartments | Heyse T.J.; Figiel J.; Hähnlein U.; Timmesfeld N.; Lakemeier S.; Schofer M.D.; Fuchs-Winkelmann S.; Efe T. | European Journal of Radiology | 2012 | 10.1016/j.ejrad.2011.06.012 | English |
| Four-Year Follow Up Outcome Study of Patellofemoral Arthroplasty at a Single Institution | Goh G.S.H.; Liow M.H.L.; Tay D.K.J.; Lo N.N.; Yeo S.J. | Journal of Arthroplasty | 2015 | 10.1016/j.arth.2015.01.020 | English |
| Patellofemoral arthroplasty in the athlete | Farr J.; Arendt E.; Dahm D.; Daynes J. | Clinics in Sports Medicine | 2014 | 10.1016/j.csm.2014.03.003 | English |
| Advanced patellofemoral cartilage lesions in patients younger than 50 years of age: Is there an ideal operative option? | Noyes F.R.; Barber-Westin S.D. | Arthroscopy - Journal of Arthroscopic and Related Surgery | 2013 | 10.1016/j.arthro.2013.03.077 | English |
| Long-term results of compartmental arthroplasties of the knee: Long term results of partial knee arthroplasty | Parratte S.; Ollivier M.; Lunebourg A.; Abdel M.P.; Argenson J.-N. | Bone and Joint Journal | 2015 | 10.1302/0301-620X.97B10.36426 | English |
| Association and impact of patellofemoral dysplasia on patellofemoral arthropathy and arthroplasty | Mofidi A.; Veravalli K.; Jinnah R.H.; Poehling G.G. | Knee | 2014 | 10.1016/j.knee.2013.09.009 | English |
| The Journey patellofemoral joint arthroplasty: A minimum 5 year follow-up study | Ahearn N.; Metcalfe A.J.; Hassaballa M.A.; Porteous A.J.; Robinson J.R.; Murray J.R.; Newman J.H. | Knee | 2016 | 10.1016/j.knee.2016.03.004 | English |
| Patellofemoral arthroplasty versus total knee arthroplasty in patients with isolated patellofemoral osteoarthritis. | Dahm D.L.; Al-Rayashi W.; Dajani K.; Shah J.P.; Levy B.A.; Stuart M.J. | American journal of orthopedics (Belle Mead, N.J.) | 2010 |  | English |
| Patellofemoral joint replacement | Van Jonbergen H.P.W.; Van Lingen C.P. | Minerva Ortopedica e Traumatologica | 2010 |  | English |
| Patellofemoral joint arthroplasty: Our experience in isolated patellofemoral and bicompartmental arthritic knees | Sabatini L.; Schirò M.; Atzori F.; Ferrero G.; Massè A. | Clinical Medicine Insights: Arthritis and Musculoskeletal Disorders | 2016 | 10.4137/CMAMD.S40498 | English |
| Complications after patello-femoral versus total knee replacement in the treatment of isolated patello-femoral osteoarthritis. A meta-analysis | Dy C.J.; Franco N.; Ma Y.; Mazumdar M.; McCarthy M.M.; Gonzalez Della Valle A. | Knee Surgery, Sports Traumatology, Arthroscopy | 2012 | 10.1007/s00167-011-1677-8 | English |
| Isolated patellofemoral arthroplasty reproduces natural patellofemoral joint kinematics when the patella is resurfaced | Vandenneucker H.; Labey L.; Vander Sloten J.; Desloovere K.; Bellemans J. | Knee Surgery, Sports Traumatology, Arthroscopy | 2016 | 10.1007/s00167-014-3415-5 | English |
| Design, operative technique and ten-year results of the Hermes™ patellofemoral arthroplasty | Philippe H.; Caton J. | International Orthopaedics | 2014 | 10.1007/s00264-013-2158-0 | English |
| Mid-term results of the FPV patellofemoral joint replacement | Al-Hadithy N.; Patel R.; Navadgi B.; Deo S.; Hollinghurst D.; Satish V. | Knee | 2014 | 10.1016/j.knee.2013.08.010 | English |
| MRI After Patellofemoral Replacement: The Component-Bone Interface and Rotational Alignment | Heyse T.J.; Figiel J.; Hähnlein U.; Timmesfeld N.; Schofer M.D.; Fuchs-Winkelmann S.; Efe T. | HSS Journal | 2013 | 10.1007/s11420-013-9336-x | English |
| Trochlear Inclination Angles in Normal and Dysplastic Knees | Kamath A.F.; Slattery T.R.; Levack A.E.; Wu C.H.; Kneeland J.B.; Lonner J.H. | Journal of Arthroplasty | 2013 | 10.1016/j.arth.2012.04.017 | English |
| Patella component loosening - A case report | Bloemheuvel E.M.; Van Rooij W.M.J.; Van Den Besselaar M. | Acta Orthopaedica Belgica | 2016 |  | English |
| The low contact stress patellofemoral replacement : High early failure rate | Charalambous C.P.; Abiddin Z.; Mills S.P.; Rogers S.; Sutton P.; Parkinson R. | Journal of Bone and Joint Surgery - Series B | 2011 | 10.1302/0301-620X.93B4.25899 | English |
| Femoro Patella Vialla patellofemoral arthroplasty: An independent assessment of outcomes at minimum 2-year follow-up | Halai M.; Ker A.; Anthony I.; Holt G.; Jones B.; Blyth M. | World Journal of Orthopedics | 2016 | 10.5312/wjo.v7.i8.487 | English |
| Medial patellofemoral ligament reconstruction for subluxating patellofemoral arthroplasty | Carmont M.R.; Crane T.; Thompson P.; Spalding T. | Knee | 2011 | 10.1016/j.knee.2010.02.011 | English |
| Patellofemoral arthroplasty, where are we today? | Lustig S.; Magnussen R.A.; Dahm D.L.; Parker D. | Knee Surgery, Sports Traumatology, Arthroscopy | 2012 | 10.1007/s00167-012-1948-z | English |
| Differences in the stress distribution in the distal femur between patellofemoral joint replacement and total knee replacement: A finite element study | van Jonbergen H.-P.W.; Innocenti B.; Gervasi G.L.; Labey L.; Verdonschot N. | Journal of Orthopaedic Surgery and Research | 2012 | 10.1186/1749-799X-7-28 | English |
| Early revisions of the Femoro-Patella Vialla joint replacement | Williams D.P.; Pandit H.G.; Athanasou N.A.; Murray D.W.; Gibbons C.L.M.H. | Bone and Joint Journal | 2013 | 10.1302/0301-620X.95B6.31355 | English |
| Arthroplasty of the Femoropatellar Joint - What Data are Available? | Fink, B; Schwenninger, C | ZEITSCHRIFT FUR ORTHOPADIE UND UNFALLCHIRURGIE | 2014 | 10.1055/s-0033-1360353 | German |
| Patellofemoral Arthroplasty. 7-year Mean Follow-Up. | Mont M.A.; Johnson A.J.; Naziri Q.; Kolisek F.R.; Leadbetter W.B. | Journal of Arthroplasty | 2012 | 10.1016/j.arth.2011.07.010 | English |
| Patellectomy for osteoarthritis: A new tension preserving surgical technique to reconstruct the extensor mechanism with retrospective review of long-term follow-up | Asopa V.; Willis-Owen C.; Keene G. | Journal of Orthopaedic Surgery and Research | 2015 | 10.1186/s13018-015-0237-1 | English |
| Surgical Treatment of Isolated Patellofemoral Osteoarthritis | Rodriguez-Merchan E.C. | HSS Journal | 2014 | 10.1007/s11420-013-9375-3 | English |
| Patellofemoral replacement: the third compartment | Hofmann A.A.; McCandless J.B.; Shaeffer J.F.; Magee T.H. | The bone & joint journal | 2013 | 10.1302/0301-620X.95B11.32985 | English |
| Significant Functional Improvement at 2 Years After Isolated Patellofemoral Arthroplasty With an Onlay Trochlear Implant, But Low Mental Health Scores Predispose to Dissatisfaction | Kazarian G.S.; Tarity T.D.; Hansen E.N.; Cai J.; Lonner J.H. | Journal of Arthroplasty | 2016 | 10.1016/j.arth.2015.08.033 | English |
| Patellar Fracture Following Patellofemoral Arthroplasty | King A.H.; Engasser W.M.; Sousa P.L.; Arendt E.A.; Dahm D.L. | Journal of Arthroplasty | 2015 | 10.1016/j.arth.2015.02.007 | English |
| Unusual mechanical complications of unicompartmental low contact stress mobile bearing patellofemoral arthroplasty: A cause for concern? | Arumilli B.R.B.; Ng A.B.Y.; Ellis D.J.; Hirst P. | Knee | 2010 | 10.1016/j.knee.2009.10.006 | English |
| Clinical results of patellofemoral arthroplasty | Morris M.J.; Lombardi A.V.; Berend K.R.; Hurst J.M.; Adams J.B. | Journal of Arthroplasty | 2013 | 10.1016/j.arth.2013.05.012 | English |
| Outcome of patellofemoral arthroplasty, determinants for success | Willekens P.; Victor J.; Verbruggen D.; Kerckhove M.V.; Van Der Straeten C. | Acta Orthopaedica Belgica | 2015 |  | English |
| The Warwick patellofemoral arthroplasty trial: A randomised clinical trial of total knee arthroplasty versus patellofemoral arthroplasty in patients with severe arthritis of the patellofemoral joint | Odumenya M.; McGuinness K.; Achten J.; Parsons N.; Spalding T.; Costa M. | BMC Musculoskeletal Disorders | 2011 | 10.1186/1471-2474-12-265 | English |
| Patellofemoral arthroplasty: outcomes and factors associated with early progression of tibiofemoral arthritis | Dahm D.L.; Kalisvaart M.M.; Stuart M.J.; Slettedahl S.W. | Knee Surgery, Sports Traumatology, Arthroscopy | 2014 | 10.1007/s00167-014-3202-3 | English |
| The correct rotation of the femoral component in patellofemoral replacement: A laboratory assessment of a surgical technique | Clark D.A.; Upadhyay N.; Gillespie G.; Wakeley C.; Eldridge J.D. | Journal of Bone and Joint Surgery - Series B | 2012 | 10.1302/0301-620X.94B12.29506 | English |
| Outcomes of Patellofemoral Arthroplasty Based on Radiographic Severity | deDeugd C.M.; Pareek A.; Krych A.J.; Cummings N.M.; Dahm D.L. | Journal of Arthroplasty | 2017 | 10.1016/j.arth.2016.11.006 | English |
| Update on Patellofemoral Arthroplasty | Crowe M.M.; Dahm D.L. | Operative Techniques in Sports Medicine | 2015 | 10.1053/j.otsm.2015.05.001 | English |
| In vivo sagittal plane kinematics of the FPV patellofemoral replacement | Monk A.P.; van Duren B.H.; Pandit H.; Shakespeare D.; Murray D.W.; Gill H.S. | Knee Surgery, Sports Traumatology, Arthroscopy | 2012 | 10.1007/s00167-011-1717-4 | English |
| Long-term outcomes of patellofemoral arthroplasty | van Jonbergen H.-P.W.; Werkman D.M.; Barnaart L.F.; van Kampen A. | Journal of Arthroplasty | 2010 | 10.1016/j.arth.2009.08.023 | English |
| Patellar polyethylene spinout after low-contact stress, high-congruity, mobile-bearing patellofemoral arthroplasty | Amanatullah D.F.; Jamali A.A. | Orthopedics | 2012 | 10.3928/01477447-20120123-27 | English |
| Incidence of symptomatic thromboembolic disease after patellofemoral arthroplasty | Levack A.; Kamath A.F.; Lonner J.H. | American journal of orthopedics (Belle Mead, N.J.) | 2012 |  | English |
| Revision cartilage cell transplantation for failed autologous chondrocyte transplantation in chronic osteochondral defects of the knee | Vijayan S.; Bentley G.; Rahman J.; Briggs T.W.R.; Skinner J.A.; Carrington R.W.J. | Bone and Joint Journal | 2014 | 10.1302/0301-620X.96B1.31979 | English |
| Midterm Outcome of Avon Patellofemoral Arthroplasty for Posttraumatic Unicompartmental Osteoarthritis | Konan S.; Haddad F.S. | Journal of Arthroplasty | 2016 | 10.1016/j.arth.2016.06.005 | English |
| Results of a French multicentre retrospective experience with four hundred and eighteen failed unicondylar knee arthroplasties | Saragaglia D.; Bonnin M.; Dejour D.; Deschamps G.; Chol C.; Chabert B.; Refaie R. | International Orthopaedics | 2013 | 10.1007/s00264-013-1915-4 | English |
| Coronal alignment of patellofemoral arthroplasty | Thienpont E.; Lonner J.H. | Knee | 2014 | 10.1016/S0968-0160(14)50011-7 | English |
| Partial knee arthroplasty: Patellofemoral arthroplasty and combined unicompartmental and patellofemoral arthroplasty implants - general considerations and indications, technique and clinical experience | Benazzo F.; Rossi S.M.P.; Ghiara M. | Knee | 2014 | 10.1016/S0968-0160(14)50009-9 | English |
| Isolated patellofemoral osteoarthritis: A systematic review of treatment options using the GRADE approach | Van Jonbergen H.-P.W.; Poolman R.W.; Van Kampen A. | Acta Orthopaedica | 2010 | 10.3109/17453671003628756 | English |
| Return to work following knee arthroplasty | Foote J.A.J.; Smith H.K.; Jonas S.C.; Greenwood R.; Weale A.E. | Knee | 2010 | 10.1016/j.knee.2009.06.001 | English |
| Isolated patellofemoral arthroplasty | Oni J.K.; Hochfelder J.; Dayan A. | Bulletin of the Hospital for Joint Diseases | 2014 |  | English |
| Patellofemoral arthroplasty for symptomatic nonunion after trochlear osteotomy for patellar instability: A case report | Van Jonbergen H.-P.W.; Van Egmond K. | Cases Journal | 2009 | 10.1186/1757-1626-2-9086 | English |
| Patellofemoral replacement. | Blazina M.E.; Fox J.M.; Del Pizzo W.; Broukhim B.; Ivey F.M. | Clinical orthopaedics and related research | 1979 | 10.1097/00003086-197910000-00017 | English |
| Medium-term results of patellofemoral joint arthroplasty | Mohammed R.; Jimulia T.; Durve K.; Bansal M.; Green M.; Learmonth D. | Acta Orthopaedica Belgica | 2008 |  | English |
| Custom patellofemoral arthroplasty of the knee | Sisto D.J.; Sarin V.K. | Journal of Bone and Joint Surgery | 2006 | 10.2106/JBJS.E.00382 | English |
| The Avon patellofemoral joint replacement: Independent assessment of early functional outcomes | Starks I.; Roberts S.; White S.H. | Journal of Bone and Joint Surgery - Series B | 2009 | 10.1302/0301-620X.91B12.23018 | English |
| Conversion of patellofemoral arthroplasty to total knee arthroplasty: A matched case-control study of 13 patients | Van Jonbergen H.-P.W.; Werkman D.M.; Kampen A.V. | Acta Orthopaedica | 2009 | 10.1080/17453670902805031 | English |
| Patellofemoral Arthroplasty with a Customized Trochlear Prosthesis | Sisto D.J.; Sarin V.K. | Orthopedic Clinics of North America | 2008 | 10.1016/j.ocl.2008.03.002 | English |
| Long-term results of patellofemoral arthroplasty | Kooijman H.J.; Driessen A.P.P.M.; van Horn J.R. | Journal of Bone and Joint Surgery - Series B | 2003 | 10.1302/0301-620x.85b6.13741 | English |
| Patellofemoral arthroplasty: 2–12-year follow-up study | Cartier P. | Journal of Arthroplasty | 1990 | 10.1016/S0883-5403(06)80009-4 | English |
| [Patellofemoral replacement for severe patellofemoral osteoarthritis: a 2-10 years follow-up study] | Zhang J, Ye QB, Qiu GX, Wang YP. | Zhongguo Yi Xue Ke Xue Yuan Xue Bao | 2002 |  | Chinese |
| In Vivo Sagittal Plane Kinematics of the Avon Patellofemoral Arthroplasty | Hollinghurst D.; Stoney J.; Ward T.; Pandit H.; Beard D.; Murray D.W. | Journal of Arthroplasty | 2007 | 10.1016/j.arth.2006.02.160 | English |
| Dislocation of the mobile bearing component of a patellofemoral arthroplasty : A report of two cases | Witjes S.; Van Den Broek C.; Koëter S.; Van Loon C. | Acta Orthopaedica Belgica | 2009 |  | English |
| Patellofemoral resurfacing arthroplasty: literature review and description of a novel technique. | Cannon A.; Stolley M.; Wolf B.; Amendola A. | The Iowa orthopaedic journal | 2008 |  | English |
| Patellofemoral arthroplasty with a custom-fit femoral prosthesis | Butler J.E.; Shannon R. | Orthopedics | 2009 |  | English |
| Patellofemoral arthroplasty | Lonner J.H. | Journal of the American Academy of Orthopaedic Surgeons | 2007 | 10.5435/00124635-200708000-00006 | English |
| The Lubinus patellofemoral arthroplasty | Tauro B.; Ackroyd C.E.; Newman J.H.; Shah N.A. | Journal of Bone and Joint Surgery - Series B | 2001 | 10.1302/0301-620X.83B5.11577 | English |
| Early results with a total patellofemoral joint replacement arthroplasty prosthesis | Merchant A.C. | Journal of Arthroplasty | 2004 | 10.1016/j.arth.2004.03.011 | English |
| Arthritis progression after patellofemoral joint replacement | Nicol S.G.; Loveridge J.M.; Weale A.E.; Ackroyd C.E.; Newman J.H. | Knee | 2006 | 10.1016/j.knee.2006.04.005 | English |
| Controversies and techniques in the surgical management of patellofemoral arthritis. | Mihalko W.M.; Boachie-Adjei Y.; Spang J.T.; Fulkerson J.P.; Arendt E.A.; Saleh K.J. | Instructional course lectures | 2008 |  | English |
| Anterior knee pain: diagnosis and treatment. | Post W.R. | The Journal of the American Academy of Orthopaedic Surgeons | 2005 | 10.5435/00124635-200512000-00006 | English |
| Patellofemoral Replacement: The Third Compartment | Hofmann A.A.; Clark C.D.; Ponder C.; Hoffman M. | Seminars in Arthroplasty JSES | 2009 | 10.1053/j.sart.2008.11.014 | English |
| Modular bicompartmental knee arthroplasty with robotic arm assistance | Lonner J.H. | American journal of orthopedics (Belle Mead, N.J.) | 2009 |  | English |
| The Richards type II patellofemoral arthroplasty: 26 Cases followed for 1-20 years | De Winter W.E.A.E.; Feith R.; Van Loon C.J.M. | Acta Orthopaedica Scandinavica | 2001 | 10.1080/000164701753532826 | English |
| Patellofemoral arthrosis: the treatment options | Oberlander M.A.; Baker C.L.; Morgan B.E. | American journal of orthopedics (Belle Mead, N.J.) | 1998 |  | English |
| The Lubinus patellofemoral arthroplasty: A series of 17 cases | Board T.N.; Mahmood A.; Ryan W.G.; Banks A.J. | Archives of Orthopaedic and Trauma Surgery | 2004 | 10.1007/s00402-004-0645-x | English |
| The avon patellofemoral arthroplasty: Five-year survivorship and functional results | Ackroyd C.E.; Newman J.H.; Evans R.; Edridge J.D.J.; Joslin C.C. | Journal of Bone and Joint Surgery - Series B | 2007 | 10.1302/0301-620X.89B3.18062 | English |
| Midterm clinical results of the Autocentric II patellofemoral prosthesis | Van Wagenberg J.M.F.; Speigner B.; Gosens T.; De Waal Malefijt J. | International Orthopaedics | 2009 | 10.1007/s00264-009-0719-z | English |
| Manipulation under anaesthesia for stiffness following knee arthroplasty | Mohammed R.; Syed S.; Ahmed N. | Annals of the Royal College of Surgeons of England | 2009 | 10.1308/003588409X359321 | English |
| Patellar button dissociation in a mobile-bearing LCS patellofemoral joint arthroplasty. | Sreekumar R.; Subramanian S.; Mohammed A. | The journal of knee surgery | 2009 | 10.1055/s-0030-1247763 | English |
| Habitual patellar subluxation | Cameron H.U. | Orthopaedic Review | 1985 |  | English |
| [Preliminary investigation on the pathogeny, diagnosis and treatment of chondromalacia patella] | Ye QB, Wu ZH, Wang YP, Lin J, Qiu GX. | Zhongguo Yi Xue Ke Xue Yuan Xue Bao | 2001 |  | Chinese |
| Patellar clunk syndrome in patellofemoral arthroplasty - A case report | Sringari T.; Maheswaran S.S. | Knee | 2005 | 10.1016/j.knee.2004.11.008 | English |
| Unicompartmental knee arthroplasty with patelloplasty | Antoniou J.; Hadjipavlou A.; Enker P.; Antoniou A. | International Orthopaedics | 1996 | 10.1007/s002640050038 | English |
| Ipsilateral Patellofemoral Arthroplasty and Autogenous Osteochondral Femoral Condylar Transplantation | Lonner J.H.; Mehta S.; Booth Jr. R.E. | Journal of Arthroplasty | 2007 | 10.1016/j.arth.2005.08.012 | English |
| Functional outcomes after different types of knee arthroplasty: Kneeling ability versus descending stairs | Hassaballa M.A.; Porteous A.J.; Learmonth I.D. | Medical Science Monitor | 2007 |  | English |
| Patellofemoral Arthroplasty in the Treatment of Patellofemoral Arthritis: Rationale and Outcomes in Younger Patients | Leadbetter W.B. | Orthopedic Clinics of North America | 2008 | 10.1016/j.ocl.2008.04.001 | English |
| Results of Total Knee Replacement for Isolated Patellofemoral Arthritis: When Not to Perform a Patellofemoral Arthroplasty | Delanois R.E.; McGrath M.S.; Ulrich S.D.; Marker D.R.; Seyler T.M.; Bonutti P.M.; Mont M.A. | Orthopedic Clinics of North America | 2008 | 10.1016/j.ocl.2008.03.003 | English |
| Revision of a failed patellofemoral arthroplasty to a total knee arthroplasty | Lonner J.H.; Jasko J.G.; Booth R.E., Jr. | Journal of Bone and Joint Surgery | 2006 | 10.2106/JBJS.F.00282 | English |
| Optimizing patellofemoral arthroplasty | Farr II J.; Barrett D. | Knee | 2008 | 10.1016/j.knee.2008.05.008 | English |
| Unique combination of patellofemoral joint arthroplasty with Osteochondral Autograft Transfer System (OATS) - A case series of six knees in five patients | Unnithan A.; Jimulia T.; Mohammed R.; Learmonth D.J.A. | Knee | 2008 | 10.1016/j.knee.2008.01.007 | English |
| Blood loss with total knee arthroplasty | Berman A.T.; Geissele A.E.; Bosacco S.J. | Clinical Orthopaedics and Related Research | 1988 | 10.1097/00003086-198809000-00024 | English |
| MBARS: mini bone-attached robotic system for joint arthroplasty | Wolf A.; Jaramaz B.; Lisien B.; DiGioia A.M. | International Journal of Medical Robotics and Computer Assisted Surgery | 2005 | 10.1002/rcs.20 | English |
| Is anterior knee pain a predisposing factor to patellofemoral osteoarthritis? | Utting M.R.; Davies G.; Newman J.H. | Knee | 2005 | 10.1016/j.knee.2004.12.006 | English |
| Computer-Assisted Patellofemoral Arthroplasty. A Mechanism for Optimizing Rotation | Cossey A.J.; Spriggins A.J. | Journal of Arthroplasty | 2006 | 10.1016/j.arth.2005.08.010 | English |
| Patellofemoral Arthroplasty: The Impact of Design on Outcomes | Lonner J.H. | Orthopedic Clinics of North America | 2008 | 10.1016/j.ocl.2008.02.002 | English |
| Revision Patellofemoral Arthroplasty. Three- to Seven-Year Follow-Up | Hendrix M.R.G.; Ackroyd C.E.; Lonner J.H. | Journal of Arthroplasty | 2008 | 10.1016/j.arth.2007.10.019 | English |
| Operative management of patellofemoral pain with degenerative arthrosis | Herrenbruck T.M.; Mullen D.J.; Parker R.D. | Sports Medicine and Arthroscopy Review | 2001 | 10.1097/00132585-200110000-00009 | English |
| Treatment of patello-femoral arthritis using the Lubinus patello-femoral arthroplasty: A retrospective review | Smith A.M.; Peckett W.R.C.; Butler-Manuel P.A.; Venu K.M.; D'Arcy J.C. | Knee | 2002 | 10.1016/S0968-0160(01)00127-2 | English |
| Patient-based outcomes in patellofemoral arthroplasty. | Utukuri M.M.; Khanduja V.; Somayaji H.S.; Dowd G.S. | The journal of knee surgery | 2008 | 10.1055/s-0030-1247830 | English |
| HyBAR: Hybrid bone-attached robot for joint arthroplasty | Song S.; Mor A.; Jaramaz B. | International Journal of Medical Robotics and Computer Assisted Surgery | 2009 | 10.1002/rcs.254 | English |
| Patellofemoral arthroplasty. A three- to nine-year follow-up study | Arciero R.A.; Toomey H.E. | Clinical Orthopaedics and Related Research | 1988 |  | English |
| Patellofemoral arthroplasty: A 2- to 18-year followup study | Krajca-Radcliffe J.B.; Coker T.P. | Clinical Orthopaedics and Related Research | 1996 | 10.1097/00003086-199609000-00017 | English |
| Surgery for Osteoarthritis of the Knee | Richmond J.C. | Rheumatic Disease Clinics of North America | 2008 | 10.1016/j.rdc.2008.05.010 | English |
| Surgical options for patients with osteoarthritis of the knee | Lützner J.; Kasten P.; Günther K.-P.; Kirschner S. | Nature Reviews Rheumatology | 2009 | 10.1038/nrrheum.2009.88 | English |
| Surgery for Osteoarthritis of the Knee | Richmond J.C. | Medical Clinics of North America | 2009 | 10.1016/j.mcna.2008.09.012 | English |
| Pre-operative mental wellbeing and the outcome of knee replacement | Walton M.J.; Newman J.H. | Knee | 2008 | 10.1016/j.knee.2008.03.001 | English |
| Dissociation of mobile-bearing patellar component in low contact stress patellofemoral arthroplasty, its mechanism and management: Two case reports | Van Jonbergen H.-P.W.; Werkman D.M.; Barnaart A.F.W. | Cases Journal | 2009 | 10.1186/1757-1626-2-7502 | English |
| [Patellofemoral inlay implants-an innovation in patellofemoral joint arthroplasty?] | Degenhardt H, Imhoff AB, Feucht MJ, Pogorzelski J. | Orthopade | 2021 | 10.1007/s00132-020-04059-4 | German |

| **Table S4 Detailed bibliographies searched from Springer in the field of patellofemoral arthroplasty** | | | | | |
| --- | --- | --- | --- | --- | --- |
| **Article Title** | **Authors** | **Journal** | **Publication Year** | **DOI** | **Language** |
| Medium term results of Avon patellofemoral joint replacement | Dr. Praveen K. SardaAnup ShettyShanmuga S. Maheswaran | Indian Journal of Orthopaedics | 2011 | 10.4103/0019-5413.83761 | English |
| The Warwick patellofemoral arthroplasty trial: a randomised clinical trial of total knee arthroplasty versus patellofemoral arthroplasty in patients with severe arthritis of the patellofemoral joint | Michelle OdumenyaKatie McGuinnessJuul AchtenNick ParsonsTim SpaldingMatthew Costa | BMC Musculoskeletal Disorders | 2011 | 10.1186/1471-2474-12-265 | English |
| No bias for developer publications and no difference between first-generation trochlear-resurfacing versus trochlear-cutting implants in 15,306 cases of patellofemoral joint arthroplasty | Birgit Reihs, Florian Reihs, Gerold Labek, Markus Hochegger, Andreas Leithner, Nikolaus Böhler, Patrick Sadoghi | Knee Surgery, Sports Traumatology, Arthroscopy | 2018 | 10.1007/s00167-017-4692-6 | English |
| Posters |  | Knee Surgery, Sports Traumatology, Arthroscopy | 2012 | 10.1007/s00167-012-1933-6 | English |
| Differences in the stress distribution in the distal femur between patellofemoral joint replacement and total knee replacement: a finite element study | Hans-Peter W van JonbergenBernardo InnocentiGian Luca GervasiLuc LabeyNico Verdonschot | Journal of Orthopaedic Surgery and Research | 2012 | 10.1186/1749-799X-7-28 | English |
| Advances in Patellofemoral Arthroplasty | Sabrina M. StricklandMackenzie L. BirdAlexander B. Christ | Current Reviews in Musculoskeletal Medicine | 2018 | 10.1007/s12178-018-9477-0 | English |
| Dissociation of mobile-bearing patellar component in low contact stress patellofemoral arthroplasty, its mechanism and management: two case reports | Hans-Peter W van JonbergenDirk M WerkmanAlexander FW Barnaart | Cases Journal | 2009 | 10.1186/1757-1626-2-7502 | English |
| Robotic Assisted Patellofemoral Joint Replacement: Surgical Technique, Tips and Tricks | Veenesh SelvaratnamAndrew D. TomsVipul I. Mandalia | Indian Journal of Orthopaedics | 2022 | 10.1007/s43465-022-00746-w | English |
| Patellofemoral arthroplasty for symptomatic nonunion after trochlear osteotomy for patellar instability: a case report | Hans-Peter W van JonbergenKees van Egmond | Cases Journal | 2009 | 10.1186/1757-1626-2-9086 | English |
| Patellofemoral arthroplasty, where are we today? | Sébastien Lustig, Robert A. Magnussen, Diane L. Dahm & David Parker | Knee Surgery, Sports Traumatology, Arthroscopy | 2012 | 10.1007/s00167-012-1948-z | English |
| Patellar tendon shortening following patellofemoral joint replacement | Ludo A. H. van EngenEllie B. M. LandmanYdo V. KleinlugtenbeltHans-Peter W. van Jonbergen | International Orthopaedics | 2019 | 10.1007/s00264-018-4194-2 | English |
| Patella tracking and patella contact pressure in modular patellofemoral arthroplasty: a biomechanical in vitro analysis | Tilman CalliessMax EttingerSsuheib SchadoChristoph BecherChristof HurschlerSven Ostermeier | Archives of Orthopaedic and Trauma Surgery | 2016 | 10.1007/s00402-016-2451-7 | English |
| Midterm results of modern patellofemoral arthroplasty versus total knee arthroplasty for isolated patellofemoral arthritis: systematic review and meta-analysis of comparative studies | Hany Elbardesy, André McLeod, Rehan Gul & James Harty | Archives of Orthopaedic and Trauma Surgery | 2022 | 10.1007/s00402-021-03882-4 | English |
| Return to the operating room after patellofemoral arthroplasty versus total knee arthroplasty for isolated patellofemoral arthritis—a systematic review | Colin Y. L. WoonAlexander B. ChristRie GotoKate ShanaghanBeth E. Shubin SteinAlejandro Gonzalez Della Valle | International Orthopaedics | 2019 | 10.1007/s00264-018-04280-z | English |
| Pre-operative patella alta does not affect midterm clinical outcomes and survivorship of patellofemoral arthroplasty | Christopher D. BernardAyoosh PareekCasey M. SabbagChad W. ParkesAaron J. KrychNancy M. CummingsDiane L. Dahm | Knee Surgery, Sports Traumatology, Arthroscopy | 2021 | 10.1007/s00167-020-06205-z | English |
| Patellofemoral arthroplasty: obesity linked to high risk of revision and progression of medial tibiofemoral osteoarthritis | Matteo Marullo, Marco Bargagliotti, Marco Vigano’, Claudio Lacagnina & Sergio Romagnoli | Knee Surgery, Sports Traumatology, Arthroscopy | 2022 | 10.1007/s00167-022-06947-y | English |
| Design, operative technique and ten-year results of the Hermes?patellofemoral arthroplasty | Hernigou PhilippeJacques Caton | International Orthopaedics | 2014 | 10.1007/s00264-013-2158-0 | English |
| High mid-term revision rate after treatment of large, full-thickness cartilage lesions and OA in the patellofemoral joint using a large inlay resurfacing prosthesis: HemiCAP-Wave® | Jens Ole Laursen | Knee Surgery, Sports Traumatology, Arthroscopy | 2017 | 10.1007/s00167-016-4352-2 | English |
| Design, operative technique and ten-year results of the Hermes™ patellofemoral arthroplasty | Philippe HernigouJacques Caton | International Orthopaedics | 2014 | 10.1007/s00264-013-2158-0 | English |
| In vivo sagittal plane kinematics of the FPV patellofemoral replacement | A. P. MonkB. H. van DurenH. PanditD. ShakespeareD. W. MurrayH. S. Gill | Knee Surgery, Sports Traumatology, Arthroscopy | 2012 | 10.1007/s00167-011-1717-4 | English |
| MRI After Patellofemoral Replacement: the Component–Bone Interface and Rotational Alignment | Thomas J. Heyse MD, Jens Figiel MD, Ulrike Hähnlein, Nina Timmesfeld MSc, Markus D. Schofer MD, Susanne Fuchs-Winkelmann MD & Turgay Efe MD | HSS Journal | 2013 | 10.1007/s11420-013-9336-x | English |
| Posters |  | Knee Surgery, Sports Traumatology, Arthroscopy | 2008 | 10.1007/s00167-008-0548-4 | English |
| Isolated patellofemoral arthroplasty reproduces natural patellofemoral joint kinematics when the patella is resurfaced | Hilde VandenneuckerLuc LabeyJos Vander SlotenKaat DesloovereJohan Bellemans | Knee Surgery, Sports Traumatology, Arthroscopy | 2016 | 10.1007/s00167-014-3415-5 | English |
| Distal femoral bone mineral density decreases following patellofemoral arthroplasty: 1-year follow-up study of 14 patients | Hans-Peter W van JonbergenKenneth KosterLuc LabeyBernardo InnocentiAlbert van Kampen | BMC Musculoskeletal Disorders | 2010 | 10.1186/1471-2474-11-74 | English |
| The Lubinus patellofemoral arthroplasty: a series of 17 cases | T. N. BoardA. MahmoodW. G. RyanA. J. Banks | Archives of Orthopaedic and Trauma Surgery | 2004 | 10.1007/s00402-004-0645-x | English |
| Inadequacy of computed tomography for pre-operative planning of patellofemoral arthroplasty | Mo Saffarini, Jacobus H Müller, Giuseppe La Barbera, Gerjon Hannink, Kyung Jin Cho, Cécile Toanen, David Dejour | Knee Surgery, Sports Traumatology, Arthroscopy | 2018 | 10.1007/s00167-017-4474-1 | English |
| Prospective clinical and radiological two-year results after patellofemoral arthroplasty using an implant with an asymmetric trochlea design | Knut Beitzel, Philip B Schöttle, Matthias Cotic, Vyas Dharmesh, Andreas B Imhoff | Knee Surgery, Sports Traumatology, Arthroscopy | 2013 | 10.1007/s00167-012-2022-6 | English |
| Patellofemoral arthroplasty: a multi-centre study with minimum 2-year follow-up | Wayne B. LeadbetterFrank R. KolisekRichard L. LevittAndrew F. BrookerPatrick ZietzDavid R. MarkerPeter M. BonuttiMichael A. Mont | International Orthopaedics | 2009 | 10.1007/s00264-008-0692-y | English |
| Smoking, unemployment, female sex, obesity, and medication use yield worse outcomes in patellofemoral arthroplasty | Vishal S. DesaiAyoosh PareekCasey M. DeDeugdOrlando D. SabbagAaron J. KrychNancy M. CummingsDiane L. Dahm | Knee Surgery, Sports Traumatology, Arthroscopy | 2020 | 10.1007/s00167-019-05704-y | English |
| The short-term effectiveness and safety of second-generation patellofemoral arthroplasty and total knee arthroplasty on isolated patellofemoral osteoarthritis: a systematic review and meta-analysis | Chengxin LiZhizhuo LiLijun ShiFuqiang GaoWei Sun | Journal of Orthopaedic Surgery and Research | 2021 | 10.1186/s13018-021-02509-z | English |
| Similar postoperative patient-reported outcome in both second generation patellofemoral arthroplasty and total knee arthroplasty for treatment of isolated patellofemoral osteoarthritis: a systematic review | Kristine Ifigenia Bunyoz, Sébastien Lustig, Anders Troelsen | Knee Surgery, Sports Traumatology, Arthroscopy | 2019 | 10.1007/s00167-018-5151-8 | English |
| Computer-Assisted Navigation in Patellofemoral Arthroplasty: a New Technique to Improve Rotational Position of the Trochlea | Philippe Hernigou MDCharles Henri Flouzat-Lachaniette MDWilliam Delblond MDPascal Duffiet MDDidier Julian MD | HSS Journal 庐 | 2013 | 10.1007/s11420-013-9328-x | English |
| Early outcomes of an anatomic trochlear-cutting patellofemoral arthroplasty: patient selection is key | David DejourMo SaffariniYves MalemoMarco PungitoreJeremy ValluyLuca NoverGuillaume Demey | Knee Surgery, Sports Traumatology, Arthroscopy | 2019 | 10.1007/s00167-019-05368-8 | English |
| Patellofemoral arthroplasty influences tibiofemoral kinematics: the effect of patellar thickness | Hilde VandenneuckerLuc LabeyJan VictorJos Vander SlotenKaat DesloovereJohan Bellemans | Knee Surgery, Sports Traumatology, Arthroscopy | 2014 | 10.1007/s00167-014-3160-9 | English |
| Patient-related outcomes of patellofemoral arthroplasty: experience of a single center | W. Y. M. AbeysekeraW. Schenk | Arthroplasty | 2021 | 10.1186/s42836-021-00074-8 | English |
| Evidence of trochlear dysplasia in patellofemoral arthroplasty designs | Mo SaffariniPanagiotis G. NtagiopoulosGuillaume DemeyBenoit Le NegaretDavid H. Dejour | Knee Surgery, Sports Traumatology, Arthroscopy | 2014 | 10.1007/s00167-014-2967-8 | English |
| Patellofemoral arthroplasty: outcomes and factors associated with early progression of tibiofemoral arthritis | Diane L. DahmMichael M. KalisvaartMichael J. StuartSeth W. Slettedahl | Knee Surgery, Sports Traumatology, Arthroscopy | 2014 | 10.1007/s00167-014-3202-3 | English |
| Survivorship and functional outcomes of patellofemoral arthroplasty: a systematic review | J. P. van der ListH. ChawlaH. A. ZuiderbaanA. D. Pearle | Knee Surgery, Sports Traumatology, Arthroscopy | 2017 | 10.1007/s00167-015-3878-z | English |
| A matched-pair comparison of inlay and onlay trochlear designs for patellofemoral arthroplasty: no differences in clinical outcome but less progression of osteoarthritis with inlay designs | Matthias J. FeuchtMatthias CoticKnut BeitzelJulia F. BaldiniGebhart MeidingerPhilip B. Sch枚ttleAndreas B. Imhoff | Knee Surgery, Sports Traumatology, Arthroscopy | 2017 | 10.1007/s00167-015-3733-2 | English |
| Patellofemoral arthroplasty: expert opinion | Paul HoogervorstElizabeth A. Arendt | Journal of Experimental Orthopaedics | 2022 | 10.1186/s40634-022-00457-z | English |
| Patellofemoral arthroplasty versus total knee arthroplasty for isolated patellofemoral osteoarthritis: a systematic review and meta-analysis | Guanrong PengMin LiuZhenhua GuanYunfei HouQiang LiuXiaobo SunXingyang ZhuWenjun FengJianchun ZengZhangrong ZhongYirong Zeng | Journal of Orthopaedic Surgery and Research | 2021 | 10.1186/s13018-021-02414-5 | English |
| Surgical management of young arthritic knee: a review | Varatharaj MounasamySenthil Sambandam | European Journal of Orthopaedic Surgery & Traumatology | 2008 | 10.1007/s00590-008-0353-2 | English |
| La protesi femoro-rotulea: a che punto siamo? | S. RomagnoliF. VerdeE. BibbianiN. CastelnuovoN. Gioni | Archivio di Ortopedia e Reumatologia | 2008 | 10.1007/s10261-008-0233-7 | Italian |
| The patellofemoral joint and its historical roots: the Lyon School of Knee Surgery | David H. Dejour | Knee Surgery, Sports Traumatology, Arthroscopy | 2013 | 10.1007/s00167-012-2331-9 | English |
| Posters |  | Knee Surgery, Sports Traumatology, Arthroscopy | 2016 | 10.1007/s00167-016-4080-7 | English |
| Malrotation deformities of the lower extremity and implications on total knee arthroplasty: a narrative review | Geert PeersmanKim TaeymansChristophe JansPhilippe VuylstekePeter FennemaThomas Heyse | Archives of Orthopaedic and Trauma Surgery | 2016 | 10.1007/s00402-016-2554-1 | English |
| Tissue sparing surgery in knee reconstruction: unicompartmental (UKA), patellofemoral (PFA), UKA + PFA, bi-unicompartmental (Bi-UKA) arthroplasties | N. ConfalonieriA. ManzottiF. MontironiC. Pullen | Journal of Orthopaedics and Traumatology | 2008 | 10.1007/s10195-008-0015-5 | English |
| Complications after patello-femoral versus total knee replacement in the treatment of isolated patello-femoral osteoarthritis. A meta-analysis | C. J. DyN. FrancoY. MaM. MazumdarM. M. McCarthyA. Gonzalez Della Valle | Knee Surgery, Sports Traumatology, Arthroscopy | 2012 | 10.1007/s00167-011-1677-8 | English |
| Significant increase in quantity and quality of knee arthroplasty related research in KSSTA over the past 15 years | Stephanie KirschbaumThilo KakzhadFabian GranrathAndrzej JasinaJakub OronowiczCarsten PerkaSebastian KopfClemens GwinnerMatthias Pumberger | Knee Surgery, Sports Traumatology, Arthroscopy | 2022 | 10.1007/s00167-021-06555-2 | English |
| Bicompartmental (uni plus patellofemoral) versus total knee arthroplasty: a match-paired study | A. BiazzoF. SilvestriniA. ManzottiN. Confalonieri | MUSCULOSKELETAL SURGERY | 2019 | 10.1007/s12306-018-0540-1 | English |
| Patellectomy for osteoarthritis: a new tension preserving surgical technique to reconstruct the extensor mechanism with retrospective review of long-term follow-up | Vipin AsopaCharles Willis-OwenGreg Keene | Journal of Orthopaedic Surgery and Research | 2015 | 10.1186/s13018-015-0237-1 | English |
| Isolierter patellofemoraler Gelenkersatz | G. PagenstertM. Liebensteiner | Arthroskopie | 2015 | 10.1007/s00142-015-0029-y | German |
| A high level of satisfaction after bicompartmental individualized knee arthroplasty with patient-specific implants and instruments | Takahiro OguraKiet LeGergo MerkelyTim BryantTom Minas | Knee Surgery, Sports Traumatology, Arthroscopy | 2019 | 10.1007/s00167-018-5155-4 | English |
| Prospective evaluation of anatomic patellofemoral inlay resurfacing: clinical, radiographic, and sports-related results after 24 months | Andreas B. Imhoff, Matthias J. Feucht, Gebhart Meidinger, Philip B. Schöttle & Matthias Cotic | Knee Surgery, Sports Traumatology, Arthroscopy | 2015 | 10.1007/s00167-013-2786-3 | English |
| Posters |  | Knee Surgery, Sports Traumatology, Arthroscopy | 2018 | 10.1007/s00167-018-4867-9 | English |
| Contemporary knee arthroplasty: one fits all or time for diversity? | Johannes BeckmannMalin Kristin MeierChristian BenignusAndreas HeckerEmmanuel Thienpont | Archives of Orthopaedic and Trauma Surgery | 2021 | 10.1007/s00402-021-04042-4 | English |
| Midterm clinical results of the Autocentric II patellofemoral prosthesis | J. M. F. van WagenbergB. SpeignerT. GosensJ. de Waal Malefijt | International Orthopaedics | 2009 | 10.1007/s00264-009-0719-z | English |
| Reliable improvements in participation in low-impact sports following implantation of a patellofemoral inlay arthroplasty at mid-term follow-up | Jonas PogorzelskiMarco-Christopher RuppConrad KetzerMatthias CoticPatricia LutzSaskia BeeckAndreas B. ImhoffMatthias J. Feucht | Knee Surgery, Sports Traumatology, Arthroscopy | 2021 | 10.1007/s00167-020-06245-5 | English |
| Partial lateral facetectomy plus Insall procedure for the treatment of isolated patellofemoral osteoarthritis: survival analysis | Ferran Montserrat, Eduard Alentorn-Geli, Vicente León, Alberto Ginés-Cespedosa & Pau Rigol | Knee Surgery, Sports Traumatology, Arthroscopy | 2014 | 10.1007/s00167-012-2286-x | English |
| Endoprothetische Versorgung der isolierten Patellofemoralarthrose | Dr. Florian DirisamerMichael Liebensteiner | Arthroskopie | 2017 | 10.1007/s00142-017-0156-8 | German |
| The lack of retropatellar resurfacing at index surgery is significantly associated with failure in patients following patellofemoral inlay arthroplasty: a multi-center study of more than 260 patients | Andreas B. Imhoff, Eva Bartsch, Christoph Becher, Peter Behrens, Gerrit Bode, Matthias Cotic, Theresa Diermeier, Holger Falk, Matthias J. Feucht, Ulrich Haupt, Stefan Hinterwimmer, Johannes Holz, René Hutter, René Kaiser, Tobias Knoblauch, Wolfgang Nebelung, Philipp Niemeyer, Turlough O’Donnel, Geert Pagenstert, Thilo Patzer, Tim Rose, Marco C. Rupp, Thomas Tischer, Arne J. Venjakob, Stephan Vogt & Jonas Pogorzelski | Knee Surgery, Sports Traumatology, Arthroscopy | 2022 | 10.1007/s00167-021-06544-5 | English |
| A stand-alone lateral condyle-elevating trochlear osteotomy leads to high residual instability but no excessive increase in patellofemoral osteoarthritis at 12-year follow-up | S. TigchelaarJ. van SambeeckS. KoeterA. van Kampen | Knee Surgery, Sports Traumatology, Arthroscopy | 2018 | 10.1007/s00167-017-4602-y | English |
| Insufficient evidence to confirm benefits of custom partial knee arthroplasty: a systematic review | Demey G, Müller JH, Liebensteiner M, Pilot P, Nover L; European Knee Associates (EKA); Kort N. | Knee Surgery, Sports Traumatology, Arthroscopy | 2022 | 10.1007/s00167-021-06766-7 | English |
| Patellofemoral Pain in Adolescence and Adulthood: Same Same, but Different? | M. S. RathleffB. VicenzinoM. MiddelkoopT. Graven-NielsenR. van LinschotenP. H枚lmichK. Thorborg | Sports Medicine | 2015 | 10.1007/s40279-015-0364-1 | English |
| Posters |  | Knee Surgery, Sports Traumatology, Arthroscopy | 2014 | 10.1007/s00167-014-2917-5 | English |
| Patellofemoraler Gelenkersatz | J. BeckmannM. MeierA. HalderR. BestE. ThienpontDr. med. A. Beier | Arthroskopie | 2020 | 10.1007/s00142-020-00375-1 | German |
| Effects of laterally wedged insoles on symptoms and disease progression in medial knee osteoarthritis: a protocol for a randomised, double-blind, placebo controlled trial | Kim BennellKelly-Ann BowlesCraig PayneFlavia CicuttiniRichard OsborneAnthony HarrisRana Hinman | BMC Musculoskeletal Disorders | 2007 | 10.1186/1471-2474-8-96 | English |
| The evaluation and management of cartilage lesions affecting the patellofemoral joint | Eric J. StraussDavid K. Galos | Current Reviews in Musculoskeletal Medicine | 2013 | 10.1007/s12178-013-9157-z | English |
| Tibial tubercle advancement osteotomy with bone allograft for patellofemoral arthritis: a retrospective cohort study of 50 knees | Henry Dushan AtkinsonChristopher A. BaileySanjeev AnandParminder JohalRoger D. Oakeshott | Archives of Orthopaedic and Trauma Surgery | 2012 | 10.1007/s00402-011-1433-z | English |
| Endoprothetischer Teilersatz des patellofemoralen Gelenks | Hannes DegenhardtJonas PogorzelskiAndreas B. ImhoffProf. Dr. med. Matthias J. Feucht | Knie Journal | 2022 | 10.1007/s43205-022-00151-1 | German |
| Patellofemorale Inlay-Implantate – ein Fortschritt in der patellofemoralen Endoprothetik? | Hannes DegenhardtProf. Dr. med. Andreas B. Imhoffapl. Prof. Dr. med. Matthias J. FeuchtPD Dr. Jonas Pogorzelski MHBA | Der Orthopäde | 2021 | 10.1007/s00132-020-04059-4 | German |
| Primary lipoma arborescens of the knee may involve the development of early osteoarthritis if prompt synovectomy is not performed | Luis NateraPablo E. GelberJuan I. ErquiciaJuan Carlos Monllau | Journal of Orthopaedics and Traumatology | 2015 | 10.1007/s10195-014-0295-x | English |
| Patellotrochlearer Ersatz | M. CoticUniv. Prof. Dr. med. A. B. Imhoff | Der Orthopäde | 2014 | 10.1007/s00132-014-3006-7 | German |
| Are Revisions of Patellofemoral Arthroplasties More Like Primary or Revision TKAs | Sébastien Parratte, Alexandre Lunebourg, Matthieu Ollivier, Matthew P Abdel, Jean-Noël A Argenson | Clinical Orthopaedics and Related Research | 2015 | 10.1007/s11999-014-3756-x | English |
| Patellofemoraler Ersatz und Schlittenprothese | Dr. med. A. BeierD. SchrednitzkiA. MarxC. LohmannA. M. Halder | Arthroskopie | 2017 | 10.1007/s00142-017-0164-8 | German |
| UKA in combination with PFR at average 12-year follow-up | Thomas Jan HeyseAhmed KhefachaPhilippe Cartier | Archives of Orthopaedic and Trauma Surgery | 2010 | 10.1007/s00402-009-0997-3 | English |
| Development of a patient-reported outcome measure (PROM) and change measure for use in early recovery following hip or knee replacement | Louise H. StricklandDavid W. MurrayHemant G. PanditCrispin Jenkinson | Journal of Patient-Reported Outcomes | 2020 | 10.1186/s41687-020-00262-1 | English |
| The young osteoarthritic knee: dilemmas in management | Paul M SuttonEdward S Holloway | BMC Medicine | 2013 | 10.1186/1741-7015-11-14 | English |
| Warfarin—an expensive cause of delay in discharge of elective arthroplasty patients | R. VenkataramanK. PanousisA. H. DeakinG. SaleF. Picard | European Orthopaedics and Traumatology | 2015 | 10.1007/s12570-014-0285-8 | English |
| A 6-DOF parallel bone-grinding robot for cervical disc replacement surgery | Heqiang TianChenchen WangXiaoqing DangLining Sun | Medical & Biological Engineering & Computing | 2017 | 10.1007/s11517-017-1648-4 | English |
| A 21 % conversion rate to total knee arthroplasty of a first-generation patellofemoral prosthesis at a mean follow-up of 9.7 years | Paul HoogervorstRichard J. de JongGerjon HanninkAlbert van Kampen | International Orthopaedics | 2015 | 10.1007/s00264-015-2941-1 | English |
| Unicompartmental arthritis in the aging athlete: osteotomy and beyond | Stephen F. JohnstoneMichael J. TranovichDharmesh VyasVonda J. Wright | Current Reviews in Musculoskeletal Medicine | 2013 | 10.1007/s12178-013-9171-1 | English |
| Long-term Results With a Lateral Unicondylar Replacement | Jean-Noël A. Argenson MD, Sebastien Parratte MD, Antoine Bertani MD, Xavier Flecher MD & Jean-Manuel Aubaniac MD | Clinical Orthopaedics and Related Research | 2008 | 10.1007/s11999-008-0351-z | English |
| Preoperative patellofemoral anatomy affects failure rate after isolated patellofemoral inlay arthroplasty | Matthias J. FeuchtPatricia M. LutzConrad KetzerMarco C. RuppMatthias CoticAndreas B. ImhoffJonas Pogorzelski | Archives of Orthopaedic and Trauma Surgery | 2020 | 10.1007/s00402-020-03651-9 | English |
| Surgical Treatment of Isolated Patellofemoral Osteoarthritis | E. Carlos Rodriguez-Merchan MD, PhD | HSS Journal | 2014 | 10.1007/s11420-013-9375-3 | English |
| Robotic-Assisted Knee Arthroplasty (RAKA): The Technique, the Technology and the Transition | Vaibhav BagariaOmkar S. SadigalePrashant P. PawarRavi K. BashyalAjinkya AchalareMurali Poduval | Indian Journal of Orthopaedics | 2020 | 10.1007/s43465-020-00088-5 | English |
| The effect of age on the outcomes of cementless mobile bearing unicompartmental knee replacements | Hasan Raza MohammadStephen MellonAndrew JudgeChristopher DoddDavid Murray | Knee Surgery, Sports Traumatology, Arthroscopy | 2022 | 10.1007/s00167-020-06428-0 | English |
| The effects of hip muscle strengthening on knee load, pain, and function in people with knee osteoarthritis: a protocol for a randomised, single-blind controlled trial | Kim L BennellMichael A HuntTim V WrigleyDavid J HunterRana S Hinman | BMC Musculoskeletal Disorders | 2007 | 10.1186/1471-2474-8-121 | English |
| The posterior–anterior flexed view is better than the anterior–posterior view for assessing osteoarthritis of the knee | Kilian RuecklFriedrich BoettnerNoor MazaArmin RunerUlrich BechlerPeter Sculco | Skeletal Radiology | 2018 | 10.1007/s00256-017-2815-2 | English |
| Trends in primary and revision knee arthroplasty among orthopaedic surgeons who take the American Board of Orthopaedics part II exam | Aidin Eslam PourThomas L. BradburyPatrick HorstJohn J. HarrastGreg A. ErensJames R. Roberson | International Orthopaedics | 2016 | 10.1007/s00264-016-3137-z | English |
| Surgical Treatment of Isolated Patellofemoral Osteoarthritis | Roland Becker MD, PhDMartin R枚pke MDAnja Krull MDVolker Musahl MDWolfgang Nebelung MD, PhD | Clinical Orthopaedics and Related Research | 2008 | 10.1007/s11999-007-0071-9 | English |
| Does knee awareness differ between different knee arthroplasty prostheses? A matched, case-control, cross-sectional study | Morten G. ThomsenRoshan LatifiThomas KallemoseHenrik HustedAnders Troelsen | BMC Musculoskeletal Disorders | 2016 | 10.1186/s12891-016-1001-3 | English |
| Clinical outcome of bi-unicompartmental knee arthroplasty for both medial and lateral femorotibial arthritis: a systematic review-is there proof of concept? | Keizo WadaAndrew PriceKirill GromovSebastien LustigAnders Troelsen | Archives of Orthopaedic and Trauma Surgery | 2020 | 10.1007/s00402-020-03492-6 | English |
| High patient satisfaction with significant improvement in knee function and pain relief after mid-term follow-up in patients with isolated patellofemoral inlay arthroplasty | Andreas B. ImhoffMatthias J. FeuchtEva BartschMatthias CoticJonas Pogorzelski | Knee Surgery, Sports Traumatology, Arthroscopy | 2019 | 10.1007/s00167-018-5173-2 | English |
| Joint replacement surgery in Ghana (West Africa) | Zuyun YanXinqiao TangXiaoming ChenZhong Liu | International Orthopaedics | 2019 | 10.1007/s00264-019-04321-1 | English |
| Measuring appropriate need for unicompartmental knee arthroplasty: results of the MANUKA study | Antonio KlasanMatthias LugerRainer HochgattererSimon W. Young | Knee Surgery, Sports Traumatology, Arthroscopy | 2022 | 10.1007/s00167-021-06632-6 | English |
| Treatment Options for Patellofemoral Arthritis | Anne KuwabaraMark CinqueTaylor RaySeth Lawrence Sherman | Current Reviews in Musculoskeletal Medicine | 2022 | 10.1007/s12178-022-09740-z | English |
| Survival of Bicompartmental Knee Arthroplasty at 5 to 23 Years | Sebastien Parratte MDVanessa Pauly MSJean-Manuel Aubaniac MDJean-Noel A. Argenson MD | Clinical Orthopaedics and Related Research | 2010 | 10.1007/s11999-009-1018-0 | English |
| Complications in Brief: Arthroscopic Lateral Release | Hussein Elkousy MD | Clinical Orthopaedics and Related Research | 2012 | 10.1007/s11999-012-2383-7 | English |
| Patellofemoral function in total condylar knee arthroplasty | O. Sneppen, G. H. Gudmundsson & C. Bünger | International Orthopaedics | 1985 | 10.1007/BF00267040 | English |
| Unicompartmental vs. segmental bicompartmental vs. total knee replacement: comparison of clinical outcomes | Oday Al-DadahGeorgina HawesPhilip J. Chapman-SheathJohn William TiceDavid S. Barrett | Knee Surgery & Related Research | 2020 | 10.1186/s43019-020-00065-0 | English |
| Early outcomes and predictors of patient satisfaction after TKA: a prospective study of 200 cases with a contemporary cemented rotating platform implant design | Corné van Loon, Niels Baas, Verdonna Huey, James Lesko, Geert Meermans & Diederik Vergroesen | Journal of Experimental Orthopaedics | 2021 | 10.1186/s40634-021-00347-w | English |
| Proven and nonproven facts in knee arthroplasty | N. Gschwend & D. Ivošević-Radovanović | Archives of orthopaedic and traumatic surgery | 1988 | 10.1007/BF00451593 | English |
| Automatic assessment of knee osteoarthritis severity in portable devices based on deep learning | Jianfeng YangQuanbo JiMing NiGuoqiang ZhangYan Wang | Journal of Orthopaedic Surgery and Research | 2022 | 10.1186/s13018-022-03429-2 | English |
| Results of a French multicentre retrospective experience with four hundred and eighteen failed unicondylar knee arthroplasties | Saragaglia, D; Bonnin, M; Dejour, D; Deschamps, G; Chol, C; Chabert, B; Refaie, R | International Orthopaedics | 2013 | 10.1007/s00264-013-1915-4 | English |
| Current practice of orthopaedic surgical skills training raises performance of supervised residents in total knee arthroplasty to levels equal to those of orthopaedic surgeons | Luuk TheelenCheryll BischoffBernd GrimmIde C. Heyligers | Perspectives on Medical Education | 2018 | 10.1007/s40037-018-0408-y | English |
| Free papers |  | Knee Surgery, Sports Traumatology, Arthroscopy | 2014 | 10.1007/s00167-014-2915-7 | English |
| Unicompartmental knee arthroplasty with patelloplasty | J. AntoniouA. HadjipavlouP. EnkerA. Antoniou | International Orthopaedics | 1996 | 10.1007/s002640050038 | English |
| Maquet III procedure: what remains after initial complications - long-term results | Fernando FonsecaJo茫o Pedro OliveiraPinho Marques | Journal of Orthopaedic Surgery and Research | 2013 | 10.1186/1749-799X-8-11 | English |
| A progressive scholarly acceptance analysis of robot-assisted arthroplasty: a review of the literature and prediction of future research trends | Dylan MissoEmily ZhenJohn KellyDermot CollopyGavin Clark | Journal of Robotic Surgery | 2021 | 10.1007/s11701-020-01173-5 | English |
| Management of patellar problems in skeletally mature patients with nail–patella syndrome | Lucie LouboutinDaniel WascherPhilippe Neyret | Knee Surgery, Sports Traumatology, Arthroscopy | 2017 | 10.1007/s00167-016-4044-y | English |
| Free Papers |  | Knee Surgery, Sports Traumatology, Arthroscopy | 2008 | 10.1007/s00167-008-0544-8 | English |
| Factors contributing to 1-year dissatisfaction after total knee arthroplasty: a nomogram prediction model | Mieralimu MuertizhaXinTian CaiBaochao JiAbudousaimi AimaitiLi Cao | Journal of Orthopaedic Surgery and Research | 2022 | 10.1186/s13018-022-03205-2 | English |
| Patient-specific instrumentation combined with a new tool for gap balancing is useful in total knee replacement: a 3-year follow-up of a retrospective study | Ting DengTangyou LiuQing LeiLihong CaiSong Chen | Journal of Orthopaedic Surgery and Research | 2021 | 10.1186/s13018-021-02467-6 | English |
| Effects of medications on incidence and risk of knee and hip joint replacement in patients with osteoarthritis: a systematic review and meta-analysis | Beibei CuiYuehong ChenYunru TianHuan LiuYupeng HuangGeng YinQibing Xie | Advances in Rheumatology | 2022 | 10.1186/s42358-022-00253-4 | English |
| Primary total knee arthroplasty assisted by computed tomography-free navigation for secondary knee osteoarthritis following massive calcium phosphate cement packing for distal femoral giant-cell bone tumor treatment: a case report | Akihiko TakeuchiNorio YamamotoTakaaki OhmoriKatsuhiro HayashiShinji MiwaKentaro IgarashiTakashi HiguchiKensaku AbeHirotaka YonezawaSei MorinagaYoshihiro ArakiYohei AsanoShiro SaitoHiroyuki Tsuchiya | BMC Musculoskeletal Disorders | 2022 | 10.1186/s12891-022-05131-0 | English |
| Patientenspezifische Instrumentierung und Teilprothesen am Knie | C. BenignusM. K. MeierM. T. HirschmannC. O. TibeskuProf. Dr. med. habil. J. Beckmann | Arthroskopie | 2021 | 10.1007/s00142-021-00463-w | German |
| Anterior knee pain in younger adults as a precursor to subsequent patellofemoral osteoarthritis: a systematic review | Martin J ThomasLaurence WoodJames SelfeGeorge Peat | BMC Musculoskeletal Disorders | 2010 | 10.1186/1471-2474-11-201 | English |
| Patellofemoral pain: an update on diagnostic and treatment options | Moira M. McCarthySabrina M. Strickland | Current Reviews in Musculoskeletal Medicine | 2013 | 10.1007/s12178-013-9159-x | English |
| The patient results and satisfaction of knee arthroplasty in a validated grading system | Christiaan Rudolf OosthuizenCatherine Van Der StraetenInnocent MaposaChristian Hugo SnyckersDuwayne Peter VermaakSebastian Magobotha | International Orthopaedics | 2019 | 10.1007/s00264-019-04412-z | English |
| Is isolated insert exchange a valuable choice for polyethylene wear in metal-backed unicompartmental knee arthroplasty? | Alexandre Lunebourg, Sébastien Parratte, Alexandre Galland, François Lecuire, Matthieu Ollivier & Jean-Noël Argenson | Knee Surgery, Sports Traumatology, Arthroscopy | 2016 | 10.1007/s00167-014-3392-8 | English |
| No Long-term Difference Between Fixed and Mobile Medial Unicompartmental Arthroplasty | Sebastien Parratte MDVanessa Pauly MSJean-Manuel Aubaniac MDJean-Noel A. Argenson MD | Clinical Orthopaedics and Related Research | 2012 | 10.1007/s11999-011-1961-4 | English |
| Free Papers |  | Knee Surgery, Sports Traumatology, Arthroscopy | 2010 | 10.1007/s00167-010-1116-2 | English |
| Trochleoplasty procedures show complication rates similar to other patellar-stabilizing procedures | Jordy D. P. van SambeeckSebastiaan A. W. van de GroesNico VerdonschotGerjon Hannink | Knee Surgery, Sports Traumatology, Arthroscopy | 2018 | 10.1007/s00167-017-4766-5 | English |
| Trochleoplasty provides good clinical outcomes and an acceptable complication profile in both short and long-term follow-up | Laurie A. HiemstraDevin PetersonMichael YoussefJohn SolimanLaura BanfieldOlufemi R. Ayeni | Knee Surgery, Sports Traumatology, Arthroscopy | 2019 | 10.1007/s00167-018-5311-x | English |
| Derotational osteotomy at the distal femur is effective to treat patients with patellar instability | Florian B. ImhoffMatthias CoticFranz LiskaFelix G. E. DyrnaKnut BeitzelAndreas B. ImhoffElmar Herbst | Knee Surgery, Sports Traumatology, Arthroscopy | 2019 | 10.1007/s00167-018-5212-z | English |
| Abstract Book - 93rd National Congress of the Italian Society of Orthopaedics and Traumatology |  | Journal of Orthopaedics and Traumatology | 2008 | 10.1007/s10195-008-0030-6 | English |
| Anterior Knee Pain: State of the Art | Riccardo D’Ambrosi, Amit Meena, Akshya Raj, Nicola Ursino & Timothy E. Hewett | Sports Medicine - Open | 2022 | 10.1186/s40798-022-00488-x | English |
| Unicompartmental knee arthroplasty, an enigma, and the ten enigmas of medial UKA | Anurag MittalPrashant MeshramWoo Hyun KimTae Kyun Kim | Journal of Orthopaedics and Traumatology | 2020 | 10.1186/s10195-020-00551-x | English |
| Outcomes and reoperation rates after tibial tubercle transfer and medial patellofemoral ligament reconstruction: higher revision stabilization in patients with trochlear dysplasia and patella alta | Jacob D. GorbatyDax T. VarkeyIan S. HongDavid P. TrofaSusan M. OdumDana P. PiaseckiBryan M. SaltzmanJames E. Fleischli | Knee Surgery, Sports Traumatology, Arthroscopy | 2022 | 10.1007/s00167-021-06784-5 | English |
| Patellofemorale Arthrose | Prof. Dr. A. KeshmiriA. B. ImhoffF. Dirisamer | Arthroskopie | 2020 | 10.1007/s00142-020-00379-x | German |
| Retropatellar contact characteristics in total knee arthroplasty with and without patellar resurfacing | S. Fuchs, G. Schütte, H. Witte & D. Rosenbaum | International Orthopaedics | 2000 | 10.1007/s002640000140 | English |
| Observed kneeling ability after total, unicompartmental and patellofemoral knee arthroplasty: perception versus reality | M. A. HassaballaA. J. PorteousJ. H. Newman | Knee Surgery, Sports Traumatology, Arthroscopy | 2004 | 10.1007/s00167-003-0376-5 | English |
| News-Screen Orthopädie & Traumatologie | Priv.-Doz. DDr Lukas Holzer | Journal für Mineralstoffwechsel & Muskuloskelettale Erkrankungen | 2022 | 10.1007/s41970-022-00208-7 | German |
| Rediscovering the patellofemoral joint | David DejourElizabeth ArendtStefano Zaffagnini | Knee Surgery, Sports Traumatology, Arthroscopy | 2014 | 10.1007/s00167-014-3208-x | English |
| Asia-Pacific venous thromboembolism consensus in knee and hip arthroplasty and hip fracture surgery: Part 1. Diagnosis and risk factors | Srihatach NgarmukosKang-Il KimSiwadol WongsakThanainit ChotanaphutiYutaka InabaCheng-Fong ChenDavid Liu | Knee Surgery & Related Research | 2021 | 10.1186/s43019-021-00099-y | English |
| Early failure with the Journey-Deuce bicompartmental knee arthroplasty | A. G. DudhniwalaN. K. RathS. JoshyM. C. ForsterS. P. White | European Journal of Orthopaedic Surgery & Traumatology | 2016 | 10.1007/s00590-016-1760-4 | English |
| Surgeons and robots | Andreas F. MavrogenisMarius M. Scarlat | International Orthopaedics | 2019 | 10.1007/s00264-019-04345-7 | English |
| Biplanar supracondylar femoral derotation osteotomy for patellofemoral malalignment: the anterior closed-wedge technique | Stefan HinterwimmerPhilipp MinzlaffTim SaierPhilipp NiemeyerAndreas B. ImhoffMatthias J. Feucht | Knee Surgery, Sports Traumatology, Arthroscopy | 2014 | 10.1007/s00167-014-2993-6 | English |
| News-Screen Orthopädie & Traumatologie |  | Journal für Mineralstoffwechsel & Muskuloskelettale Erkrankungen | 2019 | 10.1007/s41970-019-00081-x | German |
| Abstracts of the European Society of Skeletal Radiology |  | Skeletal Radiology | 2006 | 10.1007/s00256-006-0118-0 | English |
| Acceptable outcomes with unicompartmental knee replacement and PCL deficiency are achievable: a case series of nine patients | Pengfei LiJames KennedyHasan Raza MohammadZhihui PangStephen MellonWilliam JacksonAndrew PriceChristopher DoddDavid Murray | Knee Surgery, Sports Traumatology, Arthroscopy | 2021 | 10.1007/s00167-020-06112-3 | English |
| Modification of the Sheehan Knee arthroplasty for additional replacement of the femoropatellar articulation | R. MiehlkeH. W. Croon | Archives of orthopaedic and traumatic surgery | 1980 | 10.1007/BF00389723 | English |
| Is Recovery Faster for Mobile-bearing Unicompartmental than Total Knee Arthroplasty? | Adolph V. Lombardi Jr. MDKeith R. Berend MDChristopher A. Walter DOJorge Aziz-Jacobo MDNicholas A. Cheney DO | Clinical Orthopaedics and Related Research | 2009 | 10.1007/s11999-009-0731-z | English |
| Computer-Navigated Total Knee Replacement with GRAFTJACKET (Allograft) to Improve Patella Tracking in Osteoarthritic Valgus Knee with Chronic Patella Dislocation | Fouad A. ChaudhryTom Symes | SN Comprehensive Clinical Medicine | 2021 | 10.1007/s42399-021-00737-2 | English |
| Bicompartmental individualized knee replacement | A. F. SteinertJ. BeckmannB. M. HolzapfelM. RudertDr. J. Arnholdt | Operative Orthop盲die und Traumatologie | 2017 | 10.1007/s00064-017-0484-x | English |
| Patient-specific instrument for unicompartmental knee arthroplasty does not reduce the outliers in alignment or improve postoperative function: a meta-analysis and systematic review | Mingyang LiYi ZengYuangang WuYuan LiuWenxing WeiLimin WuBo-qiang PengJiayi LiBin Shen | Archives of Orthopaedic and Trauma Surgery | 2020 | 10.1007/s00402-020-03429-z | English |
| Kinematic alignment of medial UKA is safe: a systematic review | Charles Rivière, Sivan Sivaloganathan, Loic Villet, Philippe Cartier, Sébastien Lustig Pascal-André Vendittoli, Justin Cobb | Knee Surgery, Sports Traumatology, Arthroscopy | 2022 | 10.1007/s00167-021-06462-6 | English |
| Advances in unicompartmental knee arthroplasty with minimally invasive techniques | G. L. MaistrelliS. Riaz | Journal of Orthopaedics and Traumatology | 2006 | 10.1007/s10195-006-0122-0 | English |
| Do Patients Return to Work After Total Knee Arthroplasty? | Adolph V. Lombardi Jr MDRyan M. Nunley MDKeith R. Berend MDErin L. Ruh MSJohn C. Clohisy MDWilliam G. Hamilton MDCraig J. Della Valle MDJavad Parvizi MDRobert L. Barrack MD | Clinical Orthopaedics and Related Research庐 | 2014 | 10.1007/s11999-013-3099-z | English |
| Robotic alignment of femoral cutting mask during total knee arthroplasty | E. De MomiP. CerveriE. GambarettoM. MarchenteO. EffrettiS. BarbarigaG. GiniG. Ferrigno | International Journal of Computer Assisted Radiology and Surgery | 2008 | 10.1007/s11548-008-0253-2 | English |
| Low percentage of surgeons meet the minimum recommended unicompartmental knee arthroplasty usage thresholds: Analysis of 3037 Surgeons from Three National Joint Registries | Antonio KlasanDavid A. ParkerPeter L. LewisSimon W. Young | Knee Surgery, Sports Traumatology, Arthroscopy | 2022 | 10.1007/s00167-021-06437-7 | English |
| RETRACTED ARTICLE: Unicompartmental knee arthroplasty: a review of literature | Bernardino Saccomanni | Clinical Rheumatology | 2010 | 10.1007/s10067-009-1354-1 | English |
| Assessing function in patients undergoing joint replacement: a study protocol for a cohort study | Vikki WyldeAshley W BlomStijn BolinkLuke BruntonPaul DieppeRachael Gooberman-HillBernd GrimmCindy MannErik Lenguerrand | BMC Musculoskeletal Disorders | 2012 | 10.1186/1471-2474-13-220 | English |
| Hyaluronic acid in management of advanced osteoarthritis of the knee: retrospective analysis | Ashish AnandFrederick BalduiniKen Rogers | European Journal of Orthopaedic Surgery & Traumatology | 2010 | 10.1007/s00590-010-0635-3 | English |
| A meta-analysis of unicompartmental knee arthroplasty revised to total knee arthroplasty versus primary total knee arthroplasty | Xuedong SunZheng Su | Journal of Orthopaedic Surgery and Research | 2018 | 10.1186/s13018-018-0859-1 | English |
| Patellar osteotomy and Albee's procedure for dysplastic patellar instability | Nitin P. BadheIan W. Forster | European Journal of Orthopaedic Surgery & Traumatology | 2003 | 10.1007/s00590-003-0063-8 | English |
| Posters |  | Knee Surgery, Sports Traumatology, Arthroscopy | 2010 | 10.1007/s00167-010-1119-z | English |
| An alternative patellar resurfacing technique in knee replacement: patellofemoral fascial interposition arthroplasty | Volkan OztunaVasfi KaratosunBayram UnverIrfan AyanFehmi Kuyurtar | Knee Surgery, Sports Traumatology, Arthroscopy | 2007 | 10.1007/s00167-007-0375-z | English |
| Protesi femoro-rotulea | Carola PiloneFederico DettoniDavide E. BonasiaFederica RossoUmberto CottinoRoberto Rossi | LO SCALPELLO-OTODI Educational | 2019 | 10.1007/s11639-019-00325-z | Italian |
| Free papers |  | Knee Surgery, Sports Traumatology, Arthroscopy | 2016 | 10.1007/s00167-016-4079-0 | English |
| Joint awareness in osteoarthritis of the hip and knee evaluated with the ‘Forgotten Joint’ Score before and after joint replacement | E. ThienpontA. Vanden BergheP. E. SchwabJ. P. ForthommeO. Cornu | Knee Surgery, Sports Traumatology, Arthroscopy | 2016 | 10.1007/s00167-015-3970-4 | English |
| Age at surgery is correlated with pain scores following trochlear osteotomy in lateral patellar instability: a cross-sectional study of 113 cases | Jordy D. P. van SambeeckNico VerdonschotAlbert Van KampenSebastiaan A. W. van de Groes | Journal of Orthopaedic Surgery and Research | 2021 | 10.1186/s13018-021-02485-4 | English |
| Robotic-assisted knee arthroplasty: an evolution in progress. A concise review of the available systems and the data supporting them | Johanna ElliottJobe ShatrovBrett FritschDavid Parker | Archives of Orthopaedic and Trauma Surgery | 2021 | 10.1007/s00402-021-04134-1 | English |
| Risk factors for revision of total knee arthroplasty: a scoping review | L.L. JasperC. A. JonesJ. MollinsS. L. PoharL. A. Beaupre | BMC Musculoskeletal Disorders | 2016 | 10.1186/s12891-016-1025-8 | English |
| Growth Plate Injuries of the Lower Extremity: Case Examples and Lessons Learned | Prof. Samir SabharwalSanjeev Sabharwal | Indian Journal of Orthopaedics | 2018 | 10.4103/ortho.IJOrtho_313_17 | English |
| The John Insall Award: Both Morphotype and Gender Influence the Shape of the Knee in Patients Undergoing TKA | Johan Bellemans MD, PhDKarel Carpentier MDHilde Vandenneucker MDJohan Vanlauwe MDJan Victor MD | Clinical Orthopaedics and Related Research | 2010 | 10.1007/s11999-009-1016-2 | English |
| Extent of Tibiofemoral Osteoarthritis Before Knee Arthroplasty: Multicenter Data from the Osteoarthritis Initiative | Daniel L. Riddle PhDWilliam A. Jiranek MDRobert S. Neff MDDerek Whitaker MDJason R. Hull MD | Clinical Orthopaedics and Related Research | 2012 | 10.1007/s11999-012-2328-1 | English |
| Are results of total knee arthroplasty for isolated patellofemoral OA as good as for medial compartment OA? A medium-term retrospective comparative study | D. SaragagliaR. MaderR. Refaie | European Journal of Orthopaedic Surgery & Traumatology | 2015 | 10.1007/s00590-014-1516-y | English |
| Concordance between clinical and radiographic evaluations of knee osteoarthritis | Camille ParsonsNicholas R. FuggleMark H. EdwardsLyndsey GoulstonAnna E. LitwicDarshan JagannathSuzan van der PasCyrus CooperElaine M. Dennison | Aging Clinical and Experimental Research | 2018 | 10.1007/s40520-017-0847-z | English |
| Higher forgotten joint score for fixed-bearing than for mobile-bearing total knee arthroplasty | E. ThienpontD. Zorman | Knee Surgery, Sports Traumatology, Arthroscopy | 2016 | 10.1007/s00167-015-3663-z | English |
| Patellar thickness and lateral retinacular release affects patellofemoral kinematics in total knee arthroplasty | Azhar M. MericanKanishka M. GhoshFerdinando Rodriguez Y. BaenaDavid J. DeehanAndrew A. Amis | Knee Surgery, Sports Traumatology, Arthroscopy | 2014 | 10.1007/s00167-012-2312-z | English |
| Bicompartmental knee arthroplasty of the patellofemoral and medial compartments | Emmanuel ThienpontAndrew Price | Knee Surgery, Sports Traumatology, Arthroscopy | 2013 | 10.1007/s00167-012-2303-0 | English |
| What Is the Frequency of Noise Generation in Modern Knee Arthroplasty and Is It Associated With Residual Symptoms? | Denis Nam MD, MScToby Barrack BARyan M. Nunley MDRobert L. Barrack MD | Clinical Orthopaedics and Related Research | 2017 | 10.1007/s11999-016-4701-y | English |
| Trochlear osteotomy for patellar instability: satisfactory minimum 2-year results in patients with dysplasia of the trochlea | Sander Koëter, Dean Pakvis, Corne J. M. van Loon & Albert van Kampen | Knee Surgery, Sports Traumatology, Arthroscopy | 2007 | 10.1007/s00167-006-0174-y | English |
| Clinical and radiographic evaluation of modular knee replacement | P. CartierM. MammeriP. Villers | International Orthopaedics | 1982 | 10.1007/BF00267813 | English |
| Surgical treatment of patellofemoral instability using trochleoplasty or MPFL reconstruction: a systematic review | Enrique Adrian TestaCarlo CamathiasFelix AmslerPhilipp HenleNiklaus F. FriederichMichael Tobias Hirschmann | Knee Surgery, Sports Traumatology, Arthroscopy | 2017 | 10.1007/s00167-015-3698-1 | English |
| Lateral unicompartmental knee arthroplasty: a review | Thomas J. HeyseCarsten O. Tibesku | Archives of Orthopaedic and Trauma Surgery | 2010 | 10.1007/s00402-010-1137-9 | English |
| Relationship between patellofemoral finite helical axis and femoral trans-epicondylar axis using a static magnetic resonance-based methodology | Zhenguo YuHong CaiBin YangJie YaoKe ZhangHua TianZhongjun Liu | Journal of Orthopaedic Surgery and Research | 2021 | 10.1186/s13018-021-02328-2 | English |
| Influence of bi- and tri-compartmental knee arthroplasty on the kinematics of the knee joint | Markus Wünschel, JiaHsuan Lo, Torsten Dilger, Nikolaus Wülker & Otto Müller | BMC Musculoskeletal Disorders | 2011 | 10.1186/1471-2474-12-29 | English |
| Correlation between varus knee malalignment and patellofemoral osteoarthritis | Shuhei OtsukiMikio NakajimaYoshinori OkamotoShuhei OdaYoshiaki HoshiyamaGo IidaMasashi Neo | Knee Surgery, Sports Traumatology, Arthroscopy | 2016 | 10.1007/s00167-014-3360-3 | English |
| The efficiency and safety of steroid addition to multimodal cocktail periarticular injection in knee joint arthroplasty: a meta-analysis of randomized controlled trials | Zhenhan DengYusheng LiGarrett R. StormRonak Naveenchandra KotianXuying SunGuanghua LeiShanshan GaoWei Lu | Scientific Reports | 2019 | 10.1038/s41598-019-43540-9 | English |
| Do early life factors affect the development of knee osteoarthritis in later life: a narrative review | Benny AntonyGraeme JonesXingzhong JinChanghai Ding | Arthritis Research & Therapy | 2016 | 10.1186/s13075-016-1104-0 | English |
| Association between patellar cartilage defects and patellofemoral geometry: a matched-pair MRI comparison of patients with and without isolated patellar cartilage defects | Julian Mehl, Matthias J. Feucht, Gerrit Bode, David Dovi-Akue, Norbert P. Südkamp & Philipp Niemeyer | Knee Surgery, Sports Traumatology, Arthroscopy | 2016 | 10.1007/s00167-014-3385-7 | English |
| Proposal of a therapeutic protocol for selected patients with patellofemoral knee osteoarthritis: arthroscopic lateral retinacular release followed by viscosupplementation | M. FoscoE. Dagher | MUSCULOSKELETAL SURGERY | 2016 | 10.1007/s12306-016-0402-7 | English |
| Defining Core competencies of the European Society for Sports Traumatology, knee surgery and arthroscopy | Michael Taylor RossMartin Lind | Journal of Experimental Orthopaedics | 2020 | 10.1186/s40634-020-00276-0 | English |
| Pain management of unicompartmental (UKA) vs. total knee arthroplasty (TKA) based on a matched pair analysis of 4144 cases | Franziska Leiss, Julia Sabrina Götz, Günther Maderbacher, Florian Zeman, Winfried Meissner, Joachim Grifka, Achim Benditz & Felix Greimel | Scientific Reports | 2020 | 10.1038/s41598-020-74986-x | English |
| Does patellofemoral geometry in TKA affect patellar position in mid-flexion? | Mo SaffariniStefano ZaffagniniSimone BignozziFrancesca ColleMaurilio MarcacciDavid Dejour | Knee Surgery, Sports Traumatology, Arthroscopy | 2015 | 10.1007/s00167-015-3565-0 | English |
| Improved implant position and lower revision rate with robotic-assisted unicompartmental knee arthroplasty | Cécile Batailler, Nathan White, Filippo Maria Ranaldi, Philippe Neyret, Elvire Servien & Sébastien Lustig | Knee Surgery, Sports Traumatology, Arthroscopy | 2019 | 10.1007/s00167-018-5081-5 | English |
| A novel handheld robotic-assisted system for unicompartmental knee arthroplasty: surgical technique and early survivorship | Andrew K. BattenbergNathan A. NetravaliJess H. Lonner | Journal of Robotic Surgery | 2020 | 10.1007/s11701-018-00907-w | English |
| Robotic-assisted versus standard unicompartmental knee arthroplasty—evaluation of manuscript conflict of interests, funding, scientific quality and bibliometrics | Leonardo CavinattoMichael J. BronsonDarwin D. ChenCalin S. Moucha | International Orthopaedics | 2019 | 10.1007/s00264-018-4175-5 | English |
| Is bicompartmental knee arthroplasty more favourable to knee muscle strength and physical performance compared to total knee arthroplasty? | Jun Young ChungByoung-Hyun Min | Knee Surgery, Sports Traumatology, Arthroscopy | 2013 | 10.1007/s00167-013-2489-9 | English |
| ISS 2016 Annual meeting Paris, France: scientific paper presentations |  | Skeletal Radiology | 2016 | 10.1007/s00256-016-2400-0 | English |
| Clinical and quality-of-life outcomes of a combined synthetic scaffold and autogenous tissue graft procedure for articular cartilage repair in the knee | Fernando Martins Rosa, Julio Cesar Fernandes, Josée Delisle, Pierre Ranger, Mauro Batista Albano & Edmar Stieven Filho | Journal of Orthopaedic Surgery and Research | 2022 | 10.1186/s13018-022-03010-x | English |
| Obesity has no effect on outcomes following unicompartmental knee arthroplasty | Johannes F. PlateMarco A. AugartThorsten M. SeylerDaniel N. BraceyAneitra HoggardMichael AkbarRiyaz H. JinnahGary G. Poehling | Knee Surgery, Sports Traumatology, Arthroscopy | 2017 | 10.1007/s00167-015-3597-5 | English |
| Predictors of 90-Day All-Cause Morbidity, Mortality and Poor Functional Outcome Scores Following Elective Total Knee Arthroplasty in a High-Volume Setting: A Prospective Cohort Study | Vikram Indrajit ShahJavahir A. PachoreSachin UpadhyayKalpesh ShahAshish SethAmish KshatriyaJayesh PatilPranay GujjarMilan Kantesariya | Indian Journal of Orthopaedics | 2022 | 10.1007/s43465-021-00559-3 | English |
| The innervation of patella: anatomical and clinical study | G. MaralcanI. KuruS. IssiA.F. EsmerI. TekdemirD. Evcik | Surgical and Radiologic Anatomy | 2005 | 10.1007/s00276-005-0334-7 | English |
| Avoiding Complications with MPFL Reconstruction | Marvin K. SmithBrian C. WernerDavid R. Diduch | Current Reviews in Musculoskeletal Medicine | 2018 | 10.1007/s12178-018-9479-y | English |
| Outcomes of Bi-unicompartmental Versus Total Knee Arthroplasty for the Treatment of Medial and Lateral Knee Osteoarthritis: A Systematic Review and Meta-analysis of Comparative Studies | Michele MercurioGiorgio GaspariniFilippo FamiliariDavide CastioniOlimpio Galasso | Indian Journal of Orthopaedics | 2022 | 10.1007/s43465-022-00628-1 | English |
| Success of torsional correction surgery after failed surgeries for patellofemoral pain and instability | Peter M. StevensJeremy M. GilillandLucas A. AndersonJennifer B. MickelsonJenifer NielsonJoshua W. Klatt | Strategies in Trauma and Limb Reconstruction | 2014 | 10.1007/s11751-013-0181-8 | English |
| Results of medial patellofemoral ligament reconstruction compared with trochleoplasty plus individual extensor apparatus balancing in patellar instability caused by severe trochlear dysplasia: a systematic review and meta-analysis | Peter BalcarekStephan RehnNick R. HowellsJonathan D. EldridgeKeisuke KitaDavid DejourManfred NelitzIngo J. BankeDelphine LambrechtMarkus HardenTim Friede | Knee Surgery, Sports Traumatology, Arthroscopy | 2017 | 10.1007/s00167-016-4365-x | English |
| Creation of a specialist core curriculum for the European Society for Sports traumatology, Knee surgery and Arthroscopy (ESSKA) | Martin LindRomain SeilDavid DejourRoland BeckerJacques MenetreyMichael Ross | Knee Surgery, Sports Traumatology, Arthroscopy | 2020 | 10.1007/s00167-020-06210-2 | English |
| Rasch analysis of the Forgotten Joint Score in patients undergoing knee arthroplasty | Didier D. Niama NattaEmmanuel ThienpontAlexandre BredinGwenael SalaunChristine Detrembleur | Knee Surgery, Sports Traumatology, Arthroscopy | 2019 | 10.1007/s00167-018-5109-x | English |
| Derotational distal femoral osteotomy yields satisfactory clinical outcomes in pathological femoral rotation with failed medial patellofemoral ligament reconstruction | Yanwei CaoZhijun ZhangJiewei ShenGuanyang SongQiankun NiYue LiTong ZhengHui Zhang | Knee Surgery, Sports Traumatology, Arthroscopy | 2022 | 10.1007/s00167-021-06739-w | English |
| Robotic versus conventional primary total knee arthroplasty: clinical and radiological long-term results with a minimum follow-up of ten years | Kyu-Jin ChoJong-Keun SeonWon-Young JangChun-Gon ParkEun-Kyoo Song | International Orthopaedics | 2019 | 10.1007/s00264-018-4231-1 | English |
| The 3D analysis of the sagittal curvature of the femoral trochlea in the Chinese population | Jun WangBing YueYou WangMengning YanYiming Zeng | Knee Surgery, Sports Traumatology, Arthroscopy | 2012 | 10.1007/s00167-011-1679-6 | English |
| Bearing design influences short- to mid-term survivorship, but not functional outcomes following lateral unicompartmental knee arthroplasty: a systematic review | Joost A. BurgerLaura J. KleebladInger N. SiereveltWieger G. HorstmannPeter A. Nolte | Knee Surgery, Sports Traumatology, Arthroscopy | 2019 | 10.1007/s00167-019-05357-x | English |
| The sulcus deepening trochleoplasty—the Lyon’s procedure | David DeJourPaulo Saggin | International Orthopaedics | 2010 | 10.1007/s00264-009-0933-8 | English |
| Free Papers |  | Knee Surgery, Sports Traumatology, Arthroscopy | 2018 | 10.1007/s00167-018-4866-x | English |
| Scalpel can achieve better clinical outcomes compared with electric cautery in primary total knee arthroplasty: a comparison study | Wei LinYike DaiJinghui NiuGuangmin YangMing LiFei Wang | BMC Musculoskeletal Disorders | 2020 | 10.1186/s12891-020-03457-1 | English |
| High rate of recurrent patellar dislocation in skeletally immature patients: a long-term population-based study | Thomas L. SandersAyoosh PareekTimothy E. HewettMichael J. StuartDiane L. DahmAaron J. Krych | Knee Surgery, Sports Traumatology, Arthroscopy | 2018 | 10.1007/s00167-017-4505-y | English |
| Knee resurfacing with double unicompartimental arthroplasty: rationale, biomechanics, indications, surgical technique and outcomes | Sergio RomagnoliStefano PetrilloMatteo Marullo | Journal of Experimental Orthopaedics | 2021 | 10.1186/s40634-021-00402-6 | English |
| Complex patellofemoral reconstruction leads to improved physical and sexual activity in female patients suffering from chronic patellofemoral instability | Patricia M. LutzPhilipp W. WinklerMarco-Christopher RuppStephanie GeyerAndreas B. ImhoffMatthias J. Feucht | Knee Surgery, Sports Traumatology, Arthroscopy | 2021 | 10.1007/s00167-020-06340-7 | English |
| Intraarticular injection of microfragmented adipose tissue plus arthroscopy in isolated primary patellofemoral osteoarthritis is clinically effective and not affected by age, BMI, or stage of osteoarthritis | Michele VassoKatia CoronaLuigi CapassoGiuseppe ToroAlfredo Schiavone Panni | Journal of Orthopaedics and Traumatology | 2022 | 10.1186/s10195-022-00628-9 | English |
| Patellar chondral defects: a review of a challenging entity | George MouzopoulosCarlo BorbonRainer Siebold | Knee Surgery, Sports Traumatology, Arthroscopy | 2011 | 10.1007/s00167-011-1546-5 | English |
| Treatment of isolated patellofemoral osteoarthritis with lateral facetectomy plus Insall’s realignment procedure: long-term follow-up | Ferran Montserrat, Eduard Alentorn-Geli, Vicens León, Alberto Ginés-Cespedosa & Pau Rigol | Knee Surgery, Sports Traumatology, Arthroscopy | 2013 | 10.1007/s00167-013-2431-1 | English |
| Mini-midvastus versus medial parapatellar approach in total knee arthroplasty: difference in patient-reported outcomes measured with the Forgotten Joint Score | Wei LinJinghui NiuYike DaiGuangmin YangMing LiFei Wang | Journal of Orthopaedic Surgery and Research | 2020 | 10.1186/s13018-020-01869-2 | English |
| Outcomes and early revision rate after medial unicompartmental knee arthroplasty: prospective results from a non-designer single surgeon | Jonathan R. B. HuttAvtar SurHartej SurAine RingroseMark S. Rickman | BMC Musculoskeletal Disorders | 2018 | 10.1186/s12891-018-2099-2 | English |
| Evaluating willingness for surgery using the SMART Choice (Knee) patient prognostic tool for total knee arthroplasty: study protocol for a pragmatic randomised controlled trial | Yuxuan ZhouClaire WeedenLauren PattenMichelle DowseySamantha BunzliPeter ChoongChris Schilling | BMC Musculoskeletal Disorders | 2022 | 10.1186/s12891-022-05123-0 | English |
| Survival and clinical results of a modified 'crosse de hockey' procedure for chronic isolated patellofemoral joint osteoarthritis: mid-term follow-up | Hiroaki KanazawaYuichiro MaruyamaKatsuo ShitotoMinoru YokoyamaKazuo Kaneko | Journal of Orthopaedics and Traumatology | 2017 | 10.1007/s10195-016-0428-5 | English |
| Have the Causes of Revision for Total and Unicompartmental Knee Arthroplasties Changed During the Past Two Decades? | Gro S Dyrhovden, Stein Håkon L Lygre, Mona Badawy, Øystein Gøthesen, Ove Furnes | Clinical Orthopaedics and Related Research | 2017 | 10.1007/s11999-017-5316-7 | English |
| Removal of the infrapatella fat pad during total knee arthroplasty: does it affect patient outcomes? | Robert MoverleyDerfel WilliamsNikolaos BardakosRichard Field | International Orthopaedics | 2014 | 10.1007/s00264-014-2427-6 | English |
| Der femoropatellare Teilersatz | M. CoticP. ForkelUniv. Prof. Dr. A. B. Imhoff | Operative Orthopädie und Traumatologie | 2017 | 10.1007/s00064-016-0477-1 | German |

| **Table S5 Detailed bibliographies searched from Medline in the field of patellofemoral arthroplasty** | | | | | | |
| --- | --- | --- | --- | --- | --- | --- |
| **MEDLINE Number** | **Article Title** | **Authors** | **Journal** | **Publication Year** | **DOI** | **Language** |
| 19634736 | Patellar button dissociation in a mobile-bearing LCS patellofemoral joint arthroplasty. | Sreekumar, R; Subramanian, Sivaraman; Mohammed, Aslam | The journal of knee surgery | 2009 | 10.1055/s-0030-1247763 | English |
| 29721690 | Advances in Patellofemoral Arthroplasty. | Strickland, Sabrina M; Bird, Mackenzie L; Christ, Alexander B | Current reviews in musculoskeletal medicine | 2018 | 10.1007/s12178-018-9477-0 | English |
| 23203512 | Outcomes of total knee replacement after patellofemoral arthroplasty. | Hutt, Jonathan; Dodd, Matthew; Bourke, Henry; Bell, Jonathan | The journal of knee surgery | 2013 | 10.1055/s-0032-1329233 | English |
| 25382369 | Patellofemoral joint replacement, an evolving concept. | Borus, Todd; Brilhault, Jean; Confalonieri, Norberto; Johnson, Derek; Thienpont, Emmanuel | The Knee | 2014 | 10.1016/S0968-0160(14)50010-5 | English |
| 22310419 | Patellar polyethylene spinout after low-contact stress, high-congruity, mobile-bearing patellofemoral arthroplasty. | Amanatullah, Derek F; Jamali, Amir A | Orthopedics | 2012 | 10.3928/01477447-20120123-27 | English |
| 25382368 | Partial knee arthroplasty: patellofemoral arthroplasty and combined unicompartmental and patellofemoral arthroplasty implants--general considerations and indications, technique and clinical experience. | Benazzo, Francesco; Rossi, Stefano M P; Ghiara, Matteo | The Knee | 2014 | 10.1016/S0968-0160(14)50009-9 | English |
| 16006129 | Patellar clunk syndrome in patellofemoral arthroplasty--a case report. | Sringari, T; Maheswaran, S S | The Knee | 2005 | 10.1016/j.knee.2004.11.008 | English |
| 18372179 | Unique combination of patellofemoral joint arthroplasty with Osteochondral Autograft Transfer System (OATS) - a case series of six knees in five patients. | Unnithan, A; Jimulia, T; Mohammed, R; Learmonth, D J A | The Knee | 2008 | 10.1016/j.knee.2008.01.007 | English |
| 23102421 | Blood loss and transfusion rates following patellofemoral arthroplasty. | Courtney, Jonathan; Liebelt, David; Nett, Michael P; Cushner, Fred D | The Orthopedic clinics of North America | 2012 | 10.1016/j.ocl.2012.07.007 | English |
| 34883522 | [When nothing else works: patellofemoral joint arthroplasty].Wenn nichts mehr geht: Der patellofemorale Gelenkersatz. | Benignus, Christian; Meier, Malin; Best, Raymond; Beckmann, Johannes | Sportverletzung Sportschaden : Organ der Gesellschaft fur Orthopadisch-Traumatologische Sportmedizin | 2021 | 10.1055/a-1523-9937 | German |
| 29551276 | Femoral component rotation in patellofemoral joint replacement. | van Jonbergen, Hans-Peter W; Westerbeek, Robin E | The Knee | 2018 | 10.1016/j.knee.2018.02.007 | English |
| 20619661 | Medial patellofemoral ligament reconstruction for subluxating patellofemoral arthroplasty. | Carmont, M R; Crane, T; Thompson, P; Spalding, T | The Knee | 2011 | 10.1016/j.knee.2010.02.011 | English |
| 17079388 | Revision of a failed patellofemoral arthroplasty to a total knee arthroplasty. | Lonner, Jess H; Jasko, Jeff G; Booth, Robert E Jr | The Journal of bone and joint surgery. American volume | 2006 | 10.2106/JBJS.F.00282 | English |
| 30193976 | Patellofemoral joint replacement - Mean five year follow-up. | Ajnin, Serajdin; Buchanan, Donald; Arbuthnot, James; Fernandes, Richard | The Knee | 2018 | 10.1016/j.knee.2018.08.014 | English |
| 27891054 | Patellofemoral Joint Arthroplasty: Our Experience in Isolated Patellofemoral and Bicompartmental Arthritic Knees. | Sabatini, L; Schiro, M; Atzori, F; Ferrero, G; Masse, A | Clinical medicine insights. Arthritis and musculoskeletal disorders | 2016 | 10.4137/CMAMD.S40498 | English |
| 20004900 | Strain shielding in distal femur after patellofemoral arthroplasty under different activity conditions. | Meireles, Susana; Completo, Antonio; Antonio Simoes, Jose; Flores, Paulo | Journal of biomechanics | 2010 | 10.1016/j.jbiomech.2009.09.048 | English |
| 31500912 | The Clinical Outcome of Patellofemoral Arthroplasty vs Total Knee Arthroplasty in Patients Younger Than 55 Years. | Kamikovski, Ivan; Dobransky, Johanna; Dervin, Geoffrey F | The Journal of arthroplasty | 2019 | 10.1016/j.arth.2019.07.016 | English |
| 25861405 | Femoral component rotation in patellofemoral joint replacement: a study protocol for a prospective observational study. | Westerbeek, Robin E; Derks, Rosalie P H; Zonneveld, Bas J G L; van Jonbergen, Hans-Peter W | The open orthopaedics journal | 2015 | 10.2174/1874325001509010045 | English |
| 32659820 | Obesity Does Not Affect Patient-Reported Outcomes following Patellofemoral Arthroplasty. | Tishelman, Jared C; Pyne, Abigail; Kahlenberg, Cynthia A; Gruskay, Jordan A; Strickland, Sabrina M | The journal of knee surgery | 2022 | 10.1055/s-0040-1713862 | English |
| 28840268 | No bias for developer publications and no difference between first-generation trochlear-resurfacing versus trochlear-cutting implants in 15,306 cases of patellofemoral joint arthroplasty. | Reihs, Birgit; Reihs, Florian; Labek, Gerold; Hochegger, Markus; Leithner, Andreas; Bohler, Nikolaus; Sadoghi, Patrick | Knee surgery, sports traumatology, arthroscopy : official journal of the ESSKA | 2018 | 10.1007/s00167-017-4692-6 | English |
| 33532210 | Patellofemoral Replacement With Tibial Tubercle Osteotomy. | Arvesen, John E; Wyland, Douglas J | Arthroscopy techniques | 2021 | 10.1016/j.eats.2020.09.009 | English |
| 32115918 | [Patellofemoral arthroplasty for the treatment of patellofemoral arthritis]. | Gao, Hua-Li; Xiao, Lian-Bo; Zhang, Qian; Zhang, Jie-Chao; He, Yong; Zhai, Wei-Tao; Shi, Qi | Zhongguo gu shang = China journal of orthopaedics and traumatology | 2020 | 10.3969/j.issn.1003-0034.2020.01.003 | Chinese |
| 30662710 | The evolution and role of patellofemoral joint arthroplasty: The road less travelled, but not forgotten. | Roussot, M A; Haddad, F S | Bone & joint research | 2018 | 10.1302/2046-3758.712.BJR-2018-0303 | English |
| 30617612 | Return to the operating room after patellofemoral arthroplasty versus total knee arthroplasty for isolated patellofemoral arthritis-a systematic review. | Woon, Colin Y L; Christ, Alexander B; Goto, Rie; Shanaghan, Kate; Shubin Stein, Beth E; Gonzalez Della Valle, Alejandro | International orthopaedics | 2019 | 10.1007/s00264-018-04280-z | English |
| 36582575 | Functional Outcomes, Survival Rate, and Complications of Patellofemoral Arthroplasty: Mid-Term Results From Independent Center. | Jagadeesh, Nuthan; Sales-Fernandez, Rafael; Pammi, Srinath; Kariya, Ankur | Cureus | 2022 | 10.7759/cureus.31945 | English |
| 31495529 | Isolated Patellofemoral Joint Arthroplasty: Can Preoperative Bone Scans Predict Survivorship? | Baker, James F; Caborn, David N; Schlierf, Thomas J; Fain, Trevor B; Smith, Langan S; Malkani, Arthur L | The Journal of arthroplasty | 2020 | 10.1016/j.arth.2019.08.021 | English |
| 22704638 | Differences in the stress distribution in the distal femur between patellofemoral joint replacement and total knee replacement: a finite element study. | van Jonbergen, Hans-Peter W; Innocenti, Bernardo; Gervasi, Gian Luca; Labey, Luc; Verdonschot, Nico | Journal of orthopaedic surgery and research | 2012 | 10.1186/1749-799X-7-28 | English |
| 33355685 | [Patellofemoral inlay implants-an innovation in patellofemoral joint arthroplasty?]Patellofemorale Inlay-Implantate- ein Fortschritt in der patellofemoralen Endoprothetik? | Degenhardt, Hannes; Imhoff, Andreas B; Feucht, Matthias J; Pogorzelski, Jonas | Der Orthopade | 2021 | 10.1007/s00132-020-04059-4 | German |
| 33825970 | Midterm results of modern patellofemoral arthroplasty versus total knee arthroplasty for isolated patellofemoral arthritis: systematic review and meta-analysis of comparative studies. | Elbardesy, Hany; McLeod, Andre; Gul, Rehan; Harty, James | Archives of orthopaedic and trauma surgery | 2022 | 10.1007/s00402-021-03882-4 | English |
| 32178848 | Total knee arthroplasty reduces knee extension torque in-vitro and patellofemoral arthroplasty does not. | Joseph, Michelle N; Carmont, Michael R; Tailor, Hiteshkumar; Stephen, Joanna M; Amis, Andrew A | Journal of biomechanics | 2020 | 10.1016/j.jbiomech.2020.109739 | English |
| 32368460 | Minimally Invasive Robotic-Assisted Patellofemoral Arthroplasty. | Hassebrock, Jeffrey D; Makovicka, Justin L; Wong, Michael; Patel, Karan A; Scott, Kelly L; Deckey, David G; Chhabra, Anikar | Arthroscopy techniques | 2020 | 10.1016/j.eats.2019.11.013 | English |
| 32192817 | Aiming for anatomical femoral axis on the coronal plane leads to good-to-excellent short-term outcomes in isolated patellofemoral arthroplasty. | Vasta, Sebastiano; Rosi, Massimiliano; Tecame, Andrea; Papalia, Rocco; Adravanti, Paolo | The Knee | 2020 | 10.1016/j.knee.2020.02.016 | English |
| 32044787 | Femoropatellar Osteoarthritis and Trochlear Femoral Bone Defect due to Giant Cell Tumor of the Knee: A Selected Patellofemoral Joint Arthroplasty and Reconstructive Technique: A Case Report. | Conti, Andrea; Boffano, Michele; Pellegrino, Pietro; Ratto, Nicola; Sabatini, Luigi; Piana, Raimondo | JBJS case connector | 2020 | 10.2106/JBJS.CC.19.00378 | English |
| 25150332 | Isolated patellofemoral arthroplasty. | Oni, Julius K; Hochfelder, Jason; Dayan, Alan | Bulletin of the Hospital for Joint Disease (2013) | 2014 |  | English |
| 28861196 | Patellofemoral Joint Arthroplasty: Early Results and Functional Outcome of the Zimmer Gender Solutions Patello-Femoral Joint System. | Osarumwense, Donald; Syed, Farhan; Nzeako, Obi; Akilapa, Segun; Zubair, Omer; Waite, Jon | Clinics in orthopedic surgery | 2017 | 10.4055/cios.2017.9.3.295 | English |
| 24579096 | Mid-term results of the FPV patellofemoral joint replacement. | Al-Hadithy, N; Patel, R; Navadgi, B; Deo, S; Hollinghurst, D; Satish, V | The Knee | 2014 | 10.1016/j.knee.2013.08.010 | English |
| 15483797 | Early results with a total patellofemoral joint replacement arthroplasty prosthesis. | Merchant, Alan C | The Journal of arthroplasty | 2004 | 10.1016/j.arth.2004.03.011 | English |
| 20062723 | Patellofemoral arthroplasty for symptomatic nonunion after trochlear osteotomy for patellar instability: a case report. | van Jonbergen, Hans-Peter W; van Egmond, Kees | Cases journal | 2009 | 10.1186/1757-1626-2-9086 | English |
| 25154928 | [Patellofemoral arthroplasty: indication, technique and results].Patellotrochlearer Ersatz: Indikation, Technik und Ergebnisse. | Cotic, M; Imhoff, A B | Der Orthopade | 2014 | 10.1007/s00132-014-3006-7 | German |
| 35244809 | Patellofemoral arthroplasty: expert opinion. | Hoogervorst, Paul; Arendt, Elizabeth A | Journal of experimental orthopaedics | 2022 | 10.1186/s40634-022-00457-z | English |
| 19681331 | Dislocation of the mobile bearing component of a patellofemoral arthroplasty: a report of two cases. | Witjes, Suzanne; Van den Broek, Chris; Koeter, Sander; Van Loon, Corne | Acta orthopaedica Belgica | 2009 |  | English |
| 25382365 | Biomechanics of medial unicondylar in combination with patellofemoral knee arthroplasty. | Heyse, Thomas J; El-Zayat, Bilal F; De Corte, Ronny; Scheys, Lennart; Chevalier, Yan; Fuchs-Winkelmann, Susanne; Labey, Luc | The Knee | 2014 | 10.1016/S0968-0160(14)50002-6 | English |
| 33925287 | Treatment of Unicompartmental Cartilage Defects of the Knee with Unicompartmental Knee Arthroplasty, Patellofemoral Partial Knee Arthroplasty or Focal Resurfacing. | Springer, Bernhard; Boettner, Friedrich | Life (Basel, Switzerland) | 2021 | 10.3390/life11050394 | English |
| 32970202 | Pre-operative patella alta does not affect midterm clinical outcomes and survivorship of patellofemoral arthroplasty. | Bernard, Christopher D; Pareek, Ayoosh; Sabbag, Casey M; Parkes, Chad W; Krych, Aaron J; Cummings, Nancy M; Dahm, Diane L | Knee surgery, sports traumatology, arthroscopy : official journal of the ESSKA | 2021 | 10.1007/s00167-020-06205-z | English |
| 20021240 | Scientific evidence for the use of modern patellofemoral arthroplasty. | Gupta, Rishi R; Zywiel, Michael G; Leadbetter, Wayne B; Bonutti, Peter; Mont, Michael A | Expert review of medical devices | 2010 | 10.1586/erd.09.53 | English |
| 18602563 | Patellofemoral arthroplasty: the impact of design on outcomes. | Lonner, Jess H | The Orthopedic clinics of North America | 2008 | 10.1016/j.ocl.2008.02.002 | English |
| 15995422 | Outcome instruments for patellofemoral arthroplasty. | Paxton, Elizabeth W; Fithian, Donald C | Clinical orthopaedics and related research | 2005 | 10.1097/01.blo.0000171544.38095.77 | English |
| 23790605 | Clinical results of patellofemoral arthroplasty. | Morris, Michael J; Lombardi, Adolph V Jr; Berend, Keith R; Hurst, Jason M; Adams, Joanne B | The Journal of arthroplasty | 2013 | 10.1016/j.arth.2013.05.012 | English |
| 15995426 | The appropriate use of patellofemoral arthroplasty: an analysis of reported indications, contraindications, and failures. | Leadbetter, Wayne B; Ragland, Phillip S; Mont, Michael A | Clinical orthopaedics and related research | 2005 |  | English |
| 29475782 | Mid-term survivorship and clinical outcomes of the Avon patellofemoral joint replacement. | Middleton, S W F; Toms, A D; Schranz, P J; Mandalia, V I | The Knee | 2018 | 10.1016/j.knee.2018.01.007 | English |
| 20056375 | Long-term outcomes of patellofemoral arthroplasty. | van Jonbergen, Hans-Peter W; Werkman, Dirk M; Barnaart, Lex F; van Kampen, Albert | The Journal of arthroplasty | 2010 | 10.1016/j.arth.2009.08.023 | English |
| 19829980 | Dissociation of mobile-bearing patellar component in low contact stress patellofemoral arthroplasty, its mechanism and management: two case reports. | van Jonbergen, Hans-Peter W; Werkman, Dirk M; Barnaart, Alexander Fw | Cases journal | 2009 | 10.1186/1757-1626-2-7502 | English |
| 22111771 | The Warwick patellofemoral arthroplasty trial: a randomised clinical trial of total knee arthroplasty versus patellofemoral arthroplasty in patients with severe arthritis of the patellofemoral joint. | Odumenya, Michelle; McGuinness, Katie; Achten, Juul; Parsons, Nick; Spalding, Tim; Costa, Matthew | BMC musculoskeletal disorders | 2011 | 10.1186/1471-2474-12-265 | English |
| 19057900 | Patellofemoral arthroplasty: a multi-centre study with minimum 2-year follow-up. | Leadbetter, Wayne B; Kolisek, Frank R; Levitt, Richard L; Brooker, Andrew F; Zietz, Patrick; Marker, David R; Bonutti, Peter M; Mont, Michael A | International orthopaedics | 2009 | 10.1007/s00264-008-0692-y | English |
| 12905797 | [Patellofemoral replacement for severe patellofemoral osteoarthritis: a 2-10 years follow-up study]. | Zhang, Jia; Ye, Qi-bin; Qiu, Gui-xing; Wang, Yi-peng | Zhongguo yi xue ke xue yuan xue bao. Acta Academiae Medicinae Sinicae | 2002 |  | Chinese |
| 18602564 | Patellofemoral arthroplasty with a customized trochlear prosthesis. | Sisto, Domenick J; Sarin, Vineet K | The Orthopedic clinics of North America | 2008 | 10.1016/j.ocl.2008.03.002 | English |
| 23827831 | The clinical outcome of patellofemoral arthroplasty. | Lonner, Jess H; Bloomfield, Michael R | The Orthopedic clinics of North America | 2013 | 10.1016/j.ocl.2013.03.002 | English |
| 26790802 | Outcome of patellofemoral arthroplasty, determinants for success. | Willekens, Philippe; Victor, Jan; Verbruggen, Dimitri; Vande Kerckhove, Michiel; Van Der Straeten, Catherine | Acta orthopaedica Belgica | 2015 |  | English |
| 7497663 | Is there a place for patellofemoral arthroplasty? | Argenson, J N; Guillaume, J M; Aubaniac, J M | Clinical orthopaedics and related research | 1995 |  | English |
| 18534464 | Revision patellofemoral arthroplasty: three- to seven-year follow-up. | Hendrix, Michiel R G; Ackroyd, Christopher E; Lonner, Jess H | The Journal of arthroplasty | 2008 | 10.1016/j.arth.2007.10.019 | English |
| 26543657 | Patellofemoral Joint Replacement and Nickel Allergy: An Unusual Presentation. | Syed, Farhan; Jenner, Edward; Faisal, Mohammad | Case reports in orthopedics | 2015 | 10.1155/2015/635082 | English |
| 19085728 | [Patellofemoral arthroplasty--results of a nation-wide survey in Germany and review of the literature].Patellofemorale Endoprothetik in Deutschland - eine Standortbestimmung und Uberblick uber die Literatur. | Becher, C; Renke, A; Heyse, T J; Schofer, M; Tibesku, C O; Fuchs-Winkelmann, S | Zeitschrift fur Orthopadie und Unfallchirurgie | 2008 | 10.1055/s-2008-1039000 | German |
| 29628679 | Patellofemoral arthroplasty: Current concepts. | Cuthbert, Rory; Tibrewal, Saket; Tibrewal, Sheo B | Journal of clinical orthopaedics and trauma | 2018 | 10.1016/j.jcot.2017.11.006 | English |
| 15991118 | Patellofemoral arthroplasty: the third compartment. | Lotke, Paul A; Lonner, Jess H; Nelson, Charles L | The Journal of arthroplasty | 2005 | 10.1016/j.arth.2005.03.011 | English |
| 21964234 | Patellofemoral arthroplasty: 7-year mean follow-up. | Mont, Michael A; Johnson, Aaron J; Naziri, Qais; Kolisek, Frank R; Leadbetter, Wayne B | The Journal of arthroplasty | 2012 | 10.1016/j.arth.2011.07.010 | English |
| 18979927 | Patient-based outcomes in patellofemoral arthroplasty. | Utukuri, M M; Khanduja, V; Somayaji, H S; Dowd, G S E | The journal of knee surgery | 2008 | 10.1055/s-0030-1247830 | English |
| 21162949 | A preliminary report of patellofemoral arthroplasty in isolated patellofemoral arthritis. | Gao, Xiang; Xu, Zheng-Jian; He, Rong-Xin; Yan, Shi-Gui; Wu, Li-Dong | Chinese medical journal | 2010 |  | English |
| 30315337 | Patellar tendon shortening following patellofemoral joint replacement. | van Engen, Ludo A H; Landman, Ellie B M; Kleinlugtenbelt, Ydo V; van Jonbergen, Hans-Peter W | International orthopaedics | 2019 | 10.1007/s00264-018-4194-2 | English |
| 24426856 | Computer-assisted navigation in patellofemoral arthroplasty: a new technique to improve rotational position of the trochlea. | Hernigou, Philippe; Flouzat-Lachaniette, Charles Henri; Delblond, William; Duffiet, Pascal; Julian, Didier | HSS journal : the musculoskeletal journal of Hospital for Special Surgery | 2013 | 10.1007/s11420-013-9328-x | English |
| 35236486 | Patient-related outcomes of patellofemoral arthroplasty: experience of a single center. | Abeysekera, W Y M; Schenk, W | Arthroplasty (London, England) | 2021 | 10.1186/s42836-021-00074-8 | English |
| 17664369 | Patellofemoral arthroplasty. | Lonner, Jess H | The Journal of the American Academy of Orthopaedic Surgeons | 2007 | 10.5435/00124635-200708000-00006 | English |
| 20415369 | Patellofemoral arthroplasty. | Lonner, Jess H | Instructional course lectures | 2010 |  | English |
| 26908975 | Patellofemoral arthroplasty in a bilateral Syme's amputee. | Kanna, Raj; Barrett, David S | Journal of clinical orthopaedics and trauma | 2016 | 10.1016/j.jcot.2015.09.004 | English |
| 18078881 | Ipsilateral patellofemoral arthroplasty and autogenous osteochondral femoral condylar transplantation. | Lonner, Jess H; Mehta, Samir; Booth, Robert E Jr | The Journal of arthroplasty | 2007 | 10.1016/j.arth.2005.08.012 | English |
| 19949120 | The Avon patellofemoral joint replacement: independent assessment of early functional outcomes. | Starks, I; Roberts, S; White, S H | The Journal of bone and joint surgery. British volume | 2009 | 10.1302/0301-620X.91B12.23018 | English |
| 33086962 | Combined patellofemoral arthroplasty and medial patellofemoral ligament reconstruction for chronic patellar instability with trochlear dysplasia: a report of two cases. | Yamagami, Ryota; Inui, Hiroshi; Taketomi, Shuji; Tanaka, Sakae | Modern rheumatology case reports | 2020 | 10.1080/24725625.2019.1638048 | English |
| 16239783 | Patellofemoral arthroplasty: an update. | Argenson, Jean-Noel A; Flecher, Xavier; Parratte, Sebastien; Aubaniac, Jean-Manuel | Clinical orthopaedics and related research | 2005 | 10.1097/01.blo.0000187061.27573.70 | English |
| 33214935 | Erratum: Patellofemoral Arthroplasty. | [Anonymous] | JBJS essential surgical techniques | 2020 | 10.2106/JBJS.ST.ER.18.00094 | English |
| 18722775 | Optimizing patellofemoral arthroplasty. | Farr, Jack 2nd; Barrett, David | The Knee | 2008 | 10.1016/j.knee.2008.05.008 | English |
| 20406477 | Distal femoral bone mineral density decreases following patellofemoral arthroplasty: 1-year follow-up study of 14 patients. | van Jonbergen, Hans-Peter W; Koster, Kenneth; Labey, Luc; Innocenti, Bernardo; van Kampen, Albert | BMC musculoskeletal disorders | 2010 | 10.1186/1471-2474-11-74 | English |
| 19234887 | Conversion of patellofemoral arthroplasty to total knee arthroplasty: A matched case-control study of 13 patients. | van Jonbergen, Hans-Peter W; Werkman, Dirk M; van Kampen, Albert | Acta orthopaedica | 2009 | 10.1080/17453670902805031 | English |
| 12925646 | Patellofemoral arthroplasty. | Smith, Andrew; Lucas, Duncan | The Journal of bone and joint surgery. American volume | 2003 | 10.2106/00004623-200308000-00029 | English |
| 27131405 | The Journey patellofemoral joint arthroplasty: A minimum 5year follow-up study. | Ahearn, Nathanael; Metcalfe, Andrew J; Hassaballa, Mohammed A; Porteous, Andrew J; Robinson, James R; Murray, James R; Newman, John H | The Knee | 2016 | 10.1016/j.knee.2016.03.004 | English |
| 30275797 | Patellofemoral Arthroplasty. | Godshaw, Brian; Kolodychuk, Nicholas; Williams, Gerard K Jr; Browning, Benjamin; Jones, Deryk | The Ochsner journal | 2018 | 10.31486/toj.18.0009 | English |
| 23395041 | Patellofemoral arthroplasty: the other unicompartmental knee replacement. | Walker, Torrance; Perkinson, Brian; Mihalko, William M | Instructional course lectures | 2013 |  | English |
| 25023661 | Patellofemoral arthroplasty influences tibiofemoral kinematics: the effect of patellar thickness. | Vandenneucker, Hilde; Labey, Luc; Victor, Jan; Vander Sloten, Jos; Desloovere, Kaat; Bellemans, Johan | Knee surgery, sports traumatology, arthroscopy : official journal of the ESSKA | 2014 | 10.1007/s00167-014-3160-9 | English |
| 24460149 | Biomechanical behaviour of cancellous bone on patellofemoral arthroplasty with Journey prosthesis: a finite element study. | Castro, A P G; Completo, Antonio; Simoes, Jose A; Flores, Paulo | Computer methods in biomechanics and biomedical engineering | 2015 | 10.1080/10255842.2013.870999 | English |
| 24412044 | Patellofemoral arthroplasty. | Lustig, S | Orthopaedics & traumatology, surgery & research : OTSR | 2014 | 10.1016/j.otsr.2013.06.013 | English |
| 11922380 | The Lubinus patellofemoral arthroplasty: a five- to ten-year prospective study. | Roach, R | The Journal of bone and joint surgery. British volume | 2002 | 10.1302/0301-620X.84B2.0840307 | English |
| 25660614 | Four-Year Follow Up Outcome Study of Patellofemoral Arthroplasty at a Single Institution. | Goh, Graham Seow-Hng; Liow, Ming Han Lincoln; Tay, Darren Keng-Jin; Lo, Ngai Nung; Yeo, Seng Jin | The Journal of arthroplasty | 2015 | 10.1016/j.arth.2015.01.020 | English |
| 34513103 | Total Hip Arthroplasty Concomitant with Patellofemoral Arthroplasty and Medial Patellofemoral Ligament Reconstruction for a Patient with Patellar Dislocation Combined with Hip Dysplasia: A Case Report of a Successful Outcome at 5-Year Follow-Up. | Iseki, Takuya; Iseki, Tomoya; Okahisa, Shohei; Yoshiya, Shinichi; Fukunishi, Shigeo; Tachibana, Toshiya | Case reports in orthopedics | 2021 | 10.1155/2021/9970975 | English |
| 36507211 | Robotic Assisted Patellofemoral Joint Replacement: Surgical Technique, Tips and Tricks. | Selvaratnam, Veenesh; Toms, Andrew D; Mandalia, Vipul I | Indian journal of orthopaedics | 2022 | 10.1007/s43465-022-00746-w | English |
| 19897372 | Unusual mechanical complications of unicompartmental low contact stress mobile bearing patellofemoral arthroplasty: a cause for concern? | Arumilli, B R B; Ng, A B Y; Ellis, D J; Hirst, P | The Knee | 2010 | 10.1016/j.knee.2009.10.006 | English |
| 11922379 | The Lubinus patellofemoral arthroplasty: a five- to ten-year prospective study. | Coleridge, S | The Journal of bone and joint surgery. British volume | 2002 | 10.1302/0301-620X.84B2.0840306a | English |
| 32147094 | Erratum to The midterm results of a cohort study of patellofemoral arthroplasty from a non-designer center using an asymmetric trochlear prosthesis [Knee 26 (2019) 1348-1353]. | Rammohan, R; Gupta, Shreekant; Lee, Paul Y F; Chandratreya, Amit | The Knee | 2020 | 10.1016/j.knee.2020.02.009 | English |
| 17142442 | Indications, contraindications, and pitfalls of patellofemoral arthroplasty. | Leadbetter, Wayne B; Seyler, Thorsten M; Ragland, Phillip S; Mont, Michael A | The Journal of bone and joint surgery. American volume | 2006 | 10.2106/JBJS.F.00856 | English |
| 24187369 | Patellofemoral replacement: the third compartment. | Hofmann, A A; McCandless, J B; Shaeffer, J F; Magee, T H | The bone & joint journal | 2013 | 10.1302/0301-620X.95B11.32985 | English |
| 33835108 | Letter to the Editor: Short-term Revision Risk of Patellofemoral Arthroplasty is High: An Analysis From Eight Large Arthroplasty Registries. | van Jonbergen, Hans-Peter W; Kleinlugtenbelt, Ydo V | Clinical orthopaedics and related research | 2021 | 10.1097/CORR.0000000000001717 | English |
| 24993415 | Patellofemoral arthroplasty in the athlete. | Farr, Jack; Arendt, Elizabeth; Dahm, Diane; Daynes, Jake | Clinics in sports medicine | 2014 | 10.1016/j.csm.2014.03.003 | English |
| 33835097 | Reply to the Letter to the Editor: Short-term Revision Risk of Patellofemoral Arthroplasty is High: An Analysis From Eight Large Arthroplasty Registries. | Lewis, Peter L | Clinical orthopaedics and related research | 2021 | 10.1097/CORR.0000000000001718 | English |
| 22009560 | In vivo sagittal plane kinematics of the FPV patellofemoral replacement. | Monk, A P; van Duren, B H; Pandit, H; Shakespeare, D; Murray, D W; Gill, H S | Knee surgery, sports traumatology, arthroscopy : official journal of the ESSKA | 2012 | 10.1007/s00167-011-1717-4 | English |
| 24190590 | Design, operative technique and ten-year results of the Hermes patellofemoral arthroplasty. | Hernigou, Philippe; Philippe, Hernigou; Caton, Jacques | International orthopaedics | 2014 | 10.1007/s00264-013-2158-0 | English |
| 33438913 | Partial Knee Arthroplasty: The State of the Art. | Fuller, Brian C; Lonner, Jess H; Berend, Keith R; Berger, Richard A; Gerlinger, Tad L | Instructional course lectures | 2021 |  | English |
| 18602566 | Results of total knee replacement for isolated patellofemoral arthritis: when not to perform a patellofemoral arthroplasty. | Delanois, Ronald E; McGrath, Mike S; Ulrich, Slif D; Marker, David R; Seyler, Thorsten M; Bonutti, Peter M; Mont, Michael A | The Orthopedic clinics of North America | 2008 | 10.1016/j.ocl.2008.03.003 | English |
| 36519003 | A Newly Identified Complication of Patellofemoral Arthroplasty: Case Report and Literature Review. | Solarino, Giuseppe; Maccagnano, Giuseppe; Vicenti, Giovanni; Buono, Claudio; Simone, Filippo; Ottaviani, Guglielmo; Zavattini, Giacomo; Zaccari, Domenico; Carrozzo, Massimiliano; Spinarelli, Antonio; Bizzoca, Davide; Moretti, Biagio | Geriatric orthopaedic surgery & rehabilitation | 2022 | 10.1177/21514593221138662 | English |
| 17356140 | The Avon patellofemoral arthroplasty: five-year survivorship and functional results. | Ackroyd, C E; Newman, J H; Evans, R; Eldridge, J D J; Joslin, C C | The Journal of bone and joint surgery. British volume | 2007 | 10.1302/0301-620X.89B3.18062 | English |
| 8804284 | Patellofemoral arthroplasty. A 2- to 18-year followup study. | Krajca-Radcliffe, J B; Coker, T P | Clinical orthopaedics and related research | 1996 |  | English |
| 25382370 | Coronal alignment of patellofemoral arthroplasty. | Thienpont, Emmanuel; Lonner, Jess H | The Knee | 2014 | 10.1016/S0968-0160(14)50011-7 | English |
| 33937460 | Tibial Osteolysis After Long-Term Isolated Polyethylene Patellar Resurfacing. | Apple, Andrew E; Montgomery, Corey O; Mears, Simon C | Arthroplasty today | 2021 | 10.1016/j.artd.2021.03.012 | English |
| 14503763 | Patellofemoral replacement: the third compartment. | Argenson, Jean-Noel | Orthopedics | 2003 | 10.3928/0147-7447-20030901-32 | English |
| 20839682 | Patellofemoral arthroplasty. | Lonner, Jess H | Orthopedics | 2010 | 10.3928/01477447-20100722-39 | English |
| 21464487 | The low contact stress patellofemoral replacement: high early failure rate. | Charalambous, C P; Abiddin, Z; Mills, S P; Rogers, S; Sutton, P; Parkinson, R | The Journal of bone and joint surgery. British volume | 2011 | 10.1302/0301-620X.93B4.25899 | English |
| 31791722 | The midterm results of a cohort study of patellofemoral arthroplasty from a non-designer centre using an asymmetric trochlear prosthesis. | Rammohan, R; Gupta, Shreekant; Lee, Paul Y F; Chandratreya, Amit | The Knee | 2019 | 10.1016/j.knee.2019.10.026 | English |
| 29129614 | Mid-Term Clinical, Functional, and Radiographic Outcomes of 105 Gender-Specific Patellofemoral Arthroplasties, With or Without the Association of Medial Unicompartmental Knee Arthroplasty. | Romagnoli, Sergio; Marullo, Matteo | The Journal of arthroplasty | 2018 | 10.1016/j.arth.2017.10.019 | English |
| 25381467 | Isolated patellofemoral arthroplasty reproduces natural patellofemoral joint kinematics when the patella is resurfaced. | Vandenneucker, Hilde; Labey, Luc; Vander Sloten, Jos; Desloovere, Kaat; Bellemans, Johan | Knee surgery, sports traumatology, arthroscopy : official journal of the ESSKA | 2016 | 10.1007/s00167-014-3415-5 | English |
| 27193203 | The effect of axial rotation of the anterior resection plane in patellofemoral arthroplasty. | Cho, K J; Erasmus, P J; Muller, J H | The Knee | 2016 | 10.1016/j.knee.2016.04.006 | English |
| 16627153 | Computer-assisted patellofemoral arthroplasty: a mechanism for optimizing rotation. | Cossey, A J; Spriggins, A J | The Journal of arthroplasty | 2006 | 10.1016/j.arth.2005.08.010 | English |
| 22407183 | Patellofemoral arthroplasty, where are we today? | Lustig, Sebastien; Magnussen, Robert A; Dahm, Diane L; Parker, David | Knee surgery, sports traumatology, arthroscopy : official journal of the ESSKA | 2012 | 10.1007/s00167-012-1948-z | English |
| 17975374 | Total knee arthroplasty for isolated patellofemoral arthritis in younger patients. | Meding, John B; Wing, Jennifer T; Keating, E Michael; Ritter, Merrill A | Clinical orthopaedics and related research | 2007 | 10.1097/BLO.0b013e3181576069 | English |
| 16769215 | Arthritis progression after patellofemoral joint replacement. | Nicol, Steve G; Loveridge, Jeremy M; Weale, A E; Ackroyd, C E; Newman, J H | The Knee | 2006 | 10.1016/j.knee.2006.04.005 | English |
| 10427798 | [Femoro-patellar prosthesis. A retrospective study of 45 consecutive cases with a follow-up of 3-12 years].Les protheses femoro-patellaires. Etude retrospective de 45 cas successifs avec un recul de 3 a 12 ans. | De Cloedt, P; Legaye, J; Lokietek, W | Acta orthopaedica Belgica | 1999 |  | French |
| 28594500 | Patellofemoral Arthroplasty: An Evolving Science. | Lonner, Jess H | Instructional course lectures | 2017 |  | English |
| 18814615 | Patellofemoral replacement: the third compartment. | Minas, Tom | Orthopedics | 2008 | 10.3928/01477447-20080901-24 | English |
| 2319248 | Patellofemoral arthroplasty. 2-12-year follow-up study. | Cartier, P; Sanouiller, J L; Grelsamer, R | The Journal of arthroplasty | 1990 | 10.1016/S0883-5403(06)80009-4 | English |
| 17272470 | Custom patellofemoral replacement in the presence of trochlear dysplasia. | Grelsamer, Ronald P | The Journal of bone and joint surgery. American volume | 2007 | 10.2106/00004623-200702000-00040 | English |
| 15995413 | Patellofemoral replacement. 1979. | Blazina, Martin E; Fox, James M; Del Pizzo, Wilson; Broukhim, Behrooz; Ivey, F Martin | Clinical orthopaedics and related research | 2005 |  | English |
| 26483259 | Significant Functional Improvement at 2 Years After Isolated Patellofemoral Arthroplasty With an Onlay Trochlear Implant, But Low Mental Health Scores Predispose to Dissatisfaction. | Kazarian, Gregory S; Tarity, T David; Hansen, Erik N; Cai, Jenny; Lonner, Jess H | The Journal of arthroplasty | 2016 | 10.1016/j.arth.2015.08.033 | English |
| 32766389 | The Present Situation of Patellofemoral Arthroplasty in the Management of Solitary Patellofemoral Osteoarthritis. | Rodriguez-Merchan, E Carlos | The archives of bone and joint surgery | 2020 | 10.22038/ABJS.2019.14125 | English |
| 32444231 | Patellofemoral Arthroplasty Surgical Technique: Lateral or Medial Parapatellar Approach. | Jeong, Stacy H; Schneider, Brandon; Pyne, Abigail S; Tishelman, Jared C; Strickland, Sabrina M | The Journal of arthroplasty | 2020 | 10.1016/j.arth.2020.04.026 | English |
| 15534537 | Patellofemoral arthroplasty: pros, cons, and design considerations. | Lonner, Jess H | Clinical orthopaedics and related research | 2004 |  | English |
| 17197318 | In vivo sagittal plane kinematics of the Avon patellofemoral arthroplasty. | Hollinghurst, David; Stoney, James; Ward, Thomas; Pandit, Hemant; Beard, David; Murray, David W | The Journal of arthroplasty | 2007 | 10.1016/j.arth.2006.02.160 | English |
| 30732508 | Patient satisfaction reporting for patellofemoral arthroplasty is significantly lacking: a systematic review. | Tishelman, Jared Christopher; Kahlenberg, Cynthia A; Nwachukwu, Benedict U; Gruskay, Jordan; Strickland, Sabrina M | The Physician and sportsmedicine | 2019 | 10.1080/00913847.2019.1580913 | English |
| 15995418 | A modular prosthesis for patellofemoral arthroplasty: design and initial results. | Merchant, Alan C | Clinical orthopaedics and related research | 2005 | 10.1097/01.blo.0000171917.47869.6c | English |
| 21489801 | Functional relevance of patellofemoral thickness before and after unicompartmental patellofemoral replacement. | Mofidi, A; Bajada, S; Holt, M D; Davies, A P | The Knee | 2012 | 10.1016/j.knee.2011.03.002 | English |
| 26590562 | Survivorship and functional outcomes of patellofemoral arthroplasty: a systematic review. | van der List, J P; Chawla, H; Zuiderbaan, H A; Pearle, A D | Knee surgery, sports traumatology, arthroscopy : official journal of the ESSKA | 2017 | 10.1007/s00167-015-3878-z | English |
| 25737384 | Patellar Fracture Following Patellofemoral Arthroplasty. | King, Alexander H; Engasser, William M; Sousa, Paul L; Arendt, Elizabeth A; Dahm, Diane L | The Journal of arthroplasty | 2015 | 10.1016/j.arth.2015.02.007 | English |
| 28271370 | Inadequacy of computed tomography for pre-operative planning of patellofemoral arthroplasty. | Saffarini, Mo; Muller, Jacobus H; La Barbera, Giuseppe; Hannink, Gerjon; Cho, Kyung Jin; Toanen, Cecile; Dejour, David | Knee surgery, sports traumatology, arthroscopy : official journal of the ESSKA | 2018 | 10.1007/s00167-017-4474-1 | English |
| 22704031 | Trochlear inclination angles in normal and dysplastic knees. | Kamath, Atul F; Slattery, Thomas R; Levack, Ashley E; Wu, Chia H; Kneeland, J Bruce; Lonner, Jess H | The Journal of arthroplasty | 2013 | 10.1016/j.arth.2012.04.017 | English |
| 28793978 | Patellofemoral arthroplasty conversion to total knee arthroplasty: Retrieval analysis and clinical correlation. | Christ, Alexander B; Baral, Elexis; Koch, Chelsea; Shubin Stein, Beth E; Gonzalez Della Valle, Alejandro; Strickland, Sabrina M | The Knee | 2017 | 10.1016/j.knee.2017.06.015 | English |
| 33795175 | Onlay Patellofemoral Arthroplasty in Patients With Isolated Patellofemoral Arthritis: A Systematic Review. | Villa, Jordan C; Paoli, Albit R; Nelson-Williams, Howard W; Badr, Rhamee N; Harper, Katharine D | The Journal of arthroplasty | 2021 | 10.1016/j.arth.2021.02.054 | English |
| 17768216 | Custom patellofemoral arthroplasty of the knee. Surgical technique. | Sisto, Domenick J; Sarin, Vineet K | The Journal of bone and joint surgery. American volume | 2007 | 10.2106/00004623-200702000-00041 | English |
| 31136443 | CORR Insights: What Is the Risk of Repeat Revision When Patellofemoral Replacement Is Revised to TKA? An Analysis of 482 Cases From a Large National Arthroplasty Registry. | Hallstrom, Brian R | Clinical orthopaedics and related research | 2019 | 10.1097/CORR.0000000000000583 | English |
| 32246606 | [Functional results in isolated patellofemoral arthrosis treated by arthroscopy plus Fulkerson osteotomy vs patellofemoral arthroplasty].Resultados funcionales en artrosis patelofemoral aislada tratados mediante artroscopia mas osteotomia de Fulkerson vs artroplastia patelofemoral. | Martinez-Sanchez, J A; Blanco-Bucio, P; Valencia-Martinez, G | Acta ortopedica mexicana | 2019 |  | Spanish |
| 29309448 | Return to Activities After Patellofemoral Arthroplasty. | Shubin Stein, Beth E; Brady, Jacqueline M; Grawe, Brian; Tuakli-Wosornu, Yetsa; Nguyen, Joseph T; Wolfe, Elizabeth; Voigt, Marcia; Mahony, Gregory; Strickland, Sabrina | American journal of orthopedics (Belle Mead, N.J.) | 2017 |  | English |
| 17142450 | Patellofemoral replacement polymer stress during daily activities: a finite element study. | Morra, Edward A; Greenwald, A Seth | The Journal of bone and joint surgery. American volume | 2006 | 10.2106/JBJS.F.00585 | English |
| 24426854 | MRI after patellofemoral replacement: the component-bone interface and rotational alignment. | Heyse, Thomas J; Figiel, Jens; Hahnlein, Ulrike; Timmesfeld, Nina; Schofer, Markus D; Fuchs-Winkelmann, Susanne; Efe, Turgay | HSS journal : the musculoskeletal journal of Hospital for Special Surgery | 2013 | 10.1007/s11420-013-9336-x | English |
| 21886926 | Medium term results of Avon patellofemoral joint replacement. | Sarda, Praveen K; Shetty, Anup; Maheswaran, Shanmuga S | Indian journal of orthopaedics | 2011 | 10.4103/0019-5413.83761 | English |
| 26151039 | Bi-unicompartmental and combined uni plus patellofemoral replacement: indications and surgical technique. | Romagnoli, Sergio; Marullo, Matteo; Massaro, Michele; Rustemi, Enis; D'Amario, Federico; Corbella, Michele | Joints | 2015 |  | English |
| 28735802 | Correction of Patellofemoral Malalignment With Patellofemoral Arthroplasty. | Valoroso, Marco; Saffarini, Mo; La Barbera, Giuseppe; Toanen, Cecile; Hannink, Gerjon; Nover, Luca; Dejour, David H | The Journal of arthroplasty | 2017 | 10.1016/j.arth.2017.06.048 | English |
| 36317169 | Patellofemoral arthroplasty in combination with high tibial osteotomy can achieve good outcome for patients with medial-patellofemoral osteoarthritis. | Peng, Yonggang; Lin, Wei; Zhang, Yufeng; Wang, Fei | Frontiers in surgery | 2022 | 10.3389/fsurg.2022.999208 | English |
| 17824336 | Patellofemoral arthritis and its management with isolated patellofemoral replacement: a personal experience. | Newman, John H | Orthopedics | 2007 |  | English |
| 11728076 | The Richards type II patellofemoral arthroplasty: 26 cases followed for 1-20 years. | de Winter, W E; Feith, R; van Loon, C J | Acta orthopaedica Scandinavica | 2001 | 10.1080/000164701753532826 | English |
| 12931801 | Long-term results of patellofemoral arthroplasty. A report of 56 arthroplasties with 17 years of follow-up. | Kooijman, H J; Driessen, A P P M; van Horn, J R | The Journal of bone and joint surgery. British volume | 2003 |  | English |
| 19224212 | Midterm clinical results of the Autocentric II patellofemoral prosthesis. | van Wagenberg, J M F; Speigner, B; Gosens, T; de Waal Malefijt, J | International orthopaedics | 2009 | 10.1007/s00264-009-0719-z | English |
| 30721343 | Early outcomes of an anatomic trochlear-cutting patellofemoral arthroplasty: patient selection is key. | Dejour, David; Saffarini, Mo; Malemo, Yves; Pungitore, Marco; Valluy, Jeremy; Nover, Luca; Demey, Guillaume | Knee surgery, sports traumatology, arthroscopy : official journal of the ESSKA | 2019 | 10.1007/s00167-019-05368-8 | English |
| 27979409 | Outcomes of Patellofemoral Arthroplasty Based on RadiographicSeverity. | deDeugd, Casey M; Pareek, Ayoosh; Krych, Aaron J; Cummings, Nancy M; Dahm, Diane L | The Journal of arthroplasty | 2017 | 10.1016/j.arth.2016.11.006 | English |
| 19301809 | Patellofemoral arthroplasty with a custom-fit femoral prosthesis. | Butler, James E; Shannon, Robert | Orthopedics | 2009 |  | English |
| 34078392 | The short-term effectiveness and safety of second-generation patellofemoral arthroplasty and total knee arthroplasty on isolated patellofemoral osteoarthritis: a systematic review and meta-analysis. | Li, Chengxin; Li, Zhizhuo; Shi, Lijun; Gao, Fuqiang; Sun, Wei | Journal of orthopaedic surgery and research | 2021 | 10.1186/s13018-021-02509-z | English |
| 23376989 | Incidence of symptomatic thromboembolic disease after patellofemoral arthroplasty. | Levack, Ashley; Kamath, Atul F; Lonner, Jess H | American journal of orthopedics (Belle Mead, N.J.) | 2012 |  | English |
| 17323632 | Outcomes of patellofemoral replacement in total knee arthroplasty using meticulous techniques. | Clyburn, Terry A; Weitz-Marshall, Amanda; Ambrose, Catherine M; Ursua, Victor | Orthopedics | 2007 | 10.3928/01477447-20070201-13 | English |
| 30601045 | Patellofemoral arthroplasty versus total knee arthroplasty for patients with patellofemoral osteoarthritis: equal function and satisfaction but higher revision rate for partial arthroplasty at a minimum eight years follow-up. | Clement, N D; Howard, T A; Immelman, R J; MacDonald, D; Patton, J T; Lawson, G M; Burnett, R | The bone & joint journal | 2019 | 10.1302/0301-620X.101B1.BJJ-2018-0654.R2 | English |
| 18602565 | Patellofemoral arthroplasty in the treatment of patellofemoral arthritis: rationale and outcomes in younger patients. | Leadbetter, Wayne B | The Orthopedic clinics of North America | 2008 | 10.1016/j.ocl.2008.04.001 | English |
| 26231153 | A matched-pair comparison of inlay and onlay trochlear designs for patellofemoral arthroplasty: no differences in clinical outcome but less progression of osteoarthritis with inlay designs. | Feucht, Matthias J; Cotic, Matthias; Beitzel, Knut; Baldini, Julia F; Meidinger, Gebhart; Schottle, Philip B; Imhoff, Andreas B | Knee surgery, sports traumatology, arthroscopy : official journal of the ESSKA | 2017 | 10.1007/s00167-015-3733-2 | English |
| 22547249 | Prospective clinical and radiological two-year results after patellofemoral arthroplasty using an implant with an asymmetric trochlea design. | Beitzel, Knut; Schottle, Philip B; Cotic, Matthias; Dharmesh, Vyas; Imhoff, Andreas B | Knee surgery, sports traumatology, arthroscopy : official journal of the ESSKA | 2013 | 10.1007/s00167-012-2022-6 | English |
| 30797677 | Hermes patellofemoral arthroplasty: Annual revision rate and clinical results after two to 20 years of follow-up. | Bohu, Yoann; Klouche, Shahnaz; Sezer, Hasan Basri; Gerometta, Antoine; Lefevre, Nicolas; Herman, Serge | The Knee | 2019 | 10.1016/j.knee.2019.01.014 | English |
| 27522543 | Midterm Outcome of Avon Patellofemoral Arthroplasty for Posttraumatic Unicompartmental Osteoarthritis. | Konan, Sujith; Haddad, Fares S | The Journal of arthroplasty | 2016 | 10.1016/j.arth.2016.06.005 | English |
| 26094823 | Obesity and the absence of trochlear dysplasia increase the risk of revision in patellofemoral arthroplasty. | Liow, Ming Han Lincoln; Goh, Graham Seow-Hng; Tay, Darren Keng-Jin; Chia, Shi-Lu; Lo, Ngai-Nung; Yeo, Seng-Jin | The Knee | 2016 | 10.1016/j.knee.2015.05.009 | English |
| 16818972 | Custom patellofemoral arthroplasty of the knee. | Sisto, Domenick J; Sarin, Vineet K | The Journal of bone and joint surgery. American volume | 2006 | 10.2106/JBJS.E.00382 | English |
| 3180587 | Patellofemoral arthroplasty. A three- to nine-year follow-up study. | Arciero, R A; Toomey, H E | Clinical orthopaedics and related research | 1988 |  | English |
| 26430081 | Long-term results of compartmental arthroplasties of the knee: Long term results of partial knee arthroplasty. | Parratte, S; Ollivier, M; Lunebourg, A; Abdel, M P; Argenson, J-N | The bone & joint journal | 2015 | 10.1302/0301-620X.97B10.36426 | English |
| 16146626 | Is anterior knee pain a predisposing factor to patellofemoral osteoarthritis? | Utting, M R; Davies, G; Newman, J H | The Knee | 2005 | 10.1016/j.knee.2004.12.006 | English |
| 535258 | Patellofemoral replacement. | Blazina, M E; Fox, J M; Del Pizzo, W; Broukhim, B; Ivey, F M | Clinical orthopaedics and related research | 1979 |  | English |
| 31754729 | Smoking, unemployment, female sex, obesity, and medication use yield worse outcomes in patellofemoral arthroplasty. | Desai, Vishal S; Pareek, Ayoosh; DeDeugd, Casey M; Sabbag, Orlando D; Krych, Aaron J; Cummings, Nancy M; Dahm, Diane L | Knee surgery, sports traumatology, arthroscopy : official journal of the ESSKA | 2020 | 10.1007/s00167-019-05704-y | English |
| 33749150 | Joint Awareness after Patellofemoral Arthroplasty Evaluated with the Forgotten Joint Score: A Comparison Study. | Lin, Wei; Dai, Yike; Dong, Conglei; Piao, Kang; Hao, Kuo; Wang, Fei | Orthopaedic surgery | 2021 | 10.1111/os.12921 | English |
| 35348817 | Patellofemoral arthroplasty: obesity linked to high risk of revision and progression of medial tibiofemoral osteoarthritis. | Marullo, Matteo; Bargagliotti, Marco; Vigano', Marco; Lacagnina, Claudio; Romagnoli, Sergio | Knee surgery, sports traumatology, arthroscopy : official journal of the ESSKA | 2022 | 10.1007/s00167-022-06947-y | English |
| 15024580 | The Lubinus patellofemoral arthroplasty: a series of 17 cases. | Board, T N; Mahmood, A; Ryan, W G; Banks, A J | Archives of orthopaedic and trauma surgery | 2004 | 10.1007/s00402-004-0645-x | English |
| 18811029 | Medium-term results of patellofemoral joint arthroplasty. | Mohammed, Riazuddin; Jimulia, Tushar; Durve, Kaustubh; Bansal, Manish; Green, Marcus; Learmonth, Duncan | Acta orthopaedica Belgica | 2008 |  | English |
| 15995414 | Development and early results of a new patellofemoral arthroplasty. | Ackroyd, Christopher E; Chir, B | Clinical orthopaedics and related research | 2005 | 10.1097/01.blo.0000171914.94503.d1 | English |
| 22927894 | Patellofemoral arthroplasty: a systematic review of the literature. | Tarassoli, Payam; Punwar, Shahid; Khan, Wasim; Johnstone, David | The open orthopaedics journal | 2012 | 10.2174/1874325001206010340 | English |
| 31259645 | Failure modes of patellofemoral arthroplasty-registries vs. clinical studies: a systematic review. | Bendixen, Nikolaj B; Eskelund, Peter W; Odgaard, Anders | Acta orthopaedica | 2019 | 10.1080/17453674.2019.1634865 | English |
| 21290009 | Patellofemoral arthroplasty versus total knee arthroplasty in patients with isolated patellofemoral osteoarthritis. | Dahm, Diane L; Al-Rayashi, Walid; Dajani, Khaled; Shah, Jay P; Levy, Bruce A; Stuart, Michael J | American journal of orthopedics (Belle Mead, N.J.) | 2010 |  | English |
| 23594895 | [Dislocation of the polyethylene in a patello-femoral replacement with a movable patella].Luxacion del polietileno en protesis patelo-femoral con patela movil. Caso clinico. | Sanguesa Nebot, M J; Cabanes Soriano, F; Fernandez Gabarda, R; Darder Prats, A | Revista espanola de cirugia ortopedica y traumatologia | 2012 | 10.1016/j.recot.2012.05.008 | Spanish |
| 24696005 | Evidence of trochlear dysplasia in patellofemoral arthroplasty designs. | Saffarini, Mo; Ntagiopoulos, Panagiotis G; Demey, Guillaume; Le Negaret, Benoit; Dejour, David H | Knee surgery, sports traumatology, arthroscopy : official journal of the ESSKA | 2014 | 10.1007/s00167-014-2967-8 | English |
| 29992071 | The Benefits of an In-Office Arthroscopy in the Diagnosis of Unresolved Knee Pain. | Chapman, Garrett L; Amin, Nirav H | Case reports in orthopedics | 2018 | 10.1155/2018/6125676 | English |
| 26984650 | Patella component loosening--A case report. | Bloemheuvel, E M; van Rooij, W M J; van den Besselaar, M | Acta orthopaedica Belgica | 2016 |  | English |
| 30264243 | Similar postoperative patient-reported outcome in both second generation patellofemoral arthroplasty and total knee arthroplasty for treatment of isolated patellofemoral osteoarthritis: a systematic review. | Bunyoz, Kristine Ifigenia; Lustig, Sebastien; Troelsen, Anders | Knee surgery, sports traumatology, arthroscopy : official journal of the ESSKA | 2019 | 10.1007/s00167-018-5151-8 | English |
| 34602157 | Editorial Commentary: Is the Pendulum Swinging Away From Patellar Fixation in Medial Patellofemoral Ligament Reconstruction? | Sherman, Seth L; Curtis, Daniel M | Arthroscopy : the journal of arthroscopic & related surgery : official publication of the Arthroscopy Association of North America and the International Arthroscopy Association | 2021 | 10.1016/j.arthro.2021.06.001 | English |
| 27825938 | Why do patellofemoral arthroplasties fail today? A systematic review. | van der List, J P; Chawla, H; Villa, J C; Pearle, A D | The Knee | 2017 | 10.1016/j.knee.2015.11.002 | English |
| 8926124 | Unicompartmental knee arthroplasty with patelloplasty. | Antoniou, J; Hadjipavlou, A; Enker, P; Antoniou, A | International orthopaedics | 1996 | 10.1007/s002640050038 | English |
| 18417346 | Pre-operative mental wellbeing and the outcome of knee replacement. | Walton, Michael J; Newman, John H | The Knee | 2008 | 10.1016/j.knee.2008.03.001 | English |
| 17518383 | MBARS: mini bone-attached robotic system for joint arthroplasty. | Wolf, A; Jaramaz, B; Lisien, B; DiGioia, A M | The international journal of medical robotics + computer assisted surgery : MRCAS | 2005 | 10.1581/mrcas.2005.010210 | English |
| 20044679 | The Avon patellofemoral joint replacement: Five-year results from an independent centre. | Odumenya, M; Costa, M L; Parsons, N; Achten, J; Dhillon, M; Krikler, S J | The Journal of bone and joint surgery. British volume | 2010 | 10.1302/0301-620X.92B1.23135 | English |
| 31362558 | Classification of combined partial knee arthroplasty. | Garner, A; van Arkel, R J; Cobb, J | The bone & joint journal | 2019 | 10.1302/0301-620X.101B8.BJJ-2019-0125.R1 | English |
| 35282735 | Patellar button compatibility in the conversion of Patellofemoral Arthroplasty to a Total Knee Arthroplasty: A review of the contemporary literature. | McDonald, Laura K; Kurmis, Andrew P | Journal of orthopaedic surgery (Hong Kong) | 2022 | 10.1177/10225536221084147 | English |
| 33858458 | Patellofemoral arthroplasty versus total knee arthroplasty for isolated patellofemoral osteoarthritis: a systematic review and meta-analysis. | Peng, Guanrong; Liu, Min; Guan, Zhenhua; Hou, Yunfei; Liu, Qiang; Sun, Xiaobo; Zhu, Xingyang; Feng, Wenjun; Zeng, Jianchun; Zhong, Zhangrong; Zeng, Yirong | Journal of orthopaedic surgery and research | 2021 | 10.1186/s13018-021-02414-5 | English |
| 35004156 | Arthroscopic Debridement, Facetectomy, andSynovectomy for Isolated Patellofemoral Osteoarthritis. | Zhao, Jinzhong | Arthroscopy techniques | 2021 | 10.1016/j.eats.2021.08.021 | English |
| 21703794 | MRI after patellofemoral replacement: the preserved compartments. | Heyse, Thomas J; Figiel, Jens; Hahnlein, Ulrike; Timmesfeld, Nina; Lakemeier, Stefan; Schofer, Markus D; Fuchs-Winkelmann, Susanne; Efe, Turgay | European journal of radiology | 2012 | 10.1016/j.ejrad.2011.06.012 | English |
| 22878660 | Complications in patellofemoral surgery. | Tompkins, Marc; Arendt, Elizabeth A | Sports medicine and arthroscopy review | 2012 | 10.1097/JSA.0b013e31825c74cf | English |
| 22928430 | The basic science of the patella: structure, composition, and function. | Fox, Alice J S; Wanivenhaus, Florian; Rodeo, Scott A | The journal of knee surgery | 2012 | 10.1055/s-0032-1313741 | English |
| 30635231 | Surgical technique in patellofemoral arthroplasty. | Remy, Franck | Orthopaedics & traumatology, surgery & research : OTSR | 2019 | 10.1016/j.otsr.2018.05.020 | English |
| 27916580 | Annual revision rates of partial versus total knee arthroplasty: A comparative meta-analysis. | Chawla, Harshvardhan; van der List, Jelle P; Christ, Alexander B; Sobrero, Maximiliano R; Zuiderbaan, Hendrik A; Pearle, Andrew D | The Knee | 2017 | 10.1016/j.knee.2016.11.006 | English |
| 31320188 | The Combination of Inlay Patellofemoral Arthroplasty and Medial Unicompartmental Knee Arthroplasty Versus Total Knee Arthroplasty for Mediopatellofemoral Osteoarthritis: A Comparison of Mid-Term Outcomes. | Uluyardimci, Enes; Isik, Cetin; Tahta, Mesut; Emre, Fahri; Cepni, Sahin; Oltulu, Ismail | The Journal of arthroplasty | 2019 | 10.1016/j.arth.2019.06.043 | English |
| 19223947 | Patellofemoral resurfacing arthroplasty: literature review and description of a novel technique. | Cannon, Anthony; Stolley, Mary; Wolf, Brian; Amendola, Annunziato | The Iowa orthopaedic journal | 2008 |  | English |
| 25079134 | Patellofemoral arthroplasty: outcomes and factors associated with early progression of tibiofemoral arthritis. | Dahm, Diane L; Kalisvaart, Michael M; Stuart, Michael J; Slettedahl, Seth W | Knee surgery, sports traumatology, arthroscopy : official journal of the ESSKA | 2014 | 10.1007/s00167-014-3202-3 | English |
| 23188904 | The correct rotation of the femoral component in patellofemoral replacement: a laboratory assessment of a surgical technique. | Clark, D A; Upadhyay, N; Gillespie, G; Wakeley, C; Eldridge, J D | The Journal of bone and joint surgery. British volume | 2012 | 10.1302/0301-620X.94B12.29506 | English |
| 32869235 | Patellofemoral Arthroplasty Improves Patellofemoral Alignment in Patients with Patellofemoral Osteoarthritis with Trochlear Dysplasia. | Yang, Guangmin; Wang, Jue; Dai, Yike; Lin, Wei; Niu, Jinghui; Wang, Fei | The journal of knee surgery | 2022 | 10.1055/s-0040-1715099 | English |
| 9586725 | Patellofemoral arthrosis: the treatment options. | Oberlander, M A; Baker, C L; Morgan, B E | American journal of orthopedics (Belle Mead, N.J.) | 1998 |  | English |
| 18407278 | In vitro simulation and quantification of wear within the patellofemoral joint replacement. | Ellison, P; Barton, D C; Esler, C; Shaw, D L; Stone, M H; Fisher, J | Journal of biomechanics | 2008 | 10.1016/j.jbiomech.2008.02.029 | English |
| 3409568 | Blood loss with total knee arthroplasty. | Berman, A T; Geissele, A E; Bosacco, S J | Clinical orthopaedics and related research | 1988 |  | English |
| 16330515 | Anterior knee pain: diagnosis and treatment. | Post, William R | The Journal of the American Academy of Orthopaedic Surgeons | 2005 | 10.5435/00124635-200512000-00006 | English |
| 31121631 | Patellofemoral Arthroplasty: Short-Term Complications and Risk Factors. | Rezzadeh, Kevin; Behery, Omar A; Kester, Benjamin S; Dogra, Tara; Vigdorchik, Jonathon; Schwarzkopf, Ran | The journal of knee surgery | 2020 | 10.1055/s-0039-1688960 | English |
| 32114806 | The PAT randomized clinical trial. | Joseph, Michelle N; Achten, Juul; Parsons, Nick R; Costa, Matthew L | The bone & joint journal | 2020 | 10.1302/0301-620X.102B3.BJJ-2019-0723.R1 | English |
| 22992881 | Patellofemoral arthroplasty: the other unicompartmental knee replacement. | Walker, Torrance; Perkinson, Brian; Mihalko, William M | The Journal of bone and joint surgery. American volume | 2012 | 10.2106/JBJS.L.00539 | English |
| 19347881 | HyBAR: hybrid bone-attached robot for joint arthroplasty. | Song, S; Mor, A; Jaramaz, B | The international journal of medical robotics + computer assisted surgery : MRCAS | 2009 | 10.1002/rcs.254 | English |
| 33125548 | Preoperative patellofemoral anatomy affects failure rate after isolated patellofemoral inlay arthroplasty. | Feucht, Matthias J; Lutz, Patricia M; Ketzer, Conrad; Rupp, Marco C; Cotic, Matthias; Imhoff, Andreas B; Pogorzelski, Jonas | Archives of orthopaedic and trauma surgery | 2020 | 10.1007/s00402-020-03651-9 | English |
| 33438917 | Cartilage Disease of the Patellofemoral Joint: Realignment, Restoration, Replacement. | Otlans, Peters; Lattermann, Christian; Sherman, Seth L; Gomoll, Andreas H; Lonner, Jess H; Freedman, Kevin B | Instructional course lectures | 2021 |  | English |
| 32739080 | No Major Functional Benefit After Bicompartmental Knee Arthroplasty Compared to Total Knee Arthroplasty at 5-Year Follow-Up. | Schrednitzki, Daniel; Beier, Alexander; Marx, Axel; Halder, Andreas M | The Journal of arthroplasty | 2020 | 10.1016/j.arth.2020.07.003 | English |
| 30103596 | [Progress on treatment for patellofemoral arthritis]. | Zhang, Zhong-Xing; Xu, Feng; Yang, Xiao-Long | Zhongguo gu shang = China journal of orthopaedics and traumatology | 2018 | 10.3969/j.issn.1003-0034.2018.07.019 | Chinese |
| 26217462 | The Avon Patellofemoral Joint Replacement: Mid-Term Prospective Results from an Independent Centre. | Akhbari, Pouya; Malak, Tamer; Dawson-Bowling, Sebastian; East, Debra; Miles, Kim; Butler-Manuel, P Adrian | Clinics in orthopedic surgery | 2015 | 10.4055/cios.2015.7.2.171 | English |
| 32228074 | Patellofemoral arthroplasty is cheaper and more effective in the short term than total knee arthroplasty for isolated patellofemoral osteoarthritis: cost-effectiveness analysis based on a randomized trial. | Fredborg, Charlotte; Odgaard, Anders; Sorensen, Jan | The bone & joint journal | 2020 | 10.1302/0301-620X.102B4.BJJ-2018-1580.R3 | English |
| 31888356 | Mid-term survivorship and patient-reported outcomes of robotic-arm assisted partial knee arthroplasty. | Burger, Joost A; Kleeblad, Laura J; Laas, Niels; Pearle, Andrew D | The bone & joint journal | 2020 | 10.1302/0301-620X.102B1.BJJ-2019-0510.R1 | English |
| 28768779 | Cost effectiveness of patellofemoral versus total knee arthroplasty in younger patients. | Chawla, H; Nwachukwu, B U; van der List, J P; Eggman, A A; Pearle, A D; Ghomrawi, H M | The bone & joint journal | 2017 | 10.1302/0301-620X.99B8.BJJ-2016-1032.R1 | English |
| 31579533 | Patellofemoral Arthroplasty. | Odgaard, Anders; Eldridge, Jonathan; Madsen, Frank | JBJS essential surgical techniques | 2019 | 10.2106/JBJS.ST.18.00094 | English |
| 27330013 | Knee osteoarthritis: a review of management options. | Hussain, S M; Neilly, D W; Baliga, S; Patil, S; Meek, Rmd | Scottish medical journal | 2016 | 10.1177/0036933015619588 | English |
| 19340381 | Modular bicompartmental knee arthroplasty with robotic arm assistance. | Lonner, Jess H | American journal of orthopedics (Belle Mead, N.J.) | 2009 |  | English |
| 15995424 | Alternatives to patellofemoral arthroplasty. | Fulkerson, John P | Clinical orthopaedics and related research | 2005 |  | English |
| 30168762 | The Avon patellofemoral joint arthroplasty: two- to 18-year results of a large single-centre cohort. | Metcalfe, A J; Ahearn, N; Hassaballa, M A; Parsons, N; Ackroyd, C E; Murray, J R; Robinson, J R; Eldridge, J D; Porteous, A J | The bone & joint journal | 2018 | 10.1302/0301-620X.100B9.BJJ-2018-0174.R1 | English |
| 15995419 | Long-term results with the first patellofemoral prosthesis. | Cartier, Philippe; Sanouiller, Jean-Louis; Khefacha, Ahmed | Clinical orthopaedics and related research | 2005 |  | English |
| 24395311 | Revision cartilage cell transplantation for failed autologous chondrocyte transplantation in chronic osteochondral defects of the knee. | Vijayan, S; Bentley, G; Rahman, J; Briggs, T W R; Skinner, J A; Carrington, R W J | The bone & joint journal | 2014 | 10.1302/0301-620X.96B1.31979 | English |
| 18399598 | Controversies and techniques in the surgical management of patellofemoral arthritis. | Mihalko, William M; Boachie-Adjei, Yaw; Spang, Jeffrey T; Fulkerson, John P; Arendt, Elizabeth A; Saleh, Khaled J | Instructional course lectures | 2008 |  | English |
| 32867858 | Unicompartmental vs. segmental bicompartmental vs. total knee replacement: comparison of clinical outcomes. | Al-Dadah, Oday; Hawes, Georgina; Chapman-Sheath, Philip J; Tice, John William; Barrett, David S | Knee surgery & related research | 2020 | 10.1186/s43019-020-00065-0 | English |
| 3665248 | Pathomechanics of the femoropatellar joint following total knee arthroplasty. | Hofmann, G O; Hagena, F W | Clinical orthopaedics and related research | 1987 |  | English |
| 7867096 | [Long-term follow-up of patients with patella resurfacing]. | Shen, J; Ye, Q; Li, S | Zhongguo yi xue ke xue yuan xue bao. Acta Academiae Medicinae Sinicae | 1994 |  | Chinese |
| 30291399 | A high level of satisfaction after bicompartmental individualized knee arthroplasty with patient-specific implants and instruments. | Ogura, Takahiro; Le, Kiet; Merkely, Gergo; Bryant, Tim; Minas, Tom | Knee surgery, sports traumatology, arthroscopy : official journal of the ESSKA | 2019 | 10.1007/s00167-018-5155-4 | English |
| 11476308 | The Lubinus patellofemoral arthroplasty. A five- to ten-year prospective study. | Tauro, B; Ackroyd, C E; Newman, J H; Shah, N A | The Journal of bone and joint surgery. British volume | 2001 | 10.1302/0301-620X.83B5.11577 | English |
| 35191830 | Combined procedures with unicompartmental knee arthroplasty: High risk of stiffness but promising concept in selected indications. | Derreveaux, Vianney; Schmidt, Axel; Shatrov, Jobe; Sappey-Marinier, Elliot; Batailler, Cecile; Servien, Elvire; Lustig, Sebastien | SICOT-J | 2022 | 10.1051/sicotj/2022002 | English |
| 32348089 | Short-term Revision Risk of Patellofemoral Arthroplasty Is High: An Analysis from Eight Large Arthroplasty Registries. | Lewis, Peter L; Tudor, Francois; Lorimer, Michelle; McKie, John; Bohm, Eric; Robertsson, Otto; Makela, Keijo T; Haapakoski, Jaason; Furnes, Ove; Bartz-Johannessen, Christoffer; Nelissen, Rob G H H; Van Steenbergen, Liza N; Fithian, Donald C; Prentice, Heather A | Clinical orthopaedics and related research | 2020 | 10.1097/CORR.0000000000001268 | English |
| 27714438 | High mid-term revision rate after treatment of large, full-thickness cartilage lesions and OA in the patellofemoral joint using a large inlay resurfacing prosthesis: HemiCAP-Wave. | Laursen, Jens Ole | Knee surgery, sports traumatology, arthroscopy : official journal of the ESSKA | 2017 | 10.1007/s00167-016-4352-2 | English |
| 11830378 | Treatment of patello-femoral arthritis using the Lubinus patello-femoral arthroplasty: a retrospective review. | Smith, A M; Peckett, W R C; Butler-Manuel, P A; Venu, K M; d'Arcy, J C | The Knee | 2002 | 10.1016/S0968-0160(01)00127-2 | English |
| 29614710 | Patient-reported outcome measures (PROMs) in patients undergoing patellofemoral arthroplasty and total knee replacement: A comparative study. | Perrone, Fabio Luigi; Baron, Steffen; Suero, Eduardo M; Lausmann, Christian; Kendoff, Daniel; Zahar, Akos; Gehrke, Thorsten; Citak, Mustafa | Technology and health care : official journal of the European Society for Engineering and Medicine | 2018 | 10.3233/THC-181185 | English |
| 26955615 | Are Patellofemoral Symptoms Truly Related to the Patellofemoral Joint? | Yassa, Rafik; Khalfaoui, Mahdi Yacine; Davies, Andrew P | Knee surgery & related research | 2016 | 10.5792/ksrr.2016.28.1.68 | English |
| 29270562 | Patellofemoral Arthroplasty: Current Concepts and Review of the Literature. | Pisanu, Gabriele; Rosso, Federica; Bertolo, Corrado; Dettoni, Federico; Blonna, Davide; Bonasia, Davide Edoardo; Rossi, Roberto | Joints | 2017 | 10.1055/s-0037-1606618 | English |
| 27622149 | Femoro Patella Vialla patellofemoral arthroplasty: An independent assessment of outcomes at minimum 2-year follow-up. | Halai, Mansur; Ker, Andrew; Anthony, Iain; Holt, Graeme; Jones, Bryn; Blyth, Mark | World journal of orthopedics | 2016 | 10.5312/wjo.v7.i8.487 | English |
| 3967411 | Prospective analysis of Sheehan total knee arthroplasty. | Callihan, S M; Halley, D K | Clinical orthopaedics and related research | 1985 |  | English |
| 21596570 | Patellofemoral osteoarthritis treated by partial lateral facetectomy: results at long-term follow up. | Wetzels, T; Bellemans, J | The Knee | 2012 | 10.1016/j.knee.2011.04.005 | English |
| 10546602 | Total knee replacement for patients with patellofemoral arthritis. | Laskin, R S; van Steijn, M | Clinical orthopaedics and related research | 1999 |  | English |
| 27052369 | Patella tracking and patella contact pressure in modular patellofemoral arthroplasty: a biomechanical in vitro analysis. | Calliess, Tilman; Ettinger, Max; Schado, Ssuheib; Becher, Christoph; Hurschler, Christof; Ostermeier, Sven | Archives of orthopaedic and trauma surgery | 2016 | 10.1007/s00402-016-2451-7 | English |
| 28713612 | In vivo magnetic resonance imaging morphometry of the patella bone in South Indian population. | Muhamed, Reshma; Saralaya, Vasudha V; Murlimanju, B V; Chettiar, Ganesh Kumar | Anatomy & cell biology | 2017 | 10.5115/acb.2017.50.2.99 | English |
| 2229117 | The patellofemoral joint after total knee arthroplasty without patellar resurfacing. | Picetti, G D 3rd; McGann, W A; Welch, R B | The Journal of bone and joint surgery. American volume | 1990 | 10.2106/00004623-199072090-00017 | English |
| 19491912 | Surgical options for patients with osteoarthritis of the knee. | Lutzner, Jorg; Kasten, Philip; Gunther, Klaus-Peter; Kirschner, Stephan | Nature reviews. Rheumatology | 2009 | 10.1038/nrrheum.2009.88 | English |
| 33114034 | Medical Interventions for Patellofemoral Pain and Patellofemoral Osteoarthritis: A Systematic Review. | Macri, Erin M; Hart, Harvi F; Thwaites, David; Barton, Christian J; Crossley, Kay M; Bierma-Zeinstra, Sita M A; van Middelkoop, Marienke | Journal of clinical medicine | 2020 | 10.3390/jcm9113397 | English |
| 23255644 | Mobile-bearing, congruent patellofemoral prosthesis: short-term results. | Yadav, Basavaraj; Shaw, David; Radcliffe, Graham; Dachepalli, Sunil; Kluge, Wolfram | Journal of orthopaedic surgery (Hong Kong) | 2012 | 10.1177/230949901202000317 | English |
| 17592686 | [Treatment of patellofemoral arthritis with patello-femoral arthroplasties].Behandling af patellofemoral artrose med en patellofemoral alloplastik. | Jorgensen, Per Seest; Konradsen, Lars Aage Glud; Mati, Wael B; Torholm, Carsten | Ugeskrift for laeger | 2007 |  | Danish |
| 26156155 | Patellectomy for osteoarthritis: a new tension preserving surgical technique to reconstruct the extensor mechanism with retrospective review of long-term follow-up. | Asopa, Vipin; Willis-Owen, Charles; Keene, Greg | Journal of orthopaedic surgery and research | 2015 | 10.1186/s13018-015-0237-1 | English |
| 24417902 | Association and impact of patellofemoral dysplasia on patellofemoral arthropathy and arthroplasty. | Mofidi, Ali; Veravalli, Karunakar; Jinnah, Riaz H; Poehling, Gary G | The Knee | 2014 | 10.1016/j.knee.2013.09.009 | English |
| 36215923 | Knee arthroplasty utilization trends from 2010 to 2019. | Fuller, Samuel I; Cohen, Jordan S; Malyavko, Alisa; Agarwal, Amil R; Stake, Seth; Golladay, Gregory J; Thakkar, Savyasachi C | The Knee | 2022 | 10.1016/j.knee.2022.09.006 | English |
| 28522245 | Pin Site Complications Associated With Computer-Assisted Navigation in Hip and Knee Arthroplasty. | Kamara, Eli; Berliner, Zachary P; Hepinstall, Matthew S; Cooper, H John | The Journal of arthroplasty | 2017 | 10.1016/j.arth.2017.03.073 | English |
| 1420832 | [Morphological and numerical studies of the stress compatibility of patella implants].Morphologische und numerische Untersuchungen zur Stresskompatibilitat von Patellaimplantaten. | Lengsfeld, M; Weiss, H; Kienapfel, H | Biomedizinische Technik. Biomedical engineering | 1992 | 10.1515/bmte.1992.37.10.222 | German |
| 32845359 | Reliable improvements in participation in low-impact sports following implantation of a patellofemoral inlay arthroplasty at mid-term follow-up. | Pogorzelski, Jonas; Rupp, Marco-Christopher; Ketzer, Conrad; Cotic, Matthias; Lutz, Patricia; Beeck, Saskia; Imhoff, Andreas B; Feucht, Matthias J | Knee surgery, sports traumatology, arthroscopy : official journal of the ESSKA | 2021 | 10.1007/s00167-020-06245-5 | English |
| 34241595 | Does bicompartmental knee arthroplasty hold an advantage over total knee arthroplasty? Systematic review and meta-analysis. | Elbardesy, Hany; Awad, Ahmed K; McLeod, Andre; Farahat, Samar Tarek; Sayed, Somaya Zain Elabdeen; Guerin, Shane; Harty, James | SICOT-J | 2021 | 10.1051/sicotj/2021036 | English |
| 20175647 | Isolated patellofemoral osteoarthritis. | van Jonbergen, Hans-Peter W; Poolman, Rudolf W; van Kampen, Albert | Acta orthopaedica | 2010 | 10.3109/17453671003628756 | English |
| 28138717 | [Patellofemoral arthroplasty].Der femoropatellare Teilersatz. | Cotic, M; Forkel, P; Imhoff, A B | Operative Orthopadie und Traumatologie | 2017 | 10.1007/s00064-016-0477-1 | German |
| 21987361 | Complications after patello-femoral versus total knee replacement in the treatment of isolated patello-femoral osteoarthritis. A meta-analysis. | Dy, C J; Franco, N; Ma, Y; Mazumdar, M; McCarthy, M M; Gonzalez Della Valle, A | Knee surgery, sports traumatology, arthroscopy : official journal of the ESSKA | 2012 | 10.1007/s00167-011-1677-8 | English |
| 24482626 | Surgical treatment of isolated patellofemoral osteoarthritis. | Rodriguez-Merchan, E Carlos | HSS journal : the musculoskeletal journal of Hospital for Special Surgery | 2014 | 10.1007/s11420-013-9375-3 | English |
| 33051119 | Use of Natural Language Processing Algorithms to Identify Common Data Elements in Operative Notes for Knee Arthroplasty. | Sagheb, Elham; Ramazanian, Taghi; Tafti, Ahmad P; Fu, Sunyang; Kremers, Walter K; Berry, Daniel J; Lewallen, David G; Sohn, Sunghwan; Maradit Kremers, Hilal | The Journal of arthroplasty | 2021 | 10.1016/j.arth.2020.09.029 | English |
| 29893799 | Estimating an Individual's Probability of Revision Surgery After Knee Replacement: A Comparison of Modeling Approaches Using a National Data Set. | Aram, Parham; Trela-Larsen, Lea; Sayers, Adrian; Hills, Andrew F; Blom, Ashley W; McCloskey, Eugene V; Kadirkamanathan, Visakan; Wilkinson, Jeremy M | American journal of epidemiology | 2018 | 10.1093/aje/kwy121 | English |
| 26231494 | A 21% conversion rate to total knee arthroplasty of a first-generation patellofemoral prosthesis at a mean follow-up of 9.7 years. | Hoogervorst, Paul; de Jong, Richard J; Hannink, Gerjon; van Kampen, Albert | International orthopaedics | 2015 | 10.1007/s00264-015-2941-1 | English |
| 31515593 | The patient results and satisfaction of knee arthroplasty in a validated grading system. | Oosthuizen, Christiaan Rudolf; Van Der Straeten, Catherine; Maposa, Innocent; Snyckers, Christian Hugo; Vermaak, Duwayne Peter; Magobotha, Sebastian | International orthopaedics | 2019 | 10.1007/s00264-019-04412-z | English |
| 31427243 | Early post-operative opioid consumption: A comparison between medial unicompartmental, patellofemoral, and total knee arthroplasty. | Melnic, Christopher M; Kazarian, Erick R; Dwyer, Maureen K; Domingo-Johnson, E L; Freiberg, Andrew A; Bedair, Hany S | The Knee | 2019 | 10.1016/j.knee.2019.06.016 | English |
| 29021089 | Patellofemoral arthritis treated with resurfacing implant: Clinical outcome and complications at a minimum two-year follow-up. | Zicaro, Juan Pablo; Yacuzzi, Carlos; Astoul Bonorino, Juan; Carbo, Lisandro; Costa-Paz, Matias | The Knee | 2017 | 10.1016/j.knee.2017.09.003 | English |
| 28661229 | The influence of malalignment and ageing following sterilisation by gamma irradiation in an inert atmosphere on the wear of ultra-high-molecular-weight polyethylene in patellofemoral replacements. | Maiti, Raman; Cowie, Raelene M; Fisher, John; Jennings, Louise M | Proceedings of the Institution of Mechanical Engineers. Part H, Journal of engineering in medicine | 2017 | 10.1177/0954411917696112 | English |
| 32037873 | Prospective Outcomes of Cryopreserved Osteochondral Allograft for Patellofemoral Cartilage Defects at Minimum 2-Year Follow-up. | Melugin, Heath P; Ridley, Taylor J; Bernard, Christopher D; Wischmeier, Dillen; Farr, Jack; Stuart, Michael J; Macalena, Jeffrey A; Krych, Aaron J | Cartilage | 2021 | 10.1177/1947603520903420 | English |
| 34146717 | Augmented reality in robotic assisted orthopaedic surgery: A pilot study. | Iqbal, Hisham; Tatti, Fabio; Rodriguez Y Baena, Ferdinando | Journal of biomedical informatics | 2021 | 10.1016/j.jbi.2021.103841 | English |
| 17261986 | Functional outcomes after different types of knee arthroplasty: kneeling ability versus descending stairs. | Hassaballa, Mo A; Porteous, Andrew J; Learmonth, Ian D | Medical science monitor : international medical journal of experimental and clinical research | 2007 |  | English |
| 19888591 | UKA in combination with PFR at average 12-year follow-up. | Heyse, Thomas Jan; Khefacha, Ahmed; Cartier, Philippe | Archives of orthopaedic and trauma surgery | 2010 | 10.1007/s00402-009-0997-3 | English |
| 23723274 | Early revisions of the Femoro-Patella Vialla joint replacement. | Williams, D P; Pandit, H G; Athanasou, N A; Murray, D W; Gibbons, C L M H | The bone & joint journal | 2013 | 10.1302/0301-620X.95B6.31355 | English |
| 33571948 | Recurrent patellar dislocations with patellar cartilage defects: A pain in the knee? | Dalal, Shaival; Setia, Piyush; Debnath, Anindya; Guro, Randy; Kotwal, Rahul; Chandratreya, Amit | The Knee | 2021 | 10.1016/j.knee.2021.01.019 | English |
| 31699493 | Replacement for patellofemoral arthritis. | Johnson, David Sands; Turner, Philip Gartside | The Knee | 2019 | 10.1016/j.knee.2019.10.016 | English |
| 12905899 | [Preliminary investigation on the pathogeny, diagnosis and treatment of chondromalacia patella]. | Ye, Q B; Wu, Z H; Wang, Y P; Lin, J; Qiu, G X | Zhongguo yi xue ke xue yuan xue bao. Acta Academiae Medicinae Sinicae | 2001 |  | Chinese |
| 36527357 | Short term clinical outcomes of a Prochondrix thin laser-etched osteochondral allograft for the treatment of articular cartilage defects in the knee. | Mehta, Vishal M; Mehta, Shaan; Santoro, Steven; Shriver, Ryan; Mandala, Cassie; Weess, Cameron | Journal of orthopaedic surgery (Hong Kong) | 2022 | 10.1177/10225536221141781 | English |
| 33088840 | Comparing Return to Sports After Patellofemoral and Knee Arthroplasty in an Age- and Sex-Matched Cohort. | Schneider, Brandon L; Ling, Daphne I; Kleebad, Laura J; Strickland, Sabrina; Pearle, Andrew | Orthopaedic journal of sports medicine | 2020 | 10.1177/2325967120957425 | English |
| 29654550 | Bicompartmental (uni plus patellofemoral) versus total knee arthroplasty: a match-paired study. | Biazzo, A; Silvestrini, F; Manzotti, A; Confalonieri, N | Musculoskeletal surgery | 2019 | 10.1007/s12306-018-0540-1 | English |
| 23711753 | Advanced patellofemoral cartilage lesions in patients younger than 50 years of age: is there an ideal operative option? | Noyes, Frank R; Barber-Westin, Sue D | Arthroscopy : the journal of arthroscopic & related surgery : official publication of the Arthroscopy Association of North America and the International Arthroscopy Association | 2013 | 10.1016/j.arthro.2013.03.077 | English |
| 23715952 | Results of a French multicentre retrospective experience with four hundred and eighteen failed unicondylar knee arthroplasties. | Saragaglia, Dominique; Bonnin, Michel; Dejour, David; Deschamps, Gerard; Chol, Christophe; Chabert, Benoit; Refaie, Ramsay | International orthopaedics | 2013 | 10.1007/s00264-013-1915-4 | English |
| 36479459 | Effectiveness of an Accelerated Rehabilitation Protocol After Tibial Tubercle Osteotomy. | Morgan, Courtney; Bell, Ryan M; Burland, Julie P; Kriscenski, Danielle; Ilinski, Adrian; Cote, Mark P; Edgar, Cory M | Orthopaedic journal of sports medicine | 2022 | 10.1177/23259671221133105 | English |
| 32529388 | Clinical outcome of bi-unicompartmental knee arthroplasty for both medial and lateral femorotibial arthritis: a systematic review-is there proof of concept? | Wada, Keizo; Price, Andrew; Gromov, Kirill; Lustig, Sebastien; Troelsen, Anders | Archives of orthopaedic and trauma surgery | 2020 | 10.1007/s00402-020-03492-6 | English |
| 30276437 | High patient satisfaction with significant improvement in knee function and pain relief after mid-term follow-up in patients with isolated patellofemoral inlay arthroplasty. | Imhoff, Andreas B; Feucht, Matthias J; Bartsch, Eva; Cotic, Matthias; Pogorzelski, Jonas | Knee surgery, sports traumatology, arthroscopy : official journal of the ESSKA | 2019 | 10.1007/s00167-018-5173-2 | English |
| 29529622 | The Mark Coventry Award: Patellofemoral Arthroplasty Results in Better Range of Movement and Early Patient-reported Outcomes Than TKA. | Odgaard, Anders; Madsen, Frank; Kristensen, Per Wagner; Kappel, Andreas; Fabrin, Jesper | Clinical orthopaedics and related research | 2018 | 10.1007/s11999.0000000000000017 | English |
| 28211301 | Early results of patellofemoral inlay resurfacing arthroplasty using the HemiCap Wave prosthesis. | Patel, Akash; Haider, Zakir; Anand, Amarjit; Spicer, Dominic | Journal of orthopaedic surgery (Hong Kong) | 2017 | 10.1177/2309499017692705 | English |
| 26047754 | Is knee function better with contemporary modular bicompartmental arthroplasty compared to total knee arthroplasty? Short-term outcomes of a prospective matched study including 68 cases. | Parratte, S; Ollivier, M; Opsomer, G; Lunebourg, A; Argenson, J-N; Thienpont, E | Orthopaedics & traumatology, surgery & research : OTSR | 2015 | 10.1016/j.otsr.2015.03.019 | English |
| 33934658 | Mid- to long-term follow-up of combined small implants. | Rossi, Stefano M P; Perticarini, Loris; Clocchiatti, Susanna; Ghiara, Matteo; Benazzo, Francesco | The bone & joint journal | 2021 | 10.1302/0301-620X.103B5.BJJ-2020-0720.R3 | English |
| 24519181 | Autologous chondrocyte implantation and anteromedialization for isolated patellar articular cartilage lesions: 5- to 11-year follow-up. | Gillogly, Scott D; Arnold, Ryan M | The American journal of sports medicine | 2014 | 10.1177/0363546513519077 | English |
| 19102828 | Manipulation under anaesthesia for stiffness following knee arthroplasty. | Mohammed, Riazuddin; Syed, Shakir; Ahmed, Naveed | Annals of the Royal College of Surgeons of England | 2009 | 10.1308/003588409X359321 | English |
| 33811265 | The lack of retropatellar resurfacing at index surgery is significantly associated with failure in patients following patellofemoral inlay arthroplasty: a multi-center study of more than 260 patients. | Imhoff, Andreas B; Bartsch, Eva; Becher, Christoph; Behrens, Peter; Bode, Gerrit; Cotic, Matthias; Diermeier, Theresa; Falk, Holger; Feucht, Matthias J; Haupt, Ulrich; Hinterwimmer, Stefan; Holz, Johannes; Hutter, Rene; Kaiser, Rene; Knoblauch, Tobias; Nebelung, Wolfgang; Niemeyer, Philipp; O'Donnel, Turlough; Pagenstert, Geert; Patzer, Thilo; Rose, Tim; Rupp, Marco C; Tischer, Thomas; Venjakob, Arne J; Vogt, Stephan; Pogorzelski, Jonas | Knee surgery, sports traumatology, arthroscopy : official journal of the ESSKA | 2022 | 10.1007/s00167-021-06544-5 | English |
| 34792611 | Insufficient evidence to confirm benefits of custom partial knee arthroplasty: a systematic review. | Demey, Guillaume; Muller, Jacobus H; Liebensteiner, Michael; Pilot, Peter; Nover, Luca; Kort, Nanne | Knee surgery, sports traumatology, arthroscopy : official journal of the ESSKA | 2022 | 10.1007/s00167-021-06766-7 | English |
| 30315327 | Derotational osteotomy at the distal femur is effective to treat patients with patellar instability. | Imhoff, Florian B; Cotic, Matthias; Liska, Franz; Dyrna, Felix G E; Beitzel, Knut; Imhoff, Andreas B; Herbst, Elmar | Knee surgery, sports traumatology, arthroscopy : official journal of the ESSKA | 2019 | 10.1007/s00167-018-5212-z | English |
| 19632120 | Return to work following knee arthroplasty. | Foote, J A J; Smith, H K; Jonas, S C; Greenwood, R; Weale, A E | The Knee | 2010 | 10.1016/j.knee.2009.06.001 | English |
| 28623415 | A stand-alone lateral condyle-elevating trochlear osteotomy leads to high residual instability but no excessive increase in patellofemoral osteoarthritis at 12-year follow-up. | Tigchelaar, S; van Sambeeck, J; Koeter, S; van Kampen, A | Knee surgery, sports traumatology, arthroscopy : official journal of the ESSKA | 2018 | 10.1007/s00167-017-4602-y | English |
| 32475247 | Patterns of cartilage loss and anterior cruciate ligament status in end-stage osteoarthritis of the knee. | Scott, Chloe E H; Holland, George; Krahelski, Oliver; Murray, Iain R; Keating, John F; Keenan, Oisin J F | The bone & joint journal | 2020 | 10.1302/0301-620X.102B6.BJJ-2019-1434.R1 | English |
| 33126284 | Robotic-Assisted Patellofemoral Replacement-Correlation of Preoperative Planning with Intraoperative Implant Position and Early Clinical Experience: A Minimum 2-Year Follow-up. | Selvaratnam, Veenesh; Cattell, Andrew; Eyres, Keith S; Toms, Andrew D; Phillips, Jonathan R P; Mandalia, Vipul I | The journal of knee surgery | 2022 | 10.1055/s-0040-1716848 | English |
| 33272080 | Has the modern design of Attune total knee replacement improved outcome in patients with isolated patellofemoral arthritis? | Ashraf, Yasir; Iqbal, Hafiz Javaid; Senevirathana, Shanaka; Ashraf, Tanweer | Journal of orthopaedic surgery (Hong Kong) | 2020 | 10.1177/2309499020975553 | English |
| 29299472 | Does a simple syringe applicator enhance bone cement set up time in knee arthroplasty? | Sodhi, Nipun; Dalton, Sarah E; Khlopas, Anton; Sultan, Assem A; Curtis, Gannon L; Harb, Matthew A; Naziri, Qais; Newman, Jared M; Barrington, John W; Mont, Michael A | Annals of translational medicine | 2017 | 10.21037/atm.2017.11.17 | English |
| 35315804 | Patellofemoral Arthroplasty Results in Better Time-weighted Patient-reported Outcomes After 6 Years than TKA: A Randomized Controlled Trial. | Odgaard, Anders; Kappel, Andreas; Madsen, Frank; Kristensen, Per Wagner; Stephensen, Snorre; Attarzadeh, Amir Pasha | Clinical orthopaedics and related research | 2022 | 10.1097/CORR.0000000000002178 | English |
| 24980643 | Are revisions of patellofemoral arthroplasties more like primary or revision TKAs. | Parratte, Sebastien; Lunebourg, Alexandre; Ollivier, Matthieu; Abdel, Matthew P; Argenson, Jean-Noel A | Clinical orthopaedics and related research | 2015 | 10.1007/s11999-014-3756-x | English |
| 31135543 | The Dubousset Functional Test is a Novel Assessment of Physical Function and Balance. | Diebo, Bassel G; Challier, Vincent; Shah, Neil V; Kim, David; Murray, Daniel P; Kelly, John J; Lafage, Renaud; Paulino, Carl B; Passias, Peter G; Schwab, Frank J; Lafage, Virginie | Clinical orthopaedics and related research | 2019 | 10.1097/CORR.0000000000000820 | English |
| 31136442 | What Is the Risk of Repeat Revision When Patellofemoral Replacement Is Revised to TKA? An Analysis of 482 Cases From a Large National Arthroplasty Registry. | Lewis, Peter L; Graves, Stephen E; Cuthbert, Alana; Parker, David; Myers, Peter | Clinical orthopaedics and related research | 2019 | 10.1097/CORR.0000000000000541 | English |
| 36743281 | Combined MPFL Reconstruction with Tibial Tubercle Osteotomy and Repair of Patellar Cartilage Defect with Particulated Juvenile Articular Cartilage. | Dennis, Elizabeth R; Marmor, William A; Shubin Stein, Beth E | JBJS essential surgical techniques | 2022 | 10.2106/JBJS.ST.21.00013 | English |

| **Table S6 List of bibliographies that meet the criteria and are included in bibliometric analysis** | | | | |
| --- | --- | --- | --- | --- |
| **Article Title** | **Authors** | **Journal** | **Publication Year** | **DOI** |
| Patellofemoral arthroplasty: a systematic review of the literature | Tarassoli, Payam;Punwar, Shahid; Khan, Wasim;Johnstone, David | OPEN ORTHOP J | 2012 | 10.2174/1874325001206010340 |
| Early results with a total patellofemoral joint replacement arthroplasty prosthesis | Merchant Ac | J ARTHROPLASTY | 2004 | 10.1016/j.arth.2004.03.011 |
| A radiological index that influences the outcome following patellofemoral joint arthroplasty: the anterior trochlea offset ratio | Aweid O;Ahearn N;Metcalfe Aj;Eldridge J;Porteous A;Murray Jr | KNEE SURG SPORT TR A |  | 10.1007/s00167-022-07085-1 |
| Mid-term results of the fpv patellofemoral joint replacement | Al-Hadithy N;Patel R;Navadgi B;Deo S;Hollinghurst D;Satish V | KNEE | 2014 | 10.1016/j.knee.2013.08.010 |
| Treatment of unicompartmental cartilage defects of the knee with unicompartmental knee arthroplasty, patellofemoral partial knee arthroplasty or focal resurfacing | Springer B;Boettner F | LIFE-BASEL | 2021 | 10.3390/life11050394 |
| The avon patellofemoral joint replacement independent assessment of early functional outcomes | Starks I;Roberts S;White Sh | J BONE JOINT SURG BR | 2009 | 10.1302/0301-620X.91B12.23018 |
| Patellar tendon shortening following patellofemoral joint replacement | Van Engen Lah;Landman Ebm;Kleinlugtenbelt Yv;Van Jonbergen Hpw | INT ORTHOP | 2019 | 10.1007/s00264-018-4194-2 |
| The journey patellofemoral joint arthroplasty: a minimum 5 year follow-up study | Ahearn N;Metcalfe Aj;Hassaballa Ma;Porteous Aj;Robinson Jr;Murray Jr;Newman Jh | KNEE | 2016 | 10.1016/j.knee.2016.03.004 |
| Robotic assisted patellofemoral joint replacement: surgical technique, tips and tricks | Selvaratnam V;Toms Ad;Mandalia Vi | INDIAN J ORTHOP | 2022 | 10.1007/s43465-022-00746-w |
| Arthritis progression after patellofemoral joint replacement | Nicol Sg;Loveridge Jm;Weale Ae;Ackroyd Ce;Newman Jh | KNEE | 2006 | 10.1016/j.knee.2006.04.005 |
| Medium term results of avon patellofemoral joint replacement | Sarda Pk;Shetty A;Maheswaran Ss | INDIAN J ORTHOP | 2011 | 10.4103/0019-5413.83761 |
| The avon patellofemoral joint replacement five-year results from an independent centre | Odumenya M;Costa Ml;Parsons N;Achten J;Dhillon M;Krikler Sj | J BONE JOINT SURG BR | 2010 | 10.1302/0301-620X.92B1.23135 |
| In vitro simulation and quantification of wear within the patellofemoral joint replacement | Ellison P;Barton Dc;Esler C;Shaw Dl;Stone Mh;Fisher J | J BIOMECH | 2008 | 10.1016/j.jbiomech.2008.02.029 |
| The avon patellofemoral joint arthroplasty two- to 18-year results of a large single-centre cohort | Metcalfe Aj;Ahearn N;Hassaballa Ma;Parsons N;Ackroyd Ce;Murray Jr;Robinson Jr;Eldridge Jd;Porteous Aj | BONE JOINT J | 2018 | 10.1302/0301-620X.100B9.BJJ-2018-0174.R1 |
| High mid-term revision rate after treatment of large, full-thickness cartilage lesions and oa in the patellofemoral joint using a large inlay resurfacing prosthesis: hemicap-wavea (r) | Laursen Jo | KNEE SURG SPORT TR A | 2017 | 10.1007/s00167-016-4352-2 |
| Isolated patellofemoral joint arthroplasty: can preoperative bone scans predict survivorship? | Baker Jf;Caborn Dn;Schlierf Tj;Fain Tb;Smith Ls;Malkani Al | J ARTHROPLASTY | 2020 | 10.1016/j.arth.2019.08.021 |
| Medial patellofemoral ligament reconstruction for subluxating patellofemoral arthroplasty | Carmont Mr;Crane T;Thompson P;Spalding T | KNEE | 2011 | 10.1016/j.knee.2010.02.011 |
| Patellar clunk syndrome in patellofemoral arthroplasty - a case report | Sringari T;Maheswaran Ss | KNEE | 2005 | 10.1016/j.knee.2004.11.008 |
| Revision of a failed patellofemoral arthroplasty to a total knee arthroplasty | Lonner Jh;Jasko Jg;Booth Re | J BONE JOINT SURG AM | 2006 | 10.2106/JBJS.F.00282 |
| Strain shielding in distal femur after patellofemoral arthroplasty under different activity conditions | Meireles S;Completo A;Simoes Ja;Flores P | J BIOMECH | 2010 | 10.1016/j.jbiomech.2009.09.048 |
| Comparison of commercial patellofemoral arthroplasty systems on the basis of patella kinematics, peri-patellar soft tissue tension and prosthesis design | Muller Jh;Erasmus Pj;Scheffer C | J MECH MED BIOL | 2012 | 10.1142/S0219519412500868 |
| Minimally invasive robotic-assisted patellofemoral arthroplasty | Hassebrock Jd;Makovicka Jl;Wong M;Patel Ka;Scott Kl;Deckey Dg;Chhabra A | ARTHROSC TEC | 2020 | 10.1016/j.eats.2019.11.013 |
| Patellofemoral replacement with tibial tubercle osteotomy | Arvesen Je;Wyland Dj | ARTHROSC TEC | 2021 | 10.1016/j.eats.2020.09.009 |
| Long-term outcomes of patellofemoral arthroplasty | Van Jonbergen Hpw;Werkman Dm;Barnaart Lf;Van Kampen A | J ARTHROPLASTY | 2010 | 10.1016/j.arth.2009.08.023 |
| Dislocation of the mobile bearing component of a patellofemoral arthroplasty: a report of two cases | Witjes S;Van Den Broek C;Koeter S;Van Loon C | ACTA ORTHOP BELG | 2009 |  |
| Scientific evidence for the use of modern patellofemoral arthroplasty | Gupta Rr;Zywiel Mg;Leadbetter Wb;Bonutti P;Mont Ma | EXPERT REV MED DEVIC | 2010 | 10.1586/ERD.09.53 |
| Patellofemoral arthroplasty 7-year mean follow-up | Mont Ma;Johnson Aj;Naziri Q;Kolisek Fr;Leadbetter Wb | J ARTHROPLASTY | 2012 | 10.1016/j.arth.2011.07.010 |
| Ipsilateral patellofemoral arthroplasty and autogenous osteochondral femoral condylar transplantation | Lonner Jh;Mehta S;Booth Re | J ARTHROPLASTY | 2007 | 10.1016/j.arth.2005.08.012 |
| Patellofemoral arthroplasty: expert opinion | Hoogervorst P;Arendt Ea | J EXP ORTHOP | 2022 | 10.1186/s40634-022-00457-z |
| Patellofemoral arthroplasty: the impact of design on outcomes | Lonner Jh | ORTHOP CLIN N AM | 2008 | 10.1016/j.ocl.2008.02.002 |
| Patellofemoral arthroplasty - the third compartment | Lotke Pa;Lonner Jh;Nelson Cl | J ARTHROPLASTY | 2005 | 10.1016/j.arth.2005.03.011 |
| Clinical results of patellofemoral arthroplasty | Morris Mj;Lombardi Av;Berend Kr;Hurst Jm;Adams Jb | J ARTHROPLASTY | 2013 | 10.1016/j.arth.2013.05.012 |
| Advances in patellofemoral arthroplasty | Strickland Sm;Bird Ml;Christ Ab | CURR REV MUSCULOSKE | 2018 | 10.1007/s12178-018-9477-0 |
| Midterm results of modern patellofemoral arthroplasty versus total knee arthroplasty for isolated patellofemoral arthritis: systematic review and meta-analysis of comparative studies | Elbardesy H;Mcleod A;Gul R;Harty J | ARCH ORTHOP TRAUM SU | 2022 | 10.1007/s00402-021-03882-4 |
| Patellofemoral arthroplasty with a customized trochlear prosthesis | Sisto Dj;Sarin Vk | ORTHOP CLIN N AM | 2008 | 10.1016/j.ocl.2008.03.002 |
| Trochlear inclination angles in normal and dysplastic knees | Kamath Af;Slattery Tr;Levack Ae;Wu Ch;Kneeland Jb;Lonner Jh | J ARTHROPLASTY | 2013 | 10.1016/j.arth.2012.04.017 |
| Patellofemoral arthroplasty: a multi-centre study with minimum 2-year follow-up | Leadbetter Wb;Kolisek Fr;Levitt Rl;Brooker Af;Zietz P;Marker Dr;Bonutti Pm;Mont Ma | INT ORTHOP | 2009 | 10.1007/s00264-008-0692-y |
| The appropriate use of patellofemoral arthroplasty - an analysis of reported indications, contraindications, and failures | Leadbetter Wb;Ragland Ps;Mont Ma | CLIN ORTHOP RELAT R | 2005 | 10.1097/01.blo.0000172304.12533.41 |
| The clinical outcome of patellofemoral arthroplasty | Lonner Jh;Bloomfield Mr | ORTHOP CLIN N AM | 2013 | 10.1016/j.ocl.2013.03.002 |
| The warwick patellofemoral arthroplasty trial: a randomised clinical trial of total knee arthroplasty versus patellofemoral arthroplasty in patients with severe arthritis of the patellofemoral joint | Odumenya M;Mcguinness K;Achten J;Parsons N;Spalding T;Costa M | BMC MUSCULOSKEL DIS | 2011 | 10.1186/1471-2474-12-265 |
| Revision patellofemoral arthroplasty - three- to seven-year follow-up | Hendrix Mrg;Ackroyd Ce;Lonner Jh | J ARTHROPLASTY | 2008 | 10.1016/j.arth.2007.10.019 |
| Unusual mechanical complications of unicompartmental low contact stress mobile bearing patellofemoral arthroplasty: a cause for concern? | Arumilli Brb;Ng Aby;Ellis Dj;Hirst P | KNEE | 2010 | 10.1016/j.knee.2009.10.006 |
| Is there a place for patellofemoral arthroplasty | Argenson Jna;Guillaume Jm;Aubaniac Jm | CLIN ORTHOP RELAT R | 1995 |  |
| Complications after patello-femoral versus total knee replacement in the treatment of isolated patello-femoral osteoarthritis. A meta-analysis | Dy Cj;Franco N;Ma Y;Mazumdar M;Mccarthy Mm;Della Valle Ag | KNEE SURG SPORT TR A | 2012 | 10.1007/s00167-011-1677-8 |
| Computer-assisted patellofemoral arthroplasty - a mechanism for optimizing rotation | Cossey Aj;Spriggins Aj | J ARTHROPLASTY | 2006 | 10.1016/j.arth.2005.08.010 |
| Aiming for anatomical femoral axis on the coronal plane leads to good-to-excellent short-term outcomes in isolated patellofemoral arthroplasty | Vasta S;Rosi M;Tecame A;Papalia R;Adravanti P | KNEE | 2020 | 10.1016/j.knee.2020.02.016 |
| A preliminary report of patellofemoral arthroplasty in isolated patellofemoral arthritis | Gao Xa;Xu Zj;He Rx;Yan Sg;Wu Ld | CHINESE MED J-PEKING | 2010 | 10.3760/cma.j.issn.0366-6999.2010.21.013 |
| Outcome of patellofemoral arthroplasty, determinants for success | Willekens P;Victor J;Verbruggen D;Vande Kerckhove M;Van Der Straeten C | ACTA ORTHOP BELG | 2015 |  |
| Uka in combination with pfr at average 12-year follow-up | Heyse Tj;Khefacha A;Cartier P | ARCH ORTHOP TRAUM SU | 2010 | 10.1007/s00402-009-0997-3 |
| Outcome instruments for pateflofemoral arthroplasty | Paxton Ew;Fithian Dc | CLIN ORTHOP RELAT R | 2005 | 10.1097/01.blo.0000171544.38095.77 |
| Patellofemoral arthroplasty - an update | Argenson Jna;Flecher X;Parratte S;Aubaniac Jm | CLIN ORTHOP RELAT R | 2005 | 10.1097/01.blo.0000187061.27573.70 |
| Conversion of patellofemoral arthroplasty to total knee arthroplasty | Van Jonbergen Hpw;Werkman Dm;Van Kampen A | ACTA ORTHOP | 2009 | 10.1080/17453670902805031 |
| Distal femoral bone mineral density decreases following patellofemoral arthroplasty: 1-year follow-up study of 14 patients | Van Jonbergen Hpw;Koster K;Labey L;Innocenti B;Van Kampen A | BMC MUSCULOSKEL DIS | 2010 | 10.1186/1471-2474-11-74 |
| Total knee arthroplasty reduces knee extension torque in-vitro and patellofemoral arthroplasty does not | Joseph Mn;Carmont Mr;Tailor H;Stephen Jm;Amis Aa | J BIOMECH | 2020 | 10.1016/j.jbiomech.2020.109739 |
| Patella component loosening - a case report | Bloemheuvel Em;Van Rooij Wmj;Van Den Besselaar M | ACTA ORTHOP BELG | 2016 |  |
| Update on patellofemoral arthroplasty | Crowe Mm;Dahm Dl | OPER TECHN SPORT MED | 2015 | 10.1053/j.otsm.2015.05.001 |
| Patellofemoral arthroplasty | Lonner Jh | J AM ACAD ORTHOP SUR | 2007 | 10.5435/00124635-200708000-00006 |
| Combined patellofemoral arthroplasty and medial patellofemoral ligament reconstruction for chronic patellar instability with trochlear dysplasia: a report of two cases | Yamagami R;Inui H;Taketomi S;Tanaka S | MOD RHEUMATOL CASE | 2020 | 10.1080/24725625.2019.1638048 |
| Patient-related outcomes of patellofemoral arthroplasty: experience of a single center | Abeysekera Wym;Schenk W | ARTHROPLASTY | 2021 | 10.1186/s42836-021-00074-8 |
| Pre-operative patella alta does not affect midterm clinical outcomes and survivorship of patellofemoral arthroplasty | Bernard Cd;Pareek A;Sabbag Cm;Parkes Cw;Krych Aj;Cummings Nm;Dahm Dl | KNEE SURG SPORT TR A | 2021 | 10.1007/s00167-020-06205-z |
| Optimizing patellofemoral arthroplasty | Farr J;Barrett D | KNEE | 2008 | 10.1016/j.knee.2008.05.008 |
| In vivo sagittal plane kinematics of the FPV patellofemoral replacement | Monk Ap;Van Duren Bh;Pandit H;Shakespeare D;Murray Dw;Gill Hs | KNEE SURG SPORT TR A | 2012 | 10.1007/s00167-011-1717-4 |
| Four-year follow up outcome study of patellofemoral arthroplasty at a single institution | Goh Gsh;Liow Mhl;Tay Dkj;Lo Nn;Yeo Sj | J ARTHROPLASTY | 2015 | 10.1016/j.arth.2015.01.020 |
| Patellofemoral arthroplasty | Godshaw B;Kolodychuk N;Williams Gk;Browning B;Jones D | OCHSNER J | 2018 | 10.31486/toj.18.0009 |
| Patellofemoral arthroplasty changes the trochlear groove angle | Wiesner Fj;Erasmus Pj;Cho Kj;Muller Jh | J MECH MED BIOL | 2017 | 10.1142/S0219519417500683 |
| Patellofemoral arthroplasty | Lustig S | ORTHOP TRAUMATOL-SUR | 2014 | 10.1016/j.otsr.2013.06.013 |
| Design, operative technique and ten-year results of the hermes (tm) patellofemoral arthroplasty | Philippe H;Caton J | INT ORTHOP | 2014 | 10.1007/s00264-013-2158-0 |
| Patellofemoral arthroplasty influences tibiofemoral kinematics: the effect of patellar thickness | Vandenneucker H;Labey L;Victor J;Vander Sloten J;Desloovere K;Bellemans J | KNEE SURG SPORT TR A | 2014 | 10.1007/s00167-014-3160-9 |
| Results of total knee replacement for isolated patellofemoral arthritis: when not to perform a patellofemoral arthroplasty | Delanois Re;Mcgrath Ms;Ulrich Sd;Marker Dr;Seyler Tm;Bonutti Pm;Mont Ma | ORTHOP CLIN N AM | 2008 | 10.1016/j.ocl.2008.03.003 |
| Patellofemoral arthroplasty in the athlete | Farr J;Arendt E;Dahm D;Daynes J | CLIN SPORT MED | 2014 | 10.1016/j.csm.2014.03.003 |
| Return to the operating room after patellofemoral arthroplasty versus total knee arthroplasty for isolated patellofemoral arthritisa systematic review | Woon Cyl;Christ Ab;Goto R;Shanaghan K;Shubin Stein Be;Della Valle Ag | INT ORTHOP | 2019 | 10.1007/s00264-018-04280-z |
| Biomechanical behaviour of cancellous bone on patellofemoral arthroplasty with journey prosthesis | Castro Apg;Completo A;Simoes Ja;Flores P | COMPUT METHOD BIOMEC | 2015 | 10.1080/10255842.2013.870999 |
| Early revisions of the femoro-patella vialla joint replacement | Williams Dp;Pandit Hg;Athanasou Na;Murray Dw;Gibbons Clmh | BONE JOINT J | 2013 | 10.1302/0301-620X.95B6.31355 |
| Partial knee arthroplasty: patellofemoral arthroplasty and combined unicompartmental and patellofemoral arthroplasty implants - general considerations and indications, technique and clinical experience | Benazzo F;Rossi Smp;Ghiara M | KNEE | 2014 | 10.1016/S0968-0160(14)50009-9 |
| The low contact stress patellofemoral replacement high early failure rate | Charalambous Cp;Abiddin Z;Mills Sp;Rogers S;Sutton P;Parkinson R | J BONE JOINT SURG BR | 2011 | 10.1302/0301-620X.93B4.25899 |
| Unique combination of patellofemoral joint arthroplasty with osteochondral autograft transfer system (oats) - a case series of six knees in five patients | Unnithan A;Jimulia T;Mohammed R;Learmonth Dja | KNEE | 2008 | 10.1016/j.knee.2008.01.007 |
| Total knee arthroplasty for isolated patellofemoral arthritis in younger patients | Meding Jb;Wing Jt;Keating Em;Ritter Ma | CLIN ORTHOP RELAT R | 2007 | 10.1097/BLO.0b013e3181576069 |
| The avon patellofemoral arthroplasty - five-year survivorship and functional results | Ackroyd Ce;Newman Jh;Evans R;Eldridge Jdj;Joslin Cc | J BONE JOINT SURG BR | 2007 | 10.1302/0301-620X.89B3.18062 |
| Patellar polyethylene spinout after low-contact stress, high-congruity, mobile-bearing patellofemoral arthroplasty | Amanatullah Df;Jamali Aa | ORTHOPEDICS | 2012 | 10.3928/01477447-20120123-27 |
| The lubinus patellofemoral arthroplasty: a series of 17 cases | Board Tn;Mahmood A;Ryan Wg;Banks Aj | ARCH ORTHOP TRAUM SU | 2004 | 10.1007/s00402-004-0645-x |
| The patellofemoral joint - its function and influence in patellar replacement design | Pappas Mj;Buechel Ff | J ORTHOP RHEUMATOL | 1994 |  |
| The effect of axial rotation of the anterior resection plane in patellofemoral arthroplasty | Cho Kj;Erasmus Pj;Muller Jh | KNEE | 2016 | 10.1016/j.knee.2016.04.006 |
| Coronal alignment of patellofemoral arthroplasty | Thienpont E;Lonner Jh | KNEE | 2014 | 10.1016/S0968-0160(14)50011-7 |
| The midterm results of a cohort study of patellofemoral arthroplasty from a non-designer centre using an asymmetric trochlear prosthesis | Rammohan R;Gupta S;Lee Pyf;Chandratreya A | KNEE | 2019 | 10.1016/j.knee.2019.10.026 |
| Patellofemoral arthroplasty, where are we today? | Lustig S;Magnussen Ra;Dahm Dl;Parker D | KNEE SURG SPORT TR A | 2012 | 10.1007/s00167-012-1948-z |
| Patellofemoral replacement the third compartment | Hofmann Aa;Mccandless Jb;Shaeffer Jf;Magee Th | BONE JOINT J | 2013 | 10.1302/0301-620X.95B11.32985 |
| Patellar fracture following patellofemoral arthroplasty | King Ah;Engasser Wm;Sousa Pl;Arendt Ea;Dahm Dl | J ARTHROPLASTY | 2015 | 10.1016/j.arth.2015.02.007 |
| Long-term results with the first patellotemoral prosthesis | Cartier P;Sanouiller Jl;Khefacha A | CLIN ORTHOP RELAT R | 2005 | 10.1097/01.blo.0000171918.24998.d1 |
| Patellofemoral arthroplasty - pros, cons, and design considerations | Lonner Jh | CLIN ORTHOP RELAT R | 2004 | 10.1097/01.blo.0000148896.25708.51 |
| Long-term results of patellofemoral arthroplasty - a report of 56 arthroplasties with 17 years of follow-up | Kooijman Hj;Driessen Appm;Van Horn Jr | J BONE JOINT SURG BR | 2003 | 10.1302/0301-620X.85B6.13741 |
| Prospective clinical and radiological two-year results after patellofemoral arthroplasty using an implant with an asymmetric trochlea design | Beitzel K;Schottle Pb;Cotic M;Dharmesh V;Imhoff Ab | KNEE SURG SPORT TR A | 2013 | 10.1007/s00167-012-2022-6 |
| A modular prosthesis for patellofemoral arthroplasty - design and initial results | Merchant Ac | CLIN ORTHOP RELAT R | 2005 | 10.1097/01.bio.0000171917.47869.6c |
| Midterm clinical results of the autocentric ii patellofemoral prosthesis | Van Wagenberg Jmf;Speigner B;Gosens T;Malefijt Jd | INT ORTHOP | 2009 | 10.1007/s00264-009-0719-z |
| Isolated patellofemoral arthroplasty reproduces natural patellofemoral joint kinematics when the patella is resurfaced | Vandenneucker H;Labey L;Vander Sloten J;Desloovere K;Bellemans J | KNEE SURG SPORT TR A | 2016 | 10.1007/s00167-014-3415-5 |
| The present situation of patellofemoral arthroplasty in the management of solitary patellofemoral osteoarthritis | Rodriguez-Merchan Ec | ARCH BONE JT SURG-AB | 2020 | 10.22038/ABJS.2019.14125 |
| Significant functional improvement at 2 years after isolated patellofemoral arthroplasty with an onlay trochlear implant, but low mental health scores predispose to dissatisfaction | Kazarian Gs;Tarity Td;Hansen En;Cai J;Lonner Jh | J ARTHROPLASTY | 2016 | 10.1016/j.arth.2015.08.033 |
| Functional relevance of patellofemoral thickness before and after unicompartmental patellofemoral replacement | Mofidi A;Bajada S;Holt Md;Davies Ap | KNEE | 2012 | 10.1016/j.knee.2011.03.002 |
| Development of a new femoral component for patellofemoral prosthesis | Castro A;Flores P;Completo A;Simoes Ja | 2012 IEEE 2ND PORTUGUESE MEETING IN BIOENGINEERING (ENBENG) | 2012 |  |
| Patellofemoral arthritis and its management with isolated patellofemoral replacement: a personal experience | Newman Jh | ORTHOPEDICS | 2007 |  |
| The richards type ii patellofemoral arthroplasty - 26 cases followed for 1-20 years | De Winter Weaejm;Feith R;Van Loon Cjm | ACTA ORTHOP SCAND | 2001 |  |
| In vivo sagittal plane kinematics of the avon patellofemoral arthroplasty | Hollinghurst D;Stoney J;Ward T;Pandit H;Beard D;Murray Dw | J ARTHROPLASTY | 2007 | 10.1016/j.arth.2006.02.160 |
| Avon patellofemoral arthroplasty. Five year survivorship and functional results from an independent centre | Reeve Wje;Guyver Pm;Vaughan Ajc;Farmer K;Lee As | ACTA ORTHOP BELG | 2021 |  |
| Evidence of trochlear dysplasia in patellofemoral arthroplasty designs | Saffarini M;Ntagiopoulos Pg;Demey G;Le Negaret B;Dejour Dh | KNEE SURG SPORT TR A | 2014 | 10.1007/s00167-014-2967-8 |
| Patient-reported outcomes and risk factors for decreased improvement after patellofemoral arthroplasty | Dai Yk;Diao Nc;Lin W;Yang Gm;Kang Hj;Wang F | J KNEE SURG |  | 10.1055/s-0041-1735159 |
| Effect of patellofemoral arthroplasty on patellar height in patients with patellofemoral osteoarthritis | Lee H;Fletcher C;Hartwell M;Strickland Sm | J KNEE SURG |  | 10.1055/s-0042-1755354 |
| Onlay patellofemoral arthroplasty in patients with isolated patellofemoral arthritis: a systematic review | Villa Jc;Paoli Ar;Nelson-Williams Hw;Badr Rn;Harper Kd | J ARTHROPLASTY | 2021 | 10.1016/j.arth.2021.02.054 |
| Early outcomes of an anatomic trochlear-cutting patellofemoral arthroplasty: patient selection is key | Dejour D;Saffarini M;Malemo Y;Pungitore M;Valluy J;Nover L;Demey G | KNEE SURG SPORT TR A | 2019 | 10.1007/s00167-019-05368-8 |
| Survivorship and functional outcomes of patellofemoral arthroplasty: a systematic review | Van Der List Jp;Chawla H;Zuiderbaan Ha;Pearle Ad | KNEE SURG SPORT TR A | 2017 | 10.1007/s00167-015-3878-z |
| Patellofemoral arthroplasty: outcomes and factors associated with early progression of tibiofemoral arthritis | Dahm Dl;Kalisvaart Mm;Stuart Mj;Slettedahl Sw | KNEE SURG SPORT TR A | 2014 | 10.1007/s00167-014-3202-3 |
| The short-term effectiveness and safety of second-generation patellofemoral arthroplasty and total knee arthroplasty on isolated patellofemoral osteoarthritis: a systematic review and meta-analysis | Li Cx;Li Zz;Shi Lj;Gao Fq;Sun W | J ORTHOP SURG RES | 2021 | 10.1186/s13018-021-02509-z |
| Inadequacy of computed tomography for pre-operative planning of patellofemoral arthroplasty | Saffarini M;Muller Jh;La Barbera G;Hannink G;Cho Kj;Toanen C;Dejour D | KNEE SURG SPORT TR A | 2018 | 10.1007/s00167-017-4474-1 |
| Modelling and simulation of alternative designs for the femur-implant interface of journey patellofemoral prosthesis | Castro Apg;Completo A;Simoes Ja;Flores P | P I MECH ENG L-J MAT | 2019 | 10.1177/1464420718774074 |
| Patellofemoral arthroplasty conversion to total knee arthroplasty: retrieval analysis and clinical correlation | Christ Ab;Baral E;Koch C;Stein Bes;Della Valle Ag;Strickland Sm | KNEE | 2017 | 10.1016/j.knee.2017.06.015 |
| Patient satisfaction reporting for patellofemoral arthroplasty is significantly lacking: a systematic review | Tishelman Jc;Kahlenberg Ca;Nwachukwu Bu;Gruskay J;Strickland Sm | PHYSICIAN SPORTSMED | 2019 | 10.1080/00913847.2019.1580913 |
| Obesity does not affect patient-reported outcomes following patellofemoral arthroplasty | Tishelman Jc;Pyne A;Kahlenberg Ca;Gruskay Ja;Strickland Sm | J KNEE SURG | 2022 | 10.1055/s-0040-1713862 |
| Patellofemoral joint replacement - mean five year follow-up | Ajnin S;Buchanan D;Arbuthnot J;Fernandes R | KNEE | 2018 | 10.1016/j.knee.2018.08.014 |
| Mid-term clinical, functional, and radiographic outcomes of 105 gender-specific patellofemoral arthroplasties, with or without the association of medial unicompartmental knee arthroplasty | Romagnoli S;Marullo M | J ARTHROPLASTY | 2018 | 10.1016/j.arth.2017.10.019 |
| Patellofemoral arthroplasty surgical technique: lateral or medial parapatellar approach | Jeong Sh;Schneider B;Pyne As;Tishelman Jc;Strickland Sm | J ARTHROPLASTY | 2020 | 10.1016/j.arth.2020.04.026 |
| Patellofemoral arthroplasty versus total knee arthroplasty for patients with patellofemoral osteoarthritis | Clement Nd;Howard Ta;Immelman Rj;Macdonald D;Patton Jt;Lawson Gm;Burnett R | BONE JOINT J | 2019 | 10.1302/0301-620X.101B1.BJJ-2018-0654.R2 |
| Correction of patellofemoral malalignment with patellofemoral arthroplasty | Valoroso M;Saffarini M;La Barbera G;Toanen C;Hannink G;Nover L;Dejour Dh | J ARTHROPLASTY | 2017 | 10.1016/j.arth.2017.06.048 |
| Patellofemoral arthroplasty in combination with high tibial osteotomy can achieve good outcome for patients with medial-patellofemoral osteoarthritis | Peng Yg;Lin W;Zhang Yf;Wang F | FRONT SURG | 2022 | 10.3389/fsurg.2022.999208 |
| Obesity and the absence of trochlear dysplasia increase the risk of revision in patellofemoral arthroplasty | Liow Mhl;Goh Gsh;Tay Dkj;Chia Sl;Lo Nn;Yeo Sj | KNEE | 2016 | 10.1016/j.knee.2015.05.009 |
| Patellofemoral arthroplasty improves patellofemoral alignment in patients with patellofemoral osteoarthritis with trochlear dysplasia | Yang Gm;Wang J;Dai Yk;Lin W;Niu Jh;Wang F | J KNEE SURG | 2022 | 10.1055/s-0040-1715099 |
| Outcomes of patellofemoral arthroplasty based on radiographic severity | Dedeugd Cm;Pareek A;Krych Aj;Cummings Nm;Dahm Dl | J ARTHROPLASTY | 2017 | 10.1016/j.arth.2016.11.006 |
| Custom patellofemoral arthroplasty of the knee | Sisto Dj;Sarin Vk | J BONE JOINT SURG AM | 2006 | 10.2106/JBJS.E.00382 |
| Hermes patellofemoral arthroplasty: annual revision rate and clinical results after two to 20 years of follow-up | Bohu Y;Klouche S;Sezer Hb;Gerometta A;Lefevre N;Herman S | KNEE | 2019 | 10.1016/j.knee.2019.01.014 |
| A matched-pair comparison of inlay and onlay trochlear designs for patellofemoral arthroplasty: no differences in clinical outcome but less progression of osteoarthritis with inlay designs | Feucht Mj;Cotic M;Beitzel K;Baldini Jf;Meidinger G;Schottle Pb;Imhoff Ab | KNEE SURG SPORT TR A | 2017 | 10.1007/s00167-015-3733-2 |
| Midterm outcome of avon patellofemoral arthroplasty for posttraumatic unicompartmental osteoarthritis | Konan S;Haddad Fs | J ARTHROPLASTY | 2016 | 10.1016/j.arth.2016.06.005 |
| Is anterior knee pain a predisposing factor to patellofemoral osteoarthritis? | Utting Mr;Davies G;Newman Jh | KNEE | 2005 | 10.1016/j.knee.2004.12.006 |
| Patellofemoral arthroplasty - a 2- to 18-year followup study | Krajcaradcliffe Jb;Coker Tp | CLIN ORTHOP RELAT R | 1996 |  |
| Long-term results of compartmental arthroplasties of the knee long term results of partial knee arthroplasty | Parratte S;Ollivier M;Lunebourg A;Abdel Mp;Argenson Jn | BONE JOINT J | 2015 | 10.1302/0301-620X.97B10.36426 |
| Trochlear groove alignment measurement method for surgical applications | Cho Kj;Muller Jh;Erasmus Pj | J MECH MED BIOL | 2015 | 10.1142/S0219519415400333 |
| Patellofemoral arthroplasty in the treatment of patellofemoral arthritis: rationale and outcomes in younger patients | Leadbetter Wb | ORTHOP CLIN N AM | 2008 | 10.1016/j.ocl.2008.04.001 |
| Return to work following knee arthroplasty | Foote Jaj;Smith Hk;Jonas Sc;Greenwood R;Weale Ae | KNEE | 2010 | 10.1016/j.knee.2009.06.001 |
| Patellofemoral arthroplasty: short-term complications and risk factors | Rezzadeh K;Behery Oa;Kester Bs;Dogra T;Vigdorchik J;Schwarzkopf R | J KNEE SURG | 2020 | 10.1055/s-0039-1688960 |
| Patellofemoral joint replacement, an evolving concept | Borus T;Brilhault J;Confalonieri N;Johnson D;Thienpont E | KNEE | 2014 | 10.1016/S0968-0160(14)50010-5 |
| Annual revision rates of partial versus total knee arthroplasty: a comparative meta-analysis | Chawla H;Van Der List Jp;Christ Ab;Sobrero Mr;Zuiderbaan Ha;Pearle Ad | KNEE | 2017 | 10.1016/j.knee.2016.11.006 |
| Development and early results of a new patellofemoral arthroplasty | Ackroyd Ce | CLIN ORTHOP RELAT R | 2005 | 10.1097/01.blo.0000171914.94503.d1 |
| Failure modes of patellofemoral arthroplasty-registries vs. Clinical studies: a systematic review | Bendixen Nb;Eskelund Pw;Odgaard A | ACTA ORTHOP | 2019 | 10.1080/17453674.2019.1634865 |
| Joint awareness after patellofemoral arthroplasty evaluated with the forgotten joint score: a comparison study | Lin W;Dai Yk;Dong Cl;Piao K;Hao K;Wang F | ORTHOP SURG | 2021 | 10.1111/os.12921 |
| Patellofemoral arthroplasty: obesity linked to high risk of revision and progression of medial tibiofemoral osteoarthritis | Marullo M;Bargagliotti M;Vigano M;Lacagnina C;Romagnoli S | KNEE SURG SPORT TR A | 2022 | 10.1007/s00167-022-06947-y |
| Smoking, unemployment, female sex, obesity, and medication use yield worse outcomes in patellofemoral arthroplasty | Desai Vs;Pareek A;Dedeugd Cm;Sabbag Od;Krych Aj;Cummings Nm;Dahm Dl | KNEE SURG SPORT TR A | 2020 | 10.1007/s00167-019-05704-y |
| Robotic-assisted patellofemoral replacement-correlation of preoperative planning with intraoperative implant position and early clinical experience: a minimum 2-year follow-up | Selvaratnam V;Cattell A;Eyres Ks;Toms Ad;Phillips Jrp;Mandalia Vi | J KNEE SURG | 2022 | 10.1055/s-0040-1716848 |
| Classification of combined partial knee arthroplasty | Garner A;Van Arkel Rj;Cobb J | BONE JOINT J | 2019 | 10.1302/0301-620X.101B8.BJJ-2019-0125.R1 |
| Patellofemoral arthroplasty versus total knee arthroplasty for isolated patellofemoral osteoarthritis: a systematic review and meta-analysis | Peng Gr;Liu M;Guan Zh;Hou Yf;Liu Q;Sun Xb;Zhu Xy;Feng Wj;Zeng Jc;Zhong Zr;Zeng Yr | J ORTHOP SURG RES | 2021 | 10.1186/s13018-021-02414-5 |
| Arthroscopic debridement, facetectomy, and synovectomy for isolated patellofemoral osteoarthritis | Zhao Jz | ARTHROSC TEC | 2021 | 10.1016/j.eats.2021.08.021 |
| Similar postoperative patient-reported outcome in both second generation patellofemoral arthroplasty and total knee arthroplasty for treatment of isolated patellofemoral osteoarthritis: a systematic review | Bunyoz Ki;Lustig S;Troelsen A | KNEE SURG SPORT TR A | 2019 | 10.1007/s00167-018-5151-8 |
| Rotational alignment of the femoral trochlea in asians: implication on implant choice and position for managing isolated patellofemoral osteoarthritis | Li Mkl;Wan Syc;Lo Kch;Hung Yw;Fan Jch | J ORTHOP TRAUMA REHA | 2022 | 10.1177/22104917221085718 |
| Mri after patellofemoral replacement: the preserved compartments | Heyse Tj;Figiel J;Hahnlein U;Timmesfeld N;Lakemeier S;Schofer Md;Fuchs-Winkelmann S;Efe T | EUR J RADIOL | 2012 | 10.1016/j.ejrad.2011.06.012 |
| Patellar button compatibility in the conversion of patellofemoral arthroplasty to a total knee arthroplasty: a review of the contemporary literature | Mcdonald Lk;Kurmis Ap | J ORTHOP SURG-HONG K | 2022 | 10.1177/10225536221084147 |
| Complications in patellofemoral surgery | Tompkins M;Arendt Ea | SPORTS MED ARTHROSC | 2012 | 10.1097/JSA.0b013e31825c74cf |
| Why do patellofemoral arthroplasties fail today? A systematic review | Van Der List Jp;Chawla H;Villa Jc;Pearle Ad | KNEE | 2017 | 10.1016/j.knee.2015.11.002 |
| Knee arthroplasty utilization trends from 2010 to 2019 | Fuller Si;Cohen Js;Malyavko A;Agarwal Ar;Stake S;Golladay Gj;Thakkar Sc | KNEE | 2022 | 10.1016/j.knee.2022.09.006 |
| A 21 % conversion rate to total knee arthroplasty of a first-generation patellofemoral prosthesis at a mean follow-up of 9.7 years | Hoogervorst P;De Jong Rj;Hannink G;Van Kampen A | INT ORTHOP | 2015 | 10.1007/s00264-015-2941-1 |
| The clinical outcome of patellofemoral arthroplasty vs total knee arthroplasty in patients younger than 55 years | Kamikovski I;Dobransky J;Dervin Ge | J ARTHROPLASTY | 2019 | 10.1016/j.arth.2019.07.016 |
| The correct rotation of the femoral component in patellofemoral replacement a laboratory assessment of a surgical technique | Clark Da;Upadhyay N;Gillespie G;Wakeley C;Eldridge Jd | J BONE JOINT SURG BR | 2012 | 10.1302/0301-620X.94B12.29506 |
| Outcomes and complications of unicondylar arthroplasty | Riff Aj;Sah Ap;Della Valle Cj | CLIN SPORT MED | 2014 | 10.1016/j.csm.2013.06.005 |
| The combination of inlay patellofemoral arthroplasty and medial unicompartmental knee arthroplasty versus total knee arthroplasty for mediopatellofemoral osteoarthritis: a comparison of mid-term outcomes | Uluyardimci E;Isik C;Tahta M;Emre F;Cepni S;Oltulu I | J ARTHROPLASTY | 2019 | 10.1016/j.arth.2019.06.043 |
| Mid- to long-term follow-up of combined small implants a third-generation patellofemoral arthroplasty associated with a medial or lateral unicompartmental knee arthroplasty | Rossi Smp;Perticarini L;Clocchiatti S;Ghiara M;Benazzo F | BONE JOINT J | 2021 | 10.1302/0301-620X.103B5.BJJ-2020-0720.R3 |
| Surgical technique in patellofemoral arthroplasty | Remy F | ORTHOP TRAUMATOL-SUR | 2019 | 10.1016/j.otsr.2018.05.020 |
| The PAT randomized clinical trial total knee arthroplasty versus patellofemoral arthroplasty in patients with severe arthritis of the patellofemoral joint | Joseph Mn;Achten J;Parsons Nr;Costa Ml | BONE JOINT J | 2020 | 10.1302/0301-620X.102B3.BJJ-2019-0723.R1 |
| No bias for developer publications and no difference between first-generation trochlear-resurfacing versus trochlear-cutting implants in 15,306 cases of patellofemoral joint arthroplasty | Reihs B;Reihs F;Labek G;Hochegger M;Leithner A;Bohler N;Sadoghi P | KNEE SURG SPORT TR A | 2018 | 10.1007/s00167-017-4692-6 |
| Arthroplasty - current strategies for the management of knee osteoarthritis | Ahmad Ss;Gantenbein B;Evangelopoulos Ds;Schwienbacher S;Schar Mo;Kohlhof H;Kohl S | SWISS MED WKLY | 2015 | 10.4414/smw.2015.14096 |
| Preoperative patellofemoral anatomy affects failure rate after isolated patellofemoral inlay arthroplasty | Feucht Mj;Lutz Pm;Ketzer C;Rupp Mc;Cotic M;Imhoff Ab;Pogorzelski J | ARCH ORTHOP TRAUM SU | 2020 | 10.1007/s00402-020-03651-9 |
| Alternatives to patellofemoral arthroplasty | Fulkerson Jp | CLIN ORTHOP RELAT R | 2005 | 10.1097/01.bio.0000172305.20156.ba |
| Anterior knee pain: diagnosis and treatment | Post Wr | J AM ACAD ORTHOP SUR | 2005 | 10.5435/00124635-200512000-00006 |
| No Major Functional Benefit After Bicompartmental Knee Arthroplasty Compared to Total Knee Arthroplasty at 5-Year Follow-Up | Daniel Schrednitzki; Alexander Beier; Axel Marx; Andreas M Halder | J ARTHROPLASTY | 2020 | 10.1016/j.arth.2020.07.003 |
| Patellofemoral replacement (reprinted from clin. Orthop vol. 144, pg 98-102, 1979) | Blazina Me;Fox Jm;Del Pizzo W;Broukhim B;Ivey Fm | CLIN ORTHOP RELAT R | 2005 | 10.1097/01.blo.0000172301.66790.62 |
| Knee osteoarthritis: a review of management options | Hussain Sm;Neilly Dw;Baliga S;Patil S;Meek Rmd | SCOT MED J | 2016 | 10.1177/0036933015619588 |
| Patellofemoral arthroplasty is cheaper and more effective in the short term than total knee arthroplasty for isolated patellofemoral osteoarthritis: cost-effectiveness analysis based on a randomized trial | Fredborg C;Odgaard A;Sorensen J | BONE JOINT J | 2020 | 10.1302/0301-620X.102B4.BJJ-2018-1580.R3 |
| Operative management of patellofemoral pain with degenerative arthrosis | Herrenbruck Tm;Mullen Dj;Parker Rd | SPORTS MED ARTHROSC | 2001 | 10.1097/00132585-200110000-00009 |
| Patellofemoral arthroplasty | Odgaard A;Eldridge J;Madsen F | JBJS ESSENT SURG TEC | 2019 | 10.2106/JBJS.ST.18.00094 |
| Medial bicompartmental arthroplasty patients display more normal gait and improved satisfaction, compared to matched total knee arthroplasty patients | Garner Aj;Dandridge Ow;Van Arkel Rj;Cobb Jp | KNEE SURG SPORT TR A |  | 10.1007/s00167-021-06773-8 |
| The lubinus patellofemoral arthroplasty - a five- to ten-year prospective study | Tauro B;Ackroyd Ce;Newman Jh;Shah Na | J BONE JOINT SURG BR | 2001 | 10.1302/0301-620X.83B5.11577 |
| Insufficient evidence to confirm benefits of custom partial knee arthroplasty: a systematic review | Demey G;Muller Jh;Liebensteiner M;Pilot P;Nover L;Kort N | KNEE SURG SPORT TR A | 2022 | 10.1007/s00167-021-06766-7 |
| Medium-term results of patellofemoral joint arthroplasty | Mohammed R;Jimulia T;Durve K;Bansal M;Green M;Learmonth D | ACTA ORTHOP BELG | 2008 |  |
| Patellofemoral joint replacement | Van Jonergen Hpw;Van Lingen Cp | MINERVA ORTOP TRAUMA | 2010 |  |
| Aseptic loosening of the patellar component at the cement-implant interface | Rath Nk;Dudhniwala Ag;White Sp;Forster Mc | KNEE | 2012 | 10.1016/j.knee.2011.08.006 |
| Patient-reported outcome measures (proms) in patients undergoing patellofemoral arthroplasty and total knee replacement: a comparative study | Perrone Fl;Baron S;Suero Em;Lausmann C;Kendof D;Zahar A;Gehrke T;Citak M | TECHNOL HEALTH CARE | 2018 | 10.3233/THC-181185 |
| Treatment of patello-femoral arthritis using the lubinus patello-femoral arthroplasty: a retrospective review | Smith Am;Peckett Wrc;Butler-Manuel Pa;Venu Km;D'Arcy Jc | KNEE | 2002 | 10.1016/S0968-0160(01)00127-2 |
| Short-term revision risk of patellofemoral arthroplasty is high: an analysis from eight large arthroplasty registries | Lewis Pl;Tudor F;Lorimer M;Mckie J;Bohm E;Robertsson O;Makela Kt;Haapakoski J;Furnes O;Bartz-Johannessen C;Nelissen Rghh;Van Steenbergen Ln;Fithian Dc;Prentice Ha | CLIN ORTHOP RELAT R | 2020 | 10.1097/CORR.0000000000001268 |
| Outcomes of total knee replacement after patellofemoral arthroplasty | Hutt J;Dodd M;Bourke H;Bell J | J KNEE SURG | 2013 | 10.1055/s-0032-1329233 |
| Survival of bicompartmental knee arthroplasty at 5 to 23 years | Parratte S;Pauly V;Aubaniac Jm;Argenson Jna | CLIN ORTHOP RELAT R | 2010 | 10.1007/s11999-009-1018-0 |
| Patella tracking and patella contact pressure in modular patellofemoral arthroplasty: a biomechanical in vitro analysis | Calliess T;Ettinger M;Schado S;Becher C;Hurschler C;Ostermeier S | ARCH ORTHOP TRAUM SU | 2016 | 10.1007/s00402-016-2451-7 |
| The lubinus patellofemoral arthroplasty: a five- to ten-year prospective study - reply | Ackroyd C;Newman Jh | J BONE JOINT SURG BR | 2002 | 10.1302/0301-620X.84B2.0840307a |
| Mobile-bearing, congruent patellofemoral prosthesis: short-term results | Yadav B;Shaw D;Radcliffe G;Dachepalli S;Kluge W | J ORTHOP SURG-HONG K | 2012 | 10.1177/230949901202000317 |
| Knee osteoarthritis and role for surgical intervention: lessons learned from randomized clinical trials and population-based cohorts | Buchbinder R;Richards B;Harris I | CURR OPIN RHEUMATOL | 2014 | 10.1097/BOR.0000000000000022 |
| Isolated patellofemoral osteoarthritis | Van Jonbergen Hpw;Poolman Rw;Van Kampen A | ACTA ORTHOP | 2010 | 10.3109/17453671003628756 |
| Femoral component rotation in patellofemoral joint replacement | Van Jonbergen Hpw;Westerbeek Re | KNEE | 2018 | 10.1016/j.knee.2018.02.007 |
| Association and impact of patellofemoral dysplasia on patellofemoral arthropathy and arthroplasty | Mofidi A;Veravalli K;Jinnah Rh;Poehling Gg | KNEE | 2014 | 10.1016/j.knee.2013.09.009 |
| Early post-operative opioid consumption: a comparison between medial unicompartmental, patellofemoral, and total knee arthroplasty | Melnic Cm;Kazarian Er;Dwyer Mk;Domingo-Johnson El;Freiberg Aa;Bedair Hs | KNEE | 2019 | 10.1016./j.knee.2019.06.016 |
| Prospective evaluation of anatomic patellofemoral inlay resurfacing: clinical, radiographic, and sports-related results after 24 months | Imhoff Ab;Feucht Mj;Meidinger G;Schottle P;Cotic M | KNEE SURG SPORT TR A | 2015 | 10.1007/s00167-013-2786-3 |
| Short-term outcomes of robotically assisted patello-femoral arthroplasty | Turktas U;Piskin A;Poehling Gg | INT ORTHOP | 2016 | 10.1007/s00264-015-2786-7 |
| High patient satisfaction with significant improvement in knee function and pain relief after mid-term follow-up in patients with isolated patellofemoral inlay arthroplasty | Imhoff Ab;Feucht Mj;Bartsch E;Cotic M;Pogorzelski J | KNEE SURG SPORT TR A | 2019 | 10.1007/s00167-018-5173-2 |
| Medical interventions for patellofemoral pain and patellofemoral osteoarthritis: a systematic review | Macri Em;Hart Hf;Thwaites D;Barton Cj;Crossley Km;Bierma-Zeinstra Sma;Van Middelkoop M | J CLIN MED | 2020 | 10.3390/jcm9113397 |
| Reliable improvements in participation in low-impact sports following implantation of a patellofemoral inlay arthroplasty at mid-term follow-up | Pogorzelski J;Rupp Mc;Ketzer C;Cotic M;Lutz P;Beeck S;Imhoff Ab;Feucht Mj | KNEE SURG SPORT TR A | 2021 | 10.1007/s00167-020-06245-5 |
| Biomechanics of medial unicondylar in combination with patellofemoral knee arthroplasty | Heyse Tj;El-Zayat Bf;De Corte R;Scheys L;Chevalier Y;Fuchs-Winkelmann S;Labey L | KNEE | 2014 | 10.1016/S0968-0160(14)50002-6 |
| Estimating an individual's probability of revision surgery after knee replacement: a comparison of modeling approaches using a national data set | Aram P;Trela-Larsen L;Sayers A;Hills Af;Blom Aw;Mccloskey Ev;Kadirkamanathan V;Wilkinson Jm | AM J EPIDEMIOL | 2018 | 10.1093/aje/kwy121 |
| The clinical outcome of the different hemicap and unicap knee implants: a systematic and comprehensive review | Malahias Ma;Chytas D;Thorey F | ORTHOP REV | 2018 | 10.4081/or.2018.7531 |
| In vivo kinematics of a robot-assisted uni- and multi-compartmental knee arthroplasty | Watanabe T;Abbasi Az;Conditt Ma;Christopher J;Kreuzer S;Otto Jk;Banks Sa | J ORTHOP SCI | 2014 | 10.1007/s00776-014-0578-3 |
| Functional outcomes after different types of knee arthroplasty: kneeling ability versus descending stairs | Hassaballa Ma;Porteous Aj;Learmonth Id | MED SCI MONITOR | 2007 |  |
| The influence of malalignment and ageing following sterilisation by gamma irradiation in an inert atmosphere on the wear of ultra-high-molecular-weight polyethylene in patellofemoral replacements | Maiti R;Cowie Rm;Fisher J;Jennings Lm | P I MECH ENG H | 2017 | 10.1177/0954411917696112 |
| Replacement for patellofemoral arthritis | Johnson Ds;Turner Pg | KNEE | 2019 | 10.1016/j.knee.2019.10.016 |
| Mid-term survivorship and patient-reported outcomes of robotic-arm assisted partial knee arthroplasty a single-surgeon study of 1,018 knees | Burger Ja;Kleeblad Lj;Laas N;Pearle Ad | BONE JOINT J | 2020 | 10.1302/0301-620X.102B1.BJJ-2019-0510.R1 |
| Patellofemoral arthritis treated with resurfacing implant: clinical outcome and complications at a minimum two-year follow-up | Zicaro Jp;Yacuzzi C;Bonorino Ja;Carbo L;Costa-Paz M | KNEE | 2017 | 10.1016/j.knee.2017.09.003 |
| Cost effectiveness of patellofemoral versus total knee arthroplasty in younger patients | Chawla H;Nwachukwu Bu;Van Der List Jp;Eggman Aa;Pearle Ad;Ghomrawi Hm | BONE JOINT J | 2017 | 10.1302/0301-620X.99B8.BJJ-2016-1032.R1 |
| Contemporary knee arthroplasty: one fits all or time for diversity? | Beckmann J;Meier Mk;Benignus C;Hecker A;Thienpont E | ARCH ORTHOP TRAUM SU | 2021 | 10.1007/s00402-021-04042-4 |
| Mid-term survivorship and clinical outcomes of the avon patellofemoral joint replacement | Middleton Swf;Toms Ad;Schranz Pj;Mandalia Vi | KNEE | 2018 | 10.1016/j.knee.2018.01.007 |
| Advanced patellofemoral cartilage lesions in patients younger than 50 years of age: is there an ideal operative option? | Noyes Fr;Barber-Westin Sd | ARTHROSCOPY | 2013 | 10.1016/j.arthro.2013.03.077 |
| The mark coventry award: patellofemoral arthroplasty results in better range of movement and early patient-reported outcomes than tka | Odgaard A;Madsen F;Kristensen Pw;Kappel A;Fabrin J | CLIN ORTHOP RELAT R | 2018 | 10.1007/s11999.0000000000000017 |
| Comparing return to sports after patellofemoral and knee arthroplasty in an age- and sex-matched cohort | Schneider Bl;Ling Di;Kleebad Lj;Strickland S;Pearle A | ORTHOP J SPORTS MED | 2020 | 10.1177/2325967120957425 |
| Derotational osteotomy at the distal femur is effective to treat patients with patellar instability | Imhoff Fb;Cotic M;Liska F;Dyrna Fge;Beitzel K;Imhoff Ab;Herbst E | KNEE SURG SPORT TR A | 2019 | 10.1007/s00167-018-5212-z |
| Patellofemoral arthroplasty results in better time-weighted patient-reported outcomes after 6 years than tka: a randomized controlled trial | Odgaard A;Kappel A;Madsen F;Kristensen Pw;Stephensen S;Attarzadeh Ap | CLIN ORTHOP RELAT R | 2022 | 10.1097/CORR.0000000000002178 |
| Are revisions of patellofemoral arthroplasties more like primary or revision tkas | Parratte S;Lunebourg A;Ollivier M;Abdel Mp;Argenson Jn | CLIN ORTHOP RELAT R | 2015 | 10.1007/s11999-014-3756-x |
| The lubinus patellofemoral arthroplasty: a five- to ten-year prospective study - reply | Ackroyd Ce;Newman Jh | J BONE JOINT SURG BR | 2002 | 10.1302/0301-620X.84B2.0840306b |
| What is the risk of repeat revision when patellofemoral replacement is revised to tka? An analysis of 482 cases from a large national arthroplasty registry | Lewis Pl;Graves Se;Cuthbert A;Parker D;Myers P | CLIN ORTHOP RELAT R | 2019 | 10.1097/CORR.0000000000000541 |
| The evolution and role of patellofemoral joint arthroplasty the road less travelled, but not forgotten | Roussot Ma;Haddad Fs | BONE JOINT RES | 2018 | 10.1302/2046-3758.712.BJR-2018-0303 |
| Patellar tracking before and after a patellofemoral joint replacement (depuy sigma) - a cadaveric study using computer navigation | Sussmann P;Heinert G;Wolfgang K;Preiss S | SWISS MED WKLY | 2011 |  |
| Patellofemoral arthroplasty | Smith A;Lucas D | J BONE JOINT SURG AM | 2003 | 10.2106/00004623-200308000-00029 |
| Patellofemoral arthroplasty - reply | Mont Ma;Haas S | J BONE JOINT SURG AM | 2003 | 10.2106/00004623-200308000-00030 |
| Patellofemoral arthroplasty (vol 9, e15, 2019) | Odgaard | JBJS ESSENT SURG TEC | 2020 | 10.2106/JBJS.ST.ER.18.00094 |
| Early advantage of patellofemoral arthroplasty is not age dependent | Lehoczky G;Mumme M;Jakob M;Pagenstert G | SWISS MED WKLY | 2017 |  |
| The lubinus patellofemoral arthroplasty (vol 83b, pg 696, 2001) | Tauro B;Ackroyd Ce;Newman Jh;Shah Na | J BONE JOINT SURG BR | 2002 |  |
| The lubinus patellofemoral arthroplasty: a five- to ten-year prospective study | Roach R | J BONE JOINT SURG BR | 2002 | 10.1302/0301-620X.84B2.0840307 |
| Patellofemoral arthroplasty a 3-year to 9-year follow-up-study | Arciero Ra;Toomey He | CLIN ORTHOP RELAT R | 1988 |  |
| The lubinus patellofemoral arthroplasty: a five- to ten-year prospective study | Coleridge S | J BONE JOINT SURG BR | 2002 | 10.1302/0301-620X.84B2.0840306a |
| Indications, contraindications and pitfalls of patellofemoral arthroplasty | Leadbetter Wb;Seyler Tm;Ragland Ps;Mont Ma | J BONE JOINT SURG AM | 2006 | 10.2106/JBJS.F.00856 |
| A prospective matched-pair analysis of two different inlay trochlear designs for patellofemoral arthroplasty | Martinho T;Cotic M;Pogorzelski J;Imhoff A | SWISS MED WKLY | 2022 |  |
| Patellofemoral arthroplasty in a young woman with anterior knee disabling pain and developmental hip dysplasia | Spina M;Bagnis F | MINERVA ORTOP TRAUMA | 2018 | 10.23736/S0394-3410.18.03882-1 |
| Patellofemoral arthroplasty: the other unicompartmental knee replacement | Walker T;Perkinson B;Mihalko Wm | J BONE JOINT SURG AM | 2012 | 10.2106/JBJS.L.00539 |
| Letter to the editor: short-term revision risk of patellofemoral arthroplasty is high: an analysis from eight large arthroplasty registries | Van Jonbergen Hpw;Kleinlugtenbelt Yv | CLIN ORTHOP RELAT R | 2021 | 10.1097/CORR.0000000000001717 |
| Patellofemoral replacement | Blazina Me;Fox Jm;Delpizzo W;Broukhim B;Ivey Fm | CLIN ORTHOP RELAT R | 1979 |  |
| The midterm results of a cohort study of patellofemoral arthroplasty from a non-designer center using an asymmetric trochlear prosthesis (vol 26, pg 1348, 2019) | Rammohan R;Gupta S;Lee Pyf;Chandratreya A | KNEE | 2020 | 10.1016/j.knee.2020.02.009 |
| Patellofemoral replacement: the third compartment | Minas T | ORTHOPEDICS | 2008 | 10.3928/01477447-20080901-24 |
| Reply to the letter to the editor: short-term revision risk of patellofemoral arthroplasty is high: an analysis from eight large arthroplasty registries | Lewis Pl | CLIN ORTHOP RELAT R | 2021 | 10.1097/CORR.0000000000001718 |
| Patellofemoral replacement: the third compartment | Argenson Jn | ORTHOPEDICS | 2003 |  |
| Custom patellofemoral replacement in the presence of trochlear dysplasia | Grelsamer Rp | J BONE JOINT SURG AM | 2007 | 10.2106/00004623-200702000-00040 |
| Custom patellofemoral replacement in the presence of trochlear dysplasia - reply | Sisto Dj;Sarin Vk | J BONE JOINT SURG AM | 2007 | 10.2106/00004623-200702000-00041 |
| Patellofemoral replacement polymer stress during daily activities: a finite element study | Morra Ea;Greenwald As | J BONE JOINT SURG AM | 2006 | 10.2106/JBJS.F.00585 |
| Outcomes of patellofemoral replacement in total knee arthroplasty using meticulous techniques | Clyburn Ta;Weitz-Marshall A;Ambrose Cm;Ursua V | ORTHOPEDICS | 2007 | 10.3928/01477447-20070201-13 |
| Corr insights (r): what is the risk of repeat revision when patellofemoral replacement is revised to tka? An analysis of 482 cases from a large national arthroplasty registry | Hallstrom Br | CLIN ORTHOP RELAT R | 2019 | 10.1097/CORR.0000000000000583 |
